# Transcriptome Profiling of HCT-116 Colorectal Cancer Cells with RNA Sequencing Reveals Novel Targets for Polyphenol Nano Curcumin

**DOI:** 10.3390/molecules27113470

**Published:** 2022-05-27

**Authors:** Hewa Jalal Azeez, Francesco Neri, Mohammad Ali Hosseinpour Feizi, Esmaeil Babaei

**Affiliations:** 1 Department of Biology, School of Natural Sciences, University of Tabriz, Tabriz 51368, Iran; hewa.azeez@tabrizu.ac.ir; 2 Life Sciences and Systems Biology Department, University of Torino, 10124 Torino, Italy; francesco.neri@unito.it (F.N.); pourfeizi@tabrizu.ac.ir (M.A.H.F.)

**Keywords:** gemini curcumin, colorectal cancer, RNA sequencing, PPI network, differentially expressed genes

## Abstract

Colorectal cancer is one of the leading causes of cancer-related deaths worldwide. The gemini nanoparticle formulation of polyphenolic curcumin significantly inhibits the viability of cancer cells. However, the molecular mechanisms and pathways underlying its toxicity in colon cancer are unclear. Here, we aimed to uncover the possible novel targets of gemini curcumin (Gemini-Cur) on colorectal cancer and related cellular pathways. After confirming the cytotoxic effect of Gemini-Cur by MTT and apoptotic assays, RNA sequencing was employed to identify differentially expressed genes (DEGs) in HCT-116 cells. On a total of 3892 DEGs (padj < 0.01), 442 genes showed a log2 FC >|2| (including 244 upregulated and 198 downregulated). Gene ontology (GO) enrichment analysis was performed. Protein–protein interaction (PPI) and gene-pathway networks were constructed by using STRING and Cytoscape. The pathway analysis showed that Gemini-Cur predominantly modulates pathways related to the cell cycle. The gene network analysis revealed five central genes, namely GADD45G, ATF3, BUB1B, CCNA2 and CDK1. Real-time PCR and Western blotting analysis confirmed the significant modulation of these genes in Gemini-Cur-treated compared to non-treated cells. In conclusion, RNA sequencing revealed novel potential targets of curcumin on cancer cells. Further studies are required to elucidate the molecular mechanism of action of Gemini-Cur regarding the modulation of the expression of hub genes.

## 1. Introduction

With over 1.8 million new cases and around 800,000 fatalities recorded in 2018, colorectal cancer (CRC) is considered as one of the most common malignancies, worldwide [1,2]. In recent years, early age onset of CRC cases have increased dramatically [3]. Conventional chemotherapy, radiotherapy and surgery provide effective local control of colon cancer. However, serious side effects and resistance to therapies over time decrease the survival rate of patients [4]. Despite recent dramatic advances in early diagnosis and treatment, there still remains an unmet need to palliate CRC symptoms, develop novel therapeutic strategies with lower side effects, and prolong the overall survival of the patients [5]. Numerous studies have shown that different cellular pathways including cell cycle, cell proliferation, drug resistance, apoptosis and metastasis are modulated in CRC. Furthermore, recent findings show that metabolic pathways such as glycolysis can influence the apoptotic potential of cancer therapeutics. Therefore, therapies targeting various targets in cancer cells have recently raised more interest [6,7].

Herbal compounds and their derivatives have attracted huge attention and become a prominent contribution in novel drug discovery programs by exhibiting their therapeutic effects through a multi-targeted approach, which is a characteristic that is highly desirable in cancer malignancies [6]. Phytochemicals may illustrate their antitumor properties through promoting apoptosis, suppressing the cell cycle, inhibiting angiogenesis and regulating antioxidant activities. Moreover, numerous naturally bioactive compounds have been shown to modulate immune checkpoints and affect the activities of immune cells including T and B cells, Treg cells and NK cells [8]. More interestingly, these natural products have gained competing interests due to the absence of toxicity and harmful side effects commonly associated with current therapies [9].

Curcumin is a polyphenolic derivative of turmeric known to have dramatic anticancer effects on cancer cells rather than normal ones. It has been demonstrated that curcumin exerts its toxic effects through modulation of the function of multiple genes including apoptotic, metastatic, cell proliferation and transcription factors [10]. Studies reported that curcumin modulates cellular pathways involved in cancer pathogenesis including NF-kB, MAPK, PTEN, P53 and wnt [11]. Despite these tempting advantages of curcumin, the poor bioavailability limits its exploitation as a therapeutic compound [12]. Our team has recently formulated and characterized a nano-based encapsulated curcumin, gemini curcumin (Gemini-Cur), with significant anticancer effects on ovarian, gastric, breast and colorectal cancer [13,14,15,16]. Briefly, gemini surfactant nanoparticles belong to a surfactant family with two identical structures that are linked by a rigid or flexible spacer that could harbor and deliver drugs and genes into the cells and tissues. Gemini curcumin nanoparticles are spherical and well-dispersed vesicles that easily enter the cancer cells [15].

Exploration of the genes with abnormal expression during the treatment of colon cancer with Gemini-Cur is essential to provide a deeper understanding of the mechanisms involved. Because regulatory genes are affected by dietary compounds, the ability of curcumin to modulate the transcriptome profile has attracted much attention [8,17]. Based on our recent findings on the significant toxic properties of Gemini-Cur on cancer cells, here, we employed RNA sequencing and bioinformatics analysis to identify the key genes and related pathways modulated in colorectal HCT-116 cells treated with Gemini-Cur. The data of the current study help us to determine top Differentially Expressed Genes (DEGs) as possible cellular targets and figure out potential biological pathways in colon cancer that are modulated by curcumin.

## 2. Materials and Methods

### 2.1. In Vitro Studies

#### 2.1.1. Cell Culture and Reagents

The colorectal cancer HCT-116 cell line was purchased from the Iranian national cell bank (Pasteur institute, Tehran, Iran). The cells were cultured in Dulbecco’s Modified Eagle’s Medium (DMEM, Sigma-Aldrich, St. Louis, MO, USA) supplemented with 10% (*v*/*v*) fetal bovine serum and 1% (*v*/*v*) penicillin–streptomycin solution (both from GIBCO, USA) at 37 °C in a humidified environment with 5% CO_2_. Curcumin (CAS Number 458-37-7; Sigma-Aldrich, USA) and mPEG urethane gemini surfactant nanoparticles were a kind gift from the Institute for Color Science and Technology, Tehran, Iran.

#### 2.1.2. Synthesis of Gemini-Cur nanoparticles

Gemini-Cur nanoparticles were prepared by a nanoprecipitation method previously reported by our lab [16]. Briefly, we added 6 mg of Cur and 100 mg of gemini surfactants to 3 mL of methanol. Then, the solution was diluted twice in PBS under gently stirring condition, and the methanol was evaporated by using a rotary evaporator. The remaining solution was passed through a 0.22 µM syringe filter to remove possible contaminations and stored at 4 °C until use.

#### 2.1.3. Gemini-Cur Treatments

We have previously reported the IC50 values for Gemini-Cur on HCT-116 cells. Furthermore, we demonstrated that Gemini-Cur modulates the cell cycle and induces apoptosis in HCT-116 cells compared to controls [14]. To further confirm cellular toxicity on the cells treated with Gemini-Cur, we used ethidium bromide/acridine orange (EB/AO) staining. Briefly, the cells were left untreated or treated with Gemini-Cur at an IC50 dose onto 6-well plates. After 24, 48 and 72 h, the cells were detached by trypsin (0.25%; Sigma-Aldrich, USA) and transferred to glass slides. Staining solution (1 µL) containing 100 µg/mL acridine orange and 100 µg/mL ethidium bromide (Sigma-Aldrich, USA) was added to a suspension of HCT-116 cells. The cells were visualized under fluorescence microscopy (RX50, LABEX, England), and representative photographs were taken for further qualitative analysis. Fluorouracil (5-FU) apoptotic images were also provided as positive control.

For RNA sequencing, the cells were seeded on 6-well plates for 24 h and subsequently treated with Gemini-Cur. After 24 h, the cells were processed for RNA sequencing.

#### 2.1.4. RNA Extraction and Preparation

According to the protocol of TRIzol reagent (Thermo Fisher Scientific, USA), total RNA was extracted from treated and non-treated HCT-116 cells. A total of six samples including three controls and three treated cells were incorporated in the study. After validating the integrity and purity (NanoDropTM, ThermoFisher, USA), all RNAs were treated with RNase-free DNase I to remove any DNA contamination. Then, RNAs were transferred to GeneTegra-RNA tubes (GenTegra Co., Seoul, Korea), dried in a freezer dryer (Sartorius Co., Germany) and sent to Macrogen Co., for sequencing (Macrogen Co., Seoul, Korea).

#### 2.1.5. Library Construction and RNA Sequencing

Approximately 1 µg of RNA from each sample was used to generate RNA-Seq cDNA libraries for sequencing using the TruSeq RNA Sample Prep Kit v2 (Illumina, Inc., San Diego, CA, USA). Sample preparation followed the manufacturer’s protocol with a workflow that included isolation of polyadenylated RNA molecules using poly-T oligo-attached magnetic beads, enzymatic RNA fragmentation, cDNA synthesis, ligation of bar-coded adapters, and PCR amplification. Ambion External RNA Controls Consortium (ERCC) RNA Spike-In Control Mix 1 (Life Technologies Corporation, Carlsbad, CA, USA) was added to the samples. The amplified cDNA fragments were sequenced using a HiSeq 2000 sequencing system (Illumina, San Diego, CA, USA). Finally, sequencing data were converted to raw data in FASTQ format utilizing illumina package bcl2fastq.

### 2.2. Bioinformatics Studies

#### 2.2.1. Quality Assessment of RNA-seq Data, Mapping and Read Annotation

All processing and analysis on raw data were performed using Ubuntu 20.00 (64-bit) and open-source software available through the R/Bioconductor. After check and quality control of paired-end reads with the final version of MultiQC (https://github.com/ewels/MultiQC, 25 January 2022) and Trimmomatic (http://www.usadellab.org/cms/?page=trimmomatic, 25 January 2022), the remaining reads as clean reads were mapped to the genome reference GRCh37 (hg19) using the star (https://github.com/alexdobin/STAR/releases, 10 January 2022) package, and mapping efficiencies accounted for 98.50%. The counting of transcripts was also performed with Htseq-count (https://htseq.readthedocs.io/en/release_0.11.1/count.html, 10 January 2022).

#### 2.2.2. Normalization of Read Counts, Differentially Expression Analysis (DEA) and Network Construction

In order to normalize and perform differential expression analysis on counts, the standard Bioconductor RNA-seq workflow (DESeq2) was used to detect differentially expressed genes (DEGs). The distribution of expression values across all samples (normal and treatment) before and after normalization was applied to ensure that expression values were similar across normalized counts. The PPI network was constructed using STRING (*p*-value: 1.0 × 10^−164^), which resulted in 2736 interactions between 180 nodes based on a confidence score of 0.007. In order to detect the key parameters, the interaction pairs of the network obtained from STRING were visualized by Cytoscape (Version 3.6) with a cut-off value for BC > 0 and K > 8. After analyzing PPI network modules with MCODE, generally, 10 modules obtained. Three significant modules were identified with an MCODE score ≥ 3 and nodes ≥ 3. In order to conduct a gene-pathway annotated network, 300 upregulated (padj < 0.01, log2 FC > 2) and downregulated (padj < 0.01, log2 FC < −2) genes were mapped to 117 KEGG pathways. Then, an annotated network was constructed for significant KEGG pathways by using Cytoscape (Version 3.6). Gene ontology (GO) was conducted using the enrichR/Bioconductor package to clarify which biological categories (CC, MF, BP) the DEGs are enriched.

#### 2.2.3. Functional Enrichment and Gene Ontology Analysis

Gene ontology (GO) was conducted using EnrichR web tool to clarify which GO term and fanatical biology categories (CC: cellular component, MF: molecular function, BP: biological process) the DEGs are enriched.

### 2.3. Exploration and Validation of CRC-Related Genes Based on Real-Time PCR and Western Blotting

To further validation of the RNA sequencing data generated by the above-mentioned parameters, the differential expression of top five genes including two upregulated (GADD45G and ATF3) and three downregulated genes (BUB1B, CCNA2 and CDK1) were evaluated on all treated and non-treated samples in both mRNA and protein levels. Total RNAs were firstly converted to cDNA using an c (AddBio Co., Seoul, Korea). According to the manufacturer’s instructions, quantitative PCR analysis was performed by employing Add SYBER Master kit (AddBio Co., Seoul, Korea) on the CFX96 thermal cycler (Bio-Rad Co., Hercules, CA, USA). All the primers (Table 1) were designed by Gene Runner version 6 (http://generunner.net, 1 September 2020), and β-actin was used as internal control. The quantification of expression levels was studied by the 2^−∆∆Ct^ method. Furthermore, melting curves were run to confirm the specificity and consistency of the products.

Total protein was extracted from all samples using 500 µL of lysis buffer (Tris-HCl pH 8, 0.08 g NaCl, 0.003 g EDTA, 0.025 g sodium deoxycholate, 0.01 g SDS, and 1% NP40 enriched with an anti-protease cocktail). Thereafter, 10 µg of protein was electrophoresed using 10% SDS-PAGE at 120 V for 45 min and then transferred onto polyvinylidene difluoride membranes at 120 V for 1.5 h. The membranes were incubated with appropriate primary antibodies (all from Santa Cruz Biotechnology, Dallas, TX, USA) at 4 °C overnight. After three-time PBS wash, the membranes were incubated with appropriate HRP-conjugated secondary antibodies (Cat no: sc-516102 and sc-2357; Santa Cruz Biotechnology, USA) for 1 h at room temperature. The immunoblots were detected on X-ray films using chemiluminescence ECL solution (Bio-Rad, Herecules, CA, USA). β-actin (Cat no: sc-47778; Santa Cruz Biotechnology, Inc.) was considered as internal control for normalization.

## 3. Results

### 3.1. Suppressive Effect of Gemini-Cur on HCT-116 Cells

**Figure 1:**
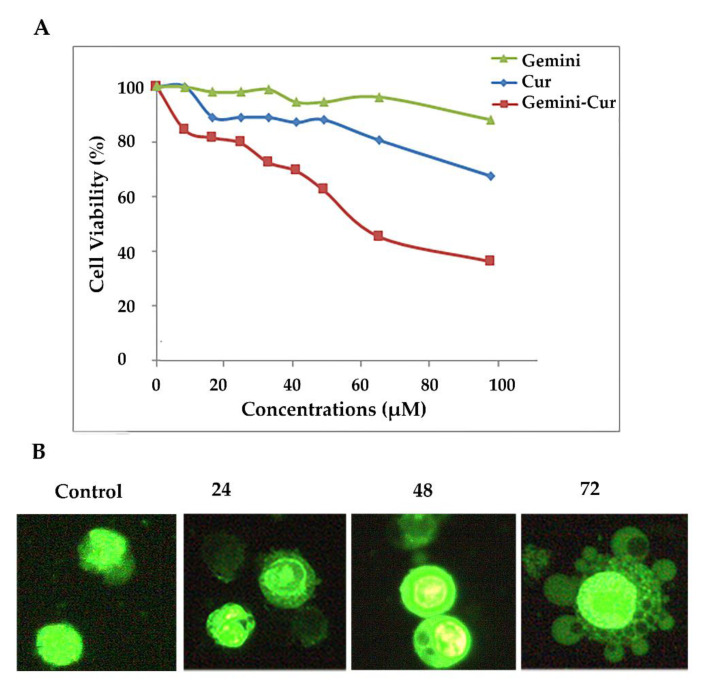
Gemini-Cur affects the proliferation of HCT-116 cells. (**A**): Gemini-Cur suppresses HCT-116 cells proliferation in a time- and dose-dependent manner. (**B**): Acridine orange/ethidium bromide staining also illustrated cells with apoptotic characteristics including membrane shrinkage and nuclear fragmentation in different incubation times (24, 48 and 72 h, Magnification ×400). Gemini: gemini surfactant nanoparticles; Cur: curcumin; Gemini-Cur: gemini curcumin.

Based on our previous reports, gemini surfactant nanoparticles significantly increase the cellular uptake of curcumin and suppress the proliferation of HCT-116 cells through the induction of apoptosis. Accordingly, Gemini-Cur significantly increases the proportion of SubG1 cells and induces apoptosis in HCT-116 cells compared to the non-treated group. Our Hoechst staining also illustrated the morphological characteristics of membrane shrinkage and nuclear fragmentation of HCT-116 cells. Here, we further confirm that Gemini-Cur modulates the growth of HCT-116 cells compared to void curcumin with IC50 value of 51.50 for 24 h (Figure 1A). In accordance with our previous work, acridine orange/ethidium bromide staining revealed that Gemini-Cur instigates apoptosis in colorectal HCT-116 cells. As Figure 1B shows, there is no significant apoptosis in non-treated cells (control). In contrast, the nucleus in dead cells reveals a condensed and granular forms with green–light orange fluorescence.

### 3.2. Raw Data Statistics and Quality Assessment

**Figure 2:**
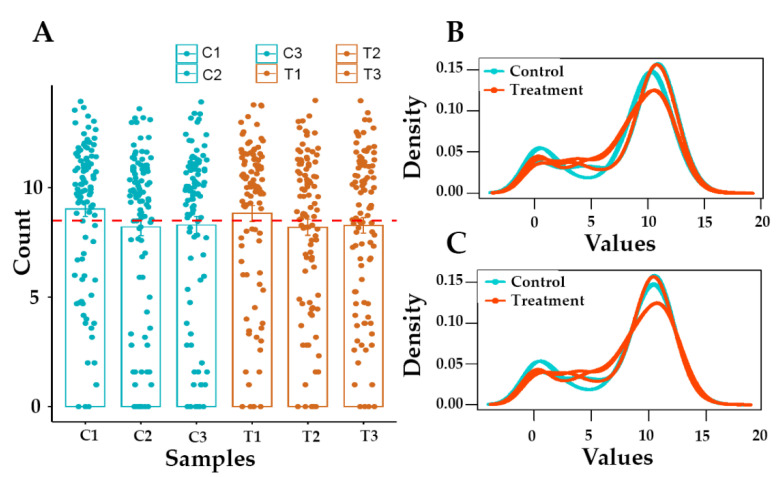
Distribution of gene counts. As shown in bar plot (**A**), more than 8 million reads are produced for each sample. According to density plot, raw read Counts (log2 (counts + 1)) are not non-normalized distributed (**B**). The density plot of normalized count (log2 (normalized counts)) per sample is shown in plot (**C**). C1-3: controls; T1-3: Treated cells with Gemini-Cur.

Three replicates of treated samples and corresponding controls were subjected to RNA sequencing. On average, more than 40 million reads per sample were recorded (Figure 2A). The proportion of bases with high quality (Q30) was more than 90, indicating that the quality of RNA sequencing was proper for further analysis. After quality control and the removal of adaptors, more than 8 million reads were produced for each sample (Figure 2B).

### 3.3. Identification of Differentially Expressed Genes (DEGs)

**Figure 3:**
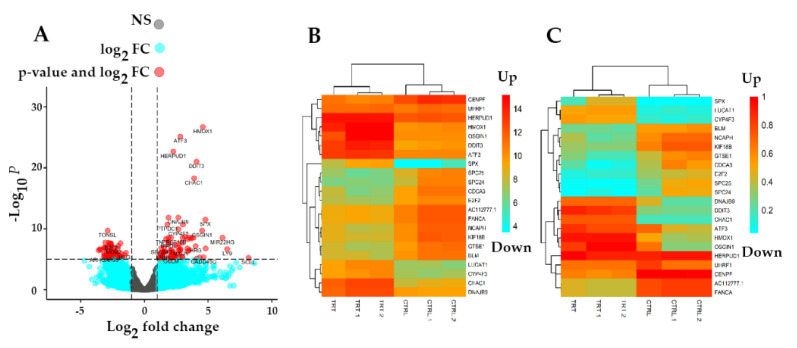
Normalization of raw read counts and Differential Expression Analysis (DEA). The volcano plot (**A**) of non-significant DEGs (colored in dark gray), significant DEGs (padj < 0.01) (colored in light blue), and up/downregulated genes (Colored in red). The x-axis represents the log2 FC, while the y-axis represents the statistical significance for each gene based on −log10 (*p* value). Heatmap of top 22 up/downregulated genes associated with non-normalized counts (**B**). Heatmap of top 22 up/downregulated DEGs associated with normalized counts (**C**). From the heatmap (downregulated genes are colored in light blue and upregulated genes are colored in orange in both plots). NS: non-significant; FC: fold change.

In order to determine DEGs in treated samples versus controls, the standard Bioconductor RNA-seq workflow (DESeq2) was employed. In total, 3892 DEGs (padj < 0.01) including 244 upregulated (padj < 0.01, log2 FC > 2) and 198 downregulated (padj < 0.01, log2 FC < −2) genes were obtained in this study (Supplementary Materials). As Figure 3A shows, all genes were categorized in non-significant (dark gray) and significant (light blue) DEGs as well as top down/upregulated genes (red). To further illustrate top-modulated genes, a heatmap with a color range of blue (down) to red (up) was employed for non-normalized (Figure 3B) and normalized genes (Figure 3C). The heatmap demonstrates the differential expression of genes in Gemini-Cur-treated cells in comparison with non-treated samples. Subsequently, a list of top ten up and downregulated DEGs (padj < 0.01) was obtained with padj < 0.01, log2 FC > 2 and padj < 0.01, log2 FC < −2, respectively (Table 2).

### 3.4. Exploration of DEGs in Protein–Protein Interaction (PPI) Network and Subnetworks (Modules)

**Figure 4:**
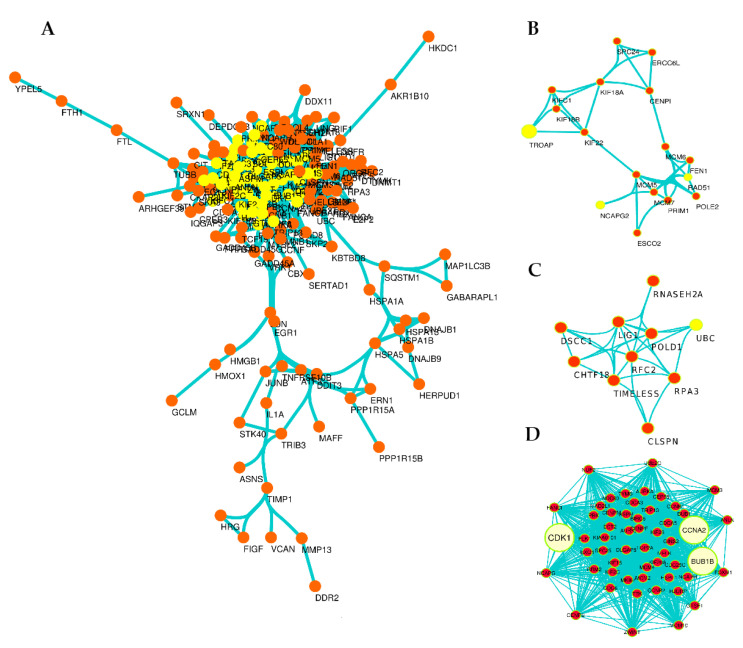
Overview of PPI network and subnetworks (modules). The PPI network (**A**); the yellow colors represent key nodes (highest BC and K) in PPI networks. Generally, 10 modules were obtained from the main network and module 1 (**D**), module 2 (**B**) and module 3 (**C**) are significant modules. Module 1 with the score of 47.057 and 54 nodes is the more significant module, covering three genes CDK1, CCNA2, and BUB1B with the highest BC and K in the PPI network and with the highest score in the MCODE plug-in.

To find out the potential interactions at the protein level, DEGs were mapped in STRING, and the PPI network (Figure 4A) was constructed for top 300 DEGs (up/downregulated genes). The significant pairs of the network (*p*-value: 1.0 × 10^−164^) in 2736 interactions between 180 nodes based on confidence score (0.007) were visualized by Cytoscape software and the network analyzer plug-in with a cut-off value for BC > 0 and K > 8. After analyzing PPI network, subnetworks (modules) with a MCODE plug-in of Cytoscape were extracted, and generally, 10 modules obtained. Three significant modules were identified with MCODE score ≥ 3 and nodes ≥ 3 (Figure 4B–D). CDK1, CCNA2, and BUB1B with the highest BC and K in the PPI network and with the highest score in the MCODE plug-in were significantly identified in Module 1 (Figure 4D and Table 3). Accordingly, it is clearly obvious that three of the DEGs (CDK1, CCNA2, and BUB1B) in the PPI network and module 1 are the genes that interact the most with others in the PPI network.

### 3.5. KEGG Pathway Analysis of DEGs

For the characterization of DEGs, we performed pathway enrichment analysis in KEGG. In total, 300 upregulated (padj < 0.01, log2 FC > 2) and downregulated (padj < 0.01, log2 FC < −2) genes were mapped to 117 KEGG pathways. In order to identify which gene is associated with biological pathways, a gene-pathway annotated network was created using 300 DEGs (up/downregulated genes) alongside the most significant KEGG pathways using Cytoscape software.

**Figure 5:**
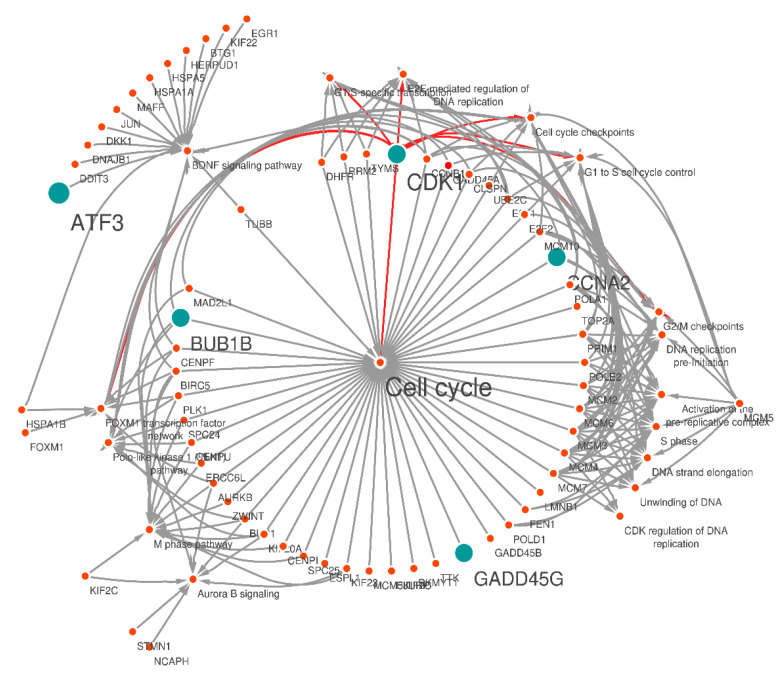
Overview of the gene-pathway network (annotated network) created by cystoscope. The blue nodes represent the CRC candidate genes connected with the pathways and other nodes in the network. The orange nodes represent other pathways in connection with CRC genes. According to this network, it can be demonstrated that CDK1 is involved in all significant first-level pathways; it is associated with related genes in each of the pathways and with other most significant CRC-related genes (CCNA2, BUB1B, GADD45G, ATF3). The dominant pathway is cell cycle with padj 1.82 × 10^−34^.

Generally, the gene-pathway annotated network showed that DEGs were significantly enriched in the cell cycle, E2F-mediated regulation of DNA replication, G1/S-specific transcription, cell cycle checkpoints, G2/M checkpoints, FOXM1 transcription factor network, and G1 to S cell cycle control (Table 4). Surprisingly, a vital gene of our study, CDK1, was significantly enriched in all significant pathways that may act as a bridge between both the CRC-related genes (CCNA2, BUB1B, GADD45G and ATF3) and other gene-pathway annotated network genes (Figure 5).

### 3.6. Functional Enrichment and Gene Ontology (GO) Analysis

To better understand the molecular role of selected genes involved in the suppressive effect of Gemini-Cur on HCT-116 cells, DEGs were mapped in the GO database using an online web tool (https://maayanlab.cloud/Enrichr, 25 January 2021), and a threshold of Padj < 0.05 and gene counts > 5 was considered. Here, gene ontology was performed for 198 downregulated and 244 upregulated genes separately, and the status of all five selected genes in all three categories (BP; biological process, MF; molecular function, and CC; cellular component) was assessed.

Interestingly, the GO results of the integrated group showed that upregulated ATF3 is significantly enriched in BP category including response to endoplasmic reticulum stress, regulation of transcription from RNA polymerase II promoter in response to stress, and MF category with protein hetero-dimerization activity. In addition, GADD45G was enriched in the regulation of p38MAPK cascade as well as control of the p38MAPK pathway, both in BP process (Table 5). In contrast, downregulated genes (CCNA2, BUB1B, and CDK1) were involved in almost all three categories and different cellular cascades.

#### In Vitro Validation Study by Real-Time PCR and Western Blotting

**Figure 6:**
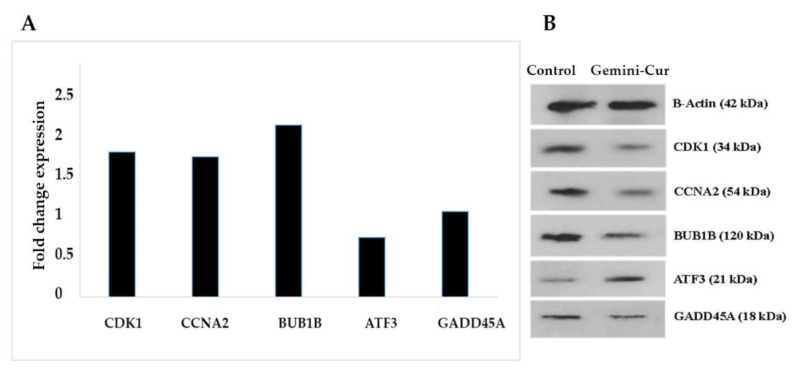
Validation of the expression of selected genes by real-time PCR and Western blotting. Nearly all genes except GADD45A validate findings from RNA seq. data. (**A**) The data show relative expression of genes compared to non-treated controls. (**B**) Blots illustrate protein expression in control and treated cells.

To further validation of the RNA sequencing data generated by the above-mentioned parameters, the differential expression of top five genes including two upregulated (GADD45G and ATF3) and three downregulated (BUB1B, CCNA2 and CDK1) were evaluated on all treated and non-treated samples in mRNA and protein levels. As Figure 6A shows, the expression of BUB1B, CCNA2 and CDK1 is significantly down-expressed in treated cells. Accordingly, the data illustrated that ATF3 (*p* value < 0.05) and GADD45G are upregulated in Gemini-Cur treated cells, although this was not significant for GADD45G. These modulations were confirmed in protein level as shown in Figure 6B.

## 4. Discussion

Despite recent advances in early diagnosis and proper treatments, colorectal cancer (CRC) is still considered as the third leading cause of cancer-related deaths worldwide [1,2]. Gemini-Cur is one of the latest nano-formulations of curcumin with a significant anticancer effect that has recently been developed by our group [15]. We have already reported that Gemini-Cur inhibits the proliferation of different cancer cells through the induction of apoptosis [13,14,15,16]. Due to the limited information on the global effect of curcumin on transcriptome profiling, we employed RNA sequencing to uncover the differentially expressed genes and cellular pathways that are affected by Gemini-Cur in colorectal cancer cells. Here, our data not only confirm the suppressive effect but also demonstrate that numerous genes in different cellular pathways are modulated by Gemini-Cur in HCT-116 cells.

Along with all the steps of quality control and RNA-seq raw data normalization, the DEA (Differentially Expressed Analysis) process introduced about 3892 genes as DEGs (padj < 0.01) with 244 upregulated (padj < 0.01, log2 FC ≥ 2), and 198 downregulated genes (padj < 0.01, log2 FC ≤ −2). The PPI network was also created by Cytoscape to better figure out the possible correlations between DEGs. Consequently, three of the top significant downregulated genes including cyclin-dependent kinase 1 (CDK; padj = 1.08 × 10^−06^, log2 FC = −3.15), cyclin A2 (CCNA2; padj = 2.87 × 10^−05^, log2 FC = −3.056) and BUB1 mitotic checkpoint serine/threonine kinase B (BUB1B; padj = 2.72 × 10^−06^, log2 FC = −2.99) as well as two upregulated genes including growth arrest and DNA damage inducible gamma (GADD45G; padj = 1.47 × 10^−06^, log2 FC = 4) and activating transcription factor 3 (ATF3; padj = 1.03 × 10^−19^, log2 FC = 4) were selected from top ten DEG lists for further validation of RNA sequencing results.

Numerous reports have shown that ATF3 and GADD45G play critical roles in cancer. Inoue et al. illustrated that the ATF3 transcription factor inhibits the migration and invasion of HCT-116 cells. ATF3 is also involved in the process of cellular stress response [18]. Here, RNA sequencing and experimental studies show that transcription factor ATF3 as a key regulator of cellular stress response is upregulated in cancer cells after treatment with Gemini-Cur. These findings demonstrate increasingly the potential of ATF3 as a therapeutic candidate in colorectal cancer. In another work, Guo et al. demonstrated that the overexpression of GADD45G acts as tumor suppressor in AML [19]. Although RNA sequencing showed that GADD45G is upregulated in treated cells, this overexpression was not detected in PCR and Western blotting. This contradictory finding may be due to the presence of different RNA reads including RNAs from paralogous genes and pseudogenes. However, PCR and Western blotting only detect main predominant variant of GADD45G.

Accordingly, CCNA2, BUB1B, and CDK1 genes have been downregulated in Gemini-Cur treated cells versus the non-treated group. According to the gene-pathway annotated network, it is revealed that CDK1 is involved in the cell cycle and associated with other CRC-related genes (CCNA2, BUB1B, GADD45G, ATF3). CDK1 contributes to the cell proliferation, apoptosis, and cell migration [20,21]. It was shown that the upregulation of CDK1 leads to poor prognosis in patients with CRC [22]. Zhu et al. and Thoma et al. indicated that the downregulation of CDK1 inhibited fluorouracil-resistant CRC cell proliferation [23,24].

Regarding the BUB1B and CCNA2, both genes have been reported as upregulated genes in CRC [25,26,27]. Gan et al. indicated that the CCNA2 knockdown could significantly suppress CRC cell growth by impairing cell cycle progression and inducing cell apoptosis [20]. Ding et al. also reported that the upregulation of BUB1B, CDK1, and CCNA2 genes contribute to the progression of tumor growth and metastasis of CRC [28]. These results further validate our analysis methods and the accuracy of RNA sequencing for exploring the novel top up/downregulated genes in different conditions.

The joint cooperation of GADD45G and CDK1 in the p53 activity regulation pathway is another attractive result of this study. GADD45G plays a role in the activation of S and G2/M checkpoints during p53-related DNA damage responses [29,30]. The modulatory effect of Gemini-Cur on these genes may highlight their collaborative role in cell stress response.

## 5. Conclusions

Taken together, our data show that Gemini-Cur comprehensively modulates gene expression in colorectal cancer HCT-116 cells. Gene ontology annotations related to DEGs found here include DNA-dependent ATPase (MF) during metabolic processes (BP) in nucleus (CC). Further analysis also demonstrated that Gemini-Cur dominantly affects cell cycle-related pathways. These RNA sequencing data greatly expand our understanding of the molecular and cellular targets of curcumin in cancer. We also reported novel potential targets for curcumin as listed in the top ten DEGs. Further investigations on the top up/downregulated genes especially in different cancer cell lines and non-cancerous controls will facilitate the findings of curcumin targets in colon cancer.

## Figures and Tables

**Table 1:** Details of primers used in real-time PCR. F: forward; R: reverse.

Gene	Sequence (5′→3′)	PCR Product
CDK1	F: 5-AGCCGGGATCTACCATACC-3 R: 5-CATGGCTACCACTTGACCTG-3	126
CCNA2	F: 5-GGACAAAGCTGGCCTGAATC-3 R: 5-CTGTTGTGCATGCTGTGGTG-3	116
BUB1B	F: 5-CAATTCCAAGCTCGAGTGTC-3 R: 5-GATGATTGGAGCTCTTGCTG-3	146
GADD45G	F: 5-GTCAGCCAAAGTCTTGAACG-3 R: 5-GCACTATGTCGATGTCGTTC-3	145
ATF3	F: 5-CAGCACCTTGCCCCAAAATC -3 R: 5-TGGATGGCAAACCTCAGCTC-3	171
β-actin	F: 5-CAGCACCTTGCCCCAAAATC -3 R: 5-TGGATGGCAAACCTCAGCTC-3	184

**Table 2:** The list of top 10 up/downregulated Differentially Expressed Genes (DEGs) based on RNA-seq data analysis. The DEGs (padj < 0.01) between control and treatment groups are shown with top 10 upregulated genes (padj < 0.01, log2 FC > 2), and top 10 downregulated genes (padj < 0.01, log2 FC < −2). Padj: adjusted *p* value.

Gene Symbol	STATUS	Base Mean	log2 Fold Change	lfcSE	Stat	*p* Value	padj
HMOX1	Up	14082.63	4.581563	0.393634	11.63913	2.61 × 10^−31^	9.29 × 10^−27^
DDIT3	Up	7029.257	4.083832	0.394679	10.34723	4.31 × 10^−25^	7.68 × 10^−21^
ATF3	Up	5160.583	2.807268	0.27917	10.05577	8.66 × 10^−24^	1.01 × 10^−19^
CHAC1	Up	2994.215	3.896157	0.400672	9.724061	2.38 × 10^−22^	2.12 × 10^−18^
HERPUD1	Up	12540.28	2.267609	0.254122	8.92332	4.53 × 10^−19^	3.23 × 10^−19^
SPX	Up	260.3101	4.781735	0.578882	8.260295	1.45 × 10^−16^	8.64 × 10^−13^
DNAJB9	Up	2736.219	2.665123	0.342986	7.770348	7.83 × 10^−15^	3.99 × 10^−11^
OSGIN1	Up	13215.95	4.534403	0.586017	7.737659	1.01 × 10^−14^	4.51 × 10^−11^
LUCAT1	Up	608.1075	3.02517	0.396356	7.632456	2.30 × 10^−14^	9.13 × 10^−11^
CYP4F3	Up	547.2113	2.671816	0.369558	7.22977	4.84 × 10^−13^	1.57 × 10^−09^
TONSL	Down	2059.716	−2.89597	0.395525	−7.32183	2.45 × 10^−13^	8.72 × 10^−10^
MCM4	Down	5519.294	−2.8152	0.421581	−6.67772	2.43 × 10^−11^	4.5 5× 10^−08^
AC112777.1	Down	221.6533	−3.06376	0.459517	−6.66735	2.60 × 10^−11^	4.64 × 10^−08^
FANCA	Down	1932.18	−2.67477	0.406894	−6.57362	4.91 × 10^−11^	7.47 × 10^−08^
E2F2	Down	629.9052	−2.62378	0.402383	−6.52061	7.00 × 10^−11^	9.25 × 10^−08^
BLM	Down	995.8815	−2.69881	0.413601	−6.52516	6.79 × 10^−11^	9.25 × 10^−08^
NCAPH	Down	1459.514	−3.16292	0.490122	−6.45332	1.09 × 10^−10^	1.32 × 10^−07^
KIF18B	Down	1583.582	−3.07757	0.477409	−6.44639	1.15 × 10^−10^	1.32 × 10^−07^
UHRF1	Down	2198.428	−2.78382	0.432416	−6.43782	1.21 × 10^−10^	1.32 × 10^−07^
CDCA3	Down	933.7762	−3.48616	0.542823	−6.42228	1.34 × 10^−10^	1.41 × 10^−07^

**Table 3:** The position of 5 CRC-related candidate genes in the PPI network and subnetworks (modules). As shown, CDK1, CCNA2 and BUB1B genes with the highest degree (K) and betweenness (BC) are clustered in subnetwork 1, demonstrating their significant influence in the network. In contrast, GADD45G and ATF3 genes are classified in clusters 5 and 8, perhaps indicating their independence to the networks in CRC.

Gene Symbol	Expression Based on RNA-seq	Degree (K)	Betweenness (BC)	Closeness Centrality (CC)	MCODE: Score	No; Cluster
CDK1	Down	106	0.94541487	0.885964912	34.77520814	1
CCNA2	Down	96	0.299119893	0.789115646	34.77520814	1
BUB1B	Down	90	0.296979671	0.724550898	34.77520814	1
GADD45G	Up	5	0	0	5	5
ATF3	Up	6	0	0.411764706	2.7	8

**Table 4:** Status of CRC-related genes—CDK1, CCNA2, BUB1B, GADD45G, and ATF3—in gene-pathway annotated networks. The table shows that CDK1 is prominently involved in different pathways. The gene pathways are significantly enriched in cell cycle-related networks. Padj: adjusted *p*-value.

Pathway	Gene Symbol	Padj
**Cell cycle**	TOP2A; ERCC6L; MCM7; PRIM1; HJURP; **BUB1B**; MCM10; TTK; PKMYT1; TYMS; AURKB; LMNB1; CCNB1; POLD1; E2F1; E2F2; CLSPN; BUB1; CENPURRM2; GADD45B; GADD45A; UBE2C; TUBB; PLK1; KIF23; ZWINT; **GADD45G**; DHFR, **CCNA2**; POLA1; CENPF; ESPL1; CENPI; POLE2; **CDK1**; MCM3; MCM4; BIRC5; MCM5; KIF2C; KIF20A; MCM6; SPC24; MCM2; SPC25; MAD2L1	1.82 × 10^−34^
**E2F-mediated regulation of DNA replication**	DHFR; POLA1; CCNB1; RRM2; PRIM1; E2F1; **CDK1**; TYMS	1.67 × 10^−08^
**G1/S-specific transcription**	DHFR; POLA1; RRM2; **CDK1**; E2F1; TYMS	1.79 × 10^−07^
**Cell cycle checkpoints**	MCM7; UBE2C; **BUB1B**; MCM10; CCNB1; **CDK1**; MCM3; MCM4; MCM5; CLSPN; MCM6; MCM2; MAD2L1	2.84 × 10^−09^
**G2/M checkpoints**	CCNB1; MCM7; **CDK1**; MCM3; MCM4; MCM10; MCM5; CLSPN; MCM6; MCM2	2.89 × 10^−10^
**FOXM1 transcription factor network**	**CCNA2**; CCNB1; CENPF; PLK1; **CDK1**; BIRC5; FOXM1; AURKB; HSPA1B	4.54 × 10^−09^
**G1 to S cell cycle control**	DHFR; POLA1; RRM2; **CDK1**; E2F1; TYMS	3.83 × 10^−12^

**Table 5:** The top significantly gene ontology (GO) categories (BP: biological process; CC: cellular component; MF; molecular function) based on upregulated genes with the threshold of |log fold change (FC)| ≥ 2 and a Bonferroni *p* < 0.05. Padj: adjusted *p* value.

GO Term	Source	Padj	Gene Symbol
microtubule cytoskeleton organization involved in mitosis	BP	3.94 × 10^−23^	ERCC6L; BUB1B; CDCA8; TTK; CENPA; TACC3; BIRC5; CENM; KIF2C; SPC24; MAD2L1;
mitotic spindle organization	BP	1.75 × 10^−22^	ERCC6L; BUB1B; CDCA8; TTK; CENPA; KIF2C; SPC24; MAD2L1; SPC25
mitotic sister chromatid segregation	BP	1.37 × 10^−21^	SPAG5; CDCA5; P LK1; NCAPG2; CDCA8; NCAPG; PSRC1; ESPL1; KIFC1; PRC1; CDK1; KIF2C
DNA metabolic process	BP	8.96 × 10^−20^	TOP2A; BLM; FEN1; RNASEH2A; MCM7; UHRF1; HMGB2; MCM10; TYMS; CDK1; MCM4; MCM5; MCM6; MCM2
positive regulation of cell cycle process	BP	9.66 × 10^−10^	UBE2C; TUBB; CCNF; PLK1; CDC7; CDC25C; PKMYT1; FOXM1; AURKA; CCNA2; CCNB2; CCNB1; CDK1; E2F1; TACC3; NEK2; CDKN3
spindle	CC	2.01 × 10^−18^	SPAG5; CKAP2L; PLK1; BUB1B; CDC7; KIF23; TTK; KIF22; SKA3; AURKB; AURKA; CDC20; CENPF; CDK1; TACC3; BIRC5; KIF2C; KIF20A
nucleus	CC	2.20 × 10^−13^	TOP2A; ARHGAP11A; FEN1; MCM7; DSCC1; CCNF; NCAPG2; HMGB2; MCM10; CCNA2; ASPM; MCM5; KIF20A; MCM6; PRR11; MCM2; BLM; RAD51; PRC1; UBE2T; CDK1; TRIP13; MAD2L1
intracellular non-membrane-bounded organelle	CC	4.72 × 10^−10^	TOP2A; FEN1; MCM7; CDCA5; HJURP; HMGB2; BUB1B; CDCA8; MCM10; TTK; MKI67; PKMYT1; AURKB; PLK1; VRK1; KIF23; ESCO2; PIMREG; CIT; CENPF; PSRC1; PRC1; UBE2T; KIF2C; KIF20A; TRIP13; SPC24; MCM2
cyclin-dependent protein kinase holoenzyme complex	CC	4.89 × 10^−05^	CCNA2; CCNB2; CCNB1; CCNF; CDK1
serine/threonine protein kinase complex	CC	1.32 × 10^−04^	CCNA2; CCNB2; CCNB1; CCNF; CDK1
histone kinase activity	MF	7.17 × 10^−04^	CDK1; AURKB; AURKA
protein serine/threonine kinase activity	MF	0.001470884	PLK1; CDK1; PBK; NEK2; VRK1; CDC7; TTK; PASK; PKMYT1; AURKB; CIT; AURKA
kinase binding	MF	0.004957845	CAV1; PLK1; VRK1; CDC25C; FOXM1; AURKB; CIT; AURKA; ARHGAP33; CCNA2
cyclin-dependent protein serine/threonine kinase regulator activity	MF	0.00820989	CCNA2; CCNB2; CCNB1; CCNF

## Data Availability

Data are contained within the article.

## Supplementary Materials

The following supporting information can be downloaded at: https://www.mdpi.com/article/10.3390/molecules27113470/s1, supplementary materials: Transcriptome Profiling of HCT-116 Colorectal Cancer Cells with RNA Sequencing Reveals Novel Targets for Polyphenol Nano Curcumin. The standard Bioconductor RNA-seq workflow (DESeq2), was employed to detect differentially expressed genes (DEGs) in Gemini-Cur treated cell compared to controls. Regarding the parameters *p* values and Log2 fold change, 3892 DEGs were selected as listed.

## Sample Availability

Samples of the compounds are available from the authors.
