# Supplementary material for: Transcriptome Profiling of HCT-116 Colorectal Cancer Cells with RNA Sequencing Reveals Novel Targets for Polyphenol Nano Curcumin
Source: Molecules. 2022 May 27;27(11):3470. doi: 10.3390/molecules27113470 (PMC9182402; doi:10.3390/molecules27113470)
Supplement: Supplementary file 1 [file molecules-27-03470-s001.zip › molecules-1730393-supplementary.pdf]

ENSEMBL Access	baseMean	log2FoldChange	lfcSE	stat	pvalue
ENSG00000100292	14082.62694	4.581562629	0.393634484	11.63912923	2.61E-31
ENSG00000175197	7029.256613	4.083832348	0.394678686	10.34723307	4.31E-25
ENSG00000162772	5160.583093	2.807268493	0.279169857	10.05577222	8.66E-24
ENSG00000128965	2994.215108	3.896156731	0.400671766	9.724061083	2.38E-22
ENSG00000051108	12540.2839	2.267609018	0.254121675	8.923319972	4.53E-19
ENSG00000134548	260.3100572	4.781735304	0.578881871	8.260295488	1.45E-16
ENSG00000128590	2736.218941	2.665123436	0.342986352	7.770348352	7.83E-15
ENSG00000140961	13215.94753	4.534403283	0.586017479	7.737658766	1.01E-14
ENSG00000248323	608.1074734	3.025170425	0.396356099	7.632455838	2.30E-14
ENSG00000160949	2059.715955	-2.895968786	0.395525451	-7.321826647	2.45E-13
ENSG00000186529	547.2113245	2.671815929	0.369557519	7.229770172	4.84E-13
ENSG00000125775	368.0621282	3.854215981	0.535212402	7.201283023	5.96E-13
ENSG00000130766	6910.754023	3.544609276	0.494533657	7.167579451	7.63E-13
ENSG00000250033	277.5812267	3.188924548	0.450900556	7.072345564	1.52E-12
ENSG00000140941	20010.47035	2.997711012	0.426804353	7.023618645	2.16E-12
ENSG00000141582	5588.017669	1.893434606	0.269914217	7.014949523	2.30E-12
ENSG00000171658	109.3127367	3.696930044	0.531123305	6.960587137	3.39E-12
ENSG00000167995	120.1244222	3.395188634	0.495143157	6.856983861	7.03E-12
ENSG00000104738	5519.294187	-2.81519816	0.421580667	-6.677721205	2.43E-11
ENSG00000256663	221.6533313	-3.063761725	0.459516819	-6.667354921	2.60E-11
ENSG00000178381	5371.921397	4.79341078	0.722529271	6.634209811	3.26E-11
ENSG00000158079	1461.089869	1.792619227	0.270471248	6.627762623	3.41E-11
ENSG00000187741	1932.180365	-2.674767211	0.406894311	-6.573616627	4.91E-11
ENSG00000120889	20952.87042	2.09069627	0.318216488	6.570043809	5.03E-11
ENSG00000120738	32667.04831	2.654225119	0.4053165	6.548524716	5.81E-11
ENSG00000197299	995.8815356	-2.698813809	0.413601216	-6.525159274	6.79E-11
ENSG00000007968	629.9051976	-2.623783095	0.40238286	-6.520613465	7.00E-11
ENSG00000113905	76.72572618	4.014459148	0.616768465	6.508859277	7.57E-11
ENSG00000121152	1459.513997	-3.162915213	0.490122466	-6.453316129	1.09E-10
ENSG00000208028	95.04051392	2.969919327	0.460380893	6.451004748	1.11E-10
ENSG00000186185	1583.582198	-3.077565981	0.477409254	-6.446389461	1.15E-10
ENSG00000034063	2198.427871	-2.78381507	0.432415614	-6.437822736	1.21E-10
ENSG00000271303	1495.637262	3.36753052	0.523180382	6.4366529	1.22E-10
ENSG00000111665	933.7761936	-3.486164471	0.542823181	-6.422283704	1.34E-10
ENSG00000087074	16161.06681	3.528430665	0.552995592	6.38057647	1.76E-10
ENSG00000075218	1050.132979	-3.031955592	0.475694621	-6.373743701	1.84E-10
ENSG00000122224	56.56979432	6.49783452	1.021624724	6.3602949	2.01E-10
ENSG00000117724	5816.325565	-2.57046537	0.404495893	-6.354737869	2.09E-10
ENSG00000133639	4392.850736	1.887927906	0.297113941	6.35422189	2.09E-10
ENSG00000152253	583.2480864	-3.433321527	0.541140507	-6.344602704	2.23E-10
ENSG00000161888	574.8987481	-3.604977246	0.568358331	-6.342789482	2.26E-10
ENSG00000161011	36805.84458	2.700353252	0.42576085	6.342417932	2.26E-10
ENSG00000059728	2038.134042	2.83595079	0.45241758	6.268436326	3.65E-10
ENSG00000073111	3444.567429	-2.429574191	0.38917836	-6.242829611	4.30E-10
ENSG00000006652	4344.375693	1.882100399	0.303619662	6.198875214	5.69E-10
ENSG00000166401	1383.403902	2.794044526	0.451019178	6.194957255	5.83E-10

ENSG00000151012	16316.80316	2.708772754	0.437856638	6.18643757	6.15E-10
ENSG00000234719	148.4642286	3.396779818	0.550880767	6.166088966	7.00E-10
ENSG00000122966	1652.700487	-2.721355246	0.443458809	-6.136658439	8.43E-10
ENSG00000076003	3358.48161	-2.064684115	0.336835662	-6.129648211	8.81E-10
ENSG00000228716	1655.517312	-1.875551738	0.307456544	-6.100217336	1.06E-09
ENSG00000170312	3674.238639	-3.158050669	0.523328225	-6.034550625	1.59E-09
ENSG00000148773	11781.90533	-2.52630164	0.418728778	-6.033264899	1.61E-09
ENSG00000255414	101.5702569	2.393797768	0.397708525	6.01897525	1.76E-09
ENSG00000131747	4670.768224	-2.718685071	0.451766339	-6.017900924	1.77E-09
ENSG00000129173	478.7296937	-2.977540435	0.494868095	-6.016836536	1.78E-09
ENSG00000117632	7721.205273	-2.097790458	0.348936176	-6.01196036	1.83E-09
ENSG00000165244	758.795139	-2.575174294	0.429399944	-5.997146317	2.01E-09
ENSG00000168496	2374.048633	-2.376954513	0.396854262	-5.989489703	2.11E-09
ENSG00000166508	7725.92833	-2.535236901	0.424343873	-5.974486872	2.31E-09
ENSG00000130222	199.1380346	4.639295764	0.778303467	5.960780028	2.51E-09
ENSG00000169435	300.2870992	1.885361164	0.317162725	5.944460107	2.77E-09
ENSG00000171320	830.0054182	-2.609868753	0.43906712	-5.94412251	2.78E-09
ENSG00000163898	2159.598059	2.695270736	0.455596444	5.915916975	3.30E-09
ENSG00000176890	2110.839807	-2.881027045	0.487642356	-5.908073841	3.46E-09
ENSG00000128165	1212.302844	2.118218472	0.35856912	5.907420219	3.48E-09
ENSG00000198576	2445.426152	2.222478129	0.376994704	5.895250259	3.74E-09
ENSG00000159556	350.9418666	1.693903233	0.288026697	5.881063295	4.08E-09
ENSG00000136982	1215.730033	-2.476521913	0.421393903	-5.876976141	4.18E-09
ENSG00000204389	876.055314	4.338295144	0.738432604	5.875004869	4.23E-09
ENSG00000126787	2214.174012	-2.963498893	0.504727616	-5.871481571	4.32E-09
ENSG00000065328	1151.177079	-2.543319634	0.433416476	-5.868073263	4.41E-09
ENSG00000004777	482.4956657	-3.156398735	0.538285244	-5.863803196	4.52E-09
ENSG00000101412	856.5758189	-2.339765421	0.400353977	-5.844241729	5.09E-09
ENSG00000100479	511.8331784	-2.808407526	0.481510367	-5.83249649	5.46E-09
ENSG00000156970	2591.459598	-2.989861925	0.513691919	-5.820340584	5.87E-09
ENSG00000099860	3153.377445	2.274074168	0.390826106	5.818634259	5.93E-09
ENSG00000131711	4646.105963	1.99930175	0.343720334	5.816652536	6.00E-09
ENSG00000115687	1034.423716	-2.235029932	0.384283236	-5.81610053	6.02E-09
ENSG00000101255	12977.05425	2.981200572	0.513322083	5.807660868	6.34E-09
ENSG00000178999	1556.032081	-3.191956736	0.550312905	-5.800257824	6.62E-09
ENSG00000135476	1695.194249	-2.627809857	0.454101629	-5.786832038	7.17E-09
ENSG00000204388	41288.99535	4.111985602	0.712642591	5.770053111	7.92E-09
ENSG00000116717	6812.727647	2.184235287	0.37890949	5.764530437	8.19E-09
ENSG00000089685	1551.621555	-2.47217417	0.429325119	-5.758279826	8.50E-09
ENSG00000127564	1122.488667	-2.948731448	0.512884343	-5.749310709	8.96E-09
ENSG00000267056	1675.527347	2.97867973	0.518745437	5.742083725	9.35E-09
ENSG00000197019	3370.042898	2.377326059	0.41474908	5.731962222	9.93E-09
ENSG00000165480	1058.804428	-2.797678744	0.488648297	-5.725342256	1.03E-08
ENSG00000255150	413.4767944	4.212587158	0.741345279	5.682355142	1.33E-08
ENSG00000144655	2734.31781	2.44639291	0.432012058	5.66278849	1.49E-08
ENSG00000168078	2597.463003	-2.772961384	0.490533115	-5.652954505	1.58E-08
ENSG00000136155	50.95206844	8.181190669	1.448327798	5.648714799	1.62E-08

ENSG00000113070	2697.032896	2.743514539	0.48590685	5.646173826	1.64E-08
ENSG00000171848	8292.143365	-2.788561779	0.49416172	-5.643014555	1.67E-08
ENSG00000196182	4019.311832	2.32933432	0.413152772	5.637949146	1.72E-08
ENSG00000186871	646.8201045	-2.722485027	0.484972442	-5.613690158	1.98E-08
ENSG00000119969	1877.969913	-2.067036266	0.369021954	-5.601391036	2.13E-08
ENSG00000198074	283.2857448	3.844171284	0.687881638	5.588419678	2.29E-08
ENSG00000079616	2821.308854	-2.496789794	0.4473297	-5.581542644	2.38E-08
ENSG00000102384	432.6772902	-2.9276817	0.524837694	-5.578261115	2.43E-08
ENSG00000101782	6249.130454	2.304326592	0.415380992	5.547501302	2.90E-08
ENSG00000137310	583.6481559	-2.448207021	0.441430204	-5.546079532	2.92E-08
ENSG00000144354	1419.149094	-2.892321214	0.521650713	-5.544555273	2.95E-08
ENSG00000109084	1266.085596	-1.850259283	0.334067651	-5.538576623	3.05E-08
ENSG00000113368	3563.925829	-2.941806618	0.532072223	-5.528961094	3.22E-08
ENSG00000151503	2864.973361	-2.117168417	0.383535637	-5.520134801	3.39E-08
ENSG00000023909	6386.108227	2.101722604	0.380837702	5.518683133	3.42E-08
ENSG00000108448	1240.796602	1.539868181	0.279383096	5.511672692	3.55E-08
ENSG00000090020	3877.245852	2.230829388	0.40495149	5.508880557	3.61E-08
ENSG00000198554	1376.001458	-2.51996643	0.457781232	-5.504739497	3.70E-08
ENSG00000071539	1264.032445	-2.443573119	0.443974309	-5.503861523	3.72E-08
ENSG00000107984	10977.71447	-3.090596433	0.561661772	-5.502593534	3.74E-08
ENSG00000159885	324.8885459	3.080612363	0.559934835	5.50173372	3.76E-08
ENSG00000092853	865.6041028	-2.371453184	0.431936818	-5.490277939	4.01E-08
ENSG00000228727	53.03226258	-4.702900958	0.857416548	-5.4849664	4.14E-08
ENSG00000129195	820.0110071	-2.799211943	0.510392751	-5.484427311	4.15E-08
ENSG00000138376	617.9682373	-1.975227316	0.360187386	-5.483888092	4.16E-08
ENSG00000171303	211.1711997	4.268692587	0.780957401	5.465973664	4.60E-08
ENSG00000177606	4766.09439	1.636050301	0.299715736	5.458673346	4.80E-08
ENSG00000120217	870.5490995	2.579715753	0.472609762	5.458447876	4.80E-08
ENSG00000112118	5471.895925	-1.949081225	0.357363705	-5.45405478	4.92E-08
ENSG00000066279	2211.688205	-2.33067172	0.428034155	-5.44506015	5.18E-08
ENSG00000261592	63.30003068	4.255523309	0.782723196	5.436817677	5.42E-08
ENSG00000261270	150.8481608	2.332285679	0.429976598	5.424215383	5.82E-08
ENSG00000010379	103.4144207	4.755497686	0.876834422	5.423484262	5.84E-08
ENSG00000164109	2923.876803	-2.545217325	0.469989702	-5.415474666	6.11E-08
ENSG00000112984	2023.481122	-3.014324773	0.55664121	-5.415202322	6.12E-08
ENSG00000237649	2232.231546	-2.615976433	0.484186461	-5.402828547	6.56E-08
ENSG00000162063	1536.049709	-2.608998353	0.484110347	-5.389263766	7.07E-08
ENSG00000100439	2269.900909	2.825269804	0.524755717	5.383971463	7.29E-08
ENSG00000198496	361.6170082	1.972785931	0.366451553	5.383483611	7.31E-08
ENSG00000168393	968.0867401	-2.037370999	0.378839363	-5.377928475	7.53E-08
ENSG00000160298	230.2083952	-2.415668186	0.449858071	-5.369845161	7.88E-08
ENSG00000109674	462.5752636	-3.297463499	0.615709071	-5.35555452	8.53E-08
ENSG00000156510	455.0836518	2.617599897	0.490574619	5.335783372	9.51E-08
ENSG00000113369	3572.118208	1.539515895	0.289033396	5.326429112	1.00E-07
ENSG00000166851	3006.888787	-2.898262895	0.545220298	-5.315764851	1.06E-07
ENSG00000214826	444.8382598	-2.272984024	0.428385223	-5.305934714	1.12E-07
ENSG00000164309	854.4787236	4.074371321	0.768107058	5.304431563	1.13E-07

ENSG00000145386	2508.287382	-3.056475969	0.576266639	-5.303926624	1.13E-07
ENSG00000251192	570.1613383	2.005952575	0.378601174	5.298326342	1.17E-07
ENSG00000145536	150.855857	2.362960649	0.446275339	5.29484926	1.19E-07
ENSG00000123485	1736.204189	-2.754235977	0.5209209	-5.28724414	1.24E-07
ENSG00000100749	1237.412438	-2.3098523	0.438113998	-5.272263176	1.35E-07
ENSG00000169679	3632.926378	-2.678263324	0.508052079	-5.271631463	1.35E-07
ENSG00000170222	495.9720182	1.534154446	0.291244853	5.267576158	1.38E-07
ENSG00000137807	2423.40703	-2.288598663	0.435426121	-5.255997637	1.47E-07
ENSG00000178607	2425.270283	1.476688351	0.281466908	5.246401298	1.55E-07
ENSG00000122952	1708.212817	-2.286801648	0.435905093	-5.246099857	1.55E-07
ENSG00000076248	2379.303198	-1.575850974	0.300494248	-5.2441968	1.57E-07
ENSG00000100297	2792.231431	-2.364194942	0.45099391	-5.242188176	1.59E-07
ENSG00000013573	1728.630171	-2.157394424	0.411621421	-5.241210286	1.60E-07
ENSG00000175063	2965.187271	-3.007835223	0.573888789	-5.241146511	1.60E-07
ENSG00000100726	1055.069635	-1.966390133	0.375565362	-5.235813346	1.64E-07
ENSG00000130816	6077.146303	-1.733255596	0.33150429	-5.228456008	1.71E-07
ENSG00000167900	3977.715024	-2.076359423	0.398220535	-5.214094305	1.85E-07
ENSG00000142945	2410.253914	-2.746678226	0.527069937	-5.211221576	1.88E-07
ENSG00000172602	324.3678263	2.967797854	0.570414617	5.202878338	1.96E-07
ENSG00000150961	3145.280334	1.329360403	0.255741344	5.198066066	2.01E-07
ENSG00000087086	184906.0298	2.499342012	0.482518736	5.179782304	2.22E-07
ENSG00000137135	494.9681011	-2.77932445	0.537198184	-5.173741338	2.29E-07
ENSG00000220773	34.19292955	3.335232231	0.644917389	5.171565051	2.32E-07
ENSG00000196230	19732.65972	-2.149715056	0.41616493	-5.165536311	2.40E-07
ENSG00000111206	1639.428196	-2.076599739	0.402279985	-5.162075705	2.44E-07
ENSG00000115008	99.66818377	3.300614221	0.640671295	5.151806003	2.58E-07
ENSG00000239705	25.96226463	7.202345491	1.398116442	5.151463266	2.58E-07
ENSG00000023171	1589.517222	1.92372419	0.373570709	5.149558421	2.61E-07
ENSG00000119333	2210.154554	-2.162067026	0.421176037	-5.133404644	2.85E-07
ENSG00000186594	6051.915669	6.122028265	1.196033413	5.118609729	3.08E-07
ENSG00000086544	1977.905688	2.597756052	0.50793052	5.114392523	3.15E-07
ENSG00000127586	1548.235789	-2.216309845	0.435227384	-5.092303307	3.54E-07
ENSG00000101868	1212.202103	-1.903363964	0.374257736	-5.085703732	3.66E-07
ENSG00000173706	517.3480249	-1.437553325	0.283032316	-5.079113741	3.79E-07
ENSG00000230799	44.00630987	5.482805376	1.08125686	5.070770486	3.96E-07
ENSG00000170801	31.80813158	4.8171811	0.950439917	5.068369935	4.01E-07
ENSG00000139988	47.12765872	2.872256387	0.567337363	5.062695631	4.13E-07
ENSG00000132002	37229.66328	2.64064691	0.521638863	5.062212759	4.14E-07
ENSG00000116830	1442.06513	-2.205888636	0.43578749	-5.061844787	4.15E-07
ENSG00000151725	539.2778577	-1.777781812	0.351285068	-5.060795269	4.18E-07
ENSG00000185022	3901.976913	2.421524357	0.478558208	5.060041425	4.19E-07
ENSG00000160957	2662.387677	-1.842434394	0.364275279	-5.057807925	4.24E-07
ENSG00000062822	1359.339795	-2.090095169	0.413451663	-5.055234645	4.30E-07
ENSG00000198056	817.9450081	-1.956029809	0.387078476	-5.053315886	4.34E-07
ENSG00000155304	4547.25581	1.769707127	0.350546788	5.048419176	4.45E-07
ENSG00000112742	1735.878779	-2.449107841	0.485249049	-5.047115178	4.49E-07
ENSG00000035499	830.4904359	-2.267223129	0.449289955	-5.046235958	4.51E-07

ENSG00000134057	5209.327474	-2.515652928	0.499158314	-5.0397897	4.66E-07
ENSG00000152433	209.791379	2.799451894	0.555927726	5.035639998	4.76E-07
ENSG00000044574	161237.651	1.976165845	0.392859715	5.030207399	4.90E-07
ENSG00000091129	56.88380735	5.402875268	1.074404092	5.028718068	4.94E-07
ENSG00000137745	39.49392446	4.793851109	0.954362818	5.023090819	5.08E-07
ENSG00000105486	1504.960127	-2.540480033	0.506439721	-5.016352253	5.27E-07
ENSG00000177854	125.9107143	-2.564613737	0.511269485	-5.016168209	5.27E-07
ENSG00000158615	12131.37785	1.495019701	0.29804998	5.016003361	5.28E-07
ENSG00000212724	140.4999166	-4.335098219	0.864644086	-5.013737202	5.34E-07
ENSG00000143228	1137.329947	-2.341574853	0.467565964	-5.008009633	5.50E-07
ENSG00000114346	4019.153243	-2.105524154	0.420536238	-5.006760328	5.54E-07
ENSG00000100867	1572.553578	-2.987750263	0.597898053	-4.997089799	5.82E-07
ENSG00000162062	426.2009292	-2.371958539	0.475665223	-4.986613325	6.14E-07
ENSG00000070669	22644.59486	2.348254588	0.47123766	4.983164094	6.26E-07
ENSG00000172403	186.1427576	2.63450374	0.528751037	4.982503214	6.28E-07
ENSG00000162733	335.5985329	2.625470787	0.52741503	4.97799766	6.42E-07
ENSG00000080986	982.2796752	-2.484884181	0.500082117	-4.96895229	6.73E-07
ENSG00000149929	549.5354005	-2.295243769	0.462108818	-4.966890221	6.80E-07
ENSG00000267149	57.71937277	4.003552497	0.807203826	4.959778893	7.06E-07
ENSG00000150991	90565.15787	2.46678605	0.49772349	4.956137494	7.19E-07
ENSG00000174371	929.3481514	-2.330486673	0.470922803	-4.94876582	7.47E-07
ENSG00000167996	36425.40361	1.391876056	0.282093325	4.934097813	8.05E-07
ENSG00000118513	139.8383063	-3.096260076	0.628473893	-4.926632768	8.37E-07
ENSG00000143067	1657.312096	2.231123279	0.452998664	4.92523148	8.43E-07
ENSG00000013810	2749.254024	-2.174910835	0.441650071	-4.924511457	8.46E-07
ENSG00000107864	373.4215133	1.850624664	0.375812937	4.924323994	8.47E-07
ENSG00000197301	112.9212352	-2.970139882	0.603572811	-4.920930548	8.61E-07
ENSG00000121621	1078.68468	-1.774037124	0.36054435	-4.920440779	8.63E-07
ENSG00000102265	9106.87257	1.526223528	0.310319488	4.918232941	8.73E-07
ENSG00000143367	1540.927409	1.752481909	0.356518977	4.91553612	8.85E-07
ENSG00000158402	526.6938771	-2.465426691	0.501562835	-4.915489187	8.86E-07
ENSG00000166278	33.47459829	2.857970446	0.581776884	4.912485398	8.99E-07
ENSG00000115902	744.3396127	1.630126803	0.331956332	4.910666397	9.08E-07
ENSG00000146918	2575.393843	-2.030620386	0.413791541	-4.90735113	9.23E-07
ENSG00000176018	2926.730433	1.284304238	0.261886018	4.904058071	9.39E-07
ENSG00000152137	1921.641609	2.963603877	0.605425864	4.895073127	9.83E-07
ENSG00000101057	2277.540997	-2.291670286	0.46893873	-4.886929014	1.02E-06
ENSG00000011426	5557.786798	-2.44937259	0.502084179	-4.878410221	1.07E-06
ENSG00000100162	221.8934773	-2.772516074	0.568874083	-4.87369025	1.10E-06
ENSG00000038427	3773.167937	-2.671106483	0.548771244	-4.867431575	1.13E-06
ENSG00000171223	7823.173544	1.253887497	0.257915176	4.861627442	1.16E-06
ENSG00000153714	401.8332583	2.165478578	0.445540781	4.860337525	1.17E-06
ENSG00000173212	53.10392293	4.846042063	0.99814611	4.85504278	1.20E-06
ENSG00000111602	2484.016683	-2.071616079	0.42757919	-4.844988083	1.27E-06
ENSG00000139112	6603.483738	2.409026217	0.497253232	4.84466678	1.27E-06
ENSG00000165813	4130.191587	1.932599298	0.399021315	4.843348521	1.28E-06
ENSG00000119801	3407.800151	2.027801361	0.419284935	4.836332505	1.32E-06

ENSG00000229808	38.74275678	4.334700385	0.897325629	4.830688264	1.36E-06
ENSG00000163808	635.7212543	-2.251489365	0.46653711	-4.82595986	1.39E-06
ENSG00000148841	2230.995879	1.647243754	0.341465446	4.824042301	1.41E-06
ENSG00000131153	1056.269512	-2.456290686	0.509316988	-4.822715021	1.42E-06
ENSG00000140451	447.6175778	-3.483100715	0.722477561	-4.821050376	1.43E-06
ENSG00000183856	1421.826762	-2.440820073	0.506597268	-4.818067976	1.45E-06
ENSG00000166750	4139.315651	1.578037182	0.327590637	4.817100986	1.46E-06
ENSG00000166803	651.4765172	-2.054794846	0.426947946	-4.812752615	1.49E-06
ENSG00000161996	620.7306776	-2.36850122	0.492446774	-4.809659324	1.51E-06
ENSG00000138078	4055.332828	1.614722584	0.335823527	4.808247352	1.52E-06
ENSG00000196550	371.3358135	-2.770618728	0.577390368	-4.798519135	1.60E-06
ENSG00000179918	4464.970532	1.292145165	0.269393108	4.796504173	1.61E-06
ENSG00000188610	221.4453024	-2.852723312	0.595148115	-4.793299752	1.64E-06
ENSG00000047346	1191.309436	2.975407563	0.620838127	4.792565783	1.65E-06
ENSG00000160588	1286.57368	2.232479476	0.466491404	4.785681918	1.70E-06
ENSG00000049541	1416.776718	-1.937314111	0.404878613	-4.784925775	1.71E-06
ENSG00000138180	2649.426356	-2.168190564	0.453204021	-4.784137973	1.72E-06
ENSG00000171471	90.71578073	2.487534026	0.520057203	4.783193103	1.73E-06
ENSG00000153930	430.9717703	3.108722164	0.649950439	4.783014177	1.73E-06
ENSG00000146670	1965.462216	-2.22167994	0.464520101	-4.782742307	1.73E-06
ENSG00000087586	2134.695556	-2.23847286	0.46812937	-4.781739843	1.74E-06
ENSG00000050628	16.11790899	7.482825017	1.565585417	4.779569952	1.76E-06
ENSG00000104889	1159.829267	-2.297659734	0.481159152	-4.775259337	1.79E-06
ENSG00000151790	18.47322722	6.704482429	1.404743989	4.772743278	1.82E-06
ENSG00000165304	2200.608683	-1.982419727	0.415529663	-4.770826021	1.83E-06
ENSG00000087494	44.33784753	2.77961734	0.583150485	4.766552396	1.87E-06
ENSG00000166086	33.40661119	-2.839125018	0.596395372	-4.760474595	1.93E-06
ENSG00000105974	10801.42482	-2.146536715	0.45115748	-4.757843565	1.96E-06
ENSG00000140525	2462.919269	-1.957342332	0.41202579	-4.750533535	2.03E-06
ENSG00000135842	2491.151896	2.379973296	0.50108076	4.749680064	2.04E-06
ENSG00000132950	1235.535595	1.662661366	0.350405065	4.744969562	2.09E-06
ENSG00000068489	1970.107851	-2.285167156	0.482165136	-4.739386956	2.14E-06
ENSG00000145604	1109.493489	-1.421569918	0.300192171	-4.735532954	2.18E-06
ENSG00000215784	277.6521944	-2.868895355	0.607041889	-4.726025346	2.29E-06
ENSG00000178385	398.9749122	2.075217318	0.439310874	4.723801395	2.31E-06
ENSG00000135451	1439.58057	-2.326860008	0.492762914	-4.722068043	2.33E-06
ENSG00000177494	262.511127	-3.052748741	0.647822791	-4.712320686	2.45E-06
ENSG00000127325	50.11632318	4.423341714	0.939344675	4.708965549	2.49E-06
ENSG00000189403	13427.66209	-1.974943229	0.419422594	-4.708719216	2.49E-06
ENSG00000099901	4080.639004	-1.983206741	0.42126129	-4.707783008	2.50E-06
ENSG00000163376	854.3389438	3.218729462	0.683742046	4.707520151	2.51E-06
ENSG00000111445	1819.446755	-1.681711744	0.357770544	-4.700531595	2.59E-06
ENSG00000246130	25.96047177	4.318031667	0.919082605	4.698197575	2.62E-06
ENSG00000184588	2125.605306	-1.982125403	0.421939502	-4.697653079	2.63E-06
ENSG00000260442	49.39386577	-3.079833743	0.655764315	-4.696555867	2.65E-06
ENSG00000051180	454.5441607	-2.310574748	0.492353872	-4.692914745	2.69E-06
ENSG00000157456	1863.652134	-2.305359533	0.491428274	-4.691141419	2.72E-06

ENSG00000112312	1056.894779	-1.76223628	0.375822607	-4.689010845	2.75E-06
ENSG00000172901	275.1194379	2.426111507	0.517554662	4.687643037	2.76E-06
ENSG00000130600	77.90195604	-2.860655962	0.610889037	-4.682775081	2.83E-06
ENSG00000109805	3111.15185	-2.270141906	0.484831779	-4.682329019	2.84E-06
ENSG00000184178	304.7131828	-1.741836869	0.372167933	-4.680244357	2.87E-06
ENSG00000171903	7538.445331	2.860734105	0.61217703	4.67305038	2.97E-06
ENSG00000109255	711.443537	-2.509693017	0.537180416	-4.671974151	2.98E-06
ENSG00000165197	103.4312236	2.019953665	0.432687218	4.66839227	3.04E-06
ENSG00000197785	1782.205563	-1.806503104	0.387414718	-4.662969738	3.12E-06
ENSG00000169607	1023.666889	-2.279966533	0.489094381	-4.661608519	3.14E-06
ENSG00000111181	47.00247837	4.78244052	1.028274557	4.650937325	3.30E-06
ENSG00000136535	79.97516948	3.581815976	0.771084708	4.64516536	3.40E-06
ENSG00000077152	1452.747917	-2.3611341	0.508448671	-4.643800315	3.42E-06
ENSG00000147883	647.2335721	1.40067539	0.301693621	4.642708014	3.44E-06
ENSG00000245954	352.8319669	3.638812232	0.784847968	4.636327517	3.55E-06
ENSG00000111788	439.7457153	-2.169312323	0.467945367	-4.63582391	3.56E-06
ENSG00000080031	1432.16721	1.511983419	0.326203123	4.635097934	3.57E-06
ENSG00000259781	422.8655263	-2.160496861	0.466175645	-4.634512517	3.58E-06
ENSG00000151694	4044.42591	1.563455779	0.337538119	4.631938411	3.62E-06
ENSG00000164463	913.2558063	1.65975146	0.358834593	4.625394235	3.74E-06
ENSG00000121931	1938.025761	1.824662809	0.394952712	4.619952599	3.84E-06
ENSG00000244693	87.08441843	2.617830206	0.567023879	4.61679006	3.90E-06
ENSG00000095794	1403.672453	2.434251748	0.527378238	4.61576071	3.92E-06
ENSG00000115163	557.1561669	-2.706333419	0.586877708	-4.611409466	4.00E-06
ENSG00000250899	302.1873853	-1.717226695	0.37289951	-4.605065567	4.12E-06
ENSG00000080839	589.7719839	-1.561954361	0.339577727	-4.599696145	4.23E-06
ENSG00000175105	1182.153312	1.670751347	0.363587506	4.595183601	4.32E-06
ENSG00000248049	487.0421013	1.77046883	0.385838436	4.588627426	4.46E-06
ENSG00000168077	1047.43054	-1.603683796	0.3497168	-4.585664159	4.53E-06
ENSG00000271522	31.47899217	4.143568351	0.903937007	4.583912729	4.56E-06
ENSG00000198901	3439.109024	-2.178673688	0.475453045	-4.58231094	4.60E-06
ENSG00000170345	6659.561529	1.798642069	0.392765413	4.5794309	4.66E-06
ENSG00000160446	611.9806135	-1.773959542	0.387420924	-4.578894506	4.67E-06
ENSG00000177225	1940.088204	-1.437496202	0.313993453	-4.578108843	4.69E-06
ENSG00000010292	7417.125098	-1.869798285	0.408562708	-4.576527051	4.73E-06
ENSG00000163918	1580.459959	-1.962732984	0.429643975	-4.568277691	4.92E-06
ENSG00000164972	59.90929787	3.849882233	0.842820564	4.567855128	4.93E-06
ENSG00000175785	74.32534594	-2.323536085	0.509116764	-4.56385696	5.02E-06
ENSG00000095002	1951.840412	-1.993863255	0.436907552	-4.56358158	5.03E-06
ENSG00000091592	1317.576427	1.692442835	0.371242021	4.558866561	5.14E-06
ENSG00000176619	9752.044507	-1.647880088	0.361647223	-4.556595442	5.20E-06
ENSG00000226979	33.06259634	4.620108319	1.01401506	4.556252169	5.21E-06
ENSG00000257052	20.54433212	5.43160651	1.192299807	4.555571071	5.22E-06
ENSG00000205274	11.02477285	6.93408165	1.522308569	4.554977743	5.24E-06
ENSG00000105011	1882.289246	-2.131541943	0.468094936	-4.553653076	5.27E-06
ENSG00000178921	1193.493676	-2.227386521	0.489346322	-4.551758995	5.32E-06
ENSG00000124171	2160.448924	1.271050271	0.279346994	4.550076777	5.36E-06

ENSG00000076382	2012.017654	-2.117908015	0.466121443	-4.543682872	5.53E-06
ENSG00000166965	813.0182517	-2.103152527	0.463558302	-4.536975215	5.71E-06
ENSG00000197744	3003.985261	-1.952427052	0.430340465	-4.536935773	5.71E-06
ENSG00000143507	306.7876093	1.531715561	0.337629038	4.536681946	5.71E-06
ENSG00000117650	1570.461081	-2.707967581	0.597164088	-4.534712715	5.77E-06
ENSG00000118785	671.5224752	2.573401042	0.568035464	4.530352775	5.89E-06
ENSG00000267259	28.97286427	3.205228284	0.708965564	4.520992904	6.16E-06
ENSG00000161800	3339.74474	-1.867323906	0.413085981	-4.520424304	6.17E-06
ENSG00000072571	1885.522707	-2.189114455	0.485123769	-4.512486489	6.41E-06
ENSG00000140830	1534.676918	2.082314994	0.461636346	4.510725839	6.46E-06
ENSG00000149503	2174.030292	-1.731609287	0.383890017	-4.510691113	6.46E-06
ENSG00000132646	5827.300899	-1.761526583	0.390613285	-4.509643296	6.49E-06
ENSG00000029993	3155.619987	-1.857489255	0.412105267	-4.507317438	6.57E-06
ENSG00000235027	106.0996482	4.30949159	0.956200427	4.506891513	6.58E-06
ENSG00000260565	496.6520584	2.015567546	0.447287045	4.506205958	6.60E-06
ENSG00000261114	81.68005049	2.177415735	0.484082394	4.49802712	6.86E-06
ENSG00000164104	6448.741202	-2.229987446	0.495864107	-4.497174553	6.89E-06
ENSG00000120885	38081.07916	3.040575711	0.67626943	4.496101077	6.92E-06
ENSG00000179409	1754.672719	-1.457030962	0.325395179	-4.477727561	7.54E-06
ENSG00000136870	1404.604603	2.101968182	0.469780367	4.47436362	7.66E-06
ENSG00000205336	2751.796964	1.141191413	0.255077076	4.47390816	7.68E-06
ENSG00000120802	5976.104041	-1.768462789	0.396131001	-4.464338274	8.03E-06
ENSG00000120686	4121.540346	1.324402054	0.296696918	4.463821412	8.05E-06
ENSG00000132780	5425.424441	-1.43348855	0.321439917	-4.459584746	8.21E-06
ENSG00000101447	1598.432081	-1.775445163	0.39991791	-4.439524008	9.02E-06
ENSG00000256128	45.37049881	2.439930571	0.549600108	4.439465231	9.02E-06
ENSG00000230825	29.96175592	4.024825299	0.906936658	4.437824037	9.09E-06
ENSG00000196632	36.31170985	3.754096852	0.84613408	4.436763558	9.13E-06
ENSG00000132823	3130.19044	2.093652792	0.472212947	4.43370476	9.26E-06
ENSG00000134222	738.1599262	-2.371695322	0.535063442	-4.432549743	9.31E-06
ENSG00000265415	163.930454	-2.264146132	0.51083911	-4.43220984	9.33E-06
ENSG00000138271	61.37151	2.094098515	0.472748403	4.429625779	9.44E-06
ENSG00000057657	193.6288804	1.861076459	0.420251024	4.428487625	9.49E-06
ENSG00000184207	832.9357294	-1.799486899	0.40637756	-4.42811581	9.51E-06
ENSG00000221963	166.6252015	2.020295693	0.45652881	4.42534107	9.63E-06
ENSG00000138378	323.6188904	1.59508304	0.360474344	4.4249558	9.65E-06
ENSG00000113742	2859.905561	2.004653822	0.45304913	4.424804488	9.65E-06
ENSG00000102359	234.7527584	2.258140292	0.510992547	4.419125689	9.91E-06
ENSG00000084764	1533.379535	1.623692303	0.367430916	4.419041053	9.91E-06
ENSG00000102580	6957.122067	1.216218829	0.275573689	4.413406935	1.02E-05
ENSG00000179562	2967.800444	1.631235282	0.369850024	4.410531774	1.03E-05
ENSG00000167670	1542.59812	-1.896260371	0.430125818	-4.408617874	1.04E-05
ENSG00000006047	99.04640596	-2.859625458	0.649345132	-4.403860627	1.06E-05
ENSG00000085999	643.7859466	-2.093249994	0.476275367	-4.39504148	1.11E-05
ENSG00000117399	6377.456511	-2.417514041	0.5502441	-4.393530149	1.12E-05
ENSG00000100219	5070.665917	1.210578919	0.275922359	4.387389711	1.15E-05
ENSG00000136492	846.8772292	-1.807712068	0.412305254	-4.384402214	1.16E-05

ENSG00000176974	1484.782742	-1.711606115	0.390397566	-4.384264305	1.16E-05
ENSG00000196110	547.4291311	1.717959615	0.392573823	4.376144093	1.21E-05
ENSG00000227398	359.2519456	2.440349678	0.559065544	4.36505112	1.27E-05
ENSG00000102879	113.5964543	-2.057138642	0.471330362	-4.364536652	1.27E-05
ENSG00000115884	2024.283232	-2.46721305	0.565496504	-4.362914769	1.28E-05
ENSG00000109220	627.7310529	1.888937374	0.433024586	4.362194278	1.29E-05
ENSG00000078900	604.8230818	-1.415866178	0.32461899	-4.361624616	1.29E-05
ENSG00000253729	15917.6592	-1.438901473	0.329926673	-4.361276581	1.29E-05
ENSG00000135828	978.8444989	2.167334858	0.497765261	4.354130404	1.34E-05
ENSG00000138160	2042.975283	-1.940636907	0.446155165	-4.349690558	1.36E-05
ENSG00000204054	2888.856851	1.759566593	0.404730302	4.347503968	1.38E-05
ENSG00000105676	843.6796847	-1.524976736	0.351026435	-4.344335879	1.40E-05
ENSG00000071537	7724.236623	1.190377425	0.274055061	4.343570299	1.40E-05
ENSG00000132837	131.5742787	1.649548975	0.379880654	4.342282134	1.41E-05
ENSG00000109685	6312.711023	-1.590227194	0.366333662	-4.34092566	1.42E-05
ENSG00000167470	8552.211002	1.385642328	0.319353114	4.338903448	1.43E-05
ENSG00000189057	316.1497367	-1.944126974	0.44810535	-4.338548906	1.43E-05
ENSG00000236039	10.66179857	6.885971466	1.587782809	4.336847222	1.45E-05
ENSG00000087589	53.1270845	2.903408947	0.669624491	4.335876283	1.45E-05
ENSG00000156504	3733.348331	-1.473086217	0.340554084	-4.325557341	1.52E-05
ENSG00000232450	94.99934198	3.160511074	0.731630637	4.319817833	1.56E-05
ENSG00000171503	1474.350882	1.543454997	0.357319759	4.31953442	1.56E-05
ENSG00000128973	642.9905167	-1.36790039	0.316782435	-4.318106809	1.57E-05
ENSG00000148926	5269.428115	1.685022105	0.390481496	4.315241878	1.59E-05
ENSG00000171877	2818.80624	-1.383435435	0.320800755	-4.312444446	1.61E-05
ENSG00000065057	385.991149	-1.918881026	0.445105805	-4.311067174	1.62E-05
ENSG00000135924	4136.819212	2.116954262	0.49142794	4.307761303	1.65E-05
ENSG00000189410	495.3794791	-1.491196214	0.346532511	-4.303192826	1.68E-05
ENSG00000249328	35.0008738	3.375443725	0.784594022	4.302153257	1.69E-05
ENSG00000097046	1119.093934	-2.077419529	0.483293158	-4.298466668	1.72E-05
ENSG00000120709	5991.751098	2.212348321	0.514979536	4.295992687	1.74E-05
ENSG00000117877	337.5870855	-1.920830415	0.447262692	-4.294635904	1.75E-05
ENSG00000131018	1289.923377	2.105691831	0.490812667	4.290214927	1.79E-05
ENSG00000251003	2010.485186	2.115140206	0.493277879	4.287928355	1.80E-05
ENSG00000101003	1147.375269	-2.039900958	0.476964211	-4.276842809	1.90E-05
ENSG00000184661	1228.42166	-1.831225364	0.428203256	-4.276533019	1.90E-05
ENSG00000196189	49.5262011	3.279630964	0.767324871	4.274110078	1.92E-05
ENSG00000113811	3659.59292	2.279728587	0.533699086	4.271561725	1.94E-05
ENSG00000170727	553.2795265	-2.189093926	0.512652065	-4.270135778	1.95E-05
ENSG00000117984	5007.016748	2.131660348	0.499420466	4.268267904	1.97E-05
ENSG00000132967	295.4259243	-2.010195693	0.47112898	-4.266762983	1.98E-05
ENSG00000197275	220.3890607	-1.953990797	0.458216914	-4.264335816	2.00E-05
ENSG00000186193	1525.02664	-1.873724194	0.43955891	-4.262737376	2.02E-05
ENSG00000023287	5932.273944	1.568286854	0.368132257	4.260118003	2.04E-05
ENSG00000116649	1768.973349	-1.819732801	0.427815535	-4.253545402	2.10E-05
ENSG00000198826	3162.146573	-2.045314876	0.480891778	-4.253170816	2.11E-05
ENSG00000267374	94.26576417	-2.156288237	0.50779842	-4.246346885	2.17E-05

ENSG00000234776	16.97719458	4.734115154	1.114947874	4.246041691	2.18E-05
ENSG00000144747	2501.876824	1.23303206	0.290414048	4.245772783	2.18E-05
ENSG00000161980	376.7857727	-1.562591665	0.368107591	-4.244931932	2.19E-05
ENSG00000091513	53.86190749	1.944823662	0.458419673	4.242452444	2.21E-05
ENSG00000164362	145.4327568	-3.469089231	0.817723291	-4.242375469	2.21E-05
ENSG00000232811	91.82952344	2.292385983	0.5403983	4.242030335	2.22E-05
ENSG00000140534	960.0192279	-2.055579019	0.48480685	-4.239995823	2.24E-05
ENSG00000102967	503.8657192	-2.062671177	0.486845989	-4.236804297	2.27E-05
ENSG00000137804	3781.757517	-1.868764749	0.44122932	-4.235359401	2.28E-05
ENSG00000120053	15344.05072	1.955654502	0.462283296	4.230424326	2.33E-05
ENSG00000186281	523.6723518	-2.152859423	0.509027394	-4.229358674	2.34E-05
ENSG00000231131	635.6393413	-2.249180553	0.532162283	-4.226493733	2.37E-05
ENSG00000229512	42.47869743	4.505724527	1.066397401	4.225183333	2.39E-05
ENSG00000176274	143.8218988	2.5662826	0.607397996	4.225042916	2.39E-05
ENSG00000156639	4286.380369	1.724096231	0.408224483	4.223402326	2.41E-05
ENSG00000088970	894.3530347	1.422993407	0.337039647	4.22203566	2.42E-05
ENSG00000138111	883.3094164	-1.831477994	0.434009411	-4.21990387	2.44E-05
ENSG00000165891	953.0866342	-1.986898644	0.470888481	-4.219467502	2.45E-05
ENSG00000117226	124.8832239	2.212906522	0.524460331	4.219397337	2.45E-05
ENSG00000130713	1810.58995	-1.411268747	0.334717809	-4.216294167	2.48E-05
ENSG00000233822	130.0045106	2.143086832	0.508314015	4.216068747	2.49E-05
ENSG00000163535	1396.800462	-1.869092913	0.443524557	-4.214181344	2.51E-05
ENSG00000182831	5543.543842	1.651180975	0.392161234	4.210464554	2.55E-05
ENSG00000173638	775.3643489	-2.180255445	0.517975549	-4.209186034	2.56E-05
ENSG00000104549	2866.183969	-1.376531496	0.327646381	-4.201271786	2.65E-05
ENSG00000101605	135.1199537	1.549131361	0.368736117	4.201192365	2.66E-05
ENSG00000185955	74.97663779	3.225854664	0.768151435	4.199503534	2.68E-05
ENSG00000100350	762.1391536	-1.684706802	0.401383839	-4.197246219	2.70E-05
ENSG00000225434	54.02556536	2.418699931	0.576744672	4.193710057	2.74E-05
ENSG00000173575	9410.817775	1.302008905	0.310687741	4.190731508	2.78E-05
ENSG00000198010	14.55916576	6.356231762	1.517230613	4.189364299	2.80E-05
ENSG00000183696	5256.609514	2.531028939	0.604264402	4.188611694	2.81E-05
ENSG00000121211	515.3849305	-2.229921699	0.5325087	-4.187577962	2.82E-05
ENSG00000166987	2291.735356	1.150892857	0.275067571	4.184036864	2.86E-05
ENSG00000179674	85.02490579	2.295421493	0.54864036	4.183836376	2.87E-05
ENSG00000102900	2924.562207	-1.459575639	0.349243664	-4.179247294	2.92E-05
ENSG00000154146	351.020917	-1.291413432	0.309006265	-4.179246767	2.92E-05
ENSG00000024526	2276.976665	-1.683434614	0.402822793	-4.179094742	2.93E-05
ENSG00000005238	1890.481792	1.878183935	0.449536627	4.17804428	2.94E-05
ENSG00000159917	449.976147	2.60446151	0.624128303	4.172958507	3.01E-05
ENSG00000175984	709.543617	1.533466575	0.367499356	4.17270547	3.01E-05
ENSG00000229261	90.42229264	3.391057102	0.813423074	4.16887252	3.06E-05
ENSG00000261824	720.8890992	1.282089912	0.307629471	4.167643329	3.08E-05
ENSG00000151025	248.8190751	1.555296054	0.373189713	4.167574828	3.08E-05
ENSG00000162944	28.08434172	2.458512257	0.590017787	4.166844307	3.09E-05
ENSG00000031081	796.562824	2.270177415	0.544830278	4.166760745	3.09E-05
ENSG00000130052	301.8166385	-2.113490106	0.507398748	-4.165343557	3.11E-05

ENSG00000111664	169.1388903	-2.039790483	0.489890001	-4.163772434	3.13E-05
ENSG00000186832	56.54314899	2.651056616	0.636979416	4.161918816	3.16E-05
ENSG00000170949	1941.52129	1.443528923	0.346867406	4.161615932	3.16E-05
ENSG00000149636	864.4543803	-1.708261092	0.410841767	-4.157953808	3.21E-05
ENSG00000148346	35.08324979	2.961730796	0.712344162	4.157724528	3.21E-05
ENSG00000174038	50.59241908	4.277279233	1.02906668	4.156464608	3.23E-05
ENSG00000134690	1736.265876	-2.218146516	0.533948086	-4.154236288	3.26E-05
ENSG00000163512	1376.416492	1.725001031	0.415946825	4.147167205	3.37E-05
ENSG00000198380	6904.488596	1.200801294	0.289571429	4.146822428	3.37E-05
ENSG00000129673	26.26951331	-3.121706788	0.75301112	-4.145631725	3.39E-05
ENSG00000140986	64.83030402	3.397413541	0.819878738	4.143800034	3.42E-05
ENSG00000164220	63.18812915	3.27534807	0.79077002	4.141972995	3.44E-05
ENSG00000068323	2792.441865	1.481473759	0.357884419	4.139531308	3.48E-05
ENSG00000181652	309.783615	1.89192357	0.457406164	4.136200422	3.53E-05
ENSG00000109083	662.1373892	1.387198551	0.335417973	4.135731122	3.54E-05
ENSG00000120549	2248.124432	1.946714417	0.470714741	4.135656367	3.54E-05
ENSG00000100714	2457.898528	-2.26506748	0.547808363	-4.134780761	3.55E-05
ENSG00000247199	24.24757132	3.821689003	0.924563391	4.133506733	3.57E-05
ENSG00000065833	9157.211265	1.934000286	0.468507948	4.127998882	3.66E-05
ENSG00000164509	200.5001307	2.411290295	0.584329883	4.126590762	3.68E-05
ENSG00000135241	2131.306328	1.525718945	0.369754802	4.1262992	3.69E-05
ENSG00000128951	1193.811628	-2.048398921	0.49653891	-4.125354288	3.70E-05
ENSG00000158793	2128.257529	1.328065588	0.321968765	4.124827413	3.71E-05
ENSG00000226430	11.0016933	5.949105635	1.443111541	4.122415673	3.75E-05
ENSG00000260916	236.438147	1.236033133	0.300036697	4.119606513	3.80E-05
ENSG00000197587	163.1696274	-2.192626961	0.53227905	-4.119318539	3.80E-05
ENSG00000171793	2437.733594	-1.540919246	0.374319826	-4.116584645	3.85E-05
ENSG00000082516	1866.497644	-1.489565704	0.361873474	-4.116261098	3.85E-05
ENSG00000157514	1083.774656	1.344885295	0.326969366	4.113184405	3.90E-05
ENSG00000171786	85.94297304	2.574760467	0.626253661	4.111369922	3.93E-05
ENSG00000103995	956.2978494	-1.410318312	0.343043438	-4.111194545	3.94E-05
ENSG00000168564	891.5364511	1.814157902	0.441790096	4.106379743	4.02E-05
ENSG00000196998	834.5370927	1.688399058	0.411429353	4.103739911	4.07E-05
ENSG00000132361	4650.225737	-1.435888432	0.349943415	-4.103201752	4.07E-05
ENSG00000137960	49.37073811	4.543073692	1.107283203	4.102901298	4.08E-05
ENSG00000173530	6572.646606	1.501788403	0.366034068	4.102865099	4.08E-05
ENSG00000164611	2421.422568	-1.908508725	0.465169796	-4.10282168	4.08E-05
ENSG00000184445	2348.981459	-1.77600863	0.433253847	-4.099233375	4.15E-05
ENSG00000260655	37.05406985	-3.356814034	0.819952639	-4.093912106	4.24E-05
ENSG00000151929	15078.90779	2.430792512	0.594104837	4.091521158	4.29E-05
ENSG00000158683	74.44429166	3.06721761	0.74968169	4.09135991	4.29E-05
ENSG00000141556	3433.558178	-1.907383942	0.46621444	-4.091215929	4.29E-05
ENSG00000159348	1905.045037	2.284142157	0.558371595	4.090720549	4.30E-05
ENSG00000187800	1255.473712	1.211938345	0.296334177	4.08976905	4.32E-05
ENSG00000183763	394.9065876	-1.872347416	0.457968303	-4.088377743	4.34E-05
ENSG00000177595	871.5218813	-1.971529687	0.48241191	-4.086818023	4.37E-05
ENSG00000165861	1229.498448	1.806189606	0.442079927	4.085663007	4.40E-05

ENSG00000106628	4251.541704	-1.495253888	0.366112017	-4.084143157	4.42E-05
ENSG00000154920	524.0450128	-2.186012913	0.535320745	-4.083557257	4.44E-05
ENSG00000185015	882.011814	1.445386387	0.353957384	4.083503982	4.44E-05
ENSG00000112039	598.1212421	-1.719554471	0.421139226	-4.083102131	4.44E-05
ENSG00000187456	95.09741601	-2.340570701	0.573325161	-4.082448947	4.46E-05
ENSG00000185924	369.00314	-1.905352564	0.467114485	-4.078984112	4.52E-05
ENSG00000164695	1050.972223	1.338890355	0.328525224	4.075456792	4.59E-05
ENSG00000074047	442.8580745	-1.772927938	0.435177689	-4.074032248	4.62E-05
ENSG00000163393	631.8461516	1.659093154	0.407319997	4.073193479	4.64E-05
ENSG00000104886	1104.73006	-1.548764232	0.380517201	-4.070155645	4.70E-05
ENSG00000171124	68.67553889	2.412476874	0.592738372	4.070053479	4.70E-05
ENSG00000243477	243.1106116	-2.197443334	0.540276754	-4.067255011	4.76E-05
ENSG00000131620	1965.82953	-1.229408725	0.302276358	-4.067167976	4.76E-05
ENSG00000164053	408.0615336	-1.472473857	0.362059573	-4.066938053	4.76E-05
ENSG00000183508	607.9702416	1.895403702	0.466179635	4.065822613	4.79E-05
ENSG00000185818	631.5178012	-1.448422275	0.356374555	-4.064325732	4.82E-05
ENSG00000180353	33.87322088	3.192872995	0.785607926	4.0642067	4.82E-05
ENSG00000165905	1641.063976	-2.14537053	0.528525039	-4.059165358	4.92E-05
ENSG00000256304	53.96776928	3.24859709	0.801297621	4.054170391	5.03E-05
ENSG00000261761	77.37367175	2.252477563	0.555797824	4.052692306	5.06E-05
ENSG00000164818	1973.29941	-1.515114485	0.373870845	-4.052507721	5.07E-05
ENSG00000164683	141.8103611	1.652621532	0.407919411	4.051343196	5.09E-05
ENSG00000033100	2542.624448	2.394175903	0.591253882	4.049319549	5.14E-05
ENSG00000136108	3402.106539	-1.624485117	0.401979037	-4.041218494	5.32E-05
ENSG00000180210	16.74832646	4.611794937	1.141641865	4.039616168	5.35E-05
ENSG00000128050	11991.84789	-2.115637764	0.523726663	-4.03958384	5.35E-05
ENSG00000120129	1465.810963	1.576848622	0.390507438	4.037947729	5.39E-05
ENSG00000169429	692.5551363	1.762633784	0.436525756	4.037868919	5.39E-05
ENSG00000164970	3541.135336	2.36496935	0.585749867	4.037507275	5.40E-05
ENSG00000100526	880.2725412	-2.010526773	0.498158004	-4.035921845	5.44E-05
ENSG00000231940	33.76133962	2.709376008	0.671414134	4.035327632	5.45E-05
ENSG00000179958	1283.115609	-1.70529968	0.423091516	-4.030569313	5.56E-05
ENSG00000198176	4670.006408	-1.482952809	0.367927047	-4.030562095	5.56E-05
ENSG00000173894	1535.084626	-1.680575091	0.41700414	-4.030116083	5.57E-05
ENSG00000135776	839.4735784	-1.496626155	0.371656307	-4.02690907	5.65E-05
ENSG00000237979	9.00722824	6.641171185	1.649392051	4.026435789	5.66E-05
ENSG00000083799	2268.76395	2.111496587	0.5245637	4.025243433	5.69E-05
ENSG00000167513	2334.135605	-1.669424376	0.414897353	-4.023704576	5.73E-05
ENSG00000116977	3185.704933	1.831368243	0.455510464	4.020474588	5.81E-05
ENSG00000263934	131.0637971	1.907566719	0.474580841	4.019476879	5.83E-05
ENSG00000241634	62.49762143	-2.304060774	0.573373768	-4.018427251	5.86E-05
ENSG00000167748	38.73771557	-2.766530019	0.688644243	-4.017357359	5.89E-05
ENSG00000225855	312.017207	-1.354235471	0.337324663	-4.014635213	5.95E-05
ENSG00000167523	472.8932888	-1.433879852	0.357252777	-4.013628287	5.98E-05
ENSG00000166483	1758.372132	-1.58325134	0.394513525	-4.013173789	5.99E-05
ENSG00000137404	265.4679374	-1.614312472	0.402415899	-4.01155242	6.03E-05
ENSG00000171889	85.28175321	2.975311828	0.741937573	4.010191608	6.07E-05

ENSG00000116254	803.7376425	-1.740252259	0.433964503	-4.01012582	6.07E-05
ENSG00000130449	1763.219855	1.649294669	0.411341926	4.009546714	6.08E-05
ENSG00000197063	8711.344342	1.751097414	0.436808086	4.008848445	6.10E-05
ENSG00000261094	90.84508358	2.015459768	0.503114537	4.005966077	6.18E-05
ENSG00000258010	7.2043708	6.327017156	1.580071037	4.004261205	6.22E-05
ENSG00000085563	715.6812857	2.111567617	0.527467096	4.003221498	6.25E-05
ENSG00000167775	1084.83107	-1.506852889	0.376441051	-4.002892044	6.26E-05
ENSG00000204604	1235.063872	1.72878882	0.432071925	4.00115981	6.30E-05
ENSG00000237813	40.91538682	-2.456999599	0.614428263	-3.998838834	6.37E-05
ENSG00000070814	3810.03069	-1.39827009	0.349672037	-3.99880443	6.37E-05
ENSG00000258667	17.02072775	3.102323984	0.776376271	3.99590263	6.44E-05
ENSG00000163009	80.81768703	-2.765650817	0.69227769	-3.99500209	6.47E-05
ENSG00000051341	761.2903452	-1.617569306	0.404914314	-3.994843476	6.47E-05
ENSG00000078081	578.6704744	1.116042921	0.279512926	3.99281328	6.53E-05
ENSG00000188522	5429.366165	1.529210571	0.383253771	3.990073124	6.61E-05
ENSG00000258469	63.98991248	2.733402989	0.685059499	3.990022757	6.61E-05
ENSG00000088325	6651.086808	-1.814110618	0.454820248	-3.988632046	6.65E-05
ENSG00000262943	1164.492419	2.314663905	0.580734458	3.985752652	6.73E-05
ENSG00000171790	156.1938967	-1.955048703	0.490522995	-3.985641288	6.73E-05
ENSG00000144554	1527.099961	-1.843120365	0.462471333	-3.985372137	6.74E-05
ENSG00000073331	1239.149214	1.181075681	0.29642298	3.984426845	6.76E-05
ENSG00000258521	24.43359117	3.853200624	0.967440802	3.982880004	6.81E-05
ENSG00000169710	32120.94722	-1.783014509	0.447689922	-3.982699677	6.81E-05
ENSG00000132600	671.6879561	-1.399497919	0.351602119	-3.980345518	6.88E-05
ENSG00000117000	3540.265923	1.244274125	0.312687666	3.979287511	6.91E-05
ENSG00000182400	2242.226516	1.119346783	0.281344617	3.978561227	6.93E-05
ENSG00000136866	286.8354626	2.206957352	0.554793229	3.977981769	6.95E-05
ENSG00000118523	3070.479512	2.460570305	0.61869255	3.977048546	6.98E-05
ENSG00000139508	356.8810169	1.691975411	0.425456483	3.976847173	6.98E-05
ENSG00000090889	1399.592542	-1.726604971	0.434263152	-3.975941692	7.01E-05
ENSG00000175643	310.6322628	-1.968429086	0.495195829	-3.975051831	7.04E-05
ENSG00000187951	966.5938761	-1.620391191	0.407972424	-3.971815485	7.13E-05
ENSG00000146587	3093.871471	1.308822598	0.329642993	3.97042445	7.17E-05
ENSG00000115053	33371.67803	-1.424226786	0.358811083	-3.969294297	7.21E-05
ENSG00000139890	72.07967176	1.971245766	0.496744125	3.968332322	7.24E-05
ENSG00000197044	218.3645698	2.531629746	0.638401903	3.965573617	7.32E-05
ENSG00000184967	756.1443218	-1.561309621	0.39375051	-3.965225648	7.33E-05
ENSG00000112763	1589.620042	1.721990504	0.4343714	3.964327538	7.36E-05
ENSG00000162302	2196.999884	-1.4277276	0.360458486	-3.960865552	7.47E-05
ENSG00000138075	15.41752634	3.891356594	0.983238749	3.957692469	7.57E-05
ENSG00000129596	16.67401337	3.813448089	0.963607234	3.95747142	7.57E-05
ENSG00000197329	836.4818835	1.265633796	0.320022834	3.954823411	7.66E-05
ENSG00000178878	142.2062976	-1.619437208	0.409698156	-3.95275689	7.73E-05
ENSG00000120437	783.0036124	-1.627476876	0.412412537	-3.946235217	7.94E-05
ENSG00000234231	238.7554659	2.229241246	0.565134715	3.944619198	7.99E-05
ENSG00000184916	2594.029596	-2.17408052	0.551554042	-3.941736173	8.09E-05
ENSG00000117593	1116.370993	-1.605755681	0.407895731	-3.936681753	8.26E-05

ENSG00000110074	906.7018757	-1.487413616	0.377869934	-3.936311101	8.27E-05
ENSG00000131462	3616.830591	-1.743279181	0.443062274	-3.93461435	8.33E-05
ENSG00000003393	5450.822554	2.028038911	0.515626467	3.933155182	8.38E-05
ENSG00000111981	1069.621128	1.410877486	0.35888023	3.93133243	8.45E-05
ENSG00000161010	2517.868846	1.406504306	0.357784904	3.931144912	8.45E-05
ENSG00000172731	955.0801918	-1.810227773	0.460548972	-3.930586934	8.47E-05
ENSG00000108433	1384.840162	1.772113832	0.451043784	3.92891754	8.53E-05
ENSG00000118894	354.1362162	-1.626362925	0.413976043	-3.928640205	8.54E-05
ENSG00000135709	1953.728093	1.809138369	0.46064673	3.927387849	8.59E-05
ENSG00000249115	953.6522344	-1.457970007	0.371362208	-3.926005329	8.64E-05
ENSG00000179041	905.6055245	-1.926441158	0.490772887	-3.925321077	8.66E-05
ENSG00000111886	55.57901649	2.024976339	0.51598787	3.924465008	8.69E-05
ENSG00000204498	759.2367871	1.171890211	0.298829539	3.921600969	8.80E-05
ENSG00000172009	2978.541796	-1.392438218	0.355267702	-3.919405591	8.88E-05
ENSG00000166471	5327.867078	1.027750623	0.262383286	3.91698206	8.97E-05
ENSG00000158865	92.69379027	2.548044545	0.650948451	3.914356875	9.06E-05
ENSG00000070190	86.61202234	2.256016327	0.576445955	3.913664943	9.09E-05
ENSG00000185507	761.0743934	1.506507318	0.384979963	3.913209684	9.11E-05
ENSG00000214776	73.12179536	-1.715667964	0.438794388	-3.909958766	9.23E-05
ENSG00000164087	761.1958344	-1.916448381	0.490194341	-3.909568555	9.25E-05
ENSG00000069482	1190.354884	-1.543132327	0.394894583	-3.907707	9.32E-05
ENSG00000215154	257.8033246	2.149042787	0.550139701	3.906358302	9.37E-05
ENSG00000131473	9903.438101	-1.072703735	0.274681918	-3.90525792	9.41E-05
ENSG00000261324	29.65946015	2.943632016	0.753778129	3.905170371	9.42E-05
ENSG00000188785	687.952974	1.89952518	0.486431708	3.905019246	9.42E-05
ENSG00000184886	385.7711053	-1.474361896	0.377566037	-3.904911334	9.43E-05
ENSG00000236081	193.4818192	-2.772628851	0.710583063	-3.90190675	9.54E-05
ENSG00000058804	2576.15617	-1.360703989	0.348890402	-3.900090059	9.62E-05
ENSG00000084774	3984.863799	-2.291137802	0.587532219	-3.899595165	9.64E-05
ENSG00000175792	3438.577096	-1.605333562	0.411766932	-3.898646144	9.67E-05
ENSG00000197857	488.5892763	2.101867636	0.539187792	3.89821073	9.69E-05
ENSG00000140297	177.941695	1.771683031	0.454500314	3.898089783	9.70E-05
ENSG00000197355	508.2601538	-1.191387907	0.30571712	-3.897027115	9.74E-05
ENSG00000106245	2125.909002	1.33015527	0.341452079	3.895584046	9.80E-05
ENSG00000233785	27.5300971	2.5652775	0.658710302	3.894394081	9.84E-05
ENSG00000147536	569.6920402	-1.524544355	0.391914763	-3.88998961	0.000100249
ENSG00000231007	195.3954388	-2.548633966	0.655375473	-3.888814994	0.000100735
ENSG00000229729	272.7698432	1.667817901	0.428938071	3.888248712	0.00010097
ENSG00000160570	3811.062792	2.001213589	0.514909488	3.886534697	0.000101685
ENSG00000255874	74.0098683	1.560948193	0.401659533	3.886247098	0.000101806
ENSG00000142528	2392.299945	1.946058042	0.501065801	3.883837288	0.000102821
ENSG00000128016	7621.830347	1.539149186	0.396593598	3.880922925	0.000104061
ENSG00000132510	4401.610786	1.464578345	0.377622519	3.878418985	0.000105138
ENSG00000166963	232.4814898	-1.618268711	0.417296241	-3.87798535	0.000105325
ENSG00000138182	1594.27776	-1.533911342	0.395606817	-3.877363276	0.000105595
ENSG00000146263	1578.468905	-1.603010431	0.413683751	-3.87496591	0.00010664
ENSG00000177410	8422.421074	1.251630043	0.323044718	3.874479211	0.000106853

ENSG00000225190	2615.414675	1.784547758	0.460596565	3.874426979	0.000106876
ENSG00000136436	8025.716769	1.397984476	0.360837094	3.874281495	0.00010694
ENSG00000100564	1090.893497	1.262906405	0.326093938	3.872830058	0.000107579
ENSG00000142731	1176.696059	-1.671800726	0.431781247	-3.871869693	0.000108004
ENSG00000173230	7804.402919	1.70029047	0.439337922	3.870119979	0.000108782
ENSG00000182010	517.6408488	-1.293953544	0.334403497	-3.869437841	0.000109087
ENSG00000215717	1868.053653	1.388822527	0.358962881	3.868986464	0.000109289
ENSG00000113739	5815.118299	1.413255841	0.365311474	3.868632498	0.000109447
ENSG00000005471	14.37010757	6.342552331	1.639946651	3.867535768	0.000109941
ENSG00000147121	682.8154893	1.539723017	0.398297013	3.865765913	0.000110741
ENSG00000173875	2777.066421	1.726566625	0.446942982	3.863057918	0.000111976
ENSG00000102547	632.6310232	2.104902117	0.545067307	3.861728798	0.000112588
ENSG00000053438	45.92721949	2.971807919	0.770351787	3.857728339	0.000114446
ENSG00000107249	607.563273	1.956121546	0.507204681	3.85667092	0.000114942
ENSG00000227218	28.45557779	2.472772692	0.641729778	3.853292735	0.00011654
ENSG00000198270	257.3301578	1.608487269	0.417886273	3.849102911	0.000118551
ENSG00000183765	1105.455873	-1.518761374	0.394843381	-3.846490654	0.000119822
ENSG00000169548	271.746216	2.365418581	0.615222414	3.844818601	0.000120642
ENSG00000118620	1028.130751	1.973029224	0.513614316	3.841460729	0.000122304
ENSG00000118193	1618.147536	-1.829012421	0.476682358	-3.836962689	0.000124565
ENSG00000162231	5732.01513	1.915480204	0.499259241	3.836644467	0.000124727
ENSG00000197780	2436.625241	1.96054128	0.511541998	3.832610589	0.000126791
ENSG00000138074	1474.560273	-1.435131695	0.374487447	-3.832255814	0.000126974
ENSG00000205853	17.50128003	3.916775978	1.022134618	3.831957073	0.000127128
ENSG00000116237	3565.895933	-1.26175633	0.32943548	-3.830055983	0.000128114
ENSG00000114771	308.0585769	1.70340651	0.444868261	3.829013348	0.000128658
ENSG00000121101	283.0895718	2.217384226	0.579718713	3.824931259	0.000130809
ENSG00000125657	3847.151204	1.996698347	0.522194667	3.823666677	0.000131482
ENSG00000104221	816.8829305	2.541284564	0.664703712	3.823183954	0.000131739
ENSG00000188878	168.5487899	-1.709993111	0.447371008	-3.822315439	0.000132204
ENSG00000185347	580.108409	-1.794150318	0.469524831	-3.821204332	0.000132802
ENSG00000135951	295.2908464	1.721929053	0.450706412	3.820511556	0.000133175
ENSG00000088726	539.0131561	1.395549302	0.36536	3.819655408	0.000133638
ENSG00000142733	769.4743876	-1.511117565	0.395662535	-3.819208112	0.000133881
ENSG00000163002	716.3864465	-1.398971581	0.366532041	-3.816778413	0.000135205
ENSG00000235448	66.17853051	2.84379421	0.745259298	3.815845329	0.000135718
ENSG00000235217	49.04005117	2.086577054	0.547019028	3.814450589	0.000136486
ENSG00000197822	1477.184166	1.042529388	0.273346884	3.81394283	0.000136767
ENSG00000143179	2752.559453	-1.353998856	0.355315733	-3.810692102	0.000138578
ENSG00000124151	4747.131552	1.224729256	0.321500494	3.809416409	0.000139295
ENSG00000048471	1145.018731	2.034912793	0.534456925	3.807440224	0.000140413
ENSG00000196262	41824.149	-1.174081628	0.308586063	-3.804713719	0.000141968
ENSG00000169359	2627.328112	0.975943544	0.256605328	3.803286355	0.000142789
ENSG00000170955	269.0880765	-1.901154602	0.499977117	-3.80248323	0.000143253
ENSG00000228960	35.44338294	2.482106975	0.652783468	3.802343498	0.000143334
ENSG00000141560	1195.26619	-1.180794807	0.310552668	-3.802236879	0.000143396
ENSG00000198798	37.02972594	2.444943326	0.643140299	3.801570712	0.000143782

ENSG00000100652	20.3651201	4.018981774	1.057745152	3.799574753	0.000144945
ENSG00000156398	408.4870263	-1.852414431	0.487630898	-3.798804461	0.000145396
ENSG00000154258	41.07751192	3.942214208	1.03791168	3.798217405	0.00014574
ENSG00000159958	28.87492414	-3.551250044	0.936382568	-3.792520457	0.000149126
ENSG00000200879	62.7491033	2.342885844	0.617768457	3.792498332	0.000149139
ENSG00000177084	3958.426403	-1.535624031	0.404958576	-3.792052128	0.000149408
ENSG00000167981	261.1958097	1.347366516	0.355319328	3.791987686	0.000149446
ENSG00000237510	37.13074072	-2.138463992	0.564008322	-3.791546877	0.000149712
ENSG00000196428	2809.41941	1.540915844	0.40671974	3.788642875	0.000151472
ENSG00000186205	506.3212747	-2.293384265	0.605543269	-3.787316913	0.000152283
ENSG00000162236	2113.727766	1.410400952	0.37251965	3.78611156	0.000153023
ENSG00000128408	108.1164113	-2.44700126	0.646406545	-3.785545304	0.000153372
ENSG00000174123	25.83450471	2.628074739	0.694239433	3.78554518	0.000153372
ENSG00000157851	589.1648655	-1.960678657	0.51801343	-3.784995803	0.000153711
ENSG00000240457	16.46668775	4.583234051	1.211363879	3.783532043	0.000154618
ENSG00000119630	721.8075904	1.799121692	0.475531692	3.783389674	0.000154707
ENSG00000083223	4123.354412	1.659319304	0.438851844	3.781046669	0.00015617
ENSG00000237772	10.7713538	4.456198722	1.178616033	3.780874007	0.000156279
ENSG00000161267	636.7414635	-1.64373266	0.434932401	-3.77928307	0.000157281
ENSG00000107175	2175.988793	1.912004011	0.505954402	3.779004599	0.000157457
ENSG00000092871	1414.821402	1.230790225	0.325692889	3.778990163	0.000157466
ENSG00000107672	1508.12055	-1.809882692	0.479338644	-3.775791318	0.0001595
ENSG00000110063	445.1049849	-1.385346766	0.366995017	-3.774838077	0.000160112
ENSG00000270020	31.13434374	2.074584416	0.549747213	3.773706107	0.00016084
ENSG00000186480	3503.902872	-1.299225751	0.344341631	-3.773071951	0.00016125
ENSG00000267898	22.21875049	3.770777903	0.999701773	3.771902787	0.000162007
ENSG00000215838	8.834078965	6.61351399	1.753873708	3.770803998	0.000162722
ENSG00000146592	486.0598313	1.201029311	0.318521222	3.770641413	0.000162829
ENSG00000116793	1691.070853	1.95978283	0.520054527	3.768417979	0.000164285
ENSG00000166225	2036.113982	1.6630623	0.441364341	3.768003314	0.000164559
ENSG00000179046	1315.828878	1.836411591	0.487883744	3.764035208	0.000167193
ENSG00000058056	1229.755214	-1.408262546	0.374197356	-3.763421955	0.000167604
ENSG00000214706	1637.415866	-1.416600316	0.37651778	-3.762372968	0.000168309
ENSG00000118985	4067.949621	1.31118863	0.34850965	3.762273528	0.000168376
ENSG00000120800	1213.484857	-1.449971942	0.385425192	-3.762006146	0.000168556
ENSG00000196639	972.6974147	1.503156583	0.399713099	3.760588748	0.000169514
ENSG00000102401	2279.097216	1.419340702	0.377535082	3.759493539	0.000170258
ENSG00000143799	10940.37901	-0.978486494	0.260422754	-3.757300313	0.000171756
ENSG00000164880	4166.315366	-1.000633452	0.266369349	-3.756563799	0.000172262
ENSG00000114124	24.61432484	3.415715039	0.909293157	3.756450837	0.00017234
ENSG00000243069	103.9952223	3.483183454	0.927401271	3.755853656	0.000172752
ENSG00000169884	230.4092891	-2.110889163	0.562271699	-3.754215561	0.000173885
ENSG00000131791	3439.150677	1.426993967	0.380136954	3.753894361	0.000174108
ENSG00000092929	372.8288935	-1.431949593	0.381530766	-3.753169397	0.000174613
ENSG00000162073	508.3633601	-1.964726932	0.523517662	-3.752933428	0.000174777
ENSG00000057468	137.3557403	1.94836871	0.51939539	3.751224499	0.000175973
ENSG00000148459	527.6497796	-1.13598791	0.3028455	-3.751047676	0.000176097

ENSG00000237452	133.3879192	-2.032618541	0.541929867	-3.750704038	0.000176339
ENSG00000197062	982.9217075	1.681537303	0.448360198	3.750416098	0.000176541
ENSG00000127081	1023.214484	1.805466196	0.481634463	3.748623353	0.000177808
ENSG00000151006	113.5137515	-1.575940241	0.420470277	-3.748041961	0.00017822
ENSG00000103111	2832.379738	1.454547794	0.388121668	3.747659341	0.000178492
ENSG00000174564	150.0474636	1.553738924	0.414748304	3.746221285	0.000179518
ENSG00000260488	14.99314882	5.429846906	1.449509785	3.745988444	0.000179685
ENSG00000260852	571.4969588	2.164484635	0.578101307	3.744126869	0.000181022
ENSG00000142867	1882.584279	0.977991984	0.261336408	3.742272235	0.000182364
ENSG00000132768	1166.312102	-1.341698541	0.358528255	-3.74223934	0.000182388
ENSG00000179454	1203.31891	1.277583402	0.341411218	3.74206627	0.000182513
ENSG00000237015	103.2925167	2.646443198	0.707496527	3.740574118	0.0001836
ENSG00000164051	761.4940316	-1.547239914	0.413646143	-3.740491578	0.000183661
ENSG00000163993	19.84910796	3.033904557	0.81115533	3.740226372	0.000183855
ENSG00000174177	1214.963371	-1.463610647	0.391835228	-3.735270699	0.000187513
ENSG00000115896	11.25803349	4.6070794	1.233817353	3.734004381	0.000188459
ENSG00000144834	36.21615869	2.814611279	0.753832741	3.733734454	0.000188661
ENSG00000107372	12000.7513	1.474264593	0.394950775	3.732780604	0.000189378
ENSG00000156136	1123.648354	-1.594112555	0.427108163	-3.732339238	0.00018971
ENSG00000138668	9682.262735	-1.592389286	0.426874118	-3.730348642	0.000191215
ENSG00000198829	93.74879371	2.474061162	0.663251678	3.730199625	0.000191328
ENSG00000188971	3308.835876	1.355460397	0.363511504	3.728796432	0.000192396
ENSG00000135111	1022.935861	1.418782168	0.380529759	3.728439457	0.000192669
ENSG00000051825	1313.660391	-1.340458244	0.35953217	-3.728340198	0.000192745
ENSG00000168003	18926.53792	1.661620988	0.445739612	3.727783991	0.000193171
ENSG00000142961	624.5492229	1.070471751	0.287209569	3.727145147	0.000193661
ENSG00000182325	1025.66677	-1.341854094	0.36009126	-3.726427837	0.000194213
ENSG00000158691	163.113658	1.467357323	0.393844863	3.725724165	0.000194755
ENSG00000145817	2871.475006	1.227896168	0.329575705	3.72568775	0.000194783
ENSG00000136052	1036.299877	1.401937796	0.376291038	3.725674154	0.000194794
ENSG00000169683	929.2137333	-2.427548706	0.651863717	-3.724012617	0.000196081
ENSG00000102393	4101.571074	2.562457806	0.688310843	3.722820631	0.000197009
ENSG00000214050	388.6996966	1.178678189	0.316663796	3.722175391	0.000197514
ENSG00000140557	18.31271172	4.124409746	1.108904425	3.719355476	0.000199732
ENSG00000024862	417.17147	1.136176358	0.305593775	3.717930307	0.000200862
ENSG00000120519	613.2667808	1.381477841	0.371750901	3.716138512	0.000202291
ENSG00000178752	325.9197283	-1.507986465	0.405812736	-3.715966336	0.000202429
ENSG00000136270	2846.294731	-1.150133585	0.309514626	-3.715926447	0.00020246
ENSG00000203709	222.8889292	2.812428513	0.756919915	3.715622297	0.000202704
ENSG00000168061	570.733182	-1.599733394	0.430594569	-3.715173176	0.000203065
ENSG00000166173	1663.442619	0.961320513	0.258813291	3.714339822	0.000203735
ENSG00000111897	5138.785594	1.238743875	0.333530129	3.714038904	0.000203977
ENSG00000265511	19.75441981	-3.477532815	0.936921599	-3.711658283	0.000205906
ENSG00000109944	491.2336345	1.338431595	0.360660997	3.711051673	0.0002064
ENSG00000245149	171.2045917	1.575116227	0.424642694	3.70927429	0.000207854
ENSG00000141140	3116.475525	-1.650847612	0.445238174	-3.707785425	0.00020908
ENSG00000072501	4349.559493	-1.28815514	0.347438336	-3.707579182	0.00020925

ENSG00000129282	255.3577715	-2.271678434	0.613217894	-3.704520786	0.000211791
ENSG00000183087	1711.340454	-0.952255204	0.257142769	-3.703215959	0.000212884
ENSG00000142700	14.98526669	4.440597659	1.19937163	3.7024368	0.000213539
ENSG00000144120	630.1292261	-1.193512348	0.322466947	-3.701192815	0.000214588
ENSG00000112715	5278.811655	1.050543003	0.284072503	3.69815097	0.000217176
ENSG00000106052	7445.769756	1.345386418	0.364000355	3.696112924	0.000218926
ENSG00000177646	1469.408169	-1.043323064	0.28247478	-3.693508722	0.000221181
ENSG00000102554	3707.67643	1.018009889	0.275703055	3.692414257	0.000222135
ENSG00000131746	2425.136359	-2.005864024	0.543328093	-3.691809885	0.000222664
ENSG00000271590	29.08982146	2.730216959	0.739560216	3.691676349	0.000222781
ENSG00000117411	1942.350363	-1.531005439	0.414719329	-3.691666465	0.00022279
ENSG00000160117	466.9537383	-1.419486008	0.384519183	-3.691586973	0.000222859
ENSG00000161618	632.2282771	-1.215100013	0.329211416	-3.690941308	0.000223426
ENSG00000196152	322.0794524	1.376633758	0.373072197	3.689992897	0.00022426
ENSG00000182687	25.72525145	3.344483952	0.906473815	3.689553847	0.000224648
ENSG00000174125	139.563934	1.668775959	0.452437452	3.688412513	0.000225658
ENSG00000067167	8408.378514	1.005967899	0.272754662	3.688178567	0.000225865
ENSG00000081237	13.90899411	5.404665764	1.465472612	3.688001892	0.000226022
ENSG00000166582	933.8767936	-1.268953399	0.344148031	-3.687231324	0.000226707
ENSG00000173621	767.9316439	-1.459630777	0.395949486	-3.686406544	0.000227443
ENSG00000100739	8.400549529	5.56111573	1.508860874	3.685638501	0.00022813
ENSG00000100485	1543.678654	1.189663877	0.32284486	3.684939809	0.000228757
ENSG00000145390	3591.765434	1.240513849	0.336865196	3.682523054	0.000230937
ENSG00000172889	1294.10575	-1.819786679	0.494360564	-3.681091924	0.000232237
ENSG00000053372	2551.56183	-1.238239031	0.336649601	-3.678124161	0.000234956
ENSG00000144677	1326.37958	-1.112157113	0.302399278	-3.677777008	0.000235276
ENSG00000185480	459.6560167	-1.667615609	0.453445909	-3.677650579	0.000235392
ENSG00000106268	351.8640906	-1.945799188	0.529676969	-3.673558231	0.000239196
ENSG00000164687	2553.00176	-1.374803604	0.374266077	-3.673332129	0.000239408
ENSG00000089101	24.88413763	2.312909076	0.62965552	3.673292778	0.000239445
ENSG00000101977	30.20780788	2.729758416	0.743522497	3.671386441	0.000241238
ENSG00000102385	49.26959698	-2.093084797	0.57036775	-3.669710981	0.000242825
ENSG00000106105	16639.26691	1.359247434	0.370590379	3.66778932	0.000244657
ENSG00000169946	251.0190341	1.877855422	0.512278623	3.665691555	0.000246671
ENSG00000174130	453.4726197	1.303099925	0.355649943	3.663995874	0.000248311
ENSG00000165271	1696.890428	-1.062496212	0.290205676	-3.661183423	0.000251053
ENSG00000108551	39.95485753	2.503619573	0.684282332	3.658752326	0.000253446
ENSG00000169155	2576.231433	1.652614355	0.451833531	3.657573511	0.000254614
ENSG00000072682	3217.237493	1.35276457	0.370206611	3.654079986	0.000258106
ENSG00000001461	2013.580501	1.486082561	0.406879476	3.652390082	0.000259811
ENSG00000138356	42.17328067	2.901165208	0.794456633	3.651760319	0.000260449
ENSG00000160447	1783.749775	-1.276040976	0.349576106	-3.650252275	0.000261983
ENSG00000099617	396.8195863	-1.20647464	0.330784823	-3.647309541	0.000265001
ENSG00000196155	1440.653344	-1.140099403	0.312596806	-3.647188267	0.000265126
ENSG00000136021	4428.404309	1.239901435	0.340206348	3.644557024	0.000267853
ENSG00000140905	506.426475	-1.94624808	0.534514468	-3.641151353	0.000271422
ENSG00000184459	12.0144644	6.080541942	1.670315077	3.640356256	0.000272261

ENSG00000167393	153.9154172	-1.551498362	0.426230858	-3.640042323	0.000272593
ENSG00000130520	5260.928576	-1.310474073	0.360046818	-3.639732415	0.000272922
ENSG00000160752	2102.229433	-1.174589829	0.32283781	-3.638327951	0.000274414
ENSG00000177943	225.7433744	-1.572479512	0.432574526	-3.635164388	0.000277803
ENSG00000100664	17971.28448	1.519515028	0.418108094	3.6342636	0.000278776
ENSG00000221926	1188.951152	1.041876979	0.286822281	3.632482714	0.000280707
ENSG00000232692	19.88376272	2.741410765	0.755409935	3.629037213	0.00028448
ENSG00000229656	40.04514003	3.507181477	0.966917603	3.627177192	0.000286537
ENSG00000107833	2804.210274	-1.800560469	0.496523828	-3.626332449	0.000287475
ENSG00000147955	2758.930651	-1.673623248	0.461558686	-3.62602481	0.000287818
ENSG00000120742	7945.317329	1.205088351	0.332408427	3.625324311	0.000288599
ENSG00000037897	488.1167553	-1.435122656	0.395932801	-3.624662199	0.000289339
ENSG00000169826	1880.776233	1.294011272	0.357184894	3.622805147	0.000291425
ENSG00000110092	18080.15727	-1.751120497	0.483394982	-3.622545876	0.000291718
ENSG00000101844	1230.780292	1.828416581	0.505006934	3.620577179	0.000293947
ENSG00000174652	1003.256449	1.546015521	0.427097161	3.619821584	0.000294806
ENSG00000112759	2425.596619	-1.914868277	0.529214884	-3.618319016	0.000296523
ENSG00000137812	2185.498856	-1.872155539	0.517435967	-3.618139552	0.000296728
ENSG00000168994	904.0653973	1.549183327	0.428191294	3.617970162	0.000296923
ENSG00000007944	1358.33509	1.34785789	0.372602134	3.617418604	0.000297556
ENSG00000137261	292.4954416	1.839248465	0.50850993	3.616937166	0.00029811
ENSG00000235643	17.73882687	4.124641943	1.140377087	3.616910572	0.00029814
ENSG00000167562	281.6316187	1.720867609	0.475785942	3.616894612	0.000298159
ENSG00000125998	17.28499953	3.488585998	0.964918937	3.615418729	0.000299863
ENSG00000196363	2668.690303	-1.2573695	0.347835022	-3.614844455	0.000300528
ENSG00000148516	598.0971177	1.997247354	0.552595051	3.614305537	0.000301154
ENSG00000167792	3497.80811	-1.34683259	0.372665355	-3.614053662	0.000301447
ENSG00000105953	3405.916852	-1.173725941	0.324858526	-3.613037205	0.000302631
ENSG00000225678	6.551404623	6.188392372	1.713072467	3.612452183	0.000303315
ENSG00000271643	55.24780631	1.909807669	0.528851227	3.611238041	0.000304739
ENSG00000223380	4092.09937	1.425503755	0.394830502	3.610419525	0.000305702
ENSG00000241839	1111.24422	1.579187404	0.437538229	3.609255828	0.000307077
ENSG00000132199	1216.188253	-1.499539538	0.41549383	-3.609053688	0.000307316
ENSG00000156802	6194.277024	-1.584162535	0.439441026	-3.60494911	0.000312214
ENSG00000158717	798.607459	-1.142682507	0.316988383	-3.604808779	0.000312383
ENSG00000003436	1434.640431	1.702091356	0.472173471	3.604800903	0.000312392
ENSG00000132692	378.0334646	1.413717857	0.392220578	3.604394919	0.000312881
ENSG00000196642	2291.994717	-1.238603505	0.343645403	-3.604306925	0.000312987
ENSG00000112029	1175.603428	-1.800101135	0.499448912	-3.604174702	0.000313146
ENSG00000160888	4291.05861	1.065576467	0.295697314	3.603605495	0.000313833
ENSG00000244567	225.6341666	1.771678951	0.491647221	3.603557335	0.000313891
ENSG00000248835	240.2632828	-1.841861444	0.511226371	-3.602829484	0.000314772
ENSG00000074071	2541.941424	-1.505717076	0.417943947	-3.602677078	0.000314957
ENSG00000111799	3392.637581	-1.040425892	0.288802531	-3.602551156	0.000315109
ENSG00000170185	3585.976879	1.776455995	0.493146164	3.602291011	0.000315425
ENSG00000069275	7457.152433	-1.435493652	0.398527536	-3.601993644	0.000315786
ENSG00000129514	236.0306703	1.806238747	0.501576037	3.601126477	0.000316841

ENSG00000186104	1028.720363	1.040950991	0.289091851	3.600762132	0.000317286
ENSG00000204789	1209.152019	1.040515535	0.288983295	3.600607901	0.000317474
ENSG00000172927	3406.608677	-1.768871565	0.491323646	-3.600216637	0.000317952
ENSG00000136122	712.4631865	-1.669827484	0.464127174	-3.597780043	0.000320945
ENSG00000027001	704.9190228	-1.980406794	0.550567939	-3.59702528	0.000321877
ENSG00000002919	1696.652811	1.540226301	0.428250838	3.596551749	0.000322463
ENSG00000156671	3831.997569	1.066784344	0.296738994	3.595025818	0.000324359
ENSG00000169972	516.5167756	-1.354228336	0.376772974	-3.594282051	0.000325287
ENSG00000154144	1886.253725	1.06871484	0.297395627	3.593579537	0.000326166
ENSG00000181690	190.3034954	1.279696603	0.356217941	3.592454101	0.000327578
ENSG00000130783	226.6829144	1.295168864	0.360934769	3.588373789	0.000332747
ENSG00000148339	1445.844221	1.052849044	0.293478783	3.587479243	0.00033389
ENSG00000197417	123.5658025	-1.368518391	0.381518955	-3.587025947	0.000334471
ENSG00000204856	318.4187245	-1.620048316	0.45165774	-3.58689373	0.000334641
ENSG00000174010	1852.52426	1.238591644	0.345351424	3.586467461	0.000335188
ENSG00000183625	21.66622703	3.619205138	1.009164786	3.586337126	0.000335355
ENSG00000138669	117.178483	1.444048247	0.402866389	3.584434658	0.000337809
ENSG00000179603	33.43031122	3.462634267	0.966151299	3.58394619	0.000338442
ENSG00000179119	3158.000296	1.369453189	0.382197872	3.583099987	0.000339541
ENSG00000111716	28081.96043	-1.099920859	0.306981838	-3.583016068	0.00033965
ENSG00000119737	226.0663724	2.061849763	0.575897214	3.580239171	0.00034328
ENSG00000260144	11.12754932	5.094230372	1.42289383	3.580190077	0.000343344
ENSG00000182177	36.45749355	2.985263733	0.833992389	3.579485586	0.000344271
ENSG00000109881	390.70119	-1.559344652	0.43582461	-3.577917852	0.000346342
ENSG00000229320	179.0021878	2.080190965	0.581401934	3.577887934	0.000346382
ENSG00000187961	1215.773198	-1.258152325	0.351796342	-3.576365565	0.000348404
ENSG00000197914	11.99837019	5.290861195	1.47949804	3.576119096	0.000348733
ENSG00000172771	111.8664045	2.087795097	0.583966316	3.575197812	0.000349963
ENSG00000160753	1603.258926	-1.343543571	0.375851898	-3.574662199	0.00035068
ENSG00000122042	1611.955834	1.157737369	0.324035944	3.572867117	0.000353094
ENSG00000257354	11.25583863	3.435695016	0.961887135	3.5718276	0.000354499
ENSG00000143322	6390.282242	1.771926322	0.496136047	3.571452495	0.000355007
ENSG00000262814	564.8419002	-1.31133558	0.367192226	-3.571250933	0.00035528
ENSG00000254237	15.08711995	6.410630455	1.797000265	3.567406516	0.000360532
ENSG00000132182	4120.262473	-1.903749377	0.533761674	-3.566665558	0.000361552
ENSG00000205084	292.0209298	-1.44127176	0.404332376	-3.564571733	0.000364451
ENSG00000176407	5591.949615	1.380974476	0.387730879	3.561682987	0.000368485
ENSG00000184343	31.13514661	-2.725520718	0.765552731	-3.560199849	0.000370573
ENSG00000179520	11.97485056	6.076593829	1.707365302	3.559047277	0.000372203
ENSG00000197714	544.5717629	1.655167076	0.46517058	3.558193803	0.000373414
ENSG00000086619	1252.351724	1.077851209	0.303016737	3.557068233	0.000375017
ENSG00000075131	630.8443326	-1.578549641	0.443848829	-3.556502885	0.000375824
ENSG00000268262	23.21956836	-2.252845816	0.633460486	-3.556410961	0.000375956
ENSG00000272486	117.8661647	1.902759924	0.535206063	3.555191274	0.000377704
ENSG00000198839	1467.680538	1.026418162	0.288799602	3.554084412	0.000379297
ENSG00000269821	619.0371336	1.921803738	0.540840294	3.553366419	0.000380334
ENSG00000112308	13999.79723	1.368627125	0.385348315	3.551662409	0.000382806

ENSG00000184984	8.495961517	5.562682182	1.56643674	3.551169376	0.000383524
ENSG00000238271	36.53364929	3.513545554	0.989446681	3.551020609	0.00038374
ENSG00000100711	1097.101096	-1.26696492	0.356902038	-3.549895445	0.000385384
ENSG00000225975	16.89971967	3.706336006	1.044243566	3.549302217	0.000386254
ENSG00000144566	3603.162987	1.105997771	0.311658298	3.548751238	0.000387063
ENSG00000229956	19.09388676	3.364487512	0.948280323	3.547988323	0.000388185
ENSG00000228393	50.58068328	2.267915601	0.639342103	3.547264589	0.000389253
ENSG00000138433	1032.653881	1.18048974	0.332807353	3.547066279	0.000389547
ENSG00000206344	132.7355566	2.532139855	0.713945077	3.546687185	0.000390107
ENSG00000244405	7502.514183	1.208465058	0.340770612	3.546271352	0.000390724
ENSG00000168140	489.5568173	1.435344479	0.404771262	3.54606321	0.000391032
ENSG00000173110	10.47041123	4.980480602	1.406308084	3.541528815	0.000397815
ENSG00000104368	522.4605036	-1.65015033	0.466065147	-3.540600147	0.000399218
ENSG00000153575	869.0858541	-1.421684774	0.401809533	-3.538205683	0.000402856
ENSG00000107165	26.83456792	2.844410029	0.804971461	3.53355388	0.000410013
ENSG00000182742	329.7081322	-1.487329667	0.421057664	-3.53236574	0.000411859
ENSG00000180900	4564.691491	-1.491183641	0.422158654	-3.532282538	0.000411989
ENSG00000093009	914.0430726	-1.993185492	0.564298003	-3.532150532	0.000412195
ENSG00000137414	2686.806544	1.098810265	0.31119134	3.530979579	0.000414024
ENSG00000036054	2231.964394	1.40097453	0.396841333	3.530314038	0.000415067
ENSG00000125037	1224.504156	1.912878044	0.5418838	3.530052098	0.000415478
ENSG00000160908	1179.018438	1.44951594	0.410623966	3.530032486	0.000415509
ENSG00000197771	6587.869603	-1.273858211	0.360959547	-3.52908857	0.000416994
ENSG00000185163	1252.416741	-1.06218291	0.301043245	-3.528339955	0.000418175
ENSG00000214279	154.9399382	-1.793123226	0.508241419	-3.528093457	0.000418564
ENSG00000160208	3670.915313	-1.138493363	0.32273765	-3.527612486	0.000419326
ENSG00000256060	242.5060367	2.027778865	0.574875163	3.527337753	0.000419761
ENSG00000167723	314.7486378	1.699987789	0.482368799	3.524249065	0.000424685
ENSG00000110002	856.4517273	1.667274884	0.473122769	3.523979384	0.000425117
ENSG00000101361	7555.032561	-0.968444429	0.274822452	-3.523891227	0.000425259
ENSG00000272512	77.61537729	-1.478705591	0.419673578	-3.52346602	0.000425942
ENSG00000155744	1596.374389	1.732596022	0.491858543	3.522549414	0.000427417
ENSG00000273271	83.09803474	2.096470003	0.595378873	3.521236808	0.000429539
ENSG00000138796	921.8579365	-1.655324518	0.470302258	-3.519703528	0.000432029
ENSG00000165943	1203.716053	1.214885569	0.345551126	3.515791091	0.000438446
ENSG00000115641	3661.23212	0.884620958	0.251745648	3.513947372	0.0004415
ENSG00000128272	27395.09862	1.158001394	0.329583234	3.513532475	0.00044219
ENSG00000183779	590.4629875	1.550265871	0.441249809	3.513351934	0.000442491
ENSG00000199177	35.5951561	8.631211772	2.457329776	3.512435269	0.00044402
ENSG00000247400	101.3107794	1.446483855	0.411822636	3.512395217	0.000444087
ENSG00000185056	14.60379282	6.36744933	1.813307153	3.511511726	0.000445566
ENSG00000199024	8.128899358	6.495558898	1.849822274	3.511450256	0.000445669
ENSG00000108561	10181.51555	-1.15551003	0.329131536	-3.510784911	0.000446786
ENSG00000132329	419.0014075	-1.074845169	0.30617428	-3.510566492	0.000447153
ENSG00000196295	770.1660727	1.140100788	0.324766322	3.51052653	0.00044722
ENSG00000163507	1317.722934	-1.719109805	0.48982397	-3.509648181	0.0004487
ENSG00000116731	1849.085812	1.762759837	0.502261642	3.509644558	0.000448706

ENSG00000232273	13.95870664	3.965510115	1.130017567	3.509246431	0.000449378
ENSG00000167011	117.4593703	-1.701515717	0.485134462	-3.507307457	0.000452666
ENSG00000197860	1870.677978	0.898028164	0.25606289	3.507060951	0.000453085
ENSG00000163762	57.77561389	-1.788222295	0.509956124	-3.506619904	0.000453837
ENSG00000109572	8771.573706	1.138183843	0.324667456	3.505691196	0.000455423
ENSG00000140511	1083.671702	1.552448818	0.443229719	3.502582862	0.00046077
ENSG00000206573	686.6975955	2.017466255	0.575999951	3.502545878	0.000460834
ENSG00000160223	566.3857145	-1.403045957	0.400611545	-3.502260413	0.000461328
ENSG00000118496	2945.998695	1.623483571	0.463664644	3.501417654	0.00046279
ENSG00000125871	1202.908668	-1.259036985	0.359596242	-3.501251784	0.000463078
ENSG00000224032	1176.918381	1.068636971	0.305311051	3.500158176	0.000464982
ENSG00000136943	558.8328099	-1.199336693	0.342841161	-3.498228416	0.00046836
ENSG00000205918	91.2023956	1.723914195	0.492987741	3.49687031	0.000470751
ENSG00000115963	1250.484709	0.969749787	0.277414521	3.495670609	0.000472872
ENSG00000236675	121.4875368	2.144528382	0.613559973	3.495222106	0.000473667
ENSG00000188554	7373.342817	1.080594966	0.30917578	3.495082849	0.000473915
ENSG00000186162	336.8759669	1.725976855	0.494025597	3.493699245	0.000476377
ENSG00000260804	359.314467	-1.164688439	0.333446994	-3.492874309	0.000477851
ENSG00000167088	3149.466966	-1.040828834	0.298057947	-3.492035175	0.000479355
ENSG00000167566	1760.457798	1.492205182	0.427346082	3.491795634	0.000479785
ENSG00000151552	864.3633268	-1.101562329	0.315607678	-3.49029002	0.000482497
ENSG00000090013	4665.092592	1.569658676	0.449767782	3.489931337	0.000483145
ENSG00000272452	244.0478785	-1.275639244	0.3659335	-3.485986506	0.000490326
ENSG00000092470	1161.838698	-1.712492169	0.491262487	-3.485900538	0.000490483
ENSG00000185697	784.4402289	-1.603829331	0.460163294	-3.485348252	0.000491497
ENSG00000171241	1561.878385	-1.879399122	0.539431275	-3.484038113	0.000493909
ENSG00000178295	897.1048952	-1.457374977	0.41833848	-3.483722029	0.000494493
ENSG00000116044	6047.252462	0.867747421	0.249167947	3.482580456	0.000496606
ENSG00000188283	395.8332961	1.292399479	0.371202158	3.481659394	0.000498317
ENSG00000121903	748.1618517	1.431122584	0.411099232	3.481209579	0.000499155
ENSG00000135316	11010.4359	-1.152736659	0.331131127	-3.481209002	0.000499156
ENSG00000151849	1045.328115	-1.221805156	0.351016385	-3.480763888	0.000499986
ENSG00000220023	654.810896	1.111385806	0.319352886	3.480118248	0.000501193
ENSG00000101911	1780.982635	-1.539976553	0.442630871	-3.479144029	0.000503018
ENSG00000161638	4538.146413	1.079220542	0.310343125	3.477507489	0.000506099
ENSG00000160072	1675.840571	-0.895558061	0.257574715	-3.476886541	0.000507272
ENSG00000224616	30.02779582	2.69797363	0.776073715	3.476439903	0.000508118
ENSG00000112305	76.32932196	1.508514897	0.434006596	3.475787947	0.000509355
ENSG00000267023	2144.27094	1.50956933	0.434341395	3.475536399	0.000509833
ENSG00000152380	94.91770496	1.791645995	0.515525024	3.475381239	0.000510128
ENSG00000073670	365.4885481	-2.039614415	0.587015814	-3.474547644	0.000511715
ENSG00000145882	710.7332362	-1.22402368	0.35235988	-3.473788449	0.000513165
ENSG00000176896	85.33526813	1.48704276	0.42809206	3.473651809	0.000513427
ENSG00000196517	527.8679859	1.159658061	0.333973281	3.472307895	0.000516004
ENSG00000198862	1685.254871	1.428384049	0.411370721	3.472255015	0.000516106
ENSG00000204929	11.96529517	4.506480285	1.298050118	3.471730578	0.000517115
ENSG00000102524	63.88011494	2.147472523	0.618594727	3.471533829	0.000517494

ENSG00000261556	109.2274756	1.546266482	0.445575524	3.470267996	0.000519939
ENSG00000101945	391.7252013	-2.164557141	0.623769566	-3.470123039	0.00052022
ENSG00000170515	6083.302307	-1.270221187	0.366050759	-3.470068445	0.000520326
ENSG00000160214	1439.794435	-0.984414044	0.283701111	-3.469898448	0.000520655
ENSG00000251015	7.327395975	5.350203831	1.542537622	3.468443009	0.000523484
ENSG00000140350	5529.464484	-1.23017796	0.354733681	-3.467891624	0.000524559
ENSG00000226314	111.6857294	1.59712068	0.460613659	3.467375854	0.000525566
ENSG00000154642	1677.019699	1.19212552	0.34386006	3.4668915	0.000526514
ENSG00000125944	8191.50637	-0.963004494	0.277818989	-3.466301914	0.00052767
ENSG00000119403	1073.030348	-1.801602064	0.519794038	-3.465992166	0.000528279
ENSG00000159082	1493.900698	1.843447988	0.531927289	3.465601456	0.000529047
ENSG00000198700	4414.173488	-0.964854672	0.278410396	-3.465584207	0.000529081
ENSG00000179598	309.0953994	-1.520060361	0.438689924	-3.464999486	0.000530232
ENSG00000272275	94.16038952	1.57418173	0.454602376	3.462766171	0.000534653
ENSG00000111816	704.7816644	1.595636094	0.460814622	3.462642067	0.000534899
ENSG00000013306	7103.475551	-1.141667117	0.32972232	-3.462510874	0.00053516
ENSG00000158560	74.96134165	2.980136452	0.860880788	3.461729538	0.000536716
ENSG00000135763	981.7422034	-1.144590228	0.330770652	-3.460374193	0.000539425
ENSG00000119397	781.3948153	-1.317074599	0.380781445	-3.458872845	0.000542441
ENSG00000130005	1257.608806	-1.659766869	0.479906596	-3.45852064	0.000543151
ENSG00000132481	1376.229461	-1.045440177	0.302374679	-3.45743295	0.000545348
ENSG00000077514	506.7543059	-1.238479643	0.358212119	-3.457391797	0.000545431
ENSG00000139734	1090.069027	-1.686153248	0.487704428	-3.457326101	0.000545564
ENSG00000154721	46.78358526	1.826482561	0.528545594	3.455676449	0.000548914
ENSG00000174844	20.8362302	2.456248767	0.710855581	3.45534147	0.000549596
ENSG00000198618	403.4716943	-1.444062697	0.417971585	-3.454930309	0.000550435
ENSG00000163806	70.64223965	1.885440462	0.545746575	3.454791195	0.000550719
ENSG00000185950	1937.940017	0.888805617	0.257291202	3.454473418	0.000551369
ENSG00000218422	78.38190528	-1.911672826	0.553397512	-3.454429746	0.000551458
ENSG00000198482	732.7288999	1.347728618	0.390166259	3.454241841	0.000551842
ENSG00000105856	1493.825529	1.381800438	0.400120831	3.453457888	0.000553449
ENSG00000246763	56.05732978	1.809499656	0.5244197	3.450479942	0.000559591
ENSG00000257954	39.29936189	2.237709592	0.648815455	3.448915364	0.000562843
ENSG00000173085	490.7609103	-1.793161517	0.520226025	-3.446889294	0.000567081
ENSG00000060140	844.7918525	1.698209011	0.492979503	3.444786243	0.000571511
ENSG00000239218	46.25587122	2.198929058	0.638410291	3.444382225	0.000572366
ENSG00000241945	2399.135505	-1.525736528	0.443031391	-3.443856484	0.00057348
ENSG00000100033	158.2108926	-2.262714045	0.65718964	-3.44301539	0.000575267
ENSG00000169689	1784.498109	-1.216434124	0.353310886	-3.442956815	0.000575391
ENSG00000138778	1531.894657	-1.392448273	0.40451586	-3.442258788	0.000576878
ENSG00000161179	1020.459521	-1.515456673	0.440510309	-3.440229754	0.000581221
ENSG00000263823	14.95992493	3.797362351	1.103911842	3.4399145	0.000581898
ENSG00000164061	169.7311643	-1.285512772	0.373760741	-3.439400209	0.000583005
ENSG00000148334	2549.877418	-1.14644756	0.33332941	-3.439383168	0.000583041
ENSG00000130748	347.6070641	-1.358495273	0.395271967	-3.436862174	0.000588495
ENSG00000137124	2154.108828	-1.54929704	0.451118451	-3.434346425	0.000593985
ENSG00000072364	6688.590613	1.233031708	0.359070212	3.433957114	0.000594838

ENSG00000013441	3417.949768	1.277383952	0.372035522	3.433499963	0.000595842
ENSG00000123091	5602.824445	1.120977698	0.326525967	3.433043043	0.000596847
ENSG00000239672	2234.226366	-1.296719968	0.377729364	-3.432933974	0.000597087
ENSG00000197619	48.95080347	2.085552042	0.607516294	3.432915403	0.000597128
ENSG00000166450	11.97318309	4.643595177	1.352937271	3.432232431	0.000598634
ENSG00000092203	4637.199575	1.621575009	0.472459193	3.432201201	0.000598703
ENSG00000160058	4826.752811	1.574496845	0.458836607	3.431497883	0.000600258
ENSG00000128891	707.9098931	1.666021009	0.485680682	3.430280576	0.000602957
ENSG00000104856	872.5249357	1.311211227	0.382386267	3.429022801	0.000605759
ENSG00000087269	2473.500389	-1.240550106	0.361814928	-3.428686908	0.000606509
ENSG00000184368	39.63860249	2.573866408	0.750713072	3.428562129	0.000606788
ENSG00000158748	20.07792426	-3.297331491	0.961897776	-3.427943772	0.000608171
ENSG00000226652	48.05482871	2.9630348	0.864399292	3.427854265	0.000608372
ENSG00000176142	2013.540676	0.893290744	0.260629133	3.427440107	0.000609301
ENSG00000103978	2830.135807	1.249288488	0.364695425	3.425566658	0.000613518
ENSG00000165997	4653.893609	0.920616033	0.268828087	3.424553004	0.000615812
ENSG00000143199	33.48749361	3.774354452	1.102201317	3.424378463	0.000616207
ENSG00000243667	185.7076258	-1.327547624	0.387789204	-3.423374372	0.000618488
ENSG00000270067	104.5734154	1.611368503	0.470811341	3.422535446	0.0006204
ENSG00000111247	556.8562752	-1.889202835	0.552060964	-3.422090959	0.000621415
ENSG00000124588	3525.208808	1.397753566	0.408602829	3.420812261	0.000624344
ENSG00000164609	3824.234941	1.208568829	0.353299785	3.420802612	0.000624366
ENSG00000159256	2523.219861	1.259111621	0.36807869	3.420767506	0.000624447
ENSG00000184106	6.042387231	6.064662988	1.773056741	3.420456237	0.000625162
ENSG00000130881	873.0478447	-1.121684448	0.327955682	-3.420231783	0.000625678
ENSG00000228389	104.6046633	2.141817487	0.626317992	3.419696567	0.00062691
ENSG00000172167	645.7836276	-1.375020206	0.40211902	-3.419435882	0.000627511
ENSG00000105971	4651.637304	-1.478948677	0.43274695	-3.417583132	0.000631798
ENSG00000104524	321.2885708	-1.872993375	0.548684279	-3.413608604	0.000641086
ENSG00000267681	17.82724222	2.555880847	0.748895476	3.412867255	0.000642832
ENSG00000065268	1601.82807	-1.093811162	0.320539668	-3.412404991	0.000643924
ENSG00000137502	616.6646694	1.255447221	0.368001678	3.411525803	0.000646004
ENSG00000232600	26.60497458	-2.550096996	0.747888532	-3.409728706	0.000650275
ENSG00000172530	1354.948912	1.592072153	0.467017768	3.40901838	0.000651971
ENSG00000156469	1242.460067	-1.213806636	0.356194127	-3.407710971	0.000655102
ENSG00000184992	3547.928712	-1.566683475	0.459960156	-3.406128669	0.000658911
ENSG00000100263	1744.011092	1.872270933	0.54971971	3.405864658	0.000659549
ENSG00000166165	10096.52722	-1.308172489	0.38411222	-3.40570391	0.000659937
ENSG00000136159	1676.340072	-1.197499586	0.351678158	-3.405100829	0.000661396
ENSG00000224271	50.31418697	1.570604691	0.46131439	3.404629743	0.000662538
ENSG00000169193	645.8946895	1.27458007	0.374423814	3.404110589	0.000663799
ENSG00000251136	286.4464032	1.710937577	0.502629131	3.403976155	0.000664126
ENSG00000269946	22.66942031	4.020729754	1.182692206	3.39964171	0.000674742
ENSG00000152049	8.408289084	5.549237385	1.632839332	3.398520158	0.000677515
ENSG00000129810	343.6139087	-1.681297378	0.494816287	-3.397821418	0.000679247
ENSG00000163913	561.4806626	-1.218901981	0.358793913	-3.397220348	0.000680741
ENSG00000143457	2024.837656	1.089218803	0.320738163	3.395975065	0.000683846

ENSG00000140105	7385.394067	1.481994362	0.436401175	3.395944944	0.000683921
ENSG00000069020	308.4757737	1.215010316	0.35780181	3.395763465	0.000684375
ENSG00000155755	704.9956671	-1.449511675	0.426950183	-3.395037014	0.000686193
ENSG00000123416	9057.081532	-1.867389414	0.550040843	-3.395001366	0.000686283
ENSG00000132429	914.3647416	1.360583392	0.400804696	3.39462937	0.000687216
ENSG00000226887	597.94755	-1.771743566	0.521984474	-3.394245718	0.000688179
ENSG00000171960	1605.544079	-1.57767097	0.464896652	-3.393595034	0.000689816
ENSG00000197746	39727.14669	1.324636028	0.390429855	3.39276316	0.000691914
ENSG00000181016	321.9334834	2.461496769	0.72560006	3.392360206	0.000692933
ENSG00000271329	5.103413379	5.825850774	1.718107411	3.390853643	0.000696753
ENSG00000204410	310.1485289	-2.848773282	0.840234625	-3.390449759	0.00069778
ENSG00000131094	123.4068716	-2.390641843	0.705172771	-3.390150529	0.000698543
ENSG00000173276	3404.960422	1.035948672	0.305860514	3.386997097	0.000706621
ENSG00000011485	2599.466938	-1.067398741	0.315185867	-3.38656918	0.000707724
ENSG00000115977	970.6053067	1.49601699	0.442050899	3.384264105	0.000713693
ENSG00000255441	5.347155545	-5.856423028	1.731249857	-3.382771704	0.000717583
ENSG00000216867	9.353666856	-3.745082043	1.107130389	-3.382692842	0.000717789
ENSG00000114784	2169.670358	1.316971404	0.389409241	3.381972654	0.000719673
ENSG00000128881	1303.259476	1.059706337	0.31339091	3.381420149	0.000721122
ENSG00000124207	7491.967117	-1.427935188	0.422299137	-3.381335793	0.000721343
ENSG00000213347	438.8598591	-1.985446148	0.587288258	-3.380701249	0.000723011
ENSG00000125901	1370.772045	-1.237104885	0.365998933	-3.380077848	0.000724653
ENSG00000127928	129.613062	1.578046502	0.466906512	3.379791159	0.000725409
ENSG00000175213	731.3052587	1.509916513	0.446939254	3.378348397	0.000729226
ENSG00000181004	300.5444146	1.253842909	0.371440784	3.375619922	0.000736496
ENSG00000136824	1118.407506	-1.36582972	0.404629673	-3.375505587	0.000736802
ENSG00000257167	39.08266233	-1.917855547	0.568226045	-3.375163042	0.00073772
ENSG00000133119	958.3995994	-1.616289409	0.478955421	-3.374613455	0.000739194
ENSG00000172216	4527.673821	1.18272251	0.350507958	3.37431001	0.00074001
ENSG00000257097	23.18242494	3.093155573	0.916748039	3.374052019	0.000740704
ENSG00000183048	185.2201762	-2.209791346	0.65505139	-3.373462571	0.000742291
ENSG00000132341	17185.78626	-0.995947844	0.295250612	-3.373228715	0.000742922
ENSG00000255468	50.61829235	-2.230866424	0.661426564	-3.372810445	0.000744052
ENSG00000126953	414.9467817	-1.430999715	0.424298036	-3.372628653	0.000744543
ENSG00000104147	250.5751019	-1.50083585	0.445073838	-3.372105308	0.000745959
ENSG00000117475	1990.489605	1.530402913	0.45398977	3.371007483	0.000748938
ENSG00000163462	222.5190569	-1.554056793	0.461264363	-3.36912391	0.000754075
ENSG00000160336	1410.020653	1.493961616	0.443476536	3.368750083	0.000755099
ENSG00000123064	3152.419314	-1.053435166	0.312721603	-3.368603757	0.0007555
ENSG00000171097	346.6142466	-1.252315387	0.371793628	-3.368307824	0.000756311
ENSG00000236256	21.82035072	2.553634773	0.758277344	3.367679113	0.000758038
ENSG00000178977	46.5040505	2.162187191	0.642170841	3.366996837	0.000759916
ENSG00000173566	179.4578048	-1.588198437	0.471999064	-3.364833868	0.000765898
ENSG00000149554	1646.780936	-1.355001392	0.402763991	-3.364256544	0.000767502
ENSG00000234292	11.96438234	4.529618077	1.346933896	3.362910452	0.000771254
ENSG00000127324	15.49772494	2.949943471	0.877605606	3.361354408	0.000775612
ENSG00000155657	438.9341532	1.420647745	0.422714129	3.360776582	0.000777237

ENSG00000134802	187.1027406	-1.371992091	0.408257213	-3.360607103	0.000777714
ENSG00000127870	3786.819753	1.423871418	0.423699081	3.360572355	0.000777812
ENSG00000159231	107.2280494	1.294565357	0.385238379	3.360426757	0.000778222
ENSG00000234329	40.37042898	2.202224264	0.655367973	3.360286668	0.000778616
ENSG00000262503	76.13123826	2.133024736	0.634868949	3.359787464	0.000780025
ENSG00000171867	9820.158476	0.982942619	0.292561239	3.359784174	0.000780034
ENSG00000144535	840.8633783	-0.935014574	0.278390005	-3.358649946	0.000783242
ENSG00000107140	1946.216484	1.644942426	0.489823731	3.358233427	0.000784423
ENSG00000099956	2536.079526	-1.201359114	0.357767107	-3.357936184	0.000785267
ENSG00000114767	1758.896921	-1.676446582	0.499280513	-3.35772484	0.000785868
ENSG00000184545	818.7224819	0.981469356	0.292364615	3.357004601	0.000787918
ENSG00000205485	290.1531772	1.664274178	0.495838419	3.356484927	0.0007894
ENSG00000083720	1335.541911	-1.393823484	0.415284589	-3.356309195	0.000789902
ENSG00000108528	1352.235302	-1.336970962	0.398428332	-3.355612177	0.000791895
ENSG00000261924	92.83300845	3.203788723	0.954862487	3.355235718	0.000792974
ENSG00000223572	160.1228054	-1.726991651	0.514967767	-3.353591745	0.0007977
ENSG00000101280	51.95910915	2.840186318	0.847362174	3.351797386	0.000802888
ENSG00000164404	41.84724828	2.029663829	0.605617127	3.351397671	0.000804047
ENSG00000140990	2648.697098	-1.441798763	0.430225099	-3.351266042	0.00080443
ENSG00000188993	41.58204785	1.946234095	0.580961761	3.35002099	0.000808054
ENSG00000206652	6.827423751	5.254945474	1.56875368	3.349758181	0.000808821
ENSG00000172432	4718.780634	1.412705888	0.421734257	3.349753696	0.000808834
ENSG00000131115	948.4422588	1.592362181	0.475476823	3.348979601	0.000811098
ENSG00000151014	2376.764746	2.071036246	0.618439107	3.348811908	0.000811589
ENSG00000161981	465.6981878	-1.208666205	0.361278519	-3.34552469	0.00082127
ENSG00000048162	1119.038131	-1.110257746	0.331881085	-3.34534806	0.000821794
ENSG00000073536	1124.679344	-1.072531829	0.320626471	-3.345113166	0.00082249
ENSG00000123219	433.1428074	-1.845001849	0.551760066	-3.343848102	0.00082625
ENSG00000080561	427.9431262	1.279811777	0.382873103	3.342652613	0.000829817
ENSG00000116991	1148.12999	1.056660172	0.316115804	3.342636333	0.000829866
ENSG00000156869	658.5570202	0.940426182	0.28134383	3.342622385	0.000829907
ENSG00000115657	229.9573142	1.478849286	0.442460471	3.342330858	0.00083078
ENSG00000259330	132.9612793	-1.226009845	0.366825402	-3.342216316	0.000831123
ENSG00000106689	362.0656351	-1.048830481	0.313830403	-3.342029555	0.000831682
ENSG00000250271	80.54721632	1.773016456	0.530592447	3.341578773	0.000833034
ENSG00000168374	11749.22752	1.247375752	0.373446828	3.340169629	0.000837272
ENSG00000214182	2838.122193	-1.555864892	0.465867388	-3.339716263	0.00083864
ENSG00000156232	1158.617839	1.525694436	0.456901466	3.339219831	0.000840141
ENSG00000095906	1764.399981	-0.87298446	0.261433892	-3.339216856	0.00084015
ENSG00000179091	6381.895057	-1.293006834	0.387427284	-3.337418114	0.000845606
ENSG00000082269	1765.318942	1.22269453	0.366463507	3.336470091	0.000848496
ENSG00000244479	287.6208052	1.494348048	0.447913827	3.336240046	0.000849198
ENSG00000213339	670.780199	-1.399611619	0.419534402	-3.33610691	0.000849605
ENSG00000176024	157.4096019	1.542937827	0.462642537	3.335053967	0.000852828
ENSG00000173193	2189.317581	1.202250399	0.360526433	3.334708055	0.00085389
ENSG00000144485	1078.937326	-1.637194878	0.490989157	-3.334482759	0.000854582
ENSG00000120262	23.39932591	3.400256645	1.019756376	3.334381353	0.000854894

ENSG00000260633	22.94969832	2.105736035	0.631712705	3.333376101	0.000857989
ENSG00000116014	88.26301332	-1.687719658	0.506432562	-3.332565446	0.000860492
ENSG00000184489	588.0736201	-2.240734296	0.672417446	-3.33235598	0.00086114
ENSG00000196950	2127.616032	-1.631182386	0.489519638	-3.332210313	0.000861591
ENSG00000257335	70.39955265	2.02157134	0.606730497	3.331909881	0.000862522
ENSG00000130826	3967.837473	-1.451955284	0.435776587	-3.331879978	0.000862614
ENSG00000132801	180.6851992	1.405207051	0.421755032	3.331808619	0.000862836
ENSG00000204228	307.5747035	-1.835057964	0.550815766	-3.331527669	0.000863707
ENSG00000141577	1114.56146	-1.071364896	0.321740871	-3.329899906	0.000868772
ENSG00000159055	796.8549634	-1.398030942	0.419910644	-3.329353426	0.000870479
ENSG00000171130	787.2603326	-0.977770796	0.293776716	-3.328278729	0.000873844
ENSG00000175216	7884.351713	-1.075827962	0.323300304	-3.327642904	0.000875841
ENSG00000205208	650.760654	-1.534405377	0.461119413	-3.327566209	0.000876082
ENSG00000185298	2319.863998	-0.842589498	0.253283143	-3.32667026	0.000878903
ENSG00000182308	198.5909368	1.978361395	0.594803724	3.326074324	0.000880784
ENSG00000162066	192.4919076	-1.676364561	0.504169824	-3.324999797	0.000884186
ENSG00000167977	5016.967452	1.424693336	0.428526884	3.324630001	0.000885359
ENSG00000094914	1929.301019	-1.132332292	0.340653059	-3.324004473	0.000887348
ENSG00000080802	1787.427843	1.348809301	0.405893114	3.323065251	0.000890341
ENSG00000114854	65.08431359	-2.343672152	0.705329268	-3.322805755	0.000891169
ENSG00000212123	62.94489271	-2.538574973	0.764128673	-3.322182587	0.000893162
ENSG00000111450	1537.557087	-1.088741698	0.327855407	-3.320798356	0.000897604
ENSG00000213160	1604.351097	-1.33263687	0.401417392	-3.319828431	0.000900728
ENSG00000183977	24.9149931	2.217200077	0.667940299	3.319458463	0.000901922
ENSG00000204178	1818.555739	1.665156798	0.501865264	3.317935946	0.000906853
ENSG00000107290	10506.85663	1.245091586	0.375360563	3.317054876	0.000909717
ENSG00000198435	953.0478814	-1.438280411	0.433660262	-3.316606424	0.000911179
ENSG00000163491	98.3152565	2.245327881	0.677748767	3.312920644	0.000923272
ENSG00000234393	18.85439172	3.264864489	0.985755004	3.31204455	0.000926168
ENSG00000111912	1474.236567	1.04977767	0.316974124	3.311871825	0.00092674
ENSG00000162377	894.5587432	-1.174997469	0.354795092	-3.311763595	0.000927099
ENSG00000148362	869.9065072	-1.516407486	0.457892407	-3.311711364	0.000927272
ENSG00000136811	2478.266255	-1.152654158	0.348078413	-3.311478436	0.000928044
ENSG00000198932	95.96758138	1.964369647	0.593223182	3.311350107	0.00092847
ENSG00000265452	28.55462092	2.392273772	0.72310199	3.308349039	0.000938478
ENSG00000213073	132.9971107	2.076389235	0.627774192	3.307541565	0.000941187
ENSG00000110104	979.5412543	-1.556668996	0.470748359	-3.306796437	0.000943694
ENSG00000170100	888.6742229	1.242213783	0.375683735	3.306541288	0.000944554
ENSG00000127616	4390.288404	-0.936411532	0.283324686	-3.305082749	0.000949484
ENSG00000044459	240.2721147	-1.257000714	0.380533937	-3.303255222	0.000955694
ENSG00000181894	369.7367205	1.598387502	0.484027338	3.302266992	0.000959067
ENSG00000106144	2300.806977	-0.909416544	0.275410823	-3.30203633	0.000959856
ENSG00000232725	15.22613123	3.148376916	0.953545642	3.301757964	0.000960809
ENSG00000267586	9.900808325	4.246163524	1.28626982	3.301145265	0.00096291
ENSG00000235109	703.3105563	1.273542444	0.385848253	3.300630323	0.000964679
ENSG00000146410	369.4163207	-1.729340993	0.523987131	-3.300350124	0.000965643
ENSG00000148572	1306.405586	0.903490151	0.273841136	3.299322239	0.000969186

ENSG00000101986	1359.97002	1.598945281	0.484698335	3.298846241	0.000970831
ENSG00000178913	3210.268057	1.012015499	0.30679068	3.298716567	0.000971279
ENSG00000196960	7.319134705	5.345248478	1.621159888	3.297175385	0.000976625
ENSG00000125520	1320.923408	-1.773833054	0.538010223	-3.297024812	0.000977149
ENSG00000102786	2342.581414	1.084644284	0.329046732	3.296322916	0.000979593
ENSG00000152463	30.43387447	2.707591766	0.821569407	3.295633631	0.000982
ENSG00000023318	2777.372723	1.134504957	0.344245305	3.295629428	0.000982015
ENSG00000144579	1441.188413	-1.367402322	0.415050541	-3.294544128	0.000985815
ENSG00000204386	1762.055733	1.319827506	0.400639486	3.294302116	0.000986664
ENSG00000107130	3019.300844	-1.048643661	0.318353627	-3.293958581	0.000987871
ENSG00000187475	7.684278143	5.415669191	1.644344898	3.293511719	0.000989442
ENSG00000136114	133.3406422	1.837844765	0.55805848	3.293283468	0.000990246
ENSG00000139291	486.4290627	-1.182585141	0.359107222	-3.293125476	0.000990802
ENSG00000117036	3131.445106	1.127054628	0.342301715	3.292576631	0.000992738
ENSG00000103202	784.2688483	-1.534280642	0.465999484	-3.292451376	0.000993181
ENSG00000173120	7374.20613	1.469773586	0.44645048	3.292131267	0.000994312
ENSG00000184697	13.53165276	2.784301522	0.845924402	3.291430671	0.000996792
ENSG00000094916	5217.346939	-1.118913192	0.340087439	-3.290075037	0.001001607
ENSG00000235631	8.272534955	4.55356268	1.384307907	3.28941463	0.00100396
ENSG00000125434	129.6285156	-1.76939595	0.5380821	-3.288338251	0.001007807
ENSG00000008838	2642.599687	-0.98926393	0.300892302	-3.287767497	0.001009852
ENSG00000196352	3439.18433	1.136026493	0.345674781	3.286402585	0.001014759
ENSG00000151553	2222.927333	1.303714776	0.396793552	3.285624903	0.001017564
ENSG00000162413	3721.676559	1.582504563	0.481823391	3.284407922	0.001021969
ENSG00000171621	1287.225475	0.972662406	0.296169843	3.28413722	0.001022951
ENSG00000106327	252.5327588	-1.156858837	0.352349061	-3.283274927	0.001026085
ENSG00000260423	28.97537799	-2.342862694	0.713706859	-3.282668037	0.001028297
ENSG00000143314	1666.750659	-1.37822605	0.419989069	-3.281575998	0.001032287
ENSG00000091164	5544.842265	1.329665642	0.40530686	3.280639372	0.001035721
ENSG00000108306	607.903911	1.211607375	0.369401697	3.279918265	0.001038372
ENSG00000105281	12027.4881	1.089788932	0.332271178	3.279817829	0.001038741
ENSG00000144040	1150.132033	1.754232749	0.535014083	3.278853407	0.001042298
ENSG00000177575	4.777914417	5.730020341	1.748011419	3.27802226	0.001045372
ENSG00000126775	1811.3172	1.330783865	0.40620488	3.276139532	0.001052366
ENSG00000133878	7.881052173	4.558457949	1.391534004	3.275850921	0.001053442
ENSG00000160767	1946.171405	-1.039037237	0.31727892	-3.274838547	0.001057224
ENSG00000177156	4200.26736	-0.896861624	0.27387887	-3.274665268	0.001057872
ENSG00000109270	1565.442152	1.403860741	0.428802592	3.273909176	0.001060707
ENSG00000178531	651.9840103	-2.309910311	0.705977024	-3.271934119	0.001068145
ENSG00000100330	3549.074127	1.077600311	0.329350279	3.271897369	0.001068283
ENSG00000028203	3558.661057	1.191549513	0.364194102	3.271743026	0.001068867
ENSG00000240602	174.2045618	1.092986326	0.334079577	3.271634667	0.001069277
ENSG00000151687	72.69454385	1.906162496	0.582686481	3.271334688	0.001070411
ENSG00000147535	1768.552389	1.530469423	0.467907808	3.270878146	0.001072141
ENSG00000112667	1033.488771	-1.693569101	0.51786575	-3.270285976	0.001074388
ENSG00000161999	860.8321097	-0.984105446	0.300991893	-3.269541366	0.00107722
ENSG00000198538	1182.015605	1.222071827	0.374057033	3.267073517	0.001086655

ENSG00000160014	9112.669137	-1.106270283	0.338725001	-3.265983561	0.001090846
ENSG00000091483	2112.865864	-1.035944221	0.317211832	-3.265780522	0.001091628
ENSG00000154035	618.1813738	1.364229316	0.417814976	3.265151787	0.001094054
ENSG00000164879	44.21482044	1.728116143	0.52935771	3.264552707	0.001096371
ENSG00000159259	1317.076352	-1.400284549	0.428953403	-3.264421123	0.00109688
ENSG00000006625	1296.236852	-1.231653773	0.37730082	-3.26438138	0.001097034
ENSG00000223802	22.78146534	2.393173757	0.733136858	3.264293333	0.001097375
ENSG00000213465	394.3319979	-1.426489702	0.43704647	-3.263931411	0.001098778
ENSG00000198298	106.1056944	-1.739046373	0.532866414	-3.263569116	0.001100183
ENSG00000136802	3660.702507	1.589847207	0.487193099	3.263279407	0.001101309
ENSG00000180011	861.4229832	-1.206224973	0.369646188	-3.263187909	0.001101664
ENSG00000080815	3225.777243	1.074199305	0.329244742	3.262616426	0.001103888
ENSG00000173812	25957.27616	1.188655375	0.364347083	3.262425938	0.00110463
ENSG00000180423	158.2534851	1.466007425	0.449420212	3.26199709	0.001106303
ENSG00000135454	2760.735259	1.675630817	0.513754666	3.26153888	0.001108092
ENSG00000165312	538.3442083	0.913753173	0.280201645	3.261055711	0.001109982
ENSG00000102743	428.2135415	-1.572104387	0.482126139	-3.26077402	0.001111086
ENSG00000116209	4653.877625	1.261842023	0.387003374	3.26054528	0.001111982
ENSG00000260751	123.3348044	2.211648088	0.678410325	3.260044851	0.001113946
ENSG00000048405	2112.820871	1.168712048	0.358665733	3.258499322	0.001120031
ENSG00000224597	1196.096165	1.485289567	0.455834084	3.258399533	0.001120425
ENSG00000105642	71.74730279	1.592137727	0.488674751	3.258072414	0.001121718
ENSG00000253304	308.4599098	-1.36066064	0.417674849	-3.257703073	0.001123179
ENSG00000075043	1160.484856	-1.890252866	0.580374404	-3.256954222	0.001126146
ENSG00000153574	1234.133118	-1.550835177	0.476419873	-3.255185739	0.001133182
ENSG00000187833	34.45774998	-2.757451108	0.847238046	-3.254635601	0.001135379
ENSG00000158234	301.8148691	-0.990880365	0.304478952	-3.254347658	0.001136531
ENSG00000154342	146.080568	-1.145845714	0.352127246	-3.254067183	0.001137654
ENSG00000269609	504.3471282	-1.30551587	0.401230061	-3.253783793	0.001138789
ENSG00000165388	159.3099557	-2.433249772	0.747852172	-3.253650739	0.001139323
ENSG00000012124	219.3467324	-1.533113611	0.471272798	-3.253134101	0.001141396
ENSG00000119878	1203.338271	1.35032146	0.415300478	3.251432473	0.00114825
ENSG00000163659	4432.51809	1.27217088	0.39134444	3.250770295	0.001150928
ENSG00000267265	144.1789058	1.664782761	0.512255828	3.249904968	0.001154436
ENSG00000057757	4617.278548	1.909426216	0.587571147	3.249693633	0.001155294
ENSG00000134070	526.4092418	1.395109074	0.429335158	3.249463844	0.001156228
ENSG00000135077	41.04202824	2.712719589	0.834882996	3.249221272	0.001157214
ENSG00000103067	1417.909681	-1.23480635	0.380047424	-3.249084906	0.001157769
ENSG00000080608	1410.466381	-1.223157472	0.376606979	-3.247835388	0.001162865
ENSG00000140983	1571.314108	-1.050493411	0.323473102	-3.24754486	0.001164053
ENSG00000160285	1924.479693	-1.75954521	0.542015521	-3.246300415	0.001169154
ENSG00000162923	8923.216735	1.281346249	0.394743055	3.246026078	0.001170281
ENSG00000237594	15.88810851	-2.719400618	0.837876182	-3.245587683	0.001172085
ENSG00000181418	294.6251369	-1.579740184	0.486955761	-3.244114377	0.001178164
ENSG00000136643	2472.924121	1.2417561	0.382790687	3.243955879	0.00117882
ENSG00000228313	16.54210686	2.957143146	0.911585521	3.243955808	0.00117882
ENSG00000123395	1534.151994	1.244143224	0.383565865	3.243623421	0.001180197

ENSG00000139173	405.5089223	1.468539228	0.452766207	3.243482411	0.001180781
ENSG00000108733	425.1892035	1.157184134	0.356886933	3.242439063	0.001185113
ENSG00000257267	1243.43038	1.407854627	0.434340158	3.241364175	0.001189591
ENSG00000069998	1783.796011	-1.172823096	0.361843715	-3.241242138	0.0011901
ENSG00000176244	78.76494179	-1.918893805	0.592217279	-3.240185443	0.00119452
ENSG00000182310	54.99255447	-1.611211614	0.497391206	-3.23932469	0.001198131
ENSG00000242180	115.8866561	-2.014950547	0.622096985	-3.238965297	0.001199642
ENSG00000182208	1016.78182	1.353594214	0.417931636	3.238793373	0.001200365
ENSG00000228782	314.5764606	1.558755793	0.481321165	3.238494187	0.001201625
ENSG00000244625	85.24861614	1.808943827	0.558647084	3.238079779	0.001203372
ENSG00000162695	2912.722477	1.232300877	0.380672861	3.23716504	0.001207236
ENSG00000178409	357.9006129	-0.997937642	0.308276156	-3.23715481	0.001207279
ENSG00000165238	961.8907208	-1.359824646	0.420167121	-3.236389946	0.001210519
ENSG00000184205	2210.551714	1.532069325	0.473427058	3.236125396	0.001211641
ENSG00000235173	28.72085069	-2.518903506	0.778492094	-3.235618611	0.001213794
ENSG00000010610	131.456432	1.679910542	0.519222758	3.235433186	0.001214583
ENSG00000130309	3871.799194	-1.527012742	0.472022718	-3.235040781	0.001216254
ENSG00000149948	6974.351714	-1.707738533	0.527963034	-3.234579739	0.001218219
ENSG00000152684	2718.302553	1.365960593	0.422480899	3.233188998	0.001224165
ENSG00000109111	12039.4813	1.611491433	0.498583834	3.232137352	0.00122868
ENSG00000228106	108.2440257	1.412965315	0.437173674	3.232045748	0.001229074
ENSG00000269175	18.36144919	-2.350606447	0.727283386	-3.232036499	0.001229114
ENSG00000162551	24.08064566	-2.657664077	0.822412879	-3.231544818	0.00123123
ENSG00000156050	222.2962481	1.161879823	0.359564907	3.23134933	0.001232073
ENSG00000197933	465.8130945	1.202456361	0.372435208	3.228632347	0.001243837
ENSG00000090447	729.5809963	-1.366294848	0.42327315	-3.227927043	0.001246908
ENSG00000256673	34.04043296	-2.503994599	0.775789124	-3.227674277	0.00124801
ENSG00000188130	2065.561783	-1.129408951	0.349972674	-3.227134672	0.001250366
ENSG00000037637	2122.968741	1.035352713	0.320879582	3.226608275	0.001252668
ENSG00000186919	33.07532091	2.799940596	0.8680319	3.225619468	0.001257004
ENSG00000119760	3009.803984	1.336583912	0.414420533	3.225187478	0.001258902
ENSG00000100979	315.9961611	1.561694865	0.484321925	3.224497561	0.001261939
ENSG00000144802	683.003368	1.398207366	0.43363355	3.224398495	0.001262376
ENSG00000184990	1307.441444	-1.211992809	0.376251532	-3.22123023	0.001276416
ENSG00000116062	2490.101982	-1.03572329	0.321805128	-3.218479753	0.001288721
ENSG00000175318	322.1543301	-1.266358135	0.393501017	-3.218182626	0.001290057
ENSG00000188033	216.2532763	1.98806835	0.617808135	3.217938122	0.001291157
ENSG00000204406	841.8358572	1.215132924	0.377688086	3.217292173	0.001294067
ENSG00000197905	743.8304572	-1.299239976	0.403863308	-3.217029006	0.001295255
ENSG00000113615	2981.621519	1.07331283	0.333651346	3.216869472	0.001295975
ENSG00000073050	598.4958944	-1.321295408	0.410749099	-3.216794416	0.001296315
ENSG00000100258	1133.546898	-1.027546422	0.319438484	-3.216727083	0.001296619
ENSG00000167601	2913.633393	-1.558864337	0.484673337	-3.216319569	0.001298462
ENSG00000145246	769.1851752	1.428575499	0.444323096	3.215172724	0.001303661
ENSG00000249911	21.45315012	3.412767172	1.06154232	3.21491391	0.001304836
ENSG00000249345	37.08395294	1.702668374	0.529622672	3.214870632	0.001305033
ENSG00000068976	119.6255617	1.821745599	0.56679412	3.214122264	0.001308439

ENSG00000121749	2497.685375	1.243154416	0.386953491	3.212671409	0.001315067
ENSG00000184348	18.19547662	3.41743645	1.063814382	3.212436783	0.001316141
ENSG00000106328	26.20945109	2.289893584	0.713091392	3.211220343	0.001321725
ENSG00000144218	6.768303676	5.229161174	1.629267968	3.209515731	0.001329588
ENSG00000171224	1461.097762	1.793444178	0.558791363	3.209505903	0.001329633
ENSG00000182173	1800.951981	-1.206990934	0.376074311	-3.209447966	0.001329901
ENSG00000089006	4438.768477	-1.210170811	0.377094475	-3.209197934	0.001331058
ENSG00000164403	433.3257382	-1.2493929	0.389386734	-3.208617018	0.00133375
ENSG00000203952	36.87185983	1.745868771	0.544155	3.208403437	0.001334741
ENSG00000162599	667.6917843	-1.1455306	0.357133712	-3.207567813	0.001338625
ENSG00000166024	1038.436145	1.184857809	0.369518667	3.206489728	0.001343651
ENSG00000215190	248.3250398	1.871973989	0.58389787	3.20599558	0.001345961
ENSG00000075407	3407.976608	1.32426636	0.413063315	3.205964585	0.001346106
ENSG00000138696	17.239945	4.001277909	1.248209365	3.205614396	0.001347745
ENSG00000149136	8738.962395	-1.101130182	0.343560555	-3.20505415	0.001350371
ENSG00000028839	1551.178405	1.19527049	0.373120884	3.203440338	0.001357962
ENSG00000184709	10.88179729	-4.136071855	1.291523479	-3.202475155	0.001362521
ENSG00000239382	329.0286322	1.846316856	0.57666004	3.201742321	0.001365991
ENSG00000101236	2018.572958	1.28214031	0.400594955	3.200590255	0.001371464
ENSG00000173545	2353.220142	1.478418515	0.462107859	3.199293165	0.00137765
ENSG00000198089	935.5172672	-1.399382819	0.437463332	-3.198857403	0.001379734
ENSG00000121957	1901.683812	-1.4258157	0.445759756	-3.198619166	0.001380875
ENSG00000171606	1016.884749	1.396480259	0.436599562	3.198537933	0.001381264
ENSG00000048342	405.5424913	-1.388475199	0.434138663	-3.198229774	0.001382741
ENSG00000163959	145.5221076	1.596456917	0.499273681	3.197558728	0.001385962
ENSG00000131781	62.16042423	1.676966515	0.524482829	3.197371625	0.001386861
ENSG00000215883	204.5372145	-1.771723943	0.554274387	-3.1964745	0.001391181
ENSG00000157326	261.1083878	-1.133490563	0.354777426	-3.194934288	0.001398627
ENSG00000116903	2167.009662	1.432380897	0.448377978	3.194583514	0.001400328
ENSG00000236199	31.17976807	2.824911318	0.884548052	3.193621093	0.001405004
ENSG00000255010	12.25295868	4.665724048	1.462003861	3.191321291	0.001416237
ENSG00000185482	112.0334278	-1.525557632	0.47804458	-3.191245539	0.001416608
ENSG00000113389	60.60491003	1.777869366	0.557325468	3.190002014	0.001422718
ENSG00000132330	116.409561	-1.220233013	0.382970962	-3.186228554	0.001441407
ENSG00000198736	1651.725633	1.571565469	0.493238645	3.186217227	0.001441463
ENSG00000124762	23399.83383	1.302856386	0.409166675	3.184170326	0.001451696
ENSG00000105497	937.1393519	1.531457586	0.481053493	3.183549454	0.001454813
ENSG00000166888	2228.565288	-1.264616685	0.397389037	-3.182313974	0.001461033
ENSG00000169813	10197.08253	-0.98304606	0.308987775	-3.1815047	0.001465121
ENSG00000115694	3050.5853	-1.123332206	0.35308397	-3.181487408	0.001465209
ENSG00000173546	1491.653377	-1.280439945	0.402518412	-3.181071742	0.001467313
ENSG00000182903	2648.051952	1.38420429	0.435139072	3.181061823	0.001467363
ENSG00000132589	2374.41087	-1.161927685	0.365500377	-3.179005432	0.001477813
ENSG00000224431	51.30109046	1.771313598	0.557288307	3.178451037	0.001480642
ENSG00000142856	805.7211498	-1.234282896	0.38836914	-3.178117846	0.001482345
ENSG00000178202	1005.262926	-0.968861912	0.304856484	-3.178091869	0.001482478
ENSG00000198853	2215.513157	1.618640099	0.509505987	3.176881408	0.001488679

ENSG00000159479	1714.599734	1.463839184	0.46089395	3.176086782	0.001492763
ENSG00000184986	143.6920322	-1.25135166	0.394041425	-3.175685548	0.001494829
ENSG00000167699	2257.976568	-1.024729955	0.322753028	-3.174966199	0.00149854
ENSG00000162783	4685.036981	1.111437966	0.350071802	3.174885723	0.001498955
ENSG00000138798	83.17445931	1.75114909	0.551876361	3.173082258	0.001508298
ENSG00000188242	168.9947939	-1.388394034	0.43762283	-3.172581362	0.001510902
ENSG00000136152	2025.239243	0.956961645	0.301812199	3.170718907	0.001520622
ENSG00000179361	1142.562706	1.504284241	0.474654602	3.169218697	0.001528493
ENSG00000075618	8660.629397	-1.0168599	0.320903389	-3.168741548	0.001531005
ENSG00000060566	22.08727685	-2.166440892	0.683703742	-3.168683685	0.00153131
ENSG00000186448	2034.976048	1.216109109	0.383795129	3.168641335	0.001531533
ENSG00000163602	3364.205461	0.936513088	0.295564706	3.168555207	0.001531987
ENSG00000244097	21.47266276	3.151548777	0.994674728	3.168421484	0.001532691
ENSG00000188290	1037.311668	-2.01778152	0.637187077	-3.166701888	0.001541783
ENSG00000180902	521.2845578	-2.063235815	0.651800335	-3.165441476	0.001548478
ENSG00000119048	2419.38163	0.945278711	0.298669193	3.164968909	0.001550995
ENSG00000213693	85.64599089	1.85000574	0.584620712	3.164454668	0.001553739
ENSG00000135452	743.9317021	1.023552344	0.323494173	3.164051874	0.001555891
ENSG00000204394	7515.476264	-1.041531118	0.329233731	-3.163500637	0.00155884
ENSG00000101871	2805.117828	-0.866678027	0.273998495	-3.163075869	0.001561116
ENSG00000272405	4548.743079	1.113781143	0.352132445	3.162960869	0.001561733
ENSG00000178952	10035.83589	-0.879948973	0.278226397	-3.162708433	0.001563088
ENSG00000042832	21.7102255	2.526941571	0.799069877	3.162353687	0.001564994
ENSG00000164117	660.3972954	1.009040385	0.319095694	3.162187407	0.001565888
ENSG00000074219	750.3343881	-1.004511432	0.317784048	-3.160987587	0.001572352
ENSG00000248206	10.53010589	4.922263428	1.557391211	3.160582514	0.00157454
ENSG00000154263	71.05293589	2.234588911	0.707255191	3.159522812	0.001580277
ENSG00000112343	1705.74111	1.24130453	0.392999089	3.158543024	0.001585599
ENSG00000160193	439.7969574	-1.300941477	0.411887136	-3.158490189	0.001585886
ENSG00000184900	927.4340523	-1.078082002	0.341345469	-3.158331078	0.001586752
ENSG00000140474	1554.28703	-1.147308017	0.363302519	-3.157996313	0.001588576
ENSG00000141027	2801.051238	0.924785382	0.293049305	3.155733069	0.001600953
ENSG00000100209	387.124706	1.070803132	0.339327185	3.155665623	0.001601323
ENSG00000235213	6.371974073	5.135635624	1.627457058	3.155619744	0.001601575
ENSG00000104369	780.4976354	-1.893194149	0.600216042	-3.154187854	0.001609454
ENSG00000170037	1830.187643	-1.040135004	0.329768989	-3.154132251	0.001609761
ENSG00000120694	15610.35721	1.903997025	0.603657579	3.154101085	0.001609933
ENSG00000168917	287.7423146	1.480227034	0.469320443	3.153979453	0.001610604
ENSG00000113356	2700.288221	-1.2279269	0.389380342	-3.153541068	0.001613025
ENSG00000253598	135.2858582	1.45422508	0.461226807	3.152950043	0.001616295
ENSG00000077312	2324.080118	-1.156768453	0.367168261	-3.150513204	0.001629839
ENSG00000100031	1148.561081	1.893229386	0.600936195	3.150466556	0.001630099
ENSG00000137700	1298.738628	-1.882306647	0.597497991	-3.150314607	0.001630947
ENSG00000007541	315.2484426	-1.154754159	0.366667252	-3.149324498	0.001636484
ENSG00000003400	127.0657029	1.219449583	0.387272033	3.148819125	0.001639316
ENSG00000138442	2966.961661	-1.014844949	0.322364737	-3.148126428	0.001643206
ENSG00000079393	59.62299593	1.84588841	0.586352138	3.14808848	0.001643419

ENSG00000132256	2078.238861	0.996663605	0.316697162	3.147055687	0.001649235
ENSG00000077616	234.9445318	1.413593395	0.449226542	3.146727238	0.001651089
ENSG00000184162	576.1935907	-1.320728634	0.41974784	-3.146481074	0.00165248
ENSG00000203760	432.5109241	-1.342053003	0.426625879	-3.145737445	0.001656687
ENSG00000164867	322.465303	-1.350020132	0.42927772	-3.144864197	0.001661639
ENSG00000196505	1520.705843	1.316323328	0.418793337	3.143133405	0.001671496
ENSG00000187953	809.3660769	0.886078819	0.281937991	3.142814544	0.001673318
ENSG00000128563	1410.573692	1.093073515	0.347852903	3.142344092	0.001676009
ENSG00000272501	82.65733643	2.799399108	0.891172457	3.141254072	0.00168226
ENSG00000132581	2324.945448	1.588776295	0.505871297	3.140672944	0.001685602
ENSG00000163877	2119.994323	1.553510271	0.494943805	3.138760917	0.001696638
ENSG00000141076	3184.26988	-1.173045206	0.373792793	-3.13822318	0.001699754
ENSG00000263806	18.62295418	2.819087391	0.898332693	3.138132914	0.001700278
ENSG00000166900	4130.605031	1.192693864	0.380098872	3.137851631	0.00170191
ENSG00000105708	499.7194299	1.117132612	0.356097567	3.13715317	0.00170597
ENSG00000129534	1228.81378	-1.228308995	0.391687755	-3.13593923	0.001713047
ENSG00000028277	118.0704879	1.222650851	0.390164184	3.133682954	0.001726273
ENSG00000138079	22.78012605	2.960579783	0.944783564	3.133606359	0.001726723
ENSG00000116120	3306.877452	-1.193059863	0.380759931	-3.133365058	0.001728144
ENSG00000112182	405.281345	1.174288059	0.374786902	3.133215311	0.001729026
ENSG00000168993	392.6569716	-1.91096621	0.609981236	-3.132827862	0.001731309
ENSG00000050327	1300.698226	1.058729456	0.337962459	3.132683609	0.00173216
ENSG00000041982	16.88961732	2.325806367	0.742456651	3.132582038	0.00173276
ENSG00000260368	27.97306395	2.575935283	0.822588953	3.131497541	0.001739172
ENSG00000165181	56.17231457	-1.633215932	0.521711001	-3.130499316	0.001745094
ENSG00000176933	20.27218534	2.464472234	0.787372343	3.129995935	0.001748087
ENSG00000148019	2045.412836	-0.82381334	0.263243705	-3.12947024	0.001751218
ENSG00000128591	9771.536602	1.74342754	0.557105804	3.129437045	0.001751416
ENSG00000145416	229.9085428	-1.276333342	0.40792888	-3.128813393	0.001755137
ENSG00000082126	31.8924124	2.767998257	0.884859798	3.128177213	0.001758941
ENSG00000177971	1089.955758	-1.335307963	0.426895563	-3.12794997	0.001760301
ENSG00000092931	1047.36841	1.083279204	0.346456784	3.126736881	0.00176758
ENSG00000204371	2191.114084	-1.289350963	0.412417373	-3.126325534	0.001770055
ENSG00000034677	1904.486878	0.921343167	0.29472314	3.126131077	0.001771226
ENSG00000108784	292.4256708	-1.921733794	0.614850047	-3.125532482	0.001774835
ENSG00000168913	52.11163596	-2.241504341	0.717400041	-3.124483153	0.001781177
ENSG00000167528	1016.693033	1.233926761	0.394924389	3.124463301	0.001781297
ENSG00000196700	644.8406856	-1.402185082	0.448778896	-3.124445235	0.001781407
ENSG00000107771	3004.6023	0.880315087	0.281763874	3.124300766	0.001782282
ENSG00000070495	4535.725072	1.299742805	0.416183289	3.12300575	0.001790143
ENSG00000127993	1218.333682	1.437572305	0.460334184	3.122888446	0.001790856
ENSG00000150990	1679.927149	-1.306750695	0.418506995	-3.122410645	0.001793765
ENSG00000159199	2475.308045	-1.344119611	0.430480718	-3.122368915	0.00179402
ENSG00000199072	8.189385801	5.508551116	1.76451992	3.121841276	0.001797238
ENSG00000099994	217.7627312	-1.879757775	0.602132967	-3.121831683	0.001797297
ENSG00000173273	5118.270726	1.275117056	0.408465565	3.121724734	0.00179795
ENSG00000124103	27.94425995	2.650552132	0.849090985	3.121634994	0.001798498

ENSG00000263316	61.40617291	1.776717781	0.569385011	3.120415441	0.001805961
ENSG00000248159	18.72720635	3.249886371	1.041599723	3.120091431	0.001807949
ENSG00000130544	896.0322529	1.20323793	0.385782744	3.118952182	0.001814954
ENSG00000164603	610.9472803	1.295484476	0.41547717	3.118064165	0.001820432
ENSG00000040487	974.8735346	1.568881912	0.50318241	3.11791883	0.00182133
ENSG00000162994	340.1109141	1.209759061	0.388005732	3.117889659	0.00182151
ENSG00000167085	5239.187068	-0.919895932	0.29508661	-3.117376059	0.001824687
ENSG00000135048	3097.11539	1.250663683	0.401212225	3.117212305	0.0018257
ENSG00000258168	257.6937395	1.129036467	0.362337555	3.1159797	0.001833349
ENSG00000176222	7.929556859	4.015721364	1.28906076	3.115230474	0.001838012
ENSG00000168769	1446.858307	1.394703824	0.447775834	3.1147367	0.001841092
ENSG00000075188	1385.825422	-1.179548878	0.378895987	-3.113120537	0.001851204
ENSG00000105991	262.4935248	1.467718107	0.471551492	3.112529873	0.001854912
ENSG00000160606	226.799569	-1.610584099	0.517618834	-3.111525304	0.001861235
ENSG00000159753	486.3389744	-1.238601572	0.398192745	-3.110557854	0.001867343
ENSG00000172888	2642.932595	0.870768572	0.279970031	3.110220651	0.001869476
ENSG00000198431	48002.76253	1.129042772	0.36313343	3.109167809	0.001876151
ENSG00000203817	39.80648109	-2.414722289	0.776648198	-3.109158427	0.001876211
ENSG00000252690	43.9338959	1.715782662	0.551921986	3.108741278	0.001878862
ENSG00000270574	8.426330228	4.776966339	1.537850992	3.106260856	0.001894695
ENSG00000175305	324.0861785	-1.356260661	0.436629269	-3.106206473	0.001895043
ENSG00000146281	2184.053471	-1.424390993	0.458579338	-3.10609501	0.001895758
ENSG00000154265	1324.493289	1.120514682	0.360760896	3.105975995	0.001896521
ENSG00000170356	10.03216926	3.791431062	1.220691474	3.105969971	0.00189656
ENSG00000184860	1948.304871	1.593279005	0.512999578	3.105809585	0.001897589
ENSG00000110811	912.298983	-1.248555191	0.402008509	-3.105792944	0.001897695
ENSG00000132000	42.28521811	2.321063764	0.747360892	3.105679986	0.00189842
ENSG00000121552	233.8426469	1.37032415	0.441384362	3.104605119	0.001905332
ENSG00000172500	1551.892041	-1.406184833	0.452942051	-3.104557926	0.001905636
ENSG00000198315	4498.64242	1.200467547	0.386697355	3.104411062	0.001906582
ENSG00000132824	6312.478843	0.958661068	0.308885946	3.103608566	0.001911761
ENSG00000188157	4693.393018	-1.653418787	0.532802396	-3.103249536	0.001914082
ENSG00000196072	4257.345883	1.414239164	0.455787837	3.102845331	0.001916698
ENSG00000140403	8269.573413	1.874034479	0.60424558	3.101445074	0.001925786
ENSG00000131969	34.1708966	-1.736525008	0.560198713	-3.099837555	0.001936268
ENSG00000185551	2926.334304	-1.507915529	0.486527715	-3.099341483	0.001939513
ENSG00000272056	9.629807434	3.139239474	1.01288553	3.09930331	0.001939763
ENSG00000066135	3642.775994	1.148902918	0.370814175	3.098325241	0.001946177
ENSG00000110060	687.8232227	1.120441618	0.361789558	3.096942946	0.001955275
ENSG00000139722	3622.825838	0.945921575	0.305673108	3.094552804	0.001971098
ENSG00000245680	543.0916099	1.098313605	0.35498656	3.093958275	0.001975053
ENSG00000242439	10.77915463	4.390434158	1.419141326	3.093725817	0.001976601
ENSG00000128059	2533.194912	-1.047646671	0.338703259	-3.093110685	0.001980702
ENSG00000165912	1291.195022	-1.271481606	0.41115973	-3.092427378	0.001985268
ENSG00000155254	1025.371792	-1.756102824	0.567924954	-3.092138869	0.001987199
ENSG00000123374	2370.25766	-1.11132121	0.359421564	-3.091971438	0.00198832
ENSG00000188523	31.74307354	-2.024479861	0.654798928	-3.091758056	0.00198975

ENSG00000116984	1565.241544	-1.665554832	0.538734386	-3.091606694	0.001990764
ENSG00000204592	7151.964762	0.871636959	0.28198475	3.091078366	0.00199431
ENSG00000136718	2654.958738	-0.904596512	0.292656123	-3.090987822	0.001994918
ENSG00000250802	73.35947305	1.460880195	0.472669266	3.090702735	0.001996834
ENSG00000169016	2039.002471	1.082343782	0.35033839	3.089423867	0.002005451
ENSG00000136104	532.7592596	-1.253048711	0.405674582	-3.088802621	0.002009649
ENSG00000196712	2676.914252	1.128609443	0.365432578	3.088420443	0.002012236
ENSG00000240280	71.14849288	-2.031248815	0.657761942	-3.08812153	0.002014261
ENSG00000112511	3042.15407	1.329749226	0.430677961	3.087572026	0.002017989
ENSG00000198890	255.4229264	-1.068739714	0.346171724	-3.087310833	0.002019763
ENSG00000185499	75.0738465	-1.769777401	0.573278244	-3.087117677	0.002021076
ENSG00000089820	1044.682717	-1.979056151	0.641079741	-3.087067061	0.00202142
ENSG00000154316	5.426896369	5.909406504	1.914286017	3.08700291	0.002021856
ENSG00000065150	10231.45455	-1.012052108	0.327910874	-3.086363367	0.00202621
ENSG00000187187	81.55150617	1.711151098	0.554574612	3.085520074	0.002031965
ENSG00000178814	691.5184127	-1.212979039	0.393140446	-3.085358047	0.002033072
ENSG00000231721	398.0974267	1.298289219	0.420852531	3.084902963	0.002036186
ENSG00000114354	8461.127151	1.048877784	0.340007886	3.084863106	0.002036459
ENSG00000101220	1493.371142	-1.786937811	0.579307472	-3.084610329	0.00203819
ENSG00000168826	345.7101046	1.346000362	0.436461741	3.083890833	0.002043126
ENSG00000172935	15.34013698	-2.956252247	0.958613875	-3.08388218	0.002043185
ENSG00000110931	1901.91285	-1.248451249	0.404847708	-3.083755259	0.002044057
ENSG00000162924	679.6619189	1.078345277	0.349828344	3.082498301	0.002052709
ENSG00000213853	1082.650122	-1.326288655	0.43032869	-3.082036324	0.002055897
ENSG00000157741	2508.644236	0.95739185	0.310683086	3.081570553	0.002059117
ENSG00000226329	35.07274139	1.813749508	0.588584522	3.081544691	0.002059296
ENSG00000234841	6.545815673	5.1881909	1.683660663	3.081494398	0.002059644
ENSG00000022840	6550.942432	1.20882489	0.392299478	3.081382866	0.002060415
ENSG00000222881	5.684811797	-4.954463311	1.6080645	-3.081010315	0.002062995
ENSG00000167799	146.5687774	-2.382038614	0.773163153	-3.08090033	0.002063757
ENSG00000150457	2014.369943	1.354601793	0.439880652	3.079475731	0.002073653
ENSG00000125089	642.1814489	1.201316538	0.390148159	3.079129066	0.002076067
ENSG00000131828	2821.118377	-0.996007215	0.323474514	-3.079090228	0.002076338
ENSG00000198018	1046.764146	1.217894794	0.395572086	3.078818846	0.00207823
ENSG00000116353	759.7768288	-1.322963946	0.429923213	-3.077209852	0.002089481
ENSG00000239900	2363.426277	-1.202552206	0.390804936	-3.07711622	0.002090138
ENSG00000129538	398.2740272	1.364202811	0.443450396	3.076336886	0.002095609
ENSG00000163584	7534.896442	-1.045899339	0.339995287	-3.076217166	0.002096451
ENSG00000153879	4554.343268	1.003294319	0.32624863	3.075244546	0.0021033
ENSG00000169718	3112.312854	-1.198035328	0.389574155	-3.075243344	0.002103309
ENSG00000187514	59346.2578	-1.537076098	0.499860815	-3.075008185	0.002104968
ENSG00000053747	808.9840381	0.802578396	0.261061169	3.074292506	0.002110024
ENSG00000198746	1031.957631	1.893914111	0.616060584	3.074233542	0.002110442
ENSG00000239322	20.50968873	2.49764745	0.812448457	3.07422265	0.002110519
ENSG00000114933	2373.232548	1.356235521	0.441187094	3.074059825	0.002111671
ENSG00000226251	27.32349253	2.516890778	0.818780932	3.07394894	0.002112456
ENSG00000189042	359.3600099	1.142482493	0.371686728	3.073778016	0.002113666

ENSG00000267501	10.03399398	3.301451699	1.074613379	3.072222776	0.002124711
ENSG00000173991	63.19182901	1.418775282	0.461847077	3.071958996	0.002126589
ENSG00000178773	3298.625056	-2.100416015	0.683945833	-3.071026846	0.00213324
ENSG00000109814	4132.437603	0.831695409	0.270868418	3.070477592	0.002137167
ENSG00000086730	606.0430607	1.079882979	0.35173535	3.070157657	0.002139458
ENSG00000124134	20.9964382	2.662835417	0.867378477	3.069980968	0.002140724
ENSG00000259319	38.5055442	1.76075829	0.573560433	3.069874052	0.002141491
ENSG00000038210	1224.118576	-1.003550186	0.327010526	-3.068862024	0.002148758
ENSG00000164338	1175.348691	-0.906576287	0.295454381	-3.068413756	0.002151984
ENSG00000040608	587.1070523	-1.272659652	0.414791815	-3.068188923	0.002153604
ENSG00000103154	19.3570639	2.29757772	0.74921072	3.066664233	0.002164619
ENSG00000131848	312.4522278	1.084589754	0.353672817	3.066647204	0.002164742
ENSG00000127311	690.6807224	1.663430544	0.542426446	3.066647203	0.002164742
ENSG00000144815	1412.840364	1.119713939	0.365138539	3.066545488	0.002165479
ENSG00000197903	1063.151471	1.252345239	0.408414614	3.066357558	0.00216684
ENSG00000142208	4104.948848	-1.066317013	0.347779738	-3.06606998	0.002168925
ENSG00000249459	227.0738715	1.065948093	0.34772691	3.06547484	0.002173247
ENSG00000156042	514.230507	1.709493567	0.557709093	3.065206553	0.002175197
ENSG00000137801	1884.159285	1.033941726	0.337416527	3.064288926	0.002181881
ENSG00000220563	21.03164388	2.63263492	0.859251829	3.063868857	0.002184947
ENSG00000205085	34.28127895	2.275118715	0.742567204	3.063855641	0.002185043
ENSG00000181085	529.2874989	-1.62018739	0.528934307	-3.063116474	0.002190448
ENSG00000160801	14.01635	-3.284547862	1.072412046	-3.06276666	0.00219301
ENSG00000239305	940.7032274	0.983510584	0.321198828	3.06199929	0.00219864
ENSG00000271605	25.64797819	1.849171668	0.604073014	3.061172451	0.002204721
ENSG00000114450	25.09138586	2.305450409	0.753129556	3.061160448	0.002204809
ENSG00000115274	600.5904025	1.171986065	0.382891159	3.060885677	0.002206833
ENSG00000126602	2997.107056	-1.638427531	0.535299499	-3.060767913	0.002207701
ENSG00000165626	1358.008976	1.076076126	0.351620849	3.060330838	0.002210926
ENSG00000230585	13.21382768	3.313038765	1.082653149	3.060110958	0.00221255
ENSG00000138942	1556.787169	1.051034655	0.343503123	3.05975284	0.002215197
ENSG00000107021	1039.787254	1.116542741	0.364962005	3.059339675	0.002218255
ENSG00000137869	63.44044857	1.756943677	0.574358905	3.058964808	0.002221032
ENSG00000240057	9.973415968	3.638822716	1.189754748	3.058464547	0.002224744
ENSG00000248734	9.99235243	3.624979155	1.185279687	3.058332303	0.002225726
ENSG00000268573	134.8448059	1.552633153	0.507689921	3.058231196	0.002226477
ENSG00000261015	390.5235989	1.881480061	0.615349568	3.057579233	0.002231326
ENSG00000198720	674.0877556	-1.406484219	0.460031472	-3.05736521	0.00223292
ENSG00000150768	3363.910858	-0.814007577	0.26630812	-3.056638217	0.002238343
ENSG00000175065	9.16186802	3.4890255	1.141500887	3.056524563	0.002239191
ENSG00000112159	4110.650369	-1.051467263	0.344032199	-3.056304802	0.002240833
ENSG00000166747	10176.81716	1.341434594	0.438928408	3.056158067	0.00224193
ENSG00000046651	1139.848282	0.939553756	0.307445229	3.056003699	0.002243085
ENSG00000130560	1756.919227	-1.139835757	0.373019384	-3.055701137	0.002245349
ENSG00000196503	12.44655629	3.436497146	1.124903083	3.054927306	0.002251151
ENSG00000100344	220.6706191	-1.191766523	0.390118105	-3.054886478	0.002251457
ENSG00000185340	1386.580412	-1.048506837	0.343249506	-3.05464922	0.002253239

ENSG00000232630	87.37061626	2.067575389	0.676930923	3.054337332	0.002255583
ENSG00000232520	5.347559697	5.888677244	1.928433527	3.053606548	0.002261084
ENSG00000089916	5808.800934	0.989236016	0.32404724	3.052752479	0.002267529
ENSG00000083093	465.9127289	-1.282196835	0.420074683	-3.052306857	0.002270898
ENSG00000138363	3237.037483	-1.267774983	0.415431237	-3.051708371	0.002275431
ENSG00000138678	3383.095824	1.243111946	0.407498667	3.050591443	0.002283911
ENSG00000004487	7229.825932	-0.891654537	0.292345144	-3.050006316	0.002288366
ENSG00000164062	4888.67897	-1.552335229	0.509038427	-3.049544291	0.002291888
ENSG00000104419	22295.03633	1.138199697	0.373255683	3.049383433	0.002293116
ENSG00000272630	75.76418256	2.362011523	0.774770063	3.048661319	0.002298635
ENSG00000172660	8598.158544	-0.840450799	0.275684082	-3.048601106	0.002299095
ENSG00000039523	1500.648192	-0.8904584	0.292146784	-3.047982896	0.00230383
ENSG00000149499	2139.502666	-1.140727247	0.374289965	-3.047709944	0.002305924
ENSG00000170364	220.0883118	-1.083331802	0.355622304	-3.046298807	0.002316775
ENSG00000197858	1944.213974	-0.926023488	0.304023179	-3.045897655	0.002319868
ENSG00000120616	1108.042304	1.206856464	0.396379298	3.044701047	0.002329117
ENSG00000174669	1001.060864	-1.775103927	0.583016881	-3.044687015	0.002329226
ENSG00000182685	130.2102067	-1.20434873	0.395579865	-3.044514738	0.00233056
ENSG00000104518	1755.236346	-1.53667503	0.504738881	-3.044495061	0.002330713
ENSG00000256269	1329.451612	-1.04293433	0.342578038	-3.04437008	0.002331681
ENSG00000099284	1252.976601	-1.084540084	0.356289382	-3.043986542	0.002334656
ENSG00000261884	42.14324235	-1.795554036	0.5899543	-3.043547673	0.002338064
ENSG00000259442	37.97953688	1.945004023	0.639155253	3.043085408	0.002341659
ENSG00000014138	659.2710503	-1.647591564	0.541485362	-3.042725954	0.002344457
ENSG00000214814	22.97011101	2.138410775	0.70288093	3.042351392	0.002347377
ENSG00000176928	58.41873777	3.153178211	1.03648928	3.04217156	0.00234878
ENSG00000163113	2888.26444	1.237753197	0.406931087	3.041677657	0.002352637
ENSG00000109971	220527.2635	2.076757963	0.683210679	3.039703603	0.002368111
ENSG00000038219	3485.280357	1.178522791	0.387760437	3.039306437	0.002371235
ENSG00000132382	2586.253662	-1.571335083	0.517047191	-3.03905545	0.002373212
ENSG00000267404	8.27122271	3.974268998	1.30779278	3.038913396	0.002374331
ENSG00000108061	2985.537858	0.856649957	0.281951848	3.038284594	0.002379291
ENSG00000095319	5282.837333	-0.957191106	0.315139676	-3.037355114	0.002386641
ENSG00000101639	1475.752032	-1.063552699	0.350166735	-3.037275081	0.002387275
ENSG00000188215	1905.310786	1.251491631	0.412096033	3.036893174	0.002390302
ENSG00000119844	2839.148173	0.829012741	0.272982283	3.036873793	0.002390455
ENSG00000197037	963.5472008	1.221943734	0.402422528	3.036469505	0.002393663
ENSG00000100304	3369.506028	-1.609743706	0.530162719	-3.036320076	0.00239485
ENSG00000126231	55.60555156	1.56294778	0.514825806	3.035876914	0.002398372
ENSG00000164556	15.55233005	3.032583458	0.998957048	3.035749599	0.002399385
ENSG00000002746	775.3194075	1.524631646	0.502269504	3.035485203	0.00240149
ENSG00000268166	18.01631165	2.256258099	0.743500354	3.034642938	0.002408206
ENSG00000144597	3427.318277	1.279867629	0.421764864	3.034552512	0.002408928
ENSG00000174442	1333.006988	-1.244045741	0.410014925	-3.034147458	0.002412165
ENSG00000106305	523.9870273	-1.343008881	0.442739402	-3.033407181	0.002418091
ENSG00000240207	30.92658011	1.983917084	0.654307406	3.032087158	0.002428691
ENSG00000105677	1974.345034	-1.115926249	0.368045961	-3.032029606	0.002429154

ENSG00000058799	1179.32516	1.155319956	0.381058492	3.031870387	0.002430435
ENSG00000179388	410.6536889	1.450870065	0.478582514	3.031598571	0.002432625
ENSG00000218896	7.279149954	-4.524625065	1.492828877	-3.030906714	0.002438206
ENSG00000158480	912.5693286	1.354034494	0.446765427	3.030750395	0.002439468
ENSG00000118655	635.4341242	-1.079108293	0.356060944	-3.030684242	0.002440003
ENSG00000188487	18.20677945	2.198741842	0.725526386	3.030547042	0.002441112
ENSG00000162595	83.91194324	1.354255696	0.446875129	3.030501381	0.002441481
ENSG00000188996	13.46112357	3.367805266	1.111397302	3.030244233	0.002443561
ENSG00000174485	1233.384019	1.034814075	0.341565355	3.029622468	0.002448596
ENSG00000112078	3537.777369	0.936019078	0.309040053	3.028795355	0.002455309
ENSG00000115998	614.201383	1.150630114	0.379903112	3.028746215	0.002455709
ENSG00000262251	92.36384973	1.805673188	0.596180917	3.02873362	0.002455811
ENSG00000269979	93.92968546	-1.342305522	0.443267661	-3.028205395	0.002460108
ENSG00000116584	4312.98583	0.938234908	0.309862181	3.027910363	0.002462511
ENSG00000073578	4300.812895	-0.952881145	0.314719659	-3.027714086	0.002464111
ENSG00000116299	135.4229244	1.86165276	0.615160258	3.026289059	0.002475755
ENSG00000141150	14.34400539	3.940697995	1.302373929	3.025780775	0.00247992
ENSG00000198855	399.0515019	0.941673908	0.31123819	3.025573138	0.002481624
ENSG00000235513	25.77229529	2.464063847	0.814416766	3.025556385	0.002481761
ENSG00000214026	1022.296519	-0.953429713	0.315209569	-3.024748638	0.002488398
ENSG00000259630	17.16319308	2.824180291	0.934060803	3.023550804	0.002498271
ENSG00000115241	6167.667769	-0.890078525	0.294392478	-3.023441803	0.002499171
ENSG00000108384	1295.832794	-1.09811358	0.363239865	-3.02310866	0.002501924
ENSG00000270109	11.37845844	-2.762390265	0.914068401	-3.02208266	0.00251042
ENSG00000263002	153.0610677	1.533888072	0.507634135	3.021640914	0.002514086
ENSG00000135519	1193.033167	-1.897464981	0.628099632	-3.020961781	0.002519732
ENSG00000096060	2546.02946	-1.043975283	0.345663709	-3.020205057	0.002526036
ENSG00000197766	760.7691946	-1.785065394	0.591127557	-3.019763456	0.002529722
ENSG00000243601	7.873421952	4.675250592	1.549236348	3.0177775	0.002546358
ENSG00000006062	1168.593927	1.198917369	0.397391421	3.016968423	0.002553164
ENSG00000130402	17789.08726	-0.93610058	0.310286682	-3.016889331	0.00255383
ENSG00000117139	3349.472113	1.493579866	0.49510895	3.016669088	0.002555686
ENSG00000035687	1672.190927	-1.024137727	0.339517971	-3.016446296	0.002557565
ENSG00000125630	2294.508934	-1.296562376	0.429845515	-3.016345016	0.00255842
ENSG00000086065	4650.877865	1.195709516	0.396564173	3.015172824	0.002568329
ENSG00000167515	1344.521462	-0.933350028	0.309620194	-3.014499854	0.002574033
ENSG00000263648	4.25216002	5.563135374	1.846121645	3.013417556	0.002583232
ENSG00000197256	1620.349322	-1.69006969	0.560869474	-3.013303038	0.002584207
ENSG00000157870	792.3759802	-1.725723516	0.572744353	-3.013078184	0.002586123
ENSG00000139292	15.7820931	3.937785164	1.307019511	3.012797538	0.002588515
ENSG00000004866	747.2505568	1.347381421	0.447236578	3.012681624	0.002589504
ENSG00000139597	93.30304332	2.589395005	0.859779475	3.011696696	0.00259792
ENSG00000231738	20.24784513	2.59744038	0.862530849	3.011417367	0.002600312
ENSG00000228679	7.359391336	4.476919191	1.486787009	3.011136878	0.002602715
ENSG00000163874	711.684283	1.209045159	0.401573283	3.010770914	0.002605854
ENSG00000204256	19392.67412	1.234654869	0.41010227	3.01060238	0.0026073
ENSG00000145777	56.51326206	2.119183504	0.703991983	3.010238119	0.00261043

ENSG00000112787	3343.750986	-1.071107726	0.355866765	-3.009856023	0.002613716
ENSG00000011478	492.4437259	-1.130681896	0.375697103	-3.009557133	0.002616289
ENSG00000169118	2335.156378	0.975308233	0.324202251	3.008332702	0.002626854
ENSG00000165752	571.0483846	-1.335886176	0.444144039	-3.00777689	0.002631663
ENSG00000210082	342587.0409	0.936335592	0.311336208	3.007474133	0.002634285
ENSG00000112406	1677.184843	0.893589396	0.29722046	3.006486825	0.002642855
ENSG00000197324	5485.696383	0.91121366	0.30309992	3.006314424	0.002644354
ENSG00000178971	1638.840388	-0.911407791	0.30321345	-3.005829036	0.002648579
ENSG00000243696	23.93228768	-1.946106945	0.647505406	-3.005545478	0.002651049
ENSG00000100075	3232.991205	-1.095649388	0.364747808	-3.003854623	0.002665827
ENSG00000173137	414.1729965	-1.462472314	0.487017901	-3.002912854	0.00267409
ENSG00000143013	1686.145508	1.106409304	0.368477909	3.002647583	0.002676422
ENSG00000161395	198.7853331	-1.116749747	0.371948937	-3.002427583	0.002678357
ENSG00000138031	1214.232167	-1.332085873	0.443746584	-3.001906768	0.002682943
ENSG00000099821	3639.095269	-1.022133215	0.34059083	-3.00105911	0.002690423
ENSG00000260942	152.1410067	1.424075586	0.47455331	3.000875889	0.002692043
ENSG00000236165	7.732323522	5.427799566	1.808924598	3.000567063	0.002694774
ENSG00000130487	17.00078748	3.313853838	1.104496311	3.000330381	0.002696869
ENSG00000117152	50.57695626	2.321506607	0.774156626	2.998755714	0.002710846
ENSG00000071243	675.8745768	0.894738805	0.298416542	2.998288232	0.002715008
ENSG00000043355	995.2131697	-1.141288972	0.38066664	-2.998132356	0.002716397
ENSG00000169738	2789.394356	-0.987359119	0.329352493	-2.997879592	0.002718651
ENSG00000160712	1318.925774	1.752218995	0.584523187	2.997689456	0.002720347
ENSG00000147050	1928.075627	1.146646872	0.38254502	2.997416801	0.002722782
ENSG00000100324	911.6701164	-0.953220102	0.318026387	-2.997298781	0.002723836
ENSG00000183778	389.5002042	1.122244118	0.37452467	2.996449122	0.002731438
ENSG00000171617	1946.353513	-1.58404317	0.528640964	-2.996444235	0.002731482
ENSG00000147255	156.8025861	1.428848504	0.477000257	2.995487913	0.002740062
ENSG00000152223	2032.628938	1.370192529	0.457458503	2.995228029	0.002742397
ENSG00000106991	59.469067	-1.403105429	0.468454716	-2.995178364	0.002742844
ENSG00000110619	6154.673432	0.976964614	0.326238542	2.99463273	0.002747755
ENSG00000235559	10.41267503	-3.157817234	1.05463575	-2.994225478	0.002751425
ENSG00000110422	3442.18828	1.07124715	0.357814617	2.993860783	0.002754716
ENSG00000131375	1864.694753	0.839356599	0.280436574	2.993035417	0.002762177
ENSG00000140854	1038.872663	-0.80055909	0.267506796	-2.992668231	0.002765502
ENSG00000154781	1055.595826	0.987471693	0.330005026	2.992292893	0.002768905
ENSG00000085433	1369.868908	1.255528195	0.41973625	2.991231268	0.00277855
ENSG00000126457	7522.120738	-1.189477332	0.397745757	-2.990546879	0.002784784
ENSG00000214063	702.3568514	-1.130801839	0.378193622	-2.990007691	0.002789704
ENSG00000101294	6157.545424	0.894626725	0.299355088	2.988513511	0.002803381
ENSG00000253251	126.7913924	1.34564531	0.450330151	2.988130615	0.002806896
ENSG00000269947	32.95461051	-2.26659215	0.758578424	-2.987947033	0.002808582
ENSG00000224080	42.56361361	2.522804288	0.844399633	2.98768994	0.002810946
ENSG00000232611	60.19848369	-1.896015349	0.634893322	-2.986352643	0.002823269
ENSG00000164402	2041.038128	-1.085099249	0.363363721	-2.986261932	0.002824106
ENSG00000188677	655.1092636	-1.511511522	0.506159823	-2.986233702	0.002824367
ENSG00000128626	1420.413166	-0.939675552	0.314774427	-2.985234729	0.002833608

ENSG00000263006	42.68376965	2.234807551	0.748782992	2.984586422	0.00283962
ENSG00000105085	623.079545	1.258394936	0.42163505	2.984559602	0.002839869
ENSG00000260792	15.25919866	2.673978271	0.896122807	2.983941766	0.002845609
ENSG00000091010	13.92002315	3.295950794	1.104578606	2.983898816	0.002846009
ENSG00000206549	6.499798009	4.270920798	1.431381109	2.983776138	0.00284715
ENSG00000136261	2539.784377	-1.140981313	0.382557174	-2.982511872	0.002858936
ENSG00000040275	1048.445449	-1.345394835	0.451183358	-2.98192478	0.002864424
ENSG00000127585	1507.888657	-1.221445983	0.409624505	-2.981867463	0.00286496
ENSG00000197181	52.7559158	1.944314346	0.652169851	2.981300567	0.002870269
ENSG00000108798	8.213162355	3.597826553	1.20681689	2.981253066	0.002870715
ENSG00000185813	1398.891215	-1.432928651	0.480701614	-2.980910839	0.002873925
ENSG00000115875	4825.143499	-0.815778721	0.273701386	-2.980542898	0.002877379
ENSG00000175137	3352.614849	1.392938423	0.467389575	2.980251373	0.002880119
ENSG00000179431	362.877339	-2.039076589	0.684405737	-2.979338833	0.002888711
ENSG00000263293	14.37313109	2.867905363	0.96280503	2.978697942	0.00289476
ENSG00000272462	65.13641453	1.350262692	0.453325411	2.978572696	0.002895943
ENSG00000005206	1367.906171	-1.220095588	0.410033668	-2.975598549	0.002924174
ENSG00000068878	8831.693414	1.276312656	0.428955487	2.9753965	0.002926101
ENSG00000162819	2755.898098	0.897289048	0.301579834	2.975295247	0.002927067
ENSG00000234684	341.6846929	1.223691117	0.411338802	2.974898337	0.002930857
ENSG00000172738	37.7791157	1.837343655	0.617667158	2.97465007	0.002933229
ENSG00000137364	2017.911027	0.877846876	0.295156283	2.974176488	0.002937761
ENSG00000186814	1216.055197	1.004035111	0.337662218	2.973489653	0.002944343
ENSG00000183260	129.3591027	2.264027452	0.761432742	2.973378117	0.002945414
ENSG00000251059	6.570659287	4.306287616	1.448513141	2.972902002	0.002949986
ENSG00000103319	1545.465812	-1.372583161	0.46170796	-2.972838417	0.002950597
ENSG00000133112	59956.80417	1.018947277	0.342793282	2.972483214	0.002954014
ENSG00000108590	473.4546054	1.539990446	0.51814074	2.972146999	0.002957251
ENSG00000255103	559.705609	-1.590396683	0.535111926	-2.972082302	0.002957874
ENSG00000188295	486.8390493	1.410207195	0.474556813	2.971629861	0.002962236
ENSG00000075303	912.6377848	-1.216578408	0.409404163	-2.971582895	0.002962689
ENSG00000143384	16164.91946	1.135832207	0.382370297	2.970503242	0.002973123
ENSG00000260276	52.45902296	1.349470174	0.454293205	2.970482851	0.00297332
ENSG00000171282	1763.679302	-1.385241044	0.466615923	-2.968696468	0.002990659
ENSG00000123213	2855.00963	-1.094077022	0.368545638	-2.968633761	0.002991269
ENSG00000142207	2421.670481	-1.276220755	0.429909321	-2.968581262	0.00299178
ENSG00000100314	40.46410937	1.836823444	0.618808834	2.968321303	0.002994312
ENSG00000167380	798.7703268	1.202962797	0.405361937	2.967626432	0.003001088
ENSG00000154309	653.8511005	1.312511998	0.44228553	2.967567121	0.003001667
ENSG00000166478	1632.10947	0.991836081	0.334233935	2.967490661	0.003002414
ENSG00000123600	1009.809534	-1.353575877	0.456161298	-2.967318539	0.003004096
ENSG00000125945	1110.498359	1.097633329	0.370049343	2.966180995	0.00301523
ENSG00000115194	838.1725952	-0.89524785	0.301916124	-2.965220398	0.003024662
ENSG00000139618	640.0974262	-1.093755042	0.368893144	-2.964964407	0.00302718
ENSG00000188938	1335.463882	0.841425137	0.283860962	2.964215757	0.003034554
ENSG00000260190	27.67303536	2.53932514	0.856790476	2.963764435	0.003039008
ENSG00000102001	8.18898104	3.899378465	1.315713357	2.963699079	0.003039654

ENSG00000116752	2938.499058	1.382551651	0.466520783	2.963537106	0.003041254
ENSG00000267350	749.6846804	1.355116403	0.45735924	2.962914673	0.00304741
ENSG00000166579	2983.028892	1.2037602	0.406336772	2.96246927	0.003051823
ENSG00000127084	142.21557	-1.66741333	0.562856373	-2.96241352	0.003052375
ENSG00000099889	1230.584897	-1.008421322	0.340601287	-2.960709073	0.003069317
ENSG00000175518	15.2884537	2.333021826	0.788001906	2.960680435	0.003069603
ENSG00000138032	2635.212678	1.179900415	0.398551782	2.960469547	0.003071705
ENSG00000125812	1426.053737	0.762053773	0.257455409	2.959944701	0.003076943
ENSG00000149257	5312.110413	1.557330589	0.526180529	2.959688744	0.0030795
ENSG00000262202	27.61053323	2.001354483	0.676398182	2.958840718	0.003087987
ENSG00000228519	32.85225719	-2.768790903	0.935899143	-2.958428717	0.003092117
ENSG00000105552	1337.476541	-1.129017871	0.381647382	-2.958274898	0.003093661
ENSG00000165689	2339.196302	-1.616354598	0.546412636	-2.958120827	0.003095208
ENSG00000055609	4452.587534	1.103588959	0.373174007	2.957303934	0.00310342
ENSG00000187713	903.9052169	-1.098769216	0.371686227	-2.95617415	0.003114812
ENSG00000156886	34.63050119	2.136371103	0.722711564	2.956049425	0.003116072
ENSG00000184811	4.089441176	5.502169174	1.862003085	2.954973179	0.003126962
ENSG00000125450	2871.114785	-1.113078866	0.376885352	-2.953361972	0.003143331
ENSG00000129518	1360.751334	0.956080282	0.32372755	2.953348521	0.003143468
ENSG00000183598	11.694523	3.930593725	1.331072123	2.95295323	0.003147496
ENSG00000148840	4543.616026	-0.946323754	0.320532604	-2.952347878	0.003153674
ENSG00000131023	2620.82446	1.097334717	0.371822361	2.95123379	0.003165072
ENSG00000059122	3855.711833	1.053471918	0.356965489	2.95118702	0.003165552
ENSG00000100554	2384.720904	1.199143479	0.406337922	2.951099106	0.003166453
ENSG00000068308	3070.756962	1.04003514	0.352509539	2.950374459	0.00317389
ENSG00000141298	2307.418504	1.215410847	0.411957002	2.950334237	0.003174303
ENSG00000189045	77.25245737	1.672417686	0.566958381	2.949806794	0.003179727
ENSG00000158406	22.9006128	2.620118897	0.888314565	2.949539499	0.003182479
ENSG00000128607	3269.974555	0.962075126	0.326196723	2.949370911	0.003184216
ENSG00000130513	16706.89267	2.018161756	0.684338025	2.949071487	0.003187302
ENSG00000138835	2239.264013	1.204530115	0.408500411	2.948663161	0.003191516
ENSG00000131584	1181.371529	-1.408794597	0.477921919	-2.947750541	0.003200953
ENSG00000037749	2387.419648	1.100000811	0.37321942	2.947330049	0.003205309
ENSG00000031691	401.8541656	-1.270142371	0.430987549	-2.947051195	0.003208201
ENSG00000175155	792.7922812	1.277836038	0.433613978	2.946943833	0.003209315
ENSG00000166938	1081.087059	-1.011894707	0.343403357	-2.946665154	0.003212208
ENSG00000109756	3160.14208	1.132768699	0.384432844	2.94659709	0.003212915
ENSG00000263624	20.50078469	2.577402094	0.874821637	2.946202958	0.003217012
ENSG00000214694	11.87105273	2.737453924	0.929263005	2.945833322	0.003220859
ENSG00000083828	224.4217805	1.370405452	0.465469361	2.94413675	0.003238569
ENSG00000251615	122.2401771	1.412551387	0.479879205	2.943556153	0.00324465
ENSG00000165661	2688.672277	-0.987145657	0.335408656	-2.943113244	0.003249295
ENSG00000105968	4536.414425	-0.790549531	0.268647502	-2.942701959	0.003253615
ENSG00000181585	61.38929724	1.741671342	0.591881002	2.942603895	0.003254646
ENSG00000268205	1508.667297	0.925461831	0.314539134	2.942278819	0.003258064
ENSG00000166847	3606.455057	1.295575906	0.440339118	2.942223056	0.003258651
ENSG00000251442	13.22445944	3.173775581	1.078790096	2.941976935	0.003261242

ENSG00000137288	706.5358022	-1.255370879	0.426806824	-2.941309294	0.00326828
ENSG00000137393	198.4595637	1.247713226	0.424294986	2.940673978	0.00327499
ENSG00000237437	67.66050646	1.780650087	0.605708868	2.939778797	0.003284466
ENSG00000163374	2009.747274	1.126311691	0.383133036	2.939740465	0.003284873
ENSG00000171552	8092.785529	-1.170355317	0.39820617	-2.939068766	0.003292
ENSG00000232712	32.08804044	1.727717929	0.587852866	2.939031224	0.003292399
ENSG00000267510	45.32419802	1.508315196	0.513309001	2.938415639	0.003298944
ENSG00000171388	21.70637788	-2.786551545	0.948328777	-2.938381302	0.00329931
ENSG00000116761	783.2040554	0.924451132	0.314708416	2.937484625	0.003308866
ENSG00000168256	1839.957395	0.902194015	0.307177918	2.937040598	0.003313607
ENSG00000110324	189.321269	1.216567134	0.414217218	2.937026957	0.003313753
ENSG00000166592	252.5709213	1.326949663	0.451860044	2.936638635	0.003317905
ENSG00000141858	1764.69842	-1.614480338	0.55000922	-2.935369588	0.003331506
ENSG00000136603	1959.236324	1.106546957	0.377021446	2.934970859	0.00333579
ENSG00000179115	3261.914143	-0.896750043	0.3055574	-2.934800606	0.003337621
ENSG00000143393	3612.063111	1.226696211	0.417985256	2.934783449	0.003337805
ENSG00000146909	2052.438033	-0.893408357	0.304448647	-2.934512489	0.003340721
ENSG00000258472	184.9343873	-1.286046123	0.43849317	-2.932876061	0.003358379
ENSG00000176076	31.40784619	2.410545084	0.821924175	2.932807133	0.003359125
ENSG00000264083	13.080421	4.060901177	1.385360747	2.931295105	0.00337552
ENSG00000089220	6878.682406	-1.118071741	0.381439542	-2.931189922	0.003376663
ENSG00000188958	20.46362147	2.357266798	0.804385873	2.9305174	0.00338398
ENSG00000168010	412.130374	-1.923850364	0.657005528	-2.928210313	0.003409194
ENSG00000188976	7557.019028	-0.869347315	0.29689847	-2.928096313	0.003410444
ENSG00000011105	2015.603997	1.296565299	0.443032683	2.926568055	0.003427245
ENSG00000164125	42.05072072	-2.207448504	0.754303825	-2.92647131	0.003428311
ENSG00000184551	65.53453992	1.199149783	0.409781843	2.926312628	0.003430061
ENSG00000256683	126.3478547	1.505142554	0.51448513	2.925531697	0.003438681
ENSG00000050438	94.43524142	-1.528747177	0.522565976	-2.92546252	0.003439446
ENSG00000166398	2086.904778	0.914471114	0.312602003	2.9253527	0.00344066
ENSG00000095574	1813.635921	1.03163427	0.352665915	2.925245187	0.003441849
ENSG00000099904	1633.985962	-1.558287761	0.53277097	-2.924873628	0.003445961
ENSG00000147421	1004.513838	0.882646974	0.301796457	2.924643261	0.003448513
ENSG00000165006	3141.439982	0.968414791	0.331156585	2.92434104	0.003451863
ENSG00000065183	3721.34605	-1.163474856	0.398009307	-2.923235303	0.003464146
ENSG00000147804	894.3168229	-1.407908876	0.481690659	-2.922848615	0.003468451
ENSG00000124795	4152.012255	-0.982609177	0.336208703	-2.922616721	0.003471035
ENSG00000099985	11.23118539	4.252432261	1.455038231	2.922557065	0.0034717
ENSG00000173402	6291.535497	-1.098649873	0.375997499	-2.921960584	0.003478356
ENSG00000068078	1439.606317	-1.084472857	0.371172225	-2.921751098	0.003480696
ENSG00000180747	205.3308382	1.200344564	0.410894899	2.921293419	0.003485814
ENSG00000183655	572.3036418	1.257237934	0.430390291	2.921157751	0.003487332
ENSG00000177192	1360.926115	-1.123287345	0.384552451	-2.921025059	0.003488818
ENSG00000144283	2901.411762	-1.165421586	0.399117329	-2.919997461	0.003500342
ENSG00000155846	965.7722252	-1.918140416	0.656950875	-2.919762329	0.003502984
ENSG00000169174	1653.782759	-2.133963997	0.731017661	-2.919168868	0.00350966
ENSG00000186638	295.2446697	-1.284590898	0.440087287	-2.918945711	0.003512174

ENSG00000164675	47.41972752	1.949191703	0.667773722	2.918940409	0.003512234
ENSG00000108604	6374.108551	-1.005149824	0.344379202	-2.918729755	0.003514608
ENSG00000272709	4.312481532	5.598836532	1.918375152	2.918530574	0.003516854
ENSG00000196417	650.220987	1.39437242	0.477917218	2.917602395	0.003527338
ENSG00000131899	2022.838014	-1.393792441	0.477737255	-2.917487438	0.003528639
ENSG00000261597	171.1676754	1.700081579	0.582727724	2.91745443	0.003529012
ENSG00000156273	2333.881063	1.013381705	0.347411377	2.916950257	0.003534721
ENSG00000246334	54.45521385	-1.366612081	0.468590865	-2.916429197	0.003540631
ENSG00000224470	3325.151287	0.994045847	0.340969958	2.915347301	0.003552929
ENSG00000113924	50.15988855	2.301233961	0.789378717	2.915247033	0.003554071
ENSG00000177628	1348.076832	1.566643931	0.537433584	2.915046582	0.003556355
ENSG00000135521	1660.308752	-0.971805515	0.333376134	-2.915042244	0.003556404
ENSG00000103254	455.5914716	-1.625874738	0.557798638	-2.914805861	0.003559099
ENSG00000147174	82.85125745	1.719695809	0.590058017	2.914452069	0.003563135
ENSG00000266278	47.62978562	-1.775609204	0.609373323	-2.913828254	0.003570263
ENSG00000105472	117.0296115	-1.434768406	0.492400986	-2.913821148	0.003570344
ENSG00000265242	133.0391956	-1.080066124	0.370688041	-2.913679439	0.003571965
ENSG00000127423	403.1525135	-1.121036205	0.38498849	-2.911869407	0.003592728
ENSG00000130948	37.22635162	1.911255457	0.656443895	2.911529028	0.003596645
ENSG00000063587	1794.556954	1.059513658	0.363983862	2.910880859	0.003604114
ENSG00000160932	3831.582944	-1.23711108	0.425043053	-2.910554754	0.003607877
ENSG00000106348	2473.058383	-1.02940645	0.353791607	-2.909640674	0.003618445
ENSG00000145833	5960.297619	-0.993591746	0.341534741	-2.909196715	0.003623588
ENSG00000138593	3121.615512	0.988017316	0.339693028	2.908559303	0.003630983
ENSG00000228028	14.59511143	3.134040041	1.077745963	2.907958044	0.003637971
ENSG00000163481	1302.199181	1.354594141	0.465863904	2.907703579	0.003640933
ENSG00000104783	1802.406387	-1.687733387	0.580578266	-2.906986854	0.003649285
ENSG00000139977	2161.089633	1.070868961	0.368432457	2.906554347	0.003654334
ENSG00000169249	464.241493	1.238716425	0.426216216	2.906309943	0.00365719
ENSG00000166341	38.1864694	2.122326239	0.730252839	2.90628961	0.003657428
ENSG00000115970	1200.578193	-0.763212337	0.262646312	-2.905855908	0.003662501
ENSG00000134905	1626.284452	-0.818902857	0.281824934	-2.905714715	0.003664154
ENSG00000230590	177.5462877	1.58650699	0.546001922	2.905680228	0.003664557
ENSG00000099377	251.7146839	-1.755559827	0.604242461	-2.905389706	0.003667961
ENSG00000176978	3664.749465	-1.022886618	0.352073588	-2.905320511	0.003668772
ENSG00000075239	2475.398688	-1.258746209	0.433285702	-2.90511827	0.003671144
ENSG00000139767	26.28023713	2.031927636	0.69950082	2.904825239	0.003674582
ENSG00000253307	26.40933034	3.058058982	1.05286374	2.904515434	0.003678221
ENSG00000073614	3066.567069	1.037337053	0.357163638	2.904374753	0.003679874
ENSG00000160796	2377.226266	-1.680528942	0.57869591	-2.903993121	0.003684363
ENSG00000145912	1445.410419	-1.179607207	0.40624239	-2.903702904	0.003687779
ENSG00000140678	325.0948068	1.101320477	0.379291511	2.903625428	0.003688692
ENSG00000185361	926.5279293	-1.328543975	0.45754757	-2.903619343	0.003688764
ENSG00000175104	901.8491653	1.017033083	0.350272578	2.90354754	0.003689609
ENSG00000225151	443.2251638	1.10022431	0.378964678	2.903237096	0.003693269
ENSG00000152402	59.28159477	2.426678738	0.835933838	2.902955507	0.003696592
ENSG00000167747	2972.15445	-1.045158854	0.360070926	-2.902647171	0.003700233

ENSG00000151746	2288.191357	1.129202857	0.389072337	2.902295408	0.00370439
ENSG00000259617	11.10804094	2.847190591	0.981160439	2.901860367	0.003709538
ENSG00000243678	173.8778474	-1.185871066	0.408661983	-2.901838475	0.003709798
ENSG00000105877	60.91361229	-1.43714563	0.495354303	-2.901247899	0.003716797
ENSG00000244480	40.83708311	1.793183298	0.618096784	2.901136753	0.003718116
ENSG00000169105	366.6939361	-1.176649757	0.405614715	-2.900905004	0.003720867
ENSG00000143554	267.7219428	-2.008563815	0.692399167	-2.900875552	0.003721216
ENSG00000232445	293.2736054	-1.350863618	0.465752664	-2.900388391	0.003727005
ENSG00000152242	4107.024414	1.058698375	0.365022661	2.900363424	0.003727302
ENSG00000130182	15.50632459	3.614694087	1.246753056	2.899286327	0.003740132
ENSG00000142449	313.9991411	-1.716612733	0.592121123	-2.899090516	0.003742468
ENSG00000049759	3325.525666	1.043573302	0.359999153	2.898821548	0.00374568
ENSG00000182372	2007.623203	1.104701377	0.381108532	2.89865297	0.003747694
ENSG00000111725	1210.706676	1.041337795	0.359262721	2.898541187	0.003749031
ENSG00000184575	12570.8692	1.031288455	0.355863518	2.897988708	0.003755641
ENSG00000124226	4016.894379	0.818790338	0.282600107	2.897346172	0.003763342
ENSG00000177125	1433.358482	1.248799622	0.431050203	2.897109466	0.003766183
ENSG00000088836	604.0535202	-1.595650858	0.550779322	-2.897078365	0.003766557
ENSG00000135312	28.00932198	-2.347987638	0.810472763	-2.897059279	0.003766786
ENSG00000168301	872.2778907	1.083249317	0.37399427	2.896432923	0.003774313
ENSG00000225968	123.1975793	-1.782817883	0.615801546	-2.895117582	0.003790165
ENSG00000232940	36.88529262	1.79477392	0.61999555	2.894817421	0.003793791
ENSG00000108272	522.1455455	-1.507137719	0.520636521	-2.894798307	0.003794022
ENSG00000212195	9.857939711	3.234504085	1.117600853	2.894149622	0.00380187
ENSG00000100532	540.0415067	1.021582952	0.353155371	2.892729475	0.003819101
ENSG00000197919	9.917281062	4.037892445	1.396543246	2.891347946	0.003835932
ENSG00000232862	6.082643862	5.079888692	1.757082799	2.891092381	0.003839053
ENSG00000183605	1396.251507	-1.22029945	0.422149108	-2.890683477	0.003844051
ENSG00000172301	1112.057887	-0.986625425	0.341506599	-2.889037658	0.003864228
ENSG00000167371	138.0018283	-1.677757123	0.580760909	-2.888894719	0.003865985
ENSG00000143630	312.6526162	-0.95112765	0.329250894	-2.888762539	0.00386761
ENSG00000101773	1862.879863	-0.935682489	0.323997428	-2.88793184	0.003877839
ENSG00000070010	5531.164723	1.613139594	0.558584202	2.887907659	0.003878137
ENSG00000036448	167.9027653	1.200406008	0.415673681	2.887856663	0.003878766
ENSG00000177169	2646.621361	1.123168871	0.388941996	2.88775417	0.00388003
ENSG00000222937	68.66548208	1.484218046	0.514022027	2.887460004	0.00388366
ENSG00000177706	844.3540197	-1.405337094	0.48677373	-2.887043828	0.003888801
ENSG00000160471	54.01872387	-1.59609507	0.55286009	-2.886978277	0.003889611
ENSG00000173567	22.47303867	2.235199274	0.774499294	2.885992655	0.003901813
ENSG00000083817	218.935249	1.267654144	0.439265673	2.885848409	0.003903601
ENSG00000143674	1385.525925	-0.837424981	0.290220727	-2.885476136	0.003908221
ENSG00000233016	3887.606604	0.900678676	0.312152795	2.885377576	0.003909445
ENSG00000135269	9572.697451	0.932213877	0.323145234	2.884813948	0.00391645
ENSG00000182179	35.01289912	-1.696850438	0.588293752	-2.884359102	0.003922112
ENSG00000026025	117.1463599	-1.582440584	0.548656298	-2.8842111	0.003923956
ENSG00000204599	552.6429488	1.06130674	0.368146311	2.882839537	0.003941081
ENSG00000261113	22.78106121	-2.245594551	0.779193741	-2.88194634	0.00395227

ENSG00000175768	216.4429247	-1.052031911	0.365073271	-2.881700732	0.003955352
ENSG00000152409	2434.026521	0.963865916	0.334502676	2.881489407	0.003958005
ENSG00000100600	3060.500443	0.812618755	0.282039702	2.881221153	0.003961376
ENSG00000118418	1083.394126	-1.313711546	0.456048593	-2.880639403	0.003968694
ENSG00000259884	23.44559591	-3.707246063	1.287048086	-2.88042545	0.003971389
ENSG00000137185	851.9551152	1.027670924	0.356796041	2.88027558	0.003973277
ENSG00000162734	4887.776913	1.333579647	0.463016996	2.880195887	0.003974282
ENSG00000068001	938.4931555	-1.631152067	0.566483576	-2.879433994	0.003983897
ENSG00000172748	325.8230112	1.247847415	0.433460576	2.878802561	0.003991882
ENSG00000115520	2292.633461	0.911229272	0.316584578	2.878312256	0.003998092
ENSG00000196584	755.642219	-1.147112272	0.398552571	-2.87819564	0.00399957
ENSG00000103253	572.3197081	-2.070361657	0.719375547	-2.877998379	0.004002072
ENSG00000105499	105.8894977	1.620877645	0.563218402	2.877884742	0.004003514
ENSG00000136518	1649.52313	-0.910846601	0.316617701	-2.876802526	0.004017269
ENSG00000114779	1448.292593	-1.47828947	0.513986711	-2.876123911	0.004025916
ENSG00000268852	131.0916014	1.167054915	0.40577529	2.876111346	0.004026077
ENSG00000083312	14837.04765	1.203917187	0.418720569	2.875228196	0.004037356
ENSG00000126216	2040.202947	-1.02862939	0.357781626	-2.875020168	0.004040017
ENSG00000266385	9.178813697	4.143987355	1.441641694	2.874491889	0.004046782
ENSG00000224420	124.7391438	-1.677477573	0.583718037	-2.873780606	0.004055906
ENSG00000198625	6274.638917	1.152079151	0.400893341	2.873779717	0.004055917
ENSG00000163472	304.9201055	1.338027547	0.465636154	2.873547374	0.004058902
ENSG00000205189	3020.968574	0.728527101	0.253529983	2.873534299	0.00405907
ENSG00000255074	8.016836939	5.490058368	1.910591104	2.873486826	0.00405968
ENSG00000120526	2202.581813	-0.865857931	0.301364553	-2.873124671	0.004064337
ENSG00000093217	257.6730727	-1.164143242	0.405219357	-2.872871745	0.004067592
ENSG00000150527	82.33545648	1.141986581	0.397529582	2.872708429	0.004069695
ENSG00000166922	36.16157301	-1.85964956	0.647459856	-2.872223725	0.004075943
ENSG00000143952	2134.543841	1.103721317	0.384292964	2.872083071	0.004077758
ENSG00000090615	5437.662365	0.970682896	0.338069513	2.87125239	0.004088489
ENSG00000029153	894.3899288	-0.78622494	0.273980786	-2.869635321	0.004109454
ENSG00000174529	339.2823373	1.12137228	0.390784515	2.869541234	0.004110677
ENSG00000156256	3779.932801	1.14875652	0.40038615	2.869121519	0.004116136
ENSG00000166189	1103.455048	-0.796220121	0.277553586	-2.868707742	0.004121524
ENSG00000150455	448.9091766	1.492324852	0.520281718	2.868301536	0.00412682
ENSG00000176700	758.6669285	1.141046291	0.397820041	2.868247382	0.004127527
ENSG00000215548	180.5968058	1.011574959	0.352682566	2.868230688	0.004127744
ENSG00000176927	13.23900944	3.29603335	1.149164653	2.868199383	0.004128153
ENSG00000253716	233.4168696	-1.389817529	0.484585511	-2.868054239	0.004130047
ENSG00000119523	1971.841071	0.83450769	0.291003286	2.867691643	0.004134783
ENSG00000244468	12.87122754	-3.045930768	1.06220818	-2.867545955	0.004136687
ENSG00000272325	1037.107341	-0.80633358	0.28129535	-2.866501639	0.00415036
ENSG00000137996	2704.523798	1.398487706	0.488021645	2.865626394	0.004161851
ENSG00000161217	3285.340839	1.117659315	0.390054485	2.865392805	0.004164923
ENSG00000128245	4472.211512	-1.07511907	0.375304363	-2.864659131	0.004174583
ENSG00000224997	58.4642142	1.468088301	0.512500567	2.864559372	0.004175899
ENSG00000204839	1415.563168	-1.110211385	0.38761458	-2.864214716	0.004180445

ENSG00000174292	1193.166046	1.128360073	0.393973242	2.864052557	0.004182586
ENSG00000207808	10.34856746	4.242000346	1.481160893	2.863969989	0.004183676
ENSG00000120949	21.81604241	2.626252865	0.917293552	2.863045161	0.004195908
ENSG00000255224	55.1771939	1.843590631	0.64393914	2.862988932	0.004196652
ENSG00000146453	23.64181088	2.361135418	0.824869566	2.862434882	0.004203996
ENSG00000240445	150.8681903	1.357330888	0.474205521	2.862326204	0.004205438
ENSG00000162642	1590.346887	1.011982027	0.353595224	2.861978776	0.004210051
ENSG00000167565	1311.997919	0.788943541	0.275729551	2.86129484	0.004219145
ENSG00000175567	324.5013858	-1.423516226	0.497515275	-2.861251299	0.004219724
ENSG00000175868	15.60816814	2.406444876	0.84104673	2.861249904	0.004219743
ENSG00000265413	8.752202988	3.360171734	1.174416971	2.861140307	0.004221202
ENSG00000231966	26.32315091	2.68695339	0.939184707	2.860942443	0.004223837
ENSG00000171295	1019.171951	1.188361265	0.415514081	2.859978323	0.0042367
ENSG00000066777	3096.761587	0.991703445	0.346793618	2.859635804	0.004241278
ENSG00000178605	527.568908	-1.126800172	0.394092422	-2.859228215	0.004246731
ENSG00000267919	24.2039398	-1.869593152	0.653894902	-2.859164594	0.004247583
ENSG00000162971	959.7497336	0.835114513	0.292083837	2.859160309	0.004247641
ENSG00000236438	130.1221624	1.220713926	0.427007326	2.858765766	0.004252927
ENSG00000151135	3034.370398	0.841792794	0.294464024	2.858728827	0.004253422
ENSG00000135045	440.1345297	-1.421482152	0.497309467	-2.85834525	0.004258568
ENSG00000139793	3917.585829	0.709742905	0.248317869	2.858203104	0.004260476
ENSG00000073803	1387.731736	0.883704629	0.309186516	2.858160309	0.004261051
ENSG00000227693	51.65666023	-2.165156784	0.757674544	-2.857634325	0.004268119
ENSG00000141696	945.8608024	-1.254166939	0.438889227	-2.857593359	0.00426867
ENSG00000140265	1955.648462	1.004937985	0.351754653	2.856928764	0.004277618
ENSG00000199032	5.817085802	6.012317781	2.104854731	2.856405096	0.004284681
ENSG00000152240	773.2414061	-1.101050551	0.385601372	-2.855411393	0.004298111
ENSG00000272327	18.15834123	-2.221660639	0.778070481	-2.855346262	0.004298993
ENSG00000070785	511.1408772	-0.882322586	0.309013122	-2.85529165	0.004299732
ENSG00000204237	721.2179235	-1.073195119	0.376039431	-2.853943048	0.004318027
ENSG00000117697	902.574568	-0.821018046	0.287816836	-2.852571295	0.004336708
ENSG00000115956	8.154521531	4.607997601	1.615547364	2.852282578	0.00434065
ENSG00000132464	4.467122931	5.628169833	1.973672251	2.851623328	0.004349661
ENSG00000236756	27.96442392	1.930378601	0.677018086	2.851295468	0.004354149
ENSG00000100823	5175.121157	-1.217599638	0.427136326	-2.850611299	0.004363528
ENSG00000232324	64.2837169	2.374225261	0.832917668	2.850492133	0.004365163
ENSG00000170608	400.9313933	1.035254307	0.363188169	2.850462638	0.004365568
ENSG00000102683	5.961600202	-5.015791188	1.759681164	-2.850397725	0.004366459
ENSG00000169727	3589.092376	-0.762987467	0.267716235	-2.849985802	0.004372118
ENSG00000120068	488.1207881	-0.993291909	0.34863382	-2.849097969	0.004384338
ENSG00000158164	229.497819	-1.771632029	0.622199998	-2.847367462	0.004408245
ENSG00000163029	2114.861254	-1.167350258	0.409979163	-2.847340459	0.004408619
ENSG00000188807	935.980932	-0.786997524	0.276419047	-2.84711756	0.004411707
ENSG00000155465	27.55005374	1.738704902	0.610798279	2.846610675	0.004418737
ENSG00000149782	4032.178908	-1.599365908	0.561973125	-2.845982905	0.004427457
ENSG00000249476	43.33401661	1.446538526	0.508280398	2.84594592	0.004427971
ENSG00000143183	4932.096545	1.254550324	0.440872263	2.845609555	0.004432651

ENSG00000182054	1484.476348	-1.609649205	0.565668885	-2.845567868	0.004433231
ENSG00000229989	30.84224335	1.993802538	0.700672642	2.845554998	0.00443341
ENSG00000113758	6178.536629	-0.999005381	0.351147368	-2.84497471	0.004441495
ENSG00000033627	3904.561791	0.985919209	0.346583496	2.844680202	0.004445603
ENSG00000159905	112.5463458	1.303716271	0.458312962	2.844598296	0.004446746
ENSG00000234350	10.35333912	2.784410888	0.97894447	2.844299114	0.004450924
ENSG00000271020	37.94447573	2.050690114	0.721018167	2.844158731	0.004452886
ENSG00000182575	7.761557993	-3.145873722	1.106187769	-2.843887639	0.004456676
ENSG00000184840	7876.03773	1.068914823	0.375937907	2.843328124	0.004464508
ENSG00000182175	292.2999761	-1.202703385	0.423051923	-2.842921444	0.004470209
ENSG00000152147	535.0958298	-0.909301042	0.319855441	-2.842850008	0.004471211
ENSG00000051596	1074.259115	-1.014378065	0.356824196	-2.842795072	0.004471981
ENSG00000100767	360.6760556	-1.324154682	0.465841021	-2.842503393	0.004476075
ENSG00000047365	1547.447898	1.121165679	0.394500806	2.841985775	0.004483349
ENSG00000108439	1176.870397	-1.098233624	0.386469495	-2.841708431	0.004487251
ENSG00000228109	78.96479155	-1.547808301	0.544790769	-2.841105958	0.004495737
ENSG00000196652	1799.220389	1.112794511	0.391690462	2.841004872	0.004497163
ENSG00000089234	1742.10323	1.226713376	0.431796081	2.840955328	0.004497861
ENSG00000164647	651.0230883	-1.206543137	0.424906723	-2.839548239	0.004517746
ENSG00000267422	12.89047164	2.673433824	0.941544207	2.839414023	0.004519647
ENSG00000062716	16202.02407	1.272568784	0.44822445	2.839132901	0.004523631
ENSG00000109576	681.4641079	-0.953128677	0.335714912	-2.839101399	0.004524078
ENSG00000189050	800.694246	0.87296876	0.307553755	2.838426602	0.004533654
ENSG00000101546	448.8251376	-1.907152867	0.67195664	-2.838208231	0.004536758
ENSG00000105929	29.42346522	1.958833797	0.690247778	2.837870487	0.004541561
ENSG00000130829	631.8645436	-1.401572879	0.494037448	-2.836977005	0.004554289
ENSG00000171004	1169.674967	-0.931280983	0.328341003	-2.83632253	0.004563633
ENSG00000220804	84.95526663	1.239673761	0.43711241	2.836052541	0.004567493
ENSG00000239908	10.08875571	2.843037286	1.002484225	2.835992044	0.004568358
ENSG00000173457	4200.072679	-1.437211622	0.506831896	-2.835677141	0.004572865
ENSG00000138780	844.6913081	-1.055531063	0.372243989	-2.835589274	0.004574123
ENSG00000237854	366.1161745	1.412833415	0.4982658	2.835501483	0.00457538
ENSG00000178093	166.5573475	1.212408687	0.42763203	2.835168092	0.004580158
ENSG00000225697	1502.064827	-1.004750472	0.354437815	-2.834772217	0.004585837
ENSG00000102172	6554.407217	-1.056813847	0.372807186	-2.834746448	0.004586207
ENSG00000138658	708.7990988	-1.220882718	0.430719615	-2.834518503	0.00458948
ENSG00000133398	1682.272159	1.267165099	0.447056804	2.83446105	0.004590305
ENSG00000198265	6280.975532	1.052047478	0.371227391	2.833970512	0.004597357
ENSG00000124216	951.330758	1.028281142	0.362845487	2.833936697	0.004597844
ENSG00000261716	440.7378414	1.072827931	0.378602077	2.83365569	0.004601889
ENSG00000088035	582.8508223	-1.098642989	0.387913018	-2.832189014	0.004623051
ENSG00000228175	60.32847447	1.748095299	0.617266965	2.83199231	0.004625896
ENSG00000004776	13.51279561	2.688952714	0.949585872	2.831710954	0.004629968
ENSG00000179134	9330.195254	1.294505972	0.457182909	2.83148374	0.004633259
ENSG00000106803	3073.619822	0.960718613	0.339319273	2.831311654	0.004635753
ENSG00000132749	433.4084236	-1.286758381	0.454502266	-2.831137439	0.004638279
ENSG00000177917	905.2543877	-1.279546776	0.451967153	-2.831061434	0.004639381

ENSG00000100284	1031.797895	1.233057639	0.435562863	2.830952188	0.004640966
ENSG00000258240	46.37408578	-1.890487915	0.668043418	-2.829887795	0.004656433
ENSG00000176840	4.045642205	5.484480645	1.938185568	2.829698423	0.00465919
ENSG00000004975	1894.441513	-0.836487698	0.295618772	-2.829616309	0.004660386
ENSG00000122565	10309.6582	-0.861838767	0.30468355	-2.828635702	0.004674688
ENSG00000167962	1418.069554	-0.877524397	0.310308095	-2.827913325	0.004685249
ENSG00000163359	56.30050448	1.464080137	0.517738966	2.827834553	0.004686402
ENSG00000203721	13.87876619	2.87572746	1.017056986	2.827498852	0.004691318
ENSG00000100036	459.7612083	0.97606671	0.345248689	2.827140961	0.004696565
ENSG00000130726	18000.03324	-0.818814885	0.28963261	-2.827081123	0.004697443
ENSG00000158373	484.761993	1.37706319	0.487098847	2.827071339	0.004697586
ENSG00000272377	64.91248545	2.477448583	0.876634238	2.826091518	0.00471198
ENSG00000196372	648.2149817	-1.710898036	0.60542785	-2.825932165	0.004714325
ENSG00000148357	15.58944769	-3.21420055	1.137549908	-2.825546842	0.004719999
ENSG00000110876	115.4668036	1.808635701	0.640144338	2.825356087	0.00472281
ENSG00000137575	7509.348972	0.952647972	0.337190365	2.825252651	0.004724335
ENSG00000137494	702.5470811	1.134118471	0.401519544	2.824566047	0.004734469
ENSG00000168038	179.3386879	1.260306163	0.446206342	2.824491822	0.004735566
ENSG00000128011	543.0793122	-1.224723027	0.433614254	-2.824452875	0.004736141
ENSG00000168067	1481.330612	-1.169349466	0.414033196	-2.824289159	0.004738561
ENSG00000227619	11.37483047	2.81994348	0.998630281	2.823811309	0.004745631
ENSG00000125458	1313.110022	-1.188218665	0.42081418	-2.823618411	0.004748488
ENSG00000124635	40.87830169	1.527772695	0.541115039	2.823378734	0.00475204
ENSG00000144063	611.1323345	-2.210395334	0.782904553	-2.823326705	0.004752811
ENSG00000135953	860.0956049	1.026567587	0.36366164	2.822864645	0.004759666
ENSG00000253710	401.1091858	1.15098604	0.407754585	2.822742115	0.004761485
ENSG00000135723	1524.488465	-0.906734895	0.321239427	-2.822613977	0.004763389
ENSG00000100221	5870.691896	0.962740766	0.341149087	2.822052889	0.004771731
ENSG00000179141	16.33377519	3.284411538	1.163896218	2.821910999	0.004773842
ENSG00000235978	4.754926775	4.707201914	1.668126284	2.821849856	0.004774753
ENSG00000169635	955.2418032	0.919461469	0.325994905	2.820478039	0.004795216
ENSG00000142512	24.01737135	-2.777668824	0.984862368	-2.820362433	0.004796944
ENSG00000188015	72.56185016	-1.642914441	0.582539602	-2.820262238	0.004798442
ENSG00000104687	14442.45874	0.916238992	0.325016573	2.819053145	0.004816554
ENSG00000128039	371.6274211	-1.250783842	0.443757439	-2.818620563	0.004823049
ENSG00000088356	1606.831039	1.342478295	0.476333924	2.818355417	0.004827035
ENSG00000261373	522.4543924	-1.109409468	0.393665701	-2.818151204	0.004830106
ENSG00000092978	1138.716407	1.151167439	0.40849691	2.818056664	0.004831528
ENSG00000143321	9301.947464	-0.993996221	0.352748504	-2.817860915	0.004834475
ENSG00000177602	458.9530091	-1.382148905	0.490512101	-2.817767193	0.004835886
ENSG00000236671	41.28371327	-1.41758311	0.503170552	-2.817301421	0.004842905
ENSG00000167522	16886.58937	0.902607239	0.320384985	2.817258236	0.004843556
ENSG00000167136	104.9017822	-1.428558096	0.507127305	-2.816961503	0.004848034
ENSG00000137221	2632.168953	1.119564048	0.397523909	2.816343927	0.004857364
ENSG00000183527	1317.937393	-1.060404078	0.376622558	-2.815561777	0.004869203
ENSG00000177981	846.4708605	1.107599941	0.393443708	2.81514209	0.004875567
ENSG00000186866	1757.167398	1.004383203	0.356849796	2.814582535	0.004884063

ENSG00000256299	8.142028578	-3.289946239	1.169042165	-2.814223762	0.004889518
ENSG00000174514	88.34244939	-1.212405895	0.430828441	-2.814126875	0.004890992
ENSG00000080823	448.979262	1.383303281	0.49161222	2.813809797	0.004895819
ENSG00000266897	45.24996872	1.759900302	0.625487899	2.813644048	0.004898343
ENSG00000244509	2148.500681	-1.272767684	0.452356584	-2.813638023	0.004898435
ENSG00000166311	467.8004069	1.71035211	0.608069005	2.812759894	0.004911831
ENSG00000101224	5228.56421	-1.51338462	0.538233875	-2.811760261	0.004927121
ENSG00000175564	116.0711707	1.544418323	0.549275308	2.811738121	0.00492746
ENSG00000262944	1088.655072	0.783981586	0.27886233	2.811357077	0.0049333
ENSG00000156990	1298.563017	-0.958468757	0.340999912	-2.810759544	0.004942471
ENSG00000155229	3129.4177	-1.074263109	0.382201279	-2.810726098	0.004942985
ENSG00000174684	252.6317535	-0.941971606	0.335239228	-2.809848987	0.004956475
ENSG00000090971	529.7756116	-1.799135473	0.640317759	-2.809754139	0.004957936
ENSG00000164080	3422.648038	1.323626296	0.471151765	2.809341691	0.004964293
ENSG00000105204	93.44924217	-1.594230987	0.56759839	-2.808730637	0.004973724
ENSG00000164175	6.623595339	4.425093819	1.576351118	2.807175234	0.004997805
ENSG00000175356	43.69342604	2.451186724	0.873451214	2.806323564	0.005011035
ENSG00000135956	2968.464266	0.857876618	0.305703631	2.806236269	0.005012392
ENSG00000119915	311.7915813	-1.103146007	0.393270539	-2.805056308	0.005030779
ENSG00000260059	7.338247923	3.348702626	1.193934702	2.804761952	0.005035375
ENSG00000079462	790.4297021	-1.297226658	0.462559157	-2.804455686	0.005040162
ENSG00000064933	1182.874161	-1.402656337	0.500187147	-2.804263056	0.005043174
ENSG00000131931	678.4172643	0.976528349	0.348253756	2.804071262	0.005046175
ENSG00000185090	302.5285242	-0.992248637	0.353927661	-2.803535148	0.005054572
ENSG00000169981	617.1199468	1.230551306	0.438992723	2.803124613	0.005061011
ENSG00000130958	1685.494899	0.890772335	0.317911214	2.801953171	0.005079425
ENSG00000126062	1667.370782	1.310299272	0.467682138	2.801687652	0.005083607
ENSG00000185379	209.3643092	-1.034333385	0.369196966	-2.801576071	0.005085365
ENSG00000154102	468.5201579	-1.488919978	0.531489639	-2.801409228	0.005087995
ENSG00000260708	192.6215633	1.212984841	0.433084426	2.800804573	0.005097538
ENSG00000160299	2133.270514	-0.740995485	0.264599148	-2.80044547	0.005103213
ENSG00000001497	1688.478405	-1.185718438	0.423462777	-2.800053521	0.005109413
ENSG00000114378	270.3193808	-1.131099948	0.404018052	-2.799627249	0.005116165
ENSG00000110955	31437.00952	-0.863823065	0.308600786	-2.799160292	0.00512357
ENSG00000162782	34.27085678	1.799179538	0.642819075	2.798889468	0.005127869
ENSG00000167272	859.3791052	-0.901884717	0.322289352	-2.798369574	0.005136131
ENSG00000171928	3066.196833	0.801189071	0.286306088	2.798365476	0.005136196
ENSG00000229915	13.74238229	3.42662355	1.224550866	2.798269672	0.00513772
ENSG00000231625	21.15117503	2.397716342	0.856917268	2.798072148	0.005140863
ENSG00000088826	1309.878943	0.824684754	0.294751192	2.797901337	0.005143582
ENSG00000090612	482.6534646	1.205283321	0.43094862	2.796814432	0.005160917
ENSG00000143374	812.0312399	-0.893959084	0.31974185	-2.79587762	0.0051759
ENSG00000229124	18.0965627	-1.928090102	0.6897608	-2.795302518	0.005185117
ENSG00000264373	5.748210198	5.994129901	2.144360762	2.79529919	0.00518517
ENSG00000182584	21.01606168	3.508195053	1.255228773	2.79486507	0.005192138
ENSG00000182795	601.4473534	1.577379055	0.564473689	2.794424407	0.005199219
ENSG00000159363	2290.358636	-0.828504566	0.296486448	-2.794409564	0.005199458

ENSG00000174951	973.4404615	0.791199987	0.28330129	2.792786394	0.005225619
ENSG00000010256	5570.340537	-0.968630579	0.346834433	-2.792775132	0.005225801
ENSG00000156030	3437.84802	0.771569467	0.276349304	2.792007999	0.005238206
ENSG00000179403	972.7348614	-1.47869467	0.52964147	-2.791878569	0.005240302
ENSG00000197808	44.00006532	2.231720935	0.799462038	2.791528339	0.005245976
ENSG00000113657	1328.514696	-1.37181413	0.491423425	-2.791511478	0.00524625
ENSG00000148468	1694.198035	-0.815733483	0.292318532	-2.790563693	0.005261635
ENSG00000204923	175.5991977	0.968665729	0.347148091	2.790353034	0.00526506
ENSG00000119321	1895.557053	0.978102433	0.350542853	2.790250681	0.005266725
ENSG00000133193	2489.22132	1.088757967	0.390252866	2.789878211	0.005272787
ENSG00000125247	644.4393404	-1.672575767	0.5995599	-2.789672502	0.005276138
ENSG00000107020	474.9889624	-1.092026061	0.391458754	-2.789632496	0.00527679
ENSG00000137962	14216.10467	-1.238330635	0.443959795	-2.78928554	0.005282447
ENSG00000165672	8764.681859	-0.7807938	0.280000524	-2.788544064	0.005294554
ENSG00000205181	29.74830486	2.45196697	0.879324668	2.788466035	0.00529583
ENSG00000248112	245.328377	-1.707299141	0.612349899	-2.788110432	0.005301647
ENSG00000155252	2295.245762	1.162764964	0.417144589	2.787438681	0.005312651
ENSG00000142677	120.9447072	-1.016331055	0.364714441	-2.786648785	0.005325616
ENSG00000115365	3129.774399	-0.95810141	0.343850608	-2.786388592	0.005329894
ENSG00000229953	525.019474	1.156662522	0.415114347	2.786370866	0.005330185
ENSG00000262248	8.80060183	-3.272371602	1.17445747	-2.786283612	0.00533162
ENSG00000154813	1493.850084	1.152145852	0.413535265	2.786088511	0.00533483
ENSG00000086475	1928.013475	-0.832083753	0.298668019	-2.785982096	0.005336582
ENSG00000042286	3102.46168	1.129728565	0.405569356	2.785537289	0.005343909
ENSG00000166133	612.5483224	-0.834958728	0.299750605	-2.785511403	0.005344336
ENSG00000273449	35.28309121	-1.848828999	0.663966939	-2.784519665	0.005360707
ENSG00000236423	22.82756654	3.008099926	1.08051357	2.783953862	0.005370067
ENSG00000179921	11.07345922	3.111930912	1.118012518	2.783449078	0.00537843
ENSG00000105321	1726.651657	1.257479275	0.451777717	2.783402607	0.005379201
ENSG00000156384	867.0642391	-1.277577672	0.459060366	-2.783027608	0.005385422
ENSG00000114491	1307.830634	-0.850305338	0.305548395	-2.782882689	0.005387828
ENSG00000126522	675.1436524	-1.09242689	0.392573528	-2.782731923	0.005390332
ENSG00000102054	5470.455295	-0.924513955	0.332248394	-2.782598716	0.005392546
ENSG00000174405	1171.380043	0.983465524	0.353459343	2.7824007	0.005395837
ENSG00000100413	1249.098017	-1.13781793	0.40895552	-2.782253511	0.005398285
ENSG00000225329	5.758640629	5.996762348	2.155925008	2.781526411	0.005410393
ENSG00000237289	203.6718843	-1.637272154	0.588702414	-2.781154136	0.005416601
ENSG00000091157	1004.467782	1.204234117	0.43303643	2.780907181	0.005420723
ENSG00000224940	163.4613029	-1.237805405	0.445284867	-2.77980569	0.005439143
ENSG00000124787	375.6785434	-1.335393234	0.480586743	-2.778672642	0.00545815
ENSG00000179029	285.7101278	-1.686646425	0.607054483	-2.778410293	0.005462559
ENSG00000172183	575.6270994	1.319015877	0.474738615	2.778404442	0.005462658
ENSG00000234500	34.06866035	1.843731422	0.663680128	2.77804223	0.005468751
ENSG00000135338	313.7881021	1.067220096	0.38421101	2.777692642	0.005474638
ENSG00000107566	3396.758748	-0.985993661	0.355019311	-2.777295859	0.005481326
ENSG00000170779	1637.778628	-1.159792841	0.417675286	-2.776781102	0.005490014
ENSG00000180530	814.1611507	1.44025725	0.518697098	2.776682682	0.005491676

ENSG00000156709	1802.589808	-0.745385305	0.268447231	-2.776654843	0.005492147
ENSG00000116954	1305.520716	0.943534836	0.339823137	2.776546774	0.005493973
ENSG00000115839	4168.986249	1.012385305	0.364625286	2.77650877	0.005494615
ENSG00000188786	3146.292747	1.125526017	0.405384086	2.776443516	0.005495718
ENSG00000110497	1782.188544	1.125395567	0.405586464	2.774736506	0.005524645
ENSG00000204568	3012.098714	-1.018923275	0.367223759	-2.77466599	0.005525843
ENSG00000135976	248.8653486	-1.15292161	0.41554477	-2.7744823	0.005528964
ENSG00000106080	1831.101086	0.914840735	0.329740885	2.774423124	0.00552997
ENSG00000141456	2347.332511	-0.836480501	0.301600114	-2.773475409	0.005546103
ENSG00000108797	525.0441781	-1.313758949	0.473691588	-2.773447917	0.005546572
ENSG00000180573	501.8042825	1.137284251	0.410140633	2.772912898	0.005555699
ENSG00000196456	315.0893492	-1.101313851	0.39718676	-2.77278591	0.005557867
ENSG00000113763	564.2029128	-1.837397001	0.662668186	-2.772725535	0.005558898
ENSG00000117148	5.888061911	-4.989965986	1.799779817	-2.772542474	0.005562026
ENSG00000132300	3841.861056	-0.72326359	0.260939029	-2.77177237	0.005575201
ENSG00000100029	4152.869985	-0.77284689	0.278867984	-2.771371885	0.005582063
ENSG00000175505	458.4169197	0.828122756	0.298835199	2.771168723	0.005585548
ENSG00000250479	1707.255334	-1.045573059	0.377375865	-2.770641043	0.005594606
ENSG00000131871	2448.802117	0.786010035	0.283706391	2.770505212	0.00559694
ENSG00000064607	3143.44332	-0.820961531	0.296389145	-2.769877186	0.005607743
ENSG00000184363	2886.037975	-1.11415761	0.40225985	-2.769745999	0.005610002
ENSG00000164542	1196.925668	1.132210889	0.408782654	2.769713635	0.00561056
ENSG00000106477	508.1135362	-0.906076081	0.327188393	-2.769279418	0.005618043
ENSG00000168356	4.861847468	4.77174525	1.72311881	2.76924912	0.005618566
ENSG00000187123	300.7893833	-1.825136156	0.659215572	-2.768648427	0.005628934
ENSG00000083844	781.8874046	1.007810403	0.364008714	2.768643622	0.005629017
ENSG00000185624	44753.78786	0.754538029	0.27253668	2.768574227	0.005630216
ENSG00000204899	945.333225	-1.312871685	0.474263988	-2.768229757	0.005636171
ENSG00000235351	10.75229344	-3.038474142	1.097625446	-2.768224948	0.005636254
ENSG00000269069	77.38304223	1.571336597	0.567692466	2.767936321	0.005641248
ENSG00000269501	16.99537782	2.164974394	0.78216914	2.76791078	0.00564169
ENSG00000072849	1888.314761	0.730680282	0.264028272	2.76743197	0.005649984
ENSG00000071894	4871.395471	-1.018187139	0.367988712	-2.766897752	0.00565925
ENSG00000199053	29.73167046	1.697308781	0.613498651	2.766605562	0.005664324
ENSG00000065883	2400.90784	0.946182099	0.342031506	2.76635948	0.005668601
ENSG00000164190	4397.030526	1.034451449	0.373977604	2.766078602	0.005673486
ENSG00000109099	1518.609717	-0.985264421	0.356247961	-2.765670347	0.005680593
ENSG00000143476	1392.748493	-1.475843091	0.533664334	-2.765489462	0.005683744
ENSG00000167680	3706.60963	-1.096088159	0.396359224	-2.765390821	0.005685463
ENSG00000225969	19.24501929	1.943719387	0.702985912	2.764947852	0.00569319
ENSG00000115084	767.8023815	0.877758209	0.317559729	2.764072802	0.00570848
ENSG00000015532	1871.03267	-0.854575762	0.309225979	-2.763596269	0.005716823
ENSG00000031003	1175.145115	1.032782079	0.373858646	2.762493498	0.00573617
ENSG00000135926	6321.778866	1.20844622	0.437524328	2.7620092	0.005744686
ENSG00000104361	673.4446802	0.981686675	0.355455859	2.761768161	0.005748928
ENSG00000169598	525.9877708	-0.903758396	0.327264102	-2.76155677	0.005752651
ENSG00000165171	313.8930738	-1.521801766	0.551138189	-2.761198181	0.005758972

ENSG00000163964	741.4836424	-1.102377586	0.399248303	-2.761132805	0.005760125
ENSG00000260498	12.63919447	2.970532903	1.076046089	2.760600066	0.005769528
ENSG00000101190	784.8553426	-1.175850564	0.425953783	-2.760512081	0.005771082
ENSG00000166166	490.2599863	-0.879683514	0.318745911	-2.759826816	0.005783201
ENSG00000130193	540.804055	-1.647938324	0.597143349	-2.759703056	0.005785392
ENSG00000079335	464.8951851	0.882386796	0.319791606	2.759255653	0.005793319
ENSG00000162769	1299.526544	-0.939288844	0.340546217	-2.758183173	0.005812362
ENSG00000022567	2151.186502	1.191225425	0.431945263	2.757815695	0.0058189
ENSG00000139718	2415.103729	1.271799667	0.461175631	2.757733892	0.005820356
ENSG00000169188	1229.011588	-0.827448461	0.300063861	-2.757574531	0.005823194
ENSG00000135968	1916.495957	0.769136449	0.278925863	2.757494196	0.005824625
ENSG00000173261	86.1541126	1.704953618	0.618304548	2.757465756	0.005825131
ENSG00000165630	357.9385774	0.939750228	0.340907237	2.756615664	0.005840295
ENSG00000103066	557.2976015	1.193112759	0.432985369	2.755549827	0.005859358
ENSG00000183828	360.4859169	-1.251804924	0.454436256	-2.754632596	0.005875808
ENSG00000143850	945.4407878	0.977475833	0.354850064	2.754616474	0.005876097
ENSG00000269337	11.55017325	2.763108933	1.003098242	2.754574593	0.005876849
ENSG00000157657	1138.977603	-1.066701603	0.3872687	-2.754422452	0.005879582
ENSG00000135631	2371.922156	1.013619225	0.368076833	2.75382511	0.005890323
ENSG00000269693	63.52751341	1.894982438	0.688272149	2.753245849	0.005900757
ENSG00000010803	742.933646	-1.094488205	0.397571346	-2.752935333	0.005906356
ENSG00000253420	18.58932906	3.060522113	1.11198001	2.752317563	0.005917511
ENSG00000172361	245.3474756	1.209087512	0.439337901	2.752067393	0.005922033
ENSG00000003096	231.5743311	-1.038678394	0.377461361	-2.751747601	0.005927819
ENSG00000168876	657.4558536	0.908319512	0.330151185	2.751222938	0.005937322
ENSG00000121064	2549.570793	1.077479132	0.391702064	2.750761949	0.005945683
ENSG00000119231	3492.729682	1.124904471	0.40902244	2.750226787	0.005955403
ENSG00000111581	2714.567351	-1.126744981	0.409692771	-2.750219334	0.005955539
ENSG00000165030	3073.695332	1.08429949	0.394262599	2.750196168	0.00595596
ENSG00000111490	898.8753542	-0.830779859	0.302082253	-2.750177647	0.005956296
ENSG00000186432	7212.649709	1.173673433	0.426797202	2.749955785	0.005960331
ENSG00000105202	6456.274203	-0.936344714	0.340529099	-2.749676069	0.00596542
ENSG00000086289	1287.445537	1.478700918	0.537777105	2.749653908	0.005965824
ENSG00000168268	2378.278277	-1.22702169	0.446261748	-2.749556049	0.005967606
ENSG00000252759	8.310479847	3.166058156	1.151626368	2.749206032	0.005973982
ENSG00000095564	5802.503759	0.81906359	0.297931555	2.749166967	0.005974694
ENSG00000166681	1480.233377	-1.296917638	0.4718189	-2.74876152	0.005982089
ENSG00000146872	3919.822127	0.923452824	0.336062755	2.74785828	0.005998593
ENSG00000197548	975.9140619	1.034853618	0.376696836	2.747178951	0.006011033
ENSG00000129353	3058.039629	-0.808479062	0.294304937	-2.747079508	0.006012856
ENSG00000102897	1490.230981	0.961474458	0.35005145	2.746666119	0.006020439
ENSG00000158796	1377.127887	1.056866475	0.384914533	2.745717254	0.006037878
ENSG00000079156	1441.662367	0.739370026	0.269315788	2.74536458	0.006044371
ENSG00000221829	1237.398048	-1.359161416	0.495091341	-2.745274063	0.006046038
ENSG00000119820	2325.168609	0.85947536	0.313081442	2.745213369	0.006047157
ENSG00000158246	1263.78241	1.620875762	0.590483382	2.744998099	0.006051125
ENSG00000213214	374.8438226	0.974234457	0.354922009	2.744925458	0.006052464

ENSG00000137491	25.15841902	2.183375534	0.795626706	2.744221023	0.006065468
ENSG00000151876	137.7278748	-1.18224493	0.430842803	-2.744028498	0.006069027
ENSG00000138758	5545.025586	-1.1337933	0.41324342	-2.743645139	0.006076118
ENSG00000224509	5.617253357	4.050463789	1.476346852	2.743571934	0.006077473
ENSG00000127838	1110.713881	-1.309249328	0.47722305	-2.743474625	0.006079275
ENSG00000121417	605.1515793	1.098838938	0.400541992	2.743380121	0.006081025
ENSG00000246228	102.291244	-1.631894058	0.594937132	-2.742968912	0.006088645
ENSG00000019169	10.63828249	3.862691694	1.408326002	2.742753943	0.006092632
ENSG00000144824	5698.334757	-1.540807164	0.561812482	-2.742564849	0.006096141
ENSG00000168569	360.5002985	-0.948903816	0.346043248	-2.742153824	0.006103775
ENSG00000170871	3159.260115	0.772577395	0.281764859	2.741922464	0.006108076
ENSG00000106003	212.3080169	-2.044944195	0.745888607	-2.741621438	0.006113676
ENSG00000257270	72.18864371	-1.460090084	0.532652742	-2.741166932	0.006122139
ENSG00000143793	692.2399468	-1.102633856	0.402418529	-2.740017607	0.006143589
ENSG00000067704	5954.453356	-1.096703979	0.400374099	-2.73919812	0.006158925
ENSG00000267758	6.478367372	4.135867187	1.509938701	2.739096086	0.006160836
ENSG00000211452	30.56922697	2.397358166	0.875277833	2.738968219	0.006163233
ENSG00000122971	611.0982883	-1.083910573	0.39574701	-2.738897691	0.006164555
ENSG00000147874	2589.972137	-0.916390833	0.334691618	-2.73801549	0.006181116
ENSG00000152465	1049.00601	-0.861190295	0.314587702	-2.737520535	0.006190426
ENSG00000179085	282.7613573	-1.269682506	0.463877927	-2.737104811	0.006198254
ENSG00000173456	1280.564471	-1.051115229	0.384118167	-2.736437167	0.006210845
ENSG00000163655	4446.048228	-0.860550742	0.314504944	-2.736207356	0.006215185
ENSG00000072135	1074.124681	-1.28641978	0.470175238	-2.736043233	0.006218286
ENSG00000068784	817.0413527	-0.978870552	0.357830066	-2.735573796	0.006227162
ENSG00000197343	3181.207552	1.034881657	0.378359065	2.735183989	0.006234542
ENSG00000134247	4065.003263	-0.721853299	0.263948355	-2.734827797	0.006241292
ENSG00000108468	5539.134966	-0.740175492	0.270671331	-2.734591389	0.006245775
ENSG00000102898	3595.364883	-0.779947491	0.285222077	-2.734527072	0.006246996
ENSG00000196275	66.67846963	1.524865475	0.557803845	2.733694807	0.006262806
ENSG00000270810	6.840035085	4.343425939	1.588985087	2.733459222	0.006267288
ENSG00000164674	1887.37976	-1.448739167	0.530011307	-2.733411812	0.006268191
ENSG00000065491	906.478316	0.856197673	0.313313933	2.732714966	0.006281467
ENSG00000108239	974.118993	1.001696287	0.366573228	2.732595324	0.006283749
ENSG00000255650	90.88244037	-1.622161909	0.593679498	-2.732386606	0.006287731
ENSG00000052344	1377.381801	0.787207445	0.288199651	2.73146564	0.006305332
ENSG00000067208	1695.388821	0.97404818	0.356661725	2.731014043	0.006313978
ENSG00000131845	813.2641558	1.224090773	0.448221199	2.730997053	0.006314304
ENSG00000244242	87.4130058	1.684807897	0.6169271	2.730967561	0.006314869
ENSG00000197841	401.7513836	1.192370903	0.437025273	2.728379746	0.006364629
ENSG00000156860	4909.558492	1.142592558	0.418924183	2.727444736	0.006382695
ENSG00000141458	4473.515948	1.104701459	0.405032557	2.727438668	0.006382812
ENSG00000138193	599.1495126	-0.897165997	0.328997182	-2.72697168	0.006391852
ENSG00000255561	68.15867604	-2.032545847	0.745357847	-2.726939625	0.006392473
ENSG00000116095	1329.725355	0.858733965	0.314974875	2.726357031	0.006403769
ENSG00000171791	183.8627315	-1.203276905	0.441395168	-2.726076296	0.006409218
ENSG00000124181	2272.708369	-0.868644834	0.31871583	-2.725452434	0.006421343

ENSG00000157881	784.4060414	-0.817510408	0.300043693	-2.72463787	0.006437205
ENSG00000135297	1706.752094	0.929990486	0.341339701	2.724530676	0.006439295
ENSG00000165886	774.999197	0.930856072	0.341661391	2.72449887	0.006439915
ENSG00000141385	2465.390435	-0.776317485	0.284943209	-2.724463894	0.006440597
ENSG00000175920	69.30598563	-1.298029394	0.47652837	-2.723928893	0.006451039
ENSG00000113732	3039.778976	0.992814844	0.364553177	2.723374551	0.006461875
ENSG00000132003	870.0078909	1.15438901	0.423962697	2.722855145	0.006472043
ENSG00000266999	77.35270462	-1.486081781	0.545806658	-2.722725637	0.00647458
ENSG00000160293	1427.594736	-1.078467363	0.39617807	-2.722178343	0.006485313
ENSG00000175106	172.189679	1.060089389	0.389438364	2.722097992	0.00648689
ENSG00000135632	1286.404547	-0.827191574	0.303936226	-2.721595856	0.006496754
ENSG00000213585	9302.577362	-0.813943194	0.29908195	-2.721472141	0.006499186
ENSG00000244607	128.0437181	1.489016533	0.547148668	2.721411238	0.006500384
ENSG00000206989	32.04229684	1.534626981	0.564055289	2.720703113	0.006514324
ENSG00000171483	16.83506552	4.002545107	1.471264765	2.720479142	0.006518738
ENSG00000099624	2972.030943	-1.155852305	0.424881965	-2.720408017	0.006520141
ENSG00000266304	17.82966066	1.999843901	0.735169624	2.720248276	0.006523292
ENSG00000133606	5214.223199	0.829524761	0.304949449	2.720204164	0.006524162
ENSG00000229020	26.45717591	2.091395453	0.768980765	2.719698007	0.006534156
ENSG00000039139	27.07595419	-2.37305502	0.872571735	-2.719610233	0.006535891
ENSG00000251467	6.735577928	4.348686063	1.599181385	2.719320087	0.006541627
ENSG00000130545	758.9115772	0.771151382	0.283603283	2.719120084	0.006545584
ENSG00000256001	234.8534735	1.371732433	0.504563523	2.718651609	0.006554861
ENSG00000099875	7431.433213	0.74976774	0.275821521	2.718307614	0.00656168
ENSG00000076685	2581.194315	0.798617331	0.293820281	2.718046989	0.006566851
ENSG00000172869	2098.280217	0.903766857	0.332511471	2.718002042	0.006567743
ENSG00000061337	283.5818566	-1.687567338	0.621054043	-2.717263269	0.006582423
ENSG00000113119	379.5931906	-1.567931499	0.577073504	-2.717039491	0.006586875
ENSG00000169696	996.6058607	-0.720911078	0.265334714	-2.716987415	0.006587911
ENSG00000171988	6127.985018	0.865049793	0.318432472	2.71658788	0.006595868
ENSG00000013375	2713.650972	0.685452078	0.252361198	2.716154794	0.006604503
ENSG00000238273	35.13655654	1.684508855	0.620285943	2.71569729	0.006613636
ENSG00000159720	3051.484008	1.360965921	0.501153436	2.715667144	0.006614238
ENSG00000167842	1341.946825	0.816278122	0.300586974	2.715613762	0.006615304
ENSG00000141699	3485.485866	1.06249072	0.391286452	2.715378246	0.006620011
ENSG00000183864	4583.418206	0.836112877	0.307929756	2.715271457	0.006622147
ENSG00000235052	6.995691264	-3.315724331	1.221186385	-2.715166473	0.006624246
ENSG00000197620	288.8196194	0.94013352	0.346288793	2.71488289	0.006629921
ENSG00000176884	293.3019338	-1.585638667	0.58431168	-2.713686413	0.006653912
ENSG00000163485	120.7611905	-1.633826087	0.602182942	-2.713172317	0.006664244
ENSG00000175054	1422.572338	-0.863687116	0.318380366	-2.712752446	0.006672693
ENSG00000168894	1715.855767	1.063801222	0.392219744	2.712258211	0.006682651
ENSG00000182459	653.0111195	1.049164249	0.386906979	2.711670521	0.00669451
ENSG00000213918	799.8795045	1.241505686	0.457916664	2.711204428	0.006703928
ENSG00000189238	23.80692525	1.887832583	0.696315157	2.711175484	0.006704513
ENSG00000247853	97.23626385	1.484880113	0.547919846	2.710031627	0.006727679
ENSG00000224660	264.88352	0.896651196	0.330886262	2.709847156	0.006731422

ENSG00000180822	466.6485005	-0.907565891	0.334990758	-2.709226658	0.006744025
ENSG00000245025	19.1140342	2.535388337	0.935943138	2.70891279	0.006750408
ENSG00000176239	48.42062351	-2.461347509	0.908962686	-2.707864191	0.006771773
ENSG00000270228	34.00579488	1.558636934	0.57578514	2.706976659	0.006789903
ENSG00000166925	1711.810936	1.046780066	0.386711189	2.706878148	0.006791918
ENSG00000117395	2775.254271	-0.976062284	0.360752153	-2.705631211	0.006817471
ENSG00000243646	852.1406334	1.013515255	0.374652067	2.705217305	0.006825972
ENSG00000066735	656.4329753	-1.342991874	0.496604807	-2.704347308	0.006843872
ENSG00000130529	1303.256691	-0.819627351	0.303102005	-2.704130417	0.00684834
ENSG00000058866	885.657361	1.253535028	0.463600569	2.703911754	0.006852849
ENSG00000163170	659.7263905	-1.044856599	0.386427204	-2.703889863	0.0068533
ENSG00000110395	6274.680684	0.824777109	0.30507467	2.703525366	0.006860821
ENSG00000105135	1267.635044	-1.217511807	0.450382084	-2.7032865	0.006865754
ENSG00000173480	342.3076489	1.339570623	0.495539731	2.703255742	0.006866389
ENSG00000154237	615.7891334	-1.168253918	0.432283812	-2.702515997	0.006881687
ENSG00000159871	127.9180475	-1.772297018	0.655881519	-2.702160325	0.006889053
ENSG00000104983	103.0062044	-1.849760514	0.684754127	-2.701349932	0.006905864
ENSG00000062194	6241.837162	0.994653072	0.368226148	2.701201634	0.006908944
ENSG00000183421	765.2191052	0.734441879	0.271898296	2.701163965	0.006909726
ENSG00000105483	961.086075	1.083851354	0.401297503	2.700867424	0.00691589
ENSG00000197283	662.0949033	-1.462301025	0.541438562	-2.70077	0.006917916
ENSG00000151292	2812.631918	0.721647635	0.267258143	2.700189517	0.006929999
ENSG00000233429	220.0660327	1.078643318	0.399477996	2.700131992	0.006931197
ENSG00000269974	14.89128458	-2.408194224	0.891952125	-2.699914216	0.006935736
ENSG00000273237	30.88884206	1.760214259	0.652053762	2.69949253	0.006944531
ENSG00000165591	199.7482843	0.97896315	0.362650358	2.699468317	0.006945037
ENSG00000117477	18.59791918	2.252727603	0.834587876	2.699209597	0.006950439
ENSG00000174776	5.544351149	4.94361899	1.83175534	2.698842406	0.006958112
ENSG00000205090	12.35107783	-2.286356374	0.847193406	-2.698741938	0.006960213
ENSG00000197409	19.93086412	2.577486602	0.95544091	2.697693363	0.006982172
ENSG00000105656	1526.02904	1.151998144	0.427099517	2.697259297	0.006991281
ENSG00000225032	79.61483202	-1.253553226	0.464753926	-2.697240745	0.00699167
ENSG00000237667	3.730543036	5.369946125	1.991038757	2.697057557	0.006995518
ENSG00000260034	26.31903868	2.00570015	0.743677181	2.697003754	0.006996648
ENSG00000110328	770.9438937	-1.645537628	0.610297151	-2.696289216	0.007011676
ENSG00000260105	58.324963	1.685174979	0.625055548	2.696040349	0.007016917
ENSG00000239670	8.655528288	3.49502992	1.296407652	2.69593435	0.00701915
ENSG00000254469	36.66035047	-1.770647977	0.656807737	-2.695839099	0.007021157
ENSG00000127445	1246.238797	-0.898634154	0.333355452	-2.695723583	0.007023592
ENSG00000141522	7059.043613	-0.819950776	0.304257942	-2.69491988	0.007040556
ENSG00000175866	4292.514327	1.075144089	0.398963245	2.694844957	0.007042139
ENSG00000100441	3312.785044	0.706943353	0.262434435	2.693790366	0.007064458
ENSG00000136938	5334.651878	-1.131654563	0.420118097	-2.693658214	0.007067259
ENSG00000153187	19885.61744	-0.686082611	0.254760001	-2.693054669	0.007080066
ENSG00000183751	1727.857934	-0.932302648	0.346196089	-2.692990122	0.007081437
ENSG00000162337	3102.144677	-1.105878682	0.410700936	-2.692661706	0.007088415
ENSG00000266972	38.24739829	-1.70875068	0.634618887	-2.692561969	0.007090536

ENSG00000198055	2293.026129	-0.875528955	0.325226585	-2.692058381	0.007101251
ENSG00000151835	4969.666145	-0.869687448	0.323058325	-2.692044691	0.007101543
ENSG00000135090	1190.345628	0.749624618	0.278494588	2.691702637	0.00710883
ENSG00000157168	109.2400177	-1.578308896	0.586442442	-2.691327883	0.007116821
ENSG00000149926	21.50451749	-2.46714265	0.916712988	-2.691292347	0.007117579
ENSG00000173473	6669.58107	-0.839964241	0.312123992	-2.691123597	0.007121181
ENSG00000204514	447.9406854	0.995938836	0.370208587	2.690209979	0.007140707
ENSG00000242337	37.35612535	1.945029904	0.723136164	2.689714609	0.007151315
ENSG00000170242	2779.492817	1.047298471	0.389372785	2.689706398	0.007151491
ENSG00000200152	12.73386233	2.665443862	0.991207616	2.68908735	0.007164767
ENSG00000135047	4296.258625	1.080890912	0.401957424	2.689068161	0.007165179
ENSG00000134363	22.88753105	1.723988192	0.641166655	2.688830086	0.007170291
ENSG00000260038	5.898781606	3.468692902	1.29024443	2.688399827	0.007179537
ENSG00000112514	1844.566344	-0.97369152	0.362196533	-2.688296083	0.007181769
ENSG00000114993	1963.785683	-0.851278321	0.316673462	-2.688189642	0.007184058
ENSG00000153140	1027.196303	-1.070752309	0.398327962	-2.688117359	0.007185614
ENSG00000166897	1899.229943	-1.599458693	0.595068566	-2.687856131	0.007191237
ENSG00000135372	3268.99797	-0.745289199	0.277282724	-2.687831354	0.007191771
ENSG00000271424	6.799656244	5.236768294	1.948426245	2.687691314	0.007194787
ENSG00000102100	1682.392848	0.899812111	0.334861614	2.687116328	0.007207185
ENSG00000112379	1937.451528	0.787951059	0.293257553	2.686890925	0.00721205
ENSG00000139990	3009.393357	0.926635461	0.344896024	2.686709609	0.007215966
ENSG00000156265	21.87385476	2.906714471	1.082318807	2.685636111	0.007239188
ENSG00000250934	23.86566894	2.385490747	0.888452174	2.684996241	0.007253062
ENSG00000075975	2908.234055	1.097782955	0.40890364	2.68469842	0.007259528
ENSG00000186019	3.344569374	5.212491614	1.941720826	2.684470159	0.007264487
ENSG00000049089	167.8132024	-1.283800791	0.478320044	-2.683978661	0.007275175
ENSG00000187630	359.5460456	-0.98609399	0.367452026	-2.683599277	0.007283435
ENSG00000027869	59.06200553	-2.401892736	0.895049412	-2.68353088	0.007284925
ENSG00000165449	438.6421272	-0.967148548	0.360449275	-2.683175177	0.007292678
ENSG00000172493	3861.086647	1.018734946	0.379751738	2.68263406	0.007304487
ENSG00000103404	852.0708566	-0.977890163	0.364553262	-2.682434268	0.007308851
ENSG00000234492	37.46855902	1.756009861	0.65480857	2.681714844	0.007324587
ENSG00000238120	22.08183607	1.771804537	0.660708808	2.681672341	0.007325517
ENSG00000134146	342.6011611	-1.288449007	0.480596	-2.68093993	0.007341569
ENSG00000119227	59.23944227	-1.34900874	0.503210739	-2.680802765	0.007344579
ENSG00000213453	23.42574596	2.258883991	0.842617769	2.680793206	0.007344789
ENSG00000258428	3.387221794	-5.198513764	1.939606217	-2.680190298	0.007358032
ENSG00000117500	3490.364362	0.969595891	0.361794283	2.679964655	0.007362993
ENSG00000166199	1028.04161	1.034144352	0.385886743	2.679916766	0.007364047
ENSG00000164744	747.9234338	1.541219506	0.575145996	2.679701356	0.007368787
ENSG00000110906	3561.569348	1.086113841	0.405336091	2.679538941	0.007372363
ENSG00000187145	2017.730514	-0.843563644	0.314819587	-2.679514489	0.007372901
ENSG00000135069	11575.64128	1.094128739	0.408353844	2.679364366	0.007376208
ENSG00000151240	3040.57793	1.181565032	0.44099757	2.679300551	0.007377614
ENSG00000137563	1286.531724	-1.02126162	0.381185561	-2.679171838	0.007380451
ENSG00000227959	60.56676583	2.327855159	0.868907576	2.679059571	0.007382926

ENSG00000251669	92.5976008	-1.550708271	0.578831515	-2.679032205	0.007383529
ENSG00000175691	422.5105555	0.859884675	0.320977912	2.678952794	0.00738528
ENSG00000185267	63.14399787	1.325013847	0.494606711	2.678924116	0.007385913
ENSG00000174792	19.02952723	1.957162739	0.73065262	2.678650133	0.007391959
ENSG00000125454	439.4605358	-0.965633907	0.360507027	-2.678543928	0.007394303
ENSG00000148358	5635.51594	0.79253734	0.295902182	2.678376129	0.007398009
ENSG00000231551	6.985430068	4.23681935	1.581893811	2.678320961	0.007399228
ENSG00000232709	47.46220833	2.304948074	0.860629863	2.678210662	0.007401665
ENSG00000188321	643.5371773	1.269335524	0.47411617	2.67726689	0.007422548
ENSG00000145016	2189.594278	1.032906185	0.385875322	2.67678736	0.007433179
ENSG00000136877	1555.262435	-0.843846486	0.31528008	-2.676497939	0.007439602
ENSG00000184507	12.03489035	-2.602784461	0.972545457	-2.676259956	0.007444888
ENSG00000171475	4254.484584	0.839052615	0.31353905	2.676070541	0.007449096
ENSG00000161513	2834.243551	-1.138530522	0.425461771	-2.675987829	0.007450935
ENSG00000180044	52.17464718	-1.892782281	0.707371222	-2.675797689	0.007455163
ENSG00000164167	855.9148858	-0.903327939	0.337748286	-2.674559651	0.007482746
ENSG00000156381	500.8824501	-1.096626099	0.410032203	-2.674487737	0.007484351
ENSG00000247077	2808.574314	-0.776622199	0.290448016	-2.673876758	0.007498
ENSG00000268759	4.605407935	4.669145733	1.74626341	2.673792343	0.007499888
ENSG00000225724	4.334107737	4.585223548	1.715308353	2.673119115	0.007514956
ENSG00000110108	1158.913379	-0.892543886	0.333925653	-2.672882055	0.007520269
ENSG00000213672	1056.018388	-1.299518342	0.486273583	-2.672401683	0.007531044
ENSG00000104643	1407.385458	0.917080782	0.343244352	2.671801519	0.007544526
ENSG00000175895	1380.307682	0.786518496	0.294433642	2.671292897	0.007555968
ENSG00000204103	63.29482831	1.531807902	0.573466068	2.671139562	0.007559421
ENSG00000189164	136.414487	1.200143841	0.449319893	2.671023159	0.007562043
ENSG00000160055	247.0795817	1.511122566	0.565834399	2.670609228	0.007571373
ENSG00000198467	6362.139152	-0.719151884	0.269303202	-2.670417134	0.007575707
ENSG00000150456	177.287524	-0.979195583	0.366768006	-2.669795531	0.007589745
ENSG00000196597	190.7094356	1.255324715	0.470270261	2.669368702	0.007599398
ENSG00000160803	3141.241629	-0.811235008	0.30397509	-2.668754893	0.007613299
ENSG00000144559	356.637147	-1.352279867	0.506772547	-2.668415793	0.007620988
ENSG00000167106	1966.047753	0.821900364	0.308055503	2.668026886	0.007629815
ENSG00000196227	1159.565206	1.226072322	0.459554515	2.667958387	0.007631371
ENSG00000137168	1740.257129	-0.806644153	0.302429212	-2.667216395	0.007648241
ENSG00000116747	4303.745228	0.824904087	0.309281262	2.667164769	0.007649416
ENSG00000113318	204.8000784	0.93770897	0.351588398	2.6670646	0.007651696
ENSG00000166435	736.7335002	0.981252782	0.367923761	2.667000302	0.00765316
ENSG00000232112	704.1586167	-1.001523572	0.375651918	-2.666094659	0.007673808
ENSG00000104450	1137.975929	1.110504081	0.416529274	2.666088918	0.007673939
ENSG00000165121	139.2788558	1.007369138	0.377960992	2.665272763	0.00769259
ENSG00000255753	13.59644758	-2.522042576	0.946284227	-2.665206187	0.007694113
ENSG00000273329	163.955587	1.50052409	0.563033748	2.665069538	0.00769724
ENSG00000119285	3235.242601	-0.922514142	0.346171302	-2.664906466	0.007700974
ENSG00000165805	6.378787673	4.265585451	1.6007055	2.664815889	0.007703048
ENSG00000198837	1711.717751	-1.137882725	0.427050898	-2.664513127	0.007709986
ENSG00000232559	426.5765086	1.007844091	0.378265045	2.664386005	0.0077129

ENSG00000177738	65.54488175	1.728049918	0.648635794	2.664129752	0.007718778
ENSG00000129472	1852.242616	0.929733898	0.348982722	2.664125874	0.007718867
ENSG00000106399	780.7538294	-1.04802086	0.393498161	-2.663343734	0.007736834
ENSG00000207652	52.90909538	-1.679112376	0.630477613	-2.663238697	0.007739249
ENSG00000135870	2606.592002	1.011327559	0.37979293	2.662839354	0.00774844
ENSG00000196646	225.2669167	1.476103723	0.554338337	2.662820923	0.007748864
ENSG00000167034	681.6604367	1.074802654	0.403763291	2.661962291	0.007768658
ENSG00000172350	211.7839583	-1.462589991	0.549572565	-2.661322787	0.00778343
ENSG00000270679	4.919894949	5.769536356	2.168259862	2.660906314	0.007793064
ENSG00000214425	279.5724319	-0.882640703	0.331736287	-2.660669749	0.007798541
ENSG00000182851	20.57721005	2.531329607	0.951414396	2.660596284	0.007800242
ENSG00000146729	2363.923824	-1.446980664	0.543897267	-2.660393334	0.007804944
ENSG00000189134	4.93032538	5.772478605	2.169836072	2.660329358	0.007806427
ENSG00000165724	1324.567095	-0.872158995	0.32789224	-2.659895195	0.007816497
ENSG00000238755	4.909464518	5.766602111	2.168045161	2.659816417	0.007818325
ENSG00000196689	24.75581195	-1.817212791	0.68321387	-2.65980079	0.007818688
ENSG00000235437	627.3672332	1.20577981	0.453338037	2.659780808	0.007819152
ENSG00000116213	1696.70704	-0.771753938	0.290200826	-2.659378851	0.007828488
ENSG00000130703	2624.724865	0.939125677	0.353274562	2.658345034	0.007852545
ENSG00000226390	13.56658506	-2.153095833	0.809939817	-2.658340519	0.007852651
ENSG00000115159	3665.750414	-0.890506269	0.334988339	-2.658320198	0.007853124
ENSG00000169126	274.9814092	-1.739405561	0.654354796	-2.658199455	0.007855938
ENSG00000103375	5.350310375	4.890904527	1.839966716	2.65814837	0.007857129
ENSG00000110330	3408.014394	0.728463307	0.274075438	2.657893434	0.007863075
ENSG00000267453	171.330856	1.053472237	0.396395412	2.657629746	0.007869229
ENSG00000188986	2786.465356	-0.803612056	0.302381381	-2.657610905	0.007869669
ENSG00000170128	3.323708512	5.204499255	1.958831136	2.656941254	0.007885318
ENSG00000132661	499.3258676	-1.076708864	0.405253848	-2.656875113	0.007886865
ENSG00000175224	3681.903648	0.833273119	0.313680746	2.656436932	0.007897122
ENSG00000103148	820.2795195	-0.999043495	0.376097036	-2.656345035	0.007899275
ENSG00000075914	1107.318545	-0.965300974	0.363395914	-2.656334147	0.00789953
ENSG00000213076	4.974736506	4.785507632	1.802059908	2.655576328	0.007917301
ENSG00000145506	746.6960374	-0.955941005	0.360044495	-2.655063525	0.007929347
ENSG00000172757	20292.76277	-0.723617299	0.272552989	-2.654960061	0.00793178
ENSG00000182318	479.2377043	1.028357833	0.387369532	2.654720484	0.007937415
ENSG00000113595	1010.738904	0.880434629	0.331831197	2.653260564	0.007971831
ENSG00000259230	74.04813584	2.07330203	0.781423349	2.653237879	0.007972366
ENSG00000249850	9.796883493	3.482999673	1.312895834	2.65291395	0.007980021
ENSG00000133134	1211.972716	0.696477214	0.262617904	2.65205534	0.008000343
ENSG00000006534	619.3165729	-1.133179113	0.42728694	-2.65203311	0.00800087
ENSG00000121350	645.4430934	1.053332744	0.397261767	2.651482806	0.008013919
ENSG00000170044	11.27761034	2.765868089	1.043170851	2.651404692	0.008015773
ENSG00000215251	1965.833287	1.270423554	0.479162797	2.651340132	0.008017306
ENSG00000155189	3234.46291	-1.39791254	0.527263149	-2.651261597	0.00801917
ENSG00000142875	8806.434374	-0.861886233	0.325115158	-2.651018299	0.008024949
ENSG00000154548	4.437706505	4.603682107	1.736911711	2.650498629	0.008037305
ENSG00000204301	15.05299431	-1.933400956	0.729496906	-2.650320981	0.008041533

ENSG00000114859	668.724455	-0.72259514	0.272713355	-2.649650727	0.008057502
ENSG00000124713	46.0592746	1.653152905	0.623923457	2.649608514	0.008058509
ENSG00000224618	3.333481176	-5.178286496	1.954385009	-2.649573382	0.008059347
ENSG00000227799	74.43693112	1.386591599	0.523332512	2.649542246	0.008060089
ENSG00000100105	1102.592783	-0.95594328	0.360852983	-2.64912118	0.008070139
ENSG00000023330	6106.456459	0.931271386	0.351722374	2.64774565	0.008103047
ENSG00000139437	608.4673222	-0.749455539	0.283075988	-2.6475419	0.008107931
ENSG00000251576	11.68942114	2.809497817	1.061356353	2.647082489	0.008118955
ENSG00000243479	83.95071751	-1.669889504	0.630933713	-2.646695633	0.008128247
ENSG00000017483	828.5236319	-0.982746453	0.371417936	-2.645931598	0.008146629
ENSG00000264918	22.34952066	1.650119113	0.623794846	2.645291355	0.008162061
ENSG00000115145	1700.367428	0.872034844	0.329692705	2.644992837	0.008169265
ENSG00000117614	2317.925749	0.802923079	0.303570009	2.644935449	0.00817065
ENSG00000096092	1057.951961	-1.027980954	0.388685654	-2.644761761	0.008174845
ENSG00000183718	474.1984467	1.004936995	0.379981673	2.644698589	0.008176371
ENSG00000158106	1083.14366	-1.452910947	0.549373232	-2.644670074	0.00817706
ENSG00000054523	7315.412592	1.143480935	0.432515675	2.643790738	0.008198332
ENSG00000132359	3071.221584	-1.409103101	0.533293288	-2.64226671	0.008235318
ENSG00000250889	4.951167346	4.756935773	1.800565678	2.641911834	0.008243951
ENSG00000183475	1544.406753	0.920644379	0.348533487	2.641480411	0.008254458
ENSG00000168936	706.3775701	-1.390574626	0.526480254	-2.641266439	0.008259674
ENSG00000181924	1515.958906	-0.858283512	0.324975619	-2.64107047	0.008264453
ENSG00000149289	1423.594443	0.780161478	0.29540408	2.640997638	0.00826623
ENSG00000170091	30.19073144	2.909965397	1.102020793	2.640572133	0.008276617
ENSG00000234698	3.204758002	5.15554256	1.95273307	2.640167588	0.008286504
ENSG00000116455	731.4589094	-0.976864718	0.370017923	-2.640047032	0.008289452
ENSG00000221838	386.5666165	-0.753073194	0.28529629	-2.639617899	0.008299955
ENSG00000178445	276.3613938	-1.218391937	0.461627701	-2.639338877	0.00830679
ENSG00000178852	141.8453089	1.577240027	0.59766085	2.639021825	0.008314563
ENSG00000184432	11873.39165	0.790014309	0.299364513	2.638971133	0.008315806
ENSG00000224621	36.06969244	2.596989188	0.984299308	2.638414115	0.00832948
ENSG00000167614	49.64075014	1.506546482	0.571171077	2.637644908	0.008348395
ENSG00000172667	2516.930265	0.714576941	0.270939984	2.63739936	0.008354441
ENSG00000157540	3309.268758	0.872013982	0.330724969	2.636674168	0.008372321
ENSG00000101444	10062.8024	-0.964947546	0.366004627	-2.636435373	0.008378216
ENSG00000271270	38.76837652	-1.401708485	0.53190541	-2.635258934	0.008407312
ENSG00000053371	1464.427633	-1.234556727	0.46847753	-2.635252807	0.008407464
ENSG00000206630	29.18518113	-1.869177285	0.709382182	-2.634936897	0.008415293
ENSG00000232716	15.39413826	2.814955312	1.068339485	2.634888397	0.008416495
ENSG00000113407	13352.40489	0.735932972	0.279358448	2.634368061	0.008429406
ENSG00000128335	1054.295799	1.41807466	0.538316366	2.634277445	0.008431656
ENSG00000180739	169.4657432	-1.350605964	0.512722187	-2.634186694	0.00843391
ENSG00000148803	127.1997382	-1.196081701	0.454061203	-2.634186079	0.008433925
ENSG00000163382	2198.798492	-0.922860208	0.350364808	-2.63399801	0.008438598
ENSG00000166848	3449.516902	1.159867206	0.440382307	2.633773402	0.008444182
ENSG00000007923	1912.372422	-0.691929578	0.262761866	-2.63329527	0.00845608
ENSG00000064687	3047.70851	-1.056752033	0.401312055	-2.633242683	0.008457389

ENSG00000167232	577.801629	1.082701938	0.411253845	2.632685267	0.008471281
ENSG00000170919	974.6886083	0.815484964	0.30976328	2.632606953	0.008473234
ENSG00000237190	1135.574794	-1.117647522	0.424567969	-2.632434857	0.008477528
ENSG00000047230	624.3187539	-0.772991846	0.293830387	-2.63074168	0.008519877
ENSG00000247796	99.65306868	-1.173158938	0.446010442	-2.630339624	0.008529961
ENSG00000149657	1883.398832	-0.841606837	0.319984141	-2.630151714	0.008534677
ENSG00000186891	731.106949	-1.104210368	0.420009905	-2.629010305	0.008563377
ENSG00000183597	604.1484257	1.185600128	0.451125357	2.62809463	0.008586463
ENSG00000177683	2521.980719	0.697009928	0.265239368	2.627852469	0.008592577
ENSG00000167333	697.8352467	1.155658238	0.439866572	2.627292712	0.008606726
ENSG00000100116	863.8022182	-1.468656763	0.559003167	-2.627278073	0.008607097
ENSG00000225648	1948.449032	1.085516348	0.413172811	2.627269555	0.008607312
ENSG00000099992	424.347462	0.848243521	0.323004101	2.62610759	0.008636751
ENSG00000065809	3049.642969	1.066088296	0.405999803	2.625834519	0.008643683
ENSG00000155363	2317.905803	-0.830502049	0.316395969	-2.62488189	0.008667903
ENSG00000204390	30.94384115	1.850115777	0.704947445	2.624473341	0.008678309
ENSG00000214413	1044.820647	1.037663521	0.395386853	2.624425958	0.008679516
ENSG00000070759	415.3692695	0.84241787	0.321005075	2.624313244	0.008682389
ENSG00000071564	4901.083867	-0.785970148	0.299535061	-2.623967106	0.008691218
ENSG00000132781	585.8972065	-1.195312431	0.455604186	-2.62357649	0.00870119
ENSG00000130021	596.8107211	-0.947737573	0.361265645	-2.623381403	0.008706175
ENSG00000156097	15.46430423	1.893735746	0.721900384	2.623264635	0.00870916
ENSG00000100393	4645.847448	0.891632394	0.339912952	2.623119801	0.008712863
ENSG00000175416	2953.934053	1.472683601	0.561534845	2.622604123	0.00872606
ENSG00000110925	2103.167263	1.009079171	0.384814901	2.622245573	0.008735246
ENSG00000153774	801.502747	-1.215696604	0.463649375	-2.622017133	0.008741104
ENSG00000145293	2324.975108	-0.882344059	0.336595309	-2.62137955	0.00875747
ENSG00000160345	154.4914126	-1.248447472	0.476272008	-2.621290885	0.008759748
ENSG00000132128	2911.01205	1.106817991	0.42228118	2.621045038	0.008766068
ENSG00000141497	53.29848641	-1.504592191	0.574052704	-2.621000095	0.008767224
ENSG00000244219	59.25628164	1.287715541	0.491387867	2.62056845	0.008778331
ENSG00000226763	42.42632687	1.711861936	0.653251812	2.620523825	0.00877948
ENSG00000197106	948.0797246	-1.259647312	0.480713609	-2.620369568	0.008783452
ENSG00000243910	5.262050745	4.847351168	1.850311359	2.619748911	0.008799453
ENSG00000157510	438.4111725	-1.061053385	0.405022302	-2.619740638	0.008799666
ENSG00000174307	1416.724976	0.875994009	0.334419418	2.619447205	0.00880724
ENSG00000213995	1637.667708	1.014391468	0.387258926	2.61941404	0.008808097
ENSG00000167889	283.0800245	-1.087227179	0.415113067	-2.619110948	0.008815927
ENSG00000213397	13.47947331	-2.220369475	0.847801428	-2.618973502	0.00881948
ENSG00000159166	1647.569559	-1.002155226	0.382662037	-2.618904225	0.008821271
ENSG00000160410	2285.090835	-0.758443761	0.289741854	-2.61765344	0.008853667
ENSG00000123737	2529.402546	-0.908393662	0.347032908	-2.617600931	0.00885503
ENSG00000174226	16.07022555	2.241717519	0.856407902	2.617581543	0.008855533
ENSG00000263045	5.028212647	4.783516045	1.82750782	2.617507839	0.008857445
ENSG00000169018	3963.74071	0.779515831	0.297830684	2.617312029	0.008862529
ENSG00000130821	3663.309903	-0.675531157	0.258103703	-2.617285796	0.00886321
ENSG00000179967	28.64455455	-1.791284413	0.684493139	-2.616950136	0.00887193

ENSG00000267546	24.2147259	1.842659327	0.7042523	2.616476123	0.008884258
ENSG00000239445	51.06478867	1.665904878	0.636792809	2.616086198	0.00889441
ENSG00000052723	3096.428771	0.723525157	0.276707179	2.614768291	0.008928801
ENSG00000130299	1354.205811	-1.239102829	0.473976121	-2.614272692	0.008941764
ENSG00000152700	2754.61457	0.961538028	0.367871195	2.613789942	0.008954407
ENSG00000253965	7.198755803	3.272074187	1.251994527	2.613489211	0.008962291
ENSG00000245748	10.51789752	3.552967871	1.359512705	2.613412775	0.008964296
ENSG00000033030	2225.339315	1.11428964	0.426460851	2.612876744	0.008978368
ENSG00000070770	6216.366878	0.826416909	0.316336848	2.61245857	0.008989359
ENSG00000179241	1222.838211	-1.440912697	0.551621782	-2.61213887	0.00899777
ENSG00000163349	4242.212626	0.898024699	0.343794918	2.61209416	0.008998947
ENSG00000087085	258.554561	1.019263831	0.390270834	2.611683327	0.009009767
ENSG00000231638	25.71263694	2.559993767	0.980298256	2.611443764	0.009016082
ENSG00000010932	18.87959171	-1.993911814	0.763896792	-2.610184823	0.009049332
ENSG00000178234	1164.825762	-0.957524191	0.366875637	-2.609942155	0.009055753
ENSG00000165678	14732.34552	1.208191037	0.462936393	2.609842422	0.009058394
ENSG00000072657	67.88838454	-1.454726575	0.557608133	-2.608869005	0.009084201
ENSG00000216895	95.19627264	1.147388113	0.439805157	2.608855525	0.009084559
ENSG00000226658	4.704993313	5.705333424	2.187402736	2.608268396	0.009100157
ENSG00000180881	753.2005373	0.833596192	0.319622958	2.608061062	0.009105671
ENSG00000170631	675.435287	0.912112808	0.349738758	2.607983211	0.009107742
ENSG00000257194	3.160959031	5.131109158	1.967625415	2.607767271	0.009113489
ENSG00000103326	1035.795938	-0.749699472	0.287512779	-2.607534432	0.00911969
ENSG00000157911	286.659816	-1.277301379	0.489960956	-2.606945236	0.009135397
ENSG00000167110	4604.991649	0.75423507	0.289330201	2.606831461	0.009138433
ENSG00000243323	17.49961946	-1.8919608	0.725841069	-2.606577226	0.00914522
ENSG00000237940	26.51997416	-2.025586848	0.77714174	-2.606457412	0.00914842
ENSG00000187097	639.9453331	-1.196636293	0.45913805	-2.60626688	0.009153511
ENSG00000121895	14.39508067	2.250836887	0.863628109	2.606257096	0.009153772
ENSG00000127080	657.8444619	1.199082684	0.460116093	2.606043785	0.009159475
ENSG00000213762	546.2328916	0.854618224	0.327937594	2.606039195	0.009159598
ENSG00000226582	5.207809601	5.851532207	2.2458403	2.605497909	0.009174083
ENSG00000132376	1413.855385	0.849784346	0.326192203	2.6051645	0.009183016
ENSG00000144214	285.2120932	1.023504846	0.392875708	2.605161951	0.009183084
ENSG00000211698	11.35343806	3.944317438	1.514040763	2.605159341	0.009183154
ENSG00000136754	3309.356644	0.918784223	0.352873771	2.603719229	0.009221827
ENSG00000253180	9.758562776	2.726878695	1.047310408	2.603696741	0.009222432
ENSG00000253326	22.0231411	2.019234708	0.775685289	2.603162309	0.009236821
ENSG00000103037	532.3424517	-1.120537889	0.430732655	-2.601469557	0.00928253
ENSG00000216901	12.69093955	2.201098978	0.846125747	2.60138518	0.009284814
ENSG00000271835	3.1505286	5.126835283	1.971135705	2.600955008	0.009296465
ENSG00000268218	231.948391	1.135002518	0.436418871	2.600718242	0.009302883
ENSG00000061455	61.10828388	1.423796184	0.54748607	2.600607142	0.009305895
ENSG00000137876	9645.388725	0.730334259	0.280837371	2.600559374	0.009307191
ENSG00000165644	317.8913179	-1.939535288	0.74594482	-2.600105579	0.009319508
ENSG00000125885	1112.46366	-1.300473148	0.500223993	-2.599781632	0.00932831
ENSG00000255837	10.5976462	-2.302197339	0.885771081	-2.599088396	0.00934717

ENSG00000137947	2734.216472	1.053768029	0.405449658	2.59901077	0.009349284
ENSG00000019144	1678.650697	-0.712176428	0.27402259	-2.598969767	0.009350401
ENSG00000162998	159.3794225	1.787904391	0.687961304	2.598844414	0.009353816
ENSG00000154734	19.03027727	2.396799037	0.92226191	2.598826873	0.009354294
ENSG00000184787	5035.362818	-0.803348241	0.309167922	-2.598420419	0.009365375
ENSG00000258366	35.57940177	-2.043667177	0.786544982	-2.598283916	0.009369099
ENSG00000181544	189.9353162	-1.262944412	0.486088723	-2.598176735	0.009372024
ENSG00000119946	4.903047477	4.748673248	1.827870192	2.597926959	0.009378844
ENSG00000240163	17.24006895	2.334187041	0.898632261	2.597488586	0.009390824
ENSG00000157450	2296.35477	0.977887904	0.376497412	2.597329686	0.00939517
ENSG00000099622	4970.224443	-0.716038265	0.275771226	-2.59649375	0.009418062
ENSG00000231503	46.96941539	-1.55630052	0.599396262	-2.596446821	0.009419349
ENSG00000140950	1788.717618	0.728358107	0.280556882	2.596115638	0.009428433
ENSG00000129250	6572.112971	-0.729424136	0.280984602	-2.595957678	0.009432768
ENSG00000214293	362.8306916	0.845400993	0.325662824	2.595939518	0.009433267
ENSG00000100982	808.9244488	-0.924820891	0.356260961	-2.595908595	0.009434116
ENSG00000265158	4.778006329	5.726795991	2.206121264	2.595866367	0.009435275
ENSG00000253676	5.201615076	-3.919496633	1.510014966	-2.595667408	0.00944074
ENSG00000175772	156.2739638	-1.71840979	0.662098914	-2.595397387	0.009448161
ENSG00000137941	2220.350467	1.138147044	0.438621567	2.594826909	0.009463857
ENSG00000141759	2652.675788	-0.958180353	0.369306194	-2.59454179	0.00947171
ENSG00000223638	8.86349264	3.471264132	1.338021319	2.594326474	0.009477644
ENSG00000236432	49.88055911	1.569783531	0.605215429	2.593759934	0.009493275
ENSG00000176994	1132.86093	1.181924127	0.455682701	2.593743685	0.009493723
ENSG00000272077	173.3511727	1.183781758	0.456445447	2.593479168	0.009501029
ENSG00000169629	295.6042778	0.953337883	0.367596558	2.593435283	0.009502242
ENSG00000166444	155.9357776	-1.017616615	0.392438056	-2.593063032	0.009512533
ENSG00000270346	357.516225	1.276205757	0.492194136	2.592891025	0.009517292
ENSG00000135698	1314.099914	-0.76757868	0.296044505	-2.592781379	0.009520327
ENSG00000086061	16426.86884	1.229513938	0.474208346	2.592771613	0.009520597
ENSG00000197451	7242.16556	-0.784822072	0.302704756	-2.59269819	0.00952263
ENSG00000130347	332.6577151	-0.763649125	0.294538488	-2.592697239	0.009522656
ENSG00000153093	136.4498941	-1.502689693	0.579680886	-2.592270554	0.009534476
ENSG00000163006	362.6807683	-1.125824545	0.434325442	-2.592122029	0.009538593
ENSG00000243648	6.090058486	4.091439743	1.578435564	2.592085376	0.009539609
ENSG00000146830	2665.925027	-0.831112715	0.320640924	-2.592035676	0.009540988
ENSG00000170458	25.96016232	2.49587534	0.963018014	2.591722381	0.00954968
ENSG00000273131	377.329779	1.081293789	0.417278366	2.591300859	0.009561386
ENSG00000066583	1788.140069	-0.790923406	0.30529567	-2.590680064	0.00957865
ENSG00000189433	73.71037558	-1.51505892	0.58482464	-2.590620876	0.009580297
ENSG00000108465	3210.393743	-1.074962084	0.415080229	-2.589769422	0.009604024
ENSG00000160051	157.3154544	-1.214924899	0.469193646	-2.589389071	0.009614639
ENSG00000162290	3652.915829	0.908335212	0.35083192	2.589089417	0.00962301
ENSG00000116133	7402.476739	-0.690915285	0.266919522	-2.588477902	0.009640113
ENSG00000106031	273.0986302	-1.169934826	0.45197798	-2.588477488	0.009640125
ENSG00000224138	113.0779653	1.218443698	0.470767757	2.5882055	0.00964774
ENSG00000204959	667.1847638	0.924346374	0.35716026	2.588043737	0.009652272

ENSG00000101311	1488.35808	-1.491493365	0.576383115	-2.587677058	0.009662552
ENSG00000257743	59.26331386	2.254190773	0.871249516	2.587307919	0.009672911
ENSG00000198917	1274.47495	-0.749018092	0.289543529	-2.586892876	0.00968457
ENSG00000144659	1953.915036	0.899903922	0.347895359	2.586708617	0.009689749
ENSG00000163536	516.6408171	0.923164827	0.356939921	2.586331126	0.009700369
ENSG00000225361	160.8795245	1.135515628	0.439374461	2.584391509	0.009755099
ENSG00000251474	706.0807586	0.874772019	0.338488873	2.584344982	0.009756415
ENSG00000139428	392.2699438	-1.39789427	0.54092687	-2.584257408	0.009758893
ENSG00000011260	2898.082785	-0.967004444	0.374199246	-2.584196663	0.009760612
ENSG00000107099	13.75206213	2.544229749	0.984641571	2.583914619	0.009768597
ENSG00000196668	245.7204163	-2.037670277	0.78866941	-2.583681135	0.009775212
ENSG00000013275	8722.955362	1.445699982	0.55958025	2.583543614	0.00977911
ENSG00000162851	887.3990943	-0.926587186	0.358650145	-2.583540532	0.009779198
ENSG00000262877	65.15119956	-1.112616358	0.430773756	-2.582832272	0.009799295
ENSG00000198331	560.0368836	-0.947028246	0.366806195	-2.581821841	0.009828031
ENSG00000141504	1454.643641	0.791387619	0.306527602	2.581782569	0.009829149
ENSG00000164985	4408.878685	-0.866228577	0.33551882	-2.581758532	0.009829834
ENSG00000101574	976.8638853	1.12445787	0.435562456	2.581622578	0.009833706
ENSG00000123870	126.2244405	1.178556991	0.456550759	2.581436932	0.009838997
ENSG00000167965	1228.998879	-0.995996423	0.385924604	-2.580805711	0.009857004
ENSG00000121989	887.0259652	1.000274276	0.387607103	2.580639695	0.009861745
ENSG00000249084	5.389885876	-4.848723682	1.878945371	-2.580555964	0.009864137
ENSG00000182004	2195.052376	-0.872608089	0.338201829	-2.580140066	0.009876025
ENSG00000090520	8053.790634	0.973354622	0.377294474	2.579827403	0.009884971
ENSG00000145331	536.7652396	0.933060962	0.361719263	2.579516931	0.009893861
ENSG00000161326	2292.607358	1.087973596	0.42177732	2.579497627	0.009894414
ENSG00000100504	7136.230206	-1.165859771	0.452006299	-2.579299832	0.009900082
ENSG00000253250	16.3143334	2.731903093	1.059251763	2.579087606	0.009906166
ENSG00000084093	3353.558771	0.758217077	0.294187915	2.577322314	0.009956906
ENSG00000100319	440.1182944	1.333189759	0.517302848	2.577193928	0.009960605
ENSG00000255142	19.44038349	-1.739567	0.675015651	-2.577076542	0.009963988
ENSG00000144320	3000.638452	0.963646425	0.373987059	2.576683875	0.009975313
ENSG00000103710	3.062869382	5.088614677	1.975361936	2.576041679	0.00999386
ENSG00000118217	4032.185863	0.716765283	0.278247986	2.575994504	0.009995224
ENSG00000175911	87.89962415	1.289854317	0.500773075	2.575726174	0.010002983
ENSG00000182628	2191.46211	-0.929134542	0.360756796	-2.57551501	0.010009093
ENSG00000160867	2313.5663	-1.026152439	0.398466205	-2.575255886	0.010016595
ENSG00000136280	1171.762878	-0.880925569	0.342079252	-2.575209004	0.010017953
ENSG00000248919	4.483749127	4.608038837	1.789488062	2.575059837	0.010022275
ENSG00000118520	9.392419844	2.422659856	0.940969951	2.574641043	0.010034417
ENSG00000120647	514.5150663	-0.951637179	0.369622168	-2.574621498	0.010034983
ENSG00000261652	63.92333172	1.143892587	0.444334884	2.574392935	0.010041616
ENSG00000171566	6784.988958	0.873270739	0.339240109	2.574196613	0.010047316
ENSG00000166902	1253.462568	-0.84293607	0.32752622	-2.573644543	0.01006336
ENSG00000269952	19.28008324	2.716527616	1.055674404	2.573262746	0.010074469
ENSG00000197140	221.3548046	1.630381325	0.633630142	2.573080439	0.010079778
ENSG00000157193	2508.763702	-1.382154609	0.53717239	-2.573018708	0.010081576

ENSG00000052841	3311.480018	0.656093559	0.255061487	2.572295672	0.010102657
ENSG00000130204	4454.304435	-0.852569228	0.331489667	-2.571933041	0.010113245
ENSG00000257122	143.6455767	1.537157803	0.597708853	2.571750102	0.01011859
ENSG00000173166	322.7660237	0.913188443	0.355100581	2.571633202	0.010122007
ENSG00000059145	2245.924521	1.376375634	0.535254858	2.571439782	0.010127663
ENSG00000150764	447.8647032	-1.221766078	0.475177808	-2.571176636	0.010135362
ENSG00000185238	1100.116112	-0.872177085	0.339234976	-2.571011677	0.010140191
ENSG00000108852	840.5139615	-0.740723872	0.28815628	-2.570563003	0.010153336
ENSG00000006007	3236.336525	0.798603005	0.310691065	2.570408664	0.010157861
ENSG00000164136	282.2324594	0.860388169	0.334750578	2.570236541	0.01016291
ENSG00000124145	12191.27632	1.011091739	0.393389782	2.570203357	0.010163883
ENSG00000171017	80.97219271	1.132956104	0.440847746	2.56994873	0.010171357
ENSG00000141644	3667.819766	1.119678248	0.435684864	2.569926889	0.010171998
ENSG00000146278	2642.743583	1.008488438	0.39247582	2.569555591	0.010182905
ENSG00000124508	1112.51918	0.891494743	0.346978943	2.569305025	0.010190272
ENSG00000116183	20.1971321	2.259416258	0.879518549	2.568923941	0.010201485
ENSG00000101417	151.3012088	-1.092803919	0.425446696	-2.56860361	0.010210918
ENSG00000103671	1432.194189	0.910436304	0.354458004	2.568530807	0.010213063
ENSG00000152234	17859.23137	-0.723410188	0.281659125	-2.568388962	0.010217244
ENSG00000260648	6.455275725	-4.247412951	1.653747848	-2.568355845	0.01021822
ENSG00000097021	4168.311603	-0.873744531	0.340202006	-2.568310934	0.010219544
ENSG00000198832	1549.09013	1.037030873	0.403831172	2.567981241	0.010229269
ENSG00000131844	1947.640198	-1.192456831	0.464367203	-2.567917852	0.01023114
ENSG00000005339	4375.966489	0.9172736	0.357240564	2.56766362	0.010238646
ENSG00000131591	515.8260538	-0.839796175	0.327067277	-2.56765575	0.010238878
ENSG00000242622	35.88910268	1.787397075	0.696210724	2.567321953	0.010248741
ENSG00000184271	384.6371486	0.85061197	0.331346969	2.5671337	0.010254307
ENSG00000169752	209.247494	0.908077604	0.353743642	2.567049966	0.010256783
ENSG00000002079	69.95614104	-1.477982802	0.575831593	-2.566692799	0.010267353
ENSG00000150687	493.7481333	-0.873486175	0.340331422	-2.56657516	0.010270836
ENSG00000105641	69.18741234	-1.795903935	0.699812567	-2.566264199	0.010280049
ENSG00000105186	2386.802807	-1.02475207	0.399345694	-2.566077674	0.010285579
ENSG00000161249	3328.724162	-1.032190385	0.402267661	-2.565929318	0.010289979
ENSG00000166396	6.184305609	-3.501270653	1.364668958	-2.565655673	0.0102981
ENSG00000054967	928.982815	1.25694747	0.489933941	2.565544792	0.010301392
ENSG00000240350	4.552674263	5.657225567	2.205260225	2.565332428	0.0103077
ENSG00000225605	4.531813401	5.650867435	2.203060966	2.565007289	0.010317364
ENSG00000178199	26.83722745	2.310253521	0.900718537	2.564900605	0.010320536
ENSG00000132142	7734.044893	-1.247053709	0.486330388	-2.564210958	0.010341067
ENSG00000237522	61.02356809	1.326485886	0.517315803	2.564170434	0.010342275
ENSG00000078668	3603.258379	-0.688975142	0.268714322	-2.563968813	0.010348284
ENSG00000181191	1145.435095	0.691683883	0.269784086	2.563842419	0.010352053
ENSG00000230581	8.071642519	4.036971585	1.574811724	2.563462999	0.010363375
ENSG00000169245	9.977474563	-3.128147516	1.220541268	-2.56291827	0.010379648
ENSG00000162616	3197.088791	1.383645813	0.539874863	2.562900976	0.010380165
ENSG00000125875	3225.485553	0.659216767	0.257297109	2.562083846	0.01040462
ENSG00000182329	7.635441102	3.977572856	1.552543908	2.56197125	0.010407993

ENSG00000183971	1514.860689	-1.401337262	0.546994681	-2.561884622	0.01041059
ENSG00000204262	203.34051	1.353352731	0.528264606	2.561884168	0.010410603
ENSG00000162521	6074.926217	-0.776782606	0.30326127	-2.561430305	0.010424215
ENSG00000244754	4498.845493	0.680372691	0.265643866	2.561221163	0.010430493
ENSG00000196976	492.4321076	-1.186349503	0.463201019	-2.561197956	0.01043119
ENSG00000169258	859.9906225	-0.819514306	0.320010019	-2.560902023	0.010440079
ENSG00000119927	1584.220817	-0.842210183	0.328965195	-2.560180211	0.01046179
ENSG00000186567	339.3255797	0.908243635	0.35477856	2.560029659	0.010466323
ENSG00000172965	1206.116334	1.516484257	0.592526576	2.559352302	0.010486741
ENSG00000126070	3283.30634	0.773860502	0.30236653	2.559345779	0.010486937
ENSG00000230055	537.857177	-1.432580535	0.559779601	-2.559186743	0.010491737
ENSG00000166669	747.5693903	1.124749943	0.439550393	2.558864607	0.010501463
ENSG00000137054	1829.55012	-0.890480348	0.348073402	-2.558311962	0.010518169
ENSG00000127337	748.2262026	-0.818643944	0.320061763	-2.55776865	0.010534615
ENSG00000134058	3930.852405	0.8535784	0.3337277	2.557709173	0.010536417
ENSG00000173581	60.12882076	1.563694277	0.61147625	2.5572445	0.010550503
ENSG00000248487	125.5381112	-1.179282802	0.46130921	-2.556382524	0.010576678
ENSG00000139865	19.52210039	2.915921634	1.140654253	2.556358884	0.010577396
ENSG00000232686	6.701988113	3.189916604	1.247837351	2.556356085	0.010577482
ENSG00000100902	631.9013916	1.215873383	0.475751081	2.555692319	0.010597678
ENSG00000100815	2251.707527	0.924653092	0.361943368	2.554689971	0.010628242
ENSG00000167291	2568.424934	-1.230319502	0.481635967	-2.554459358	0.010635284
ENSG00000161664	69.89238821	1.597648964	0.625447574	2.554409084	0.01063682
ENSG00000069248	2554.947274	-0.698004373	0.273295698	-2.554026199	0.010648524
ENSG00000185168	123.254426	-1.453328516	0.569064057	-2.553892656	0.010652609
ENSG00000083067	7.219469496	3.799279407	1.487716773	2.553765256	0.010656507
ENSG00000226416	63.38533941	-1.827588259	0.715651756	-2.553739644	0.010657291
ENSG00000213799	753.1182842	1.17808652	0.461323164	2.553712044	0.010658136
ENSG00000155393	569.241254	-1.19502531	0.467966458	-2.55365591	0.010659854
ENSG00000114738	3000.877341	-0.948794061	0.371582117	-2.553389995	0.010667997
ENSG00000100239	2526.83414	-0.778085538	0.304736251	-2.553308101	0.010670506
ENSG00000254088	12.26388426	3.12149501	1.222634616	2.553089017	0.01067722
ENSG00000123240	1388.226859	0.855659813	0.33515945	2.552993246	0.010680157
ENSG00000054267	2666.352939	0.883550904	0.34609838	2.552889448	0.01068334
ENSG00000156603	861.6217858	0.835928863	0.327465881	2.552720489	0.010688523
ENSG00000090006	5195.990797	-0.915050241	0.358463777	-2.552699323	0.010689173
ENSG00000125826	4610.796729	0.884030528	0.346328025	2.55258155	0.010692787
ENSG00000110921	748.2248561	-0.816173618	0.319755109	-2.552495945	0.010695415
ENSG00000185504	1506.533388	-0.791703663	0.310288581	-2.55150757	0.010725799
ENSG00000130590	65.39007833	-1.116304136	0.437555168	-2.551230603	0.010734327
ENSG00000219891	140.4495238	0.92096112	0.361032131	2.55091179	0.010744152
ENSG00000118939	102.3111588	1.037882755	0.406927518	2.550534702	0.010755782
ENSG00000188419	1338.948454	0.837186341	0.328257462	2.550395464	0.010760079
ENSG00000250571	576.5027062	-0.98251086	0.385283367	-2.550099338	0.010769223
ENSG00000181135	907.2192458	1.268356156	0.497390712	2.550019786	0.010771681
ENSG00000258112	3.008578705	5.070960233	1.98875843	2.549812062	0.0107781
ENSG00000166823	146.7694153	-1.450451656	0.568875355	-2.549682708	0.0107821

ENSG00000128253	67.19952074	1.492746805	0.585524925	2.549416331	0.01079034
ENSG00000078902	1589.371734	1.047045569	0.41073654	2.549190212	0.010797339
ENSG00000230772	9.423963049	2.879768021	1.129790001	2.548940969	0.010805059
ENSG00000179021	1385.666508	0.955773103	0.374972328	2.548916366	0.010805821
ENSG00000204673	3896.859191	1.151467512	0.451758311	2.548857397	0.010807648
ENSG00000205500	16.4697042	2.135380685	0.837781577	2.548851328	0.010807836
ENSG00000062370	238.0275569	1.224445773	0.480488741	2.548333956	0.01082388
ENSG00000167874	20.35450324	1.651656953	0.648156224	2.548238975	0.010826828
ENSG00000141580	8737.443944	0.660125733	0.259075077	2.548009411	0.010833955
ENSG00000185532	207.6959146	1.641169807	0.644134306	2.547868964	0.010838317
ENSG00000171163	1188.902081	-1.096406449	0.430396087	-2.547435914	0.010851778
ENSG00000269845	18.51991952	2.525973811	0.991929046	2.546526712	0.010880088
ENSG00000058262	13514.36451	0.774373941	0.304128519	2.546206264	0.010890082
ENSG00000164715	3593.614043	0.945252322	0.37125887	2.546073368	0.010894229
ENSG00000111364	1474.654168	-0.699558387	0.274766861	-2.546007129	0.010896296
ENSG00000166801	1227.955996	-0.711579841	0.279492629	-2.54596997	0.010897456
ENSG00000147533	2307.665206	0.907951773	0.35667873	2.545573083	0.010909852
ENSG00000267942	9.33995281	-2.684083359	1.0545296	-2.545289728	0.01091871
ENSG00000238181	10.37542655	3.208824772	1.26069157	2.545289306	0.010918723
ENSG00000197932	212.3600555	-1.307474075	0.513715474	-2.545132744	0.01092362
ENSG00000138413	7116.877901	0.715610729	0.281169293	2.545124051	0.010923892
ENSG00000222040	46.39290864	-1.334717257	0.524550754	-2.544495927	0.010943559
ENSG00000007520	797.6331212	-0.85226895	0.334959751	-2.544392115	0.010946812
ENSG00000270081	766.7870818	-0.683341536	0.268579617	-2.544279208	0.010950352
ENSG00000178229	549.9794503	0.888942001	0.349397913	2.544210964	0.010952491
ENSG00000168209	4150.127879	1.311834992	0.515719051	2.543700857	0.010968498
ENSG00000137547	1414.199623	-0.845212816	0.332360134	-2.543063168	0.010988536
ENSG00000137434	59.07845612	1.05736211	0.415880289	2.54246748	0.011007284
ENSG00000075336	870.3045635	-0.940719817	0.370038858	-2.542219004	0.011015113
ENSG00000137038	464.566991	-1.144913745	0.450787403	-2.539808647	0.011091313
ENSG00000265194	147.6769921	-1.979428203	0.779376969	-2.539757116	0.011092948
ENSG00000163510	2425.897097	0.988789762	0.389327897	2.539735194	0.011093643
ENSG00000164663	1657.403234	0.836995955	0.32957145	2.539649462	0.011096362
ENSG00000163521	152.8587335	-0.943507093	0.37155723	-2.53933181	0.011106443
ENSG00000178252	4040.464972	-0.713159798	0.280947196	-2.538412231	0.011135673
ENSG00000166197	13073.0212	-0.698651513	0.275239509	-2.538340207	0.011137965
ENSG00000037757	271.4421648	-1.05457303	0.415496981	-2.538100346	0.011145602
ENSG00000130173	6.95601303	3.925865804	1.546871247	2.537939607	0.011150722
ENSG00000088682	1418.139348	-0.870010165	0.342857484	-2.537527124	0.011163872
ENSG00000181666	889.1172896	0.90564674	0.356911942	2.53745149	0.011166284
ENSG00000128694	379.516951	-1.308865829	0.515857859	-2.537260618	0.011172375
ENSG00000256294	310.1300043	1.335666023	0.526482169	2.536963453	0.011181863
ENSG00000184702	381.3827881	-1.125912053	0.443827572	-2.536823138	0.011186345
ENSG00000081087	680.2260087	0.823398327	0.324613855	2.536547079	0.011195169
ENSG00000104870	28.85904968	-1.835212578	0.723510183	-2.53654008	0.011195393
ENSG00000199753	130.0843721	-0.872363215	0.343933715	-2.536428318	0.011198967
ENSG00000268816	26.04965084	-1.547886846	0.610269991	-2.536396792	0.011199975

ENSG00000116514	1529.134382	0.916805476	0.361498617	2.536124439	0.01120869
ENSG00000214198	78.74160719	1.533294749	0.604614465	2.535987538	0.011213072
ENSG00000023572	742.1742814	0.898552007	0.35439685	2.535440162	0.011230611
ENSG00000235408	13.81004821	-2.222941285	0.87691061	-2.534969082	0.011245725
ENSG00000159882	280.2734036	1.273989845	0.502594802	2.53482495	0.011250353
ENSG00000260233	21.79142302	1.711293945	0.67513155	2.534756293	0.011252558
ENSG00000100027	85.20673838	-1.280100645	0.505046155	-2.534621107	0.011256901
ENSG00000254923	3.083668969	5.109156515	2.01627234	2.533961515	0.011278112
ENSG00000080200	674.3962012	0.851878613	0.336245173	2.53350436	0.011292834
ENSG00000235481	3.248618248	5.169404514	2.040568101	2.53331634	0.011298893
ENSG00000185115	1444.874961	0.926600427	0.365811478	2.532999872	0.011309099
ENSG00000197021	869.4004004	0.975221569	0.385040676	2.532775443	0.011316342
ENSG00000135318	3788.716058	-1.374865766	0.542880641	-2.532537841	0.011324015
ENSG00000131697	632.1999895	-1.072135035	0.423438275	-2.531974783	0.011342215
ENSG00000265205	35.26043186	2.210582327	0.873144693	2.531747997	0.011349553
ENSG00000113312	3266.199573	1.066335854	0.421228785	2.531488571	0.011357952
ENSG00000249740	51.13736237	2.095143896	0.827696097	2.531296093	0.011364187
ENSG00000267595	13.05063315	2.161624872	0.853984261	2.531223317	0.011366545
ENSG00000263053	27.62828363	-1.784551636	0.705414416	-2.529791844	0.011413021
ENSG00000150776	2112.514172	0.951859396	0.376325055	2.529354298	0.011427261
ENSG00000108510	9892.619205	0.893496835	0.353262922	2.529268655	0.01143005
ENSG00000158483	177.5533554	-1.530966029	0.605323497	-2.529170001	0.011433263
ENSG00000204588	91.01437919	-1.276419727	0.504839467	-2.528367554	0.011459431
ENSG00000136738	2401.543345	1.08199211	0.428015269	2.527928762	0.011473763
ENSG00000187446	5923.521788	0.707709276	0.28000151	2.527519497	0.011487145
ENSG00000005889	1511.781317	0.92975379	0.367917228	2.527073265	0.011501751
ENSG00000270696	270.3255341	-0.996356442	0.394328991	-2.526713641	0.011513534
ENSG00000168288	5999.535333	0.983672707	0.389319171	2.526648521	0.011515669
ENSG00000090924	951.2712138	-0.883523074	0.349712881	-2.526424169	0.011523026
ENSG00000205155	380.1300364	1.035699522	0.410055576	2.525754025	0.011545028
ENSG00000127022	34775.27425	0.642759401	0.254563949	2.52494277	0.011571713
ENSG00000180626	451.8630144	1.207478816	0.478270929	2.524675329	0.011580522
ENSG00000164904	2399.576329	-1.503483546	0.595618999	-2.52423705	0.011594971
ENSG00000142864	16163.60333	-0.855357505	0.338871189	-2.524137587	0.011598253
ENSG00000247595	24.2991535	1.591434081	0.630525227	2.523981618	0.0116034
ENSG00000041515	12.0403147	2.159594826	0.855693188	2.523795744	0.011609536
ENSG00000231725	3.649238112	5.338385316	2.116246003	2.522573136	0.011649972
ENSG00000111328	1276.85815	-0.749709794	0.297230834	-2.522315013	0.011658525
ENSG00000246526	17.89004495	2.685736094	1.064936604	2.521968052	0.011670031
ENSG00000185847	630.9144417	-1.012402011	0.401539801	-2.521299281	0.011692236
ENSG00000124459	893.2046978	1.016651652	0.403244562	2.521178827	0.011696239
ENSG00000267590	5.490072214	4.00820043	1.590415735	2.520221815	0.01172809
ENSG00000134291	2458.998851	-0.86216763	0.342160059	-2.519778702	0.011742864
ENSG00000111845	1126.237988	-0.968256443	0.384291349	-2.519589488	0.011749177
ENSG00000234160	126.7582189	-1.060138312	0.420766422	-2.519541146	0.011750791
ENSG00000137674	12.36702678	-2.145431867	0.851653806	-2.519136123	0.011764317
ENSG00000166436	1086.404838	-1.40006972	0.555780556	-2.519105256	0.011765348

ENSG00000124549	306.3341568	1.130239331	0.448744645	2.518669233	0.011779926
ENSG00000176444	1997.785649	-0.787483	0.312709851	-2.518254534	0.011793806
ENSG00000182827	2571.306716	0.666165029	0.264556039	2.518048841	0.011800696
ENSG00000196705	907.7630318	1.335116833	0.530390747	2.517232512	0.011828074
ENSG00000219481	3735.566393	0.886152946	0.352109419	2.51669765	0.011846044
ENSG00000146757	1158.302407	1.223542819	0.486200662	2.516538775	0.011851386
ENSG00000269430	257.5095809	-1.838429713	0.730555255	-2.516482772	0.01185327
ENSG00000095787	9288.534903	0.740280658	0.294211046	2.516155211	0.011864292
ENSG00000198366	9.169007163	3.359726563	1.335450996	2.515799212	0.011876282
ENSG00000163625	3485.023123	0.921257802	0.366200364	2.515720604	0.011878931
ENSG00000154743	576.2702682	-1.370908961	0.545002149	-2.515419371	0.011889087
ENSG00000163738	561.2676792	0.892658577	0.354876368	2.515407213	0.011889497
ENSG00000197976	2228.682172	0.688246841	0.273640884	2.515146245	0.011898302
ENSG00000164430	5.551962474	3.896996078	1.549521801	2.514966924	0.011904355
ENSG00000138434	36022.0488	-1.212344319	0.482055567	-2.51494724	0.01190502
ENSG00000171792	633.2492824	-0.933536632	0.37120085	-2.514909738	0.011906286
ENSG00000168398	94.95622319	1.586197315	0.630760532	2.51473774	0.011912096
ENSG00000248121	31.72408968	1.501687893	0.597253997	2.514320374	0.011926204
ENSG00000138162	3194.169535	1.05885048	0.421161385	2.514120521	0.011932965
ENSG00000091972	12.31184844	2.614685667	1.040089574	2.513904313	0.011940283
ENSG00000129473	2005.216394	0.701499443	0.279060359	2.513791082	0.011944117
ENSG00000273142	480.3553931	1.166822971	0.464242119	2.51339317	0.011957599
ENSG00000162191	3284.229711	0.806856503	0.321068607	2.513034553	0.011969761
ENSG00000105879	3448.55468	1.174998985	0.467589711	2.512884601	0.01197485
ENSG00000181634	4.412623848	-5.595101703	2.227648568	-2.511662649	0.01201639
ENSG00000225330	5.142204917	4.044528993	1.610710505	2.511021677	0.01203823
ENSG00000217527	4.337570247	4.562886564	1.81733068	2.510762964	0.012047056
ENSG00000226803	37.16948275	1.327982392	0.529078126	2.509992998	0.012073356
ENSG00000136881	20.25033682	2.053021911	0.818198244	2.509198628	0.012100542
ENSG00000134463	326.8313868	-0.905686875	0.361078838	-2.508280135	0.012132044
ENSG00000224746	10.29540843	2.514600222	1.002529845	2.508254726	0.012132917
ENSG00000148735	56.39673952	2.104029094	0.838923743	2.508009949	0.012141325
ENSG00000144031	9.556395108	2.343547388	0.934565999	2.507631766	0.012154326
ENSG00000171453	1270.139582	-0.82955664	0.330841471	-2.507414314	0.012161806
ENSG00000176387	207.973696	-2.134624286	0.851560228	-2.506721447	0.01218567
ENSG00000234817	14.81153668	2.481836057	0.990077007	2.506710124	0.01218606
ENSG00000213931	5336.478272	0.854018174	0.34071408	2.506553808	0.01219145
ENSG00000030110	1580.114124	1.244619899	0.49658558	2.506355294	0.012198298
ENSG00000273366	14.49012429	-2.040561241	0.814166199	-2.506320262	0.012199506
ENSG00000157212	985.0231071	-0.762023555	0.304081576	-2.505983974	0.012211115
ENSG00000247137	71.28263015	1.552042999	0.61936955	2.505843237	0.012215977
ENSG00000186687	864.0798009	-1.049735702	0.418960071	-2.505574577	0.012225261
ENSG00000127955	3848.007045	-0.931516996	0.371784203	-2.505531401	0.012226754
ENSG00000035141	2421.931008	-0.639979537	0.255437185	-2.505428244	0.012230321
ENSG00000121741	2996.623071	0.695351232	0.277599413	2.504872844	0.012249542
ENSG00000103257	30450.14143	0.964012904	0.384877354	2.504727531	0.012254576
ENSG00000103494	567.9673703	-0.963808931	0.384827421	-2.504522489	0.012261681

ENSG00000177051	1484.349143	0.829376677	0.331177858	2.504324059	0.012268561
ENSG00000076053	1237.144603	0.678306946	0.270981351	2.503149916	0.012309339
ENSG00000022277	3004.771104	1.002407546	0.400491843	2.50294123	0.012316599
ENSG00000094804	2786.267762	-1.05282653	0.420640719	-2.502911587	0.012317631
ENSG00000197779	614.8806326	1.150592417	0.459775021	2.502511801	0.012331551
ENSG00000090097	1211.363678	-0.904498445	0.361452136	-2.502401716	0.012335387
ENSG00000181090	2310.81773	-0.671450711	0.268331968	-2.502313522	0.012338461
ENSG00000255284	86.86185115	-1.700959243	0.679919476	-2.501706899	0.01235962
ENSG00000127720	217.1452826	1.025373649	0.409875077	2.501673578	0.012360783
ENSG00000108479	847.8996761	-1.737664819	0.694636731	-2.501544681	0.012365284
ENSG00000259656	12.15588821	2.532667605	1.012482552	2.501443211	0.012368828
ENSG00000215895	39.62814151	1.947895965	0.778780552	2.501212902	0.012376875
ENSG00000168970	127.5943609	-0.971178116	0.388297001	-2.501121854	0.012380057
ENSG00000115486	1546.911942	0.854999025	0.341899475	2.500732191	0.012393686
ENSG00000140285	7.573415688	3.787915124	1.514820207	2.500570765	0.012399336
ENSG00000139496	9380.300688	0.862742756	0.345020607	2.500554278	0.012399913
ENSG00000243989	55.29249107	-1.358262283	0.543251807	-2.500244391	0.012410766
ENSG00000163795	816.2050243	0.969395821	0.387762818	2.499971053	0.012420345
ENSG00000152455	565.4643893	-0.724943778	0.290041218	-2.499450879	0.012438594
ENSG00000113712	7414.886156	0.713282753	0.285376198	2.499447252	0.012438722
ENSG00000128594	12.46795449	2.38296811	0.953471832	2.499253811	0.012445514
ENSG00000253686	3.344600011	5.209465169	2.084836741	2.498740101	0.012463568
ENSG00000135093	769.1865751	0.957194562	0.383088231	2.498626911	0.012467549
ENSG00000013619	376.2629059	-0.922207768	0.369163045	-2.498104238	0.012485947
ENSG00000173451	173.2851596	0.977498467	0.391300406	2.4980768	0.012486914
ENSG00000197070	1385.31568	-0.863486147	0.345687752	-2.497878918	0.012493886
ENSG00000235313	26.45334022	1.739078436	0.696247653	2.497787144	0.012497121
ENSG00000163141	96.32596788	1.298851292	0.520111705	2.497254491	0.01251591
ENSG00000160813	358.0394907	-1.34978153	0.540540611	-2.497095505	0.012521523
ENSG00000131043	2181.484377	1.072900232	0.429719085	2.496747919	0.012533802
ENSG00000126001	1487.874221	-0.83191102	0.333262137	-2.496266238	0.012550836
ENSG00000261026	10.23487969	2.81854243	1.129109562	2.496252379	0.012551327
ENSG00000146090	79.79600156	-1.597685707	0.64005182	-2.496181806	0.012553824
ENSG00000102984	234.7885218	0.978534691	0.392017585	2.49614999	0.01255495
ENSG00000003989	803.3476628	-1.77390994	0.710711673	-2.495962859	0.012561575
ENSG00000018699	896.9544547	-0.996428115	0.399235309	-2.495841659	0.012565868
ENSG00000174898	4.979363524	4.007426036	1.605645634	2.495834668	0.012566116
ENSG00000100647	930.4901474	0.836361479	0.335133898	2.495603947	0.012574291
ENSG00000140263	2840.501803	-0.836072311	0.335093987	-2.495038235	0.012594356
ENSG00000104884	1531.264868	-1.158158972	0.464188719	-2.495017487	0.012595092
ENSG00000268601	5.13076009	-4.015136372	1.609445412	-2.49473287	0.012605199
ENSG00000092445	1615.623681	-0.755409448	0.302830223	-2.49449821	0.012613536
ENSG00000145569	355.2530124	-1.171530233	0.469672614	-2.494355001	0.012618627
ENSG00000107362	1347.542143	1.013826492	0.406644042	2.493154667	0.012661368
ENSG00000165555	52.35321446	1.568041201	0.629152962	2.492305202	0.012691693
ENSG00000146221	37.58342148	1.832918524	0.735509056	2.492040728	0.012701147
ENSG00000134262	755.3265745	1.123861318	0.450990391	2.491985065	0.012703138

ENSG00000105711	298.2220871	-0.925135582	0.371308463	-2.491555336	0.012718515
ENSG00000164815	1021.342168	-0.853850664	0.342745148	-2.491211532	0.01273083
ENSG00000248213	92.60820155	1.461665165	0.586759658	2.491079856	0.012735549
ENSG00000157593	1558.479676	-1.112962005	0.446787722	-2.491030862	0.012737306
ENSG00000111224	224.9711172	0.904095532	0.362991164	2.490681928	0.01274982
ENSG00000013523	1021.936759	-1.099339386	0.441425896	-2.490427941	0.012758937
ENSG00000246560	49.00420282	1.376999189	0.552993123	2.490083748	0.0127713
ENSG00000162004	180.386199	-1.669361909	0.670451257	-2.489907943	0.012777619
ENSG00000066697	1425.098088	1.008015542	0.404948787	2.489242032	0.012801578
ENSG00000228146	6.860319002	-2.939698244	1.180973072	-2.489216999	0.01280248
ENSG00000140157	2993.359698	0.989049069	0.397398285	2.488810612	0.012817122
ENSG00000129007	523.4392153	-0.791485	0.318106217	-2.488115469	0.012842203
ENSG00000100744	1285.68054	0.789206711	0.317335309	2.486980456	0.012883248
ENSG00000146828	1191.250696	-1.054077085	0.423848743	-2.486918037	0.012885508
ENSG00000054148	1083.102792	-1.155028271	0.464504245	-2.486582809	0.012897655
ENSG00000133980	5.457997024	3.889887725	1.564718454	2.485998497	0.012918851
ENSG00000144583	70.00647381	-2.550871428	1.026112749	-2.485956275	0.012920384
ENSG00000106330	494.7482221	-1.13625946	0.457084619	-2.485884257	0.012922999
ENSG00000128536	197.6448498	-1.267543957	0.509924483	-2.485748376	0.012927934
ENSG00000099800	1196.621138	-0.897108416	0.360913374	-2.485661328	0.012931096
ENSG00000242687	106.2441203	1.405876528	0.565664319	2.485354794	0.012942237
ENSG00000130517	2322.000862	0.893938029	0.359684811	2.48533717	0.012942878
ENSG00000152795	9271.788339	-0.746405247	0.30038417	-2.48483549	0.012961132
ENSG00000146701	8554.506653	-0.783727567	0.315424052	-2.484679156	0.012966824
ENSG00000242779	8.56353075	3.50327226	1.40999902	2.484591981	0.01297
ENSG00000232411	12.37034315	2.810529457	1.131289856	2.4843584	0.012978511
ENSG00000139354	1255.790022	-1.019334294	0.410352524	-2.484045387	0.012989925
ENSG00000112578	1300.986748	-0.801483757	0.322670357	-2.483908854	0.012994906
ENSG00000164187	220.7071621	0.824931869	0.332145878	2.483643257	0.013004602
ENSG00000150867	3383.5032	-0.648054695	0.260929939	-2.483634869	0.013004908
ENSG00000147223	8.663923851	3.146424532	1.266913585	2.483535239	0.013008546
ENSG00000151576	1886.91197	-0.673205709	0.271076812	-2.483449998	0.01301166
ENSG00000011523	891.726105	-0.810775671	0.326494805	-2.483272808	0.013018135
ENSG00000137841	54.63395575	-2.310373417	0.930398864	-2.483207477	0.013020523
ENSG00000264769	61.605239	1.942561702	0.782288505	2.483178125	0.013021596
ENSG00000173141	988.4904032	-0.864138201	0.348124687	-2.482266365	0.013054965
ENSG00000144036	2982.30708	0.917086159	0.369472734	2.482148412	0.013059287
ENSG00000065054	1174.075894	-1.204029966	0.485092043	-2.482064968	0.013062346
ENSG00000105821	1754.885308	0.868657269	0.349976856	2.482042036	0.013063186
ENSG00000115137	338.0676493	0.781769632	0.314972778	2.482022847	0.01306389
ENSG00000122861	1838.055791	-1.350722455	0.544293664	-2.481606058	0.013079178
ENSG00000141431	5.704392332	4.970540565	2.003313862	2.481159173	0.013095588
ENSG00000153827	11829.28047	0.978584439	0.394436491	2.480968319	0.013102602
ENSG00000171428	301.2595952	1.077474595	0.434538052	2.479586289	0.01315349
ENSG00000015676	2136.260575	-0.716899471	0.289186768	-2.479018921	0.013174432
ENSG00000108296	1702.894345	0.926149979	0.373655572	2.47861948	0.013189193
ENSG00000221817	125.2600254	1.139656725	0.459803574	2.478573004	0.013190911

ENSG00000149292	305.8658279	-0.951524274	0.38390154	-2.478563317	0.01319127
ENSG00000170264	453.9650237	-1.149620769	0.463887546	-2.478231584	0.013203541
ENSG00000053900	1076.39208	-1.186803352	0.478964074	-2.477854636	0.013217498
ENSG00000259726	109.2441705	1.180961322	0.476672739	2.477509673	0.013230282
ENSG00000094975	2984.016866	0.761319991	0.307310001	2.477368092	0.013235532
ENSG00000133083	315.8940007	-1.479725982	0.597308198	-2.477324078	0.013237165
ENSG00000123901	68.57642132	1.435591097	0.579564431	2.477017258	0.01324855
ENSG00000215863	14.49535701	1.976553662	0.798035205	2.476775021	0.013257544
ENSG00000233280	524.1395823	0.926356726	0.37404408	2.476597744	0.01326413
ENSG00000104957	1386.825283	0.692623579	0.279680828	2.476478577	0.013268559
ENSG00000213699	2751.746962	0.829695129	0.335046425	2.476358701	0.013273015
ENSG00000169136	720.3099655	1.028040789	0.415190423	2.476070575	0.013283732
ENSG00000254233	5.697994186	4.97321039	2.008611284	2.475944663	0.013288417
ENSG00000177239	2342.225908	-0.972638171	0.392861297	-2.475780077	0.013294544
ENSG00000128805	378.364321	-0.798786723	0.322644281	-2.475750448	0.013295647
ENSG00000263607	5.919906123	4.12113632	1.664629861	2.475707313	0.013297254
ENSG00000225892	310.2382582	1.072614562	0.433291988	2.475500565	0.013304955
ENSG00000159915	64.41488965	1.600509055	0.646630855	2.475151073	0.013317983
ENSG00000131263	5894.60412	0.777951941	0.314315219	2.475069277	0.013321033
ENSG00000063854	1082.028608	-0.896160032	0.362084736	-2.475000854	0.013323586
ENSG00000250420	133.9279044	-1.587445751	0.641449398	-2.474779391	0.01333185
ENSG00000182446	16040.87824	1.22265678	0.494069568	2.474665228	0.013336112
ENSG00000184047	316.6198287	1.055678058	0.426627772	2.474471018	0.013343365
ENSG00000251556	24.25821542	2.193902177	0.886655395	2.474357218	0.013347616
ENSG00000213020	476.3287291	0.951401409	0.384550369	2.474061878	0.013358656
ENSG00000167395	1568.299193	1.106727165	0.447392499	2.473727581	0.013371161
ENSG00000214783	108.7644446	1.135068875	0.458916825	2.473365136	0.013384731
ENSG00000183386	904.0380664	0.812220907	0.328401859	2.473253071	0.013388929
ENSG00000110048	5980.258494	0.778324651	0.314725944	2.473023489	0.013397534
ENSG00000251361	99.96328625	-1.17071385	0.473403745	-2.472971246	0.013399492
ENSG00000240024	438.0589648	1.012713102	0.409518591	2.4729356	0.013400829
ENSG00000010810	1263.576571	0.9458146	0.382467554	2.472927678	0.013401126
ENSG00000164032	9888.809881	-0.906675337	0.366657214	-2.472814667	0.013405364
ENSG00000170854	1155.880263	-1.327006863	0.53664373	-2.472789282	0.013406316
ENSG00000235493	15.58090062	1.819733618	0.736003571	2.47245216	0.013418967
ENSG00000140365	1109.501153	-1.046962023	0.423480705	-2.472277986	0.013425507
ENSG00000047621	966.9908183	1.262896055	0.510825726	2.472264005	0.013426033
ENSG00000069812	619.5036163	-1.211384338	0.490085234	-2.471782973	0.013444111
ENSG00000082497	113.065324	-1.842253816	0.745385898	-2.471543695	0.013453111
ENSG00000126012	6141.791113	0.812321036	0.328709325	2.471244273	0.013464381
ENSG00000103269	148.4992833	-1.19387894	0.483116918	-2.471200855	0.013466016
ENSG00000033867	2467.161468	-0.919647975	0.372172982	-2.471022935	0.013472718
ENSG00000108924	137.4345971	-0.967951019	0.391733566	-2.470942249	0.013475758
ENSG00000128578	718.0328974	-0.728331154	0.294758853	-2.470939033	0.013475879
ENSG00000147155	1643.314349	-1.000437114	0.404987999	-2.470288297	0.01350042
ENSG00000071626	5999.883113	-0.848212407	0.343371528	-2.470246768	0.013501988
ENSG00000132004	180.2689023	-0.934449976	0.378356138	-2.469762964	0.013520261

ENSG00000131381	2383.109897	1.142814324	0.462803913	2.469327273	0.013536735
ENSG00000132498	15.54874073	-2.134984707	0.864650192	-2.469188958	0.013541969
ENSG00000168291	1562.04843	-0.784286241	0.317644776	-2.469067027	0.013546584
ENSG00000166667	28.2337738	1.66443271	0.674261166	2.468528209	0.013566996
ENSG00000151466	378.2300123	-1.034642742	0.419179904	-2.468254641	0.01357737
ENSG00000240270	7.964947163	3.374194395	1.367239061	2.467889113	0.013591241
ENSG00000100403	3251.689747	-0.623200015	0.252534433	-2.467782347	0.013595296
ENSG00000198585	1079.515427	-0.725856264	0.294173412	-2.467443472	0.013608171
ENSG00000132196	628.6705068	-0.972877208	0.39428616	-2.467439406	0.013608325
ENSG00000185728	4578.57431	0.918103379	0.372156567	2.466981536	0.013625738
ENSG00000221164	8.321173127	-2.7558055	1.117097084	-2.466934646	0.013627523
ENSG00000244357	3.761784796	4.353453292	1.765003102	2.466541439	0.013642494
ENSG00000176490	1552.693443	-1.152433543	0.467380024	-2.465731279	0.013673387
ENSG00000139370	793.0800883	-0.814141505	0.330252028	-2.465212735	0.013693193
ENSG00000259155	6.584137245	-4.10819466	1.666549342	-2.465090325	0.013697872
ENSG00000181788	1946.313376	0.74266474	0.301440217	2.46372149	0.013750291
ENSG00000185928	87.27284036	-1.107224192	0.449471917	-2.463389037	0.013763049
ENSG00000107862	9128.283337	0.925317117	0.375655895	2.463204037	0.013770153
ENSG00000228343	223.4311392	-0.787391413	0.319726981	-2.462699305	0.013789551
ENSG00000160505	86.2913982	1.695838774	0.688621032	2.462658988	0.013791101
ENSG00000164615	2163.838777	0.758687872	0.308137412	2.462173828	0.013809772
ENSG00000242265	646.6737458	-1.320897876	0.536507617	-2.462030051	0.013815309
ENSG00000239300	12.8158522	2.050020933	0.832861528	2.46141869	0.013838876
ENSG00000130731	677.9050184	-1.305527392	0.530481281	-2.461024428	0.013854093
ENSG00000006327	6978.700533	0.918226929	0.373141469	2.46080108	0.01386272
ENSG00000186767	329.7816857	-1.336935334	0.543346444	-2.460557805	0.013872122
ENSG00000260367	14.12591462	2.34505638	0.953060922	2.460552444	0.01387233
ENSG00000009830	1042.693428	-0.670318096	0.272452669	-2.460310258	0.013881695
ENSG00000133313	2624.32429	-0.756720461	0.307579651	-2.460242276	0.013884325
ENSG00000196132	362.4696153	-1.715149587	0.697163294	-2.460183435	0.013886602
ENSG00000151883	1305.651549	0.713562987	0.290049298	2.460143814	0.013888135
ENSG00000129691	1602.5601	-0.892454391	0.362778113	-2.460055772	0.013891543
ENSG00000169750	2725.378773	-1.219069597	0.495557011	-2.459998688	0.013893752
ENSG00000196821	5635.110058	0.826135361	0.33590683	2.459418171	0.013916242
ENSG00000005022	11033.26275	-0.861747597	0.350396698	-2.459348507	0.013918943
ENSG00000229164	4.143731853	5.521613207	2.245236764	2.459256545	0.013922509
ENSG00000148737	5160.195626	-1.035317851	0.420999185	-2.459192055	0.01392501
ENSG00000224295	10.19818285	2.751040585	1.118884126	2.458735915	0.013942714
ENSG00000110719	1119.888731	-1.098279531	0.44672694	-2.458503019	0.01395176
ENSG00000272068	1107.072337	1.183353196	0.481534115	2.457464923	0.013992147
ENSG00000237101	5.779923008	3.425018069	1.394322406	2.456403235	0.014033559
ENSG00000165716	211.0296182	-1.036585812	0.421998731	-2.456371869	0.014034784
ENSG00000198964	1330.649499	0.65152814	0.265252118	2.456259896	0.014039158
ENSG00000188706	2255.647452	0.702835508	0.286197442	2.455771453	0.014058253
ENSG00000149474	369.4573708	-1.295575319	0.527569299	-2.455744337	0.014059314
ENSG00000171174	68.62229743	1.170215003	0.476673365	2.454962014	0.014089948
ENSG00000152443	881.8006665	0.97674636	0.397980563	2.454256443	0.014117626

ENSG00000188315	793.3140016	0.913212283	0.372118757	2.454088289	0.01412423
ENSG00000271324	6.168788454	3.619057733	1.474850643	2.453846937	0.014133713
ENSG00000254093	367.9090532	-0.831005339	0.338668441	-2.453743069	0.014137795
ENSG00000259067	4.275190042	5.568040563	2.2693911	2.453539437	0.014145803
ENSG00000130383	5.340259081	3.416660654	1.392933638	2.452852427	0.014172847
ENSG00000135749	1459.822644	0.79935964	0.325993606	2.452071526	0.014203643
ENSG00000207425	81.83797422	1.242635268	0.506774413	2.452048163	0.014204565
ENSG00000073849	788.1505489	-1.330804141	0.542754982	-2.451942747	0.014208727
ENSG00000122390	114.0902872	1.221459336	0.498187624	2.45180586	0.014214133
ENSG00000169020	423.3543694	-0.827241056	0.337434798	-2.451558227	0.014223917
ENSG00000186265	15.84822902	3.021254874	1.232407534	2.451506333	0.014225968
ENSG00000077684	1280.427966	-0.899979356	0.36711529	-2.451489711	0.014226625
ENSG00000197540	20.51366213	1.819718752	0.742319906	2.451394253	0.014230399
ENSG00000228897	11.07800031	2.620304382	1.068938952	2.451313405	0.014233596
ENSG00000152022	866.154136	-0.660894319	0.269611192	-2.451286666	0.014234654
ENSG00000125650	377.5812117	1.220032926	0.497715523	2.451265571	0.014235488
ENSG00000010818	1904.302766	0.700633706	0.285880319	2.450793775	0.014254159
ENSG00000033327	569.4015171	0.975173325	0.398029008	2.450005664	0.014285397
ENSG00000086504	1216.539684	-0.712048498	0.290632305	-2.449997764	0.01428571
ENSG00000148985	713.1689635	-0.807708251	0.329690994	-2.449894795	0.014289796
ENSG00000174547	2165.745766	-0.922826994	0.376709848	-2.449702335	0.014297436
ENSG00000198171	1855.175866	1.099444403	0.448838337	2.44953319	0.014304153
ENSG00000198911	6833.14712	-1.137335879	0.464328955	-2.449418383	0.014308713
ENSG00000076706	1617.004215	-1.227248624	0.501109231	-2.449064092	0.014322796
ENSG00000124091	29.77234028	2.068503293	0.844670473	2.448887892	0.014329804
ENSG00000143862	1920.604365	0.774253924	0.316233335	2.44836277	0.014350709
ENSG00000116679	3214.347993	-1.274802685	0.520689735	-2.448296171	0.014353362
ENSG00000006194	2019.444414	0.827107265	0.3379001	2.447786385	0.014373685
ENSG00000099998	10.73378912	-3.856338662	1.576081611	-2.446788692	0.014413531
ENSG00000231868	15.98696563	-1.774388791	0.725666312	-2.445185564	0.014477763
ENSG00000122733	30.03265738	1.380461895	0.564768467	2.444297044	0.014513471
ENSG00000154710	121.8966062	1.056589654	0.432271748	2.444271824	0.014514486
ENSG00000270589	45.70433289	1.22878788	0.50273141	2.444223407	0.014516434
ENSG00000132952	1718.619533	0.736202678	0.301295888	2.443454115	0.014547419
ENSG00000103260	1500.195519	-1.329280442	0.544038841	-2.443355772	0.014551385
ENSG00000145220	1767.584687	-1.067423928	0.436868575	-2.443352505	0.014551516
ENSG00000188039	61.98717092	1.031566627	0.422265008	2.442936562	0.014568298
ENSG00000116745	215.2897059	-1.240737498	0.507925711	-2.442753874	0.014575674
ENSG00000122203	5730.439847	0.824054712	0.337463429	2.441908195	0.014609861
ENSG00000141428	817.2413998	0.870721075	0.356635334	2.441488524	0.014626853
ENSG00000243015	60.76369427	-1.135817301	0.465304849	-2.441017547	0.014645943
ENSG00000267430	5.724945685	3.993425252	1.6360455	2.440901094	0.014650667
ENSG00000138600	1350.31404	0.844458477	0.345966286	2.44086927	0.014651958
ENSG00000103707	912.2968269	-0.778830136	0.319088512	-2.44079654	0.014654909
ENSG00000173320	845.3843263	0.674326211	0.276324948	2.440337785	0.014673534
ENSG00000103249	1153.363388	-0.793089068	0.324999156	-2.440280389	0.014675866
ENSG00000153044	538.0839938	-1.238598146	0.507585311	-2.440177282	0.014680056

ENSG00000164327	2523.985851	0.73947332	0.303071143	2.439933117	0.014689981
ENSG00000239332	28.47635071	-1.453087122	0.595563952	-2.439850696	0.014693333
ENSG00000232888	103.4235574	1.761692509	0.722089528	2.439714802	0.014698861
ENSG00000026036	139.0752979	-0.952292149	0.390336146	-2.439671958	0.014700604
ENSG00000182979	3417.72993	-0.937635551	0.384353876	-2.439511108	0.014707151
ENSG00000113569	3531.732163	-0.989045409	0.405600314	-2.43847299	0.014749461
ENSG00000224837	22.08315386	-2.269917943	0.931076891	-2.437948966	0.014770859
ENSG00000240871	3.000311274	-5.020621781	2.05954937	-2.437728298	0.014779878
ENSG00000133612	2578.351513	-0.777104125	0.318863078	-2.437109153	0.01480521
ENSG00000153037	551.2886458	0.703642278	0.288724872	2.437068457	0.014806876
ENSG00000253797	1186.856337	0.797657934	0.327309471	2.437014522	0.014809085
ENSG00000086200	1422.594812	-0.912392752	0.374407084	-2.436900347	0.014813761
ENSG00000156413	11.16632269	2.88669599	1.184588006	2.436877612	0.014814693
ENSG00000264125	4.43225866	4.610427827	1.892109593	2.436660035	0.014823608
ENSG00000242802	902.6130893	1.059086606	0.434720258	2.436248568	0.014840483
ENSG00000122566	46278.13605	-0.934327937	0.383519217	-2.436195878	0.014842645
ENSG00000157823	874.4877279	0.742942979	0.304967485	2.436138329	0.014845006
ENSG00000219249	5.514404571	-4.889319781	2.007135077	-2.435969476	0.014851938
ENSG00000157927	113.3119859	-0.981509639	0.402940083	-2.435869947	0.014856025
ENSG00000111348	51.0659422	-1.987512081	0.81605954	-2.435498863	0.014871271
ENSG00000139684	4006.614413	-0.831622875	0.341494164	-2.435247694	0.014881599
ENSG00000107872	440.7123192	-0.793034576	0.325672868	-2.435064919	0.014889118
ENSG00000260349	254.104374	0.829288908	0.340570081	2.435002235	0.014891698
ENSG00000128833	1672.518453	-0.677012008	0.278095661	-2.434457289	0.01491414
ENSG00000076650	700.9662907	1.010310964	0.415026097	2.434331171	0.014919338
ENSG00000239732	7.099594164	3.988225527	1.638413946	2.434198963	0.014924789
ENSG00000107331	4536.6342	-1.378837136	0.566466107	-2.434103504	0.014928726
ENSG00000069011	705.9202379	-0.700597306	0.287831363	-2.434054783	0.014930735
ENSG00000203734	27.84735866	1.638218889	0.67318225	2.433544391	0.014951802
ENSG00000146232	717.0919121	0.790474704	0.324834027	2.433472601	0.014954767
ENSG00000258424	24.5873394	1.937843414	0.796329668	2.433468816	0.014954924
ENSG00000169894	734.0009168	-1.144433608	0.47035603	-2.433122007	0.014969256
ENSG00000270010	9.42361524	-2.549863932	1.048074048	-2.432904371	0.014978256
ENSG00000198857	42.94541816	1.887571034	0.7759786	2.432503982	0.014994827
ENSG00000133315	715.7505259	-1.34137499	0.551485777	-2.432292989	0.015003565
ENSG00000234699	11.7692059	-2.160762986	0.888444969	-2.43207296	0.015012683
ENSG00000127589	56.51274798	-2.017052912	0.829457942	-2.431772378	0.015025146
ENSG00000169919	1011.123361	-0.888547305	0.365462115	-2.43129799	0.015044835
ENSG00000167130	1605.528244	1.22105861	0.50223623	2.431243579	0.015047095
ENSG00000255517	73.47891698	1.149624499	0.472955428	2.430724823	0.015068654
ENSG00000235999	226.1240362	1.099326611	0.452283482	2.430614105	0.015073259
ENSG00000174373	1912.771918	0.742325736	0.305470399	2.430106937	0.015094369
ENSG00000075945	1118.593635	0.931179414	0.38322689	2.429838401	0.015105556
ENSG00000167693	3300.086801	-0.893135181	0.367671843	-2.429163935	0.015133688
ENSG00000015153	726.6954453	0.903390097	0.371963747	2.428704688	0.015152869
ENSG00000241749	51.78790284	-1.783114689	0.734364128	-2.428107013	0.015177864
ENSG00000169375	3432.78189	-0.662357032	0.272787486	-2.428106363	0.015177891

ENSG00000137842	506.937532	0.931612261	0.383730472	2.427777645	0.015191654
ENSG00000136444	2615.843431	-1.484438172	0.611546047	-2.427353065	0.015209446
ENSG00000173511	489.7398763	-1.350260904	0.556324864	-2.427108675	0.015219696
ENSG00000083838	219.3666022	1.045951407	0.4309562	2.427048056	0.015222239
ENSG00000166226	13271.69998	-0.694633285	0.28625531	-2.426621482	0.015240147
ENSG00000129347	1639.804219	-1.022017481	0.421177411	-2.426572401	0.015242209
ENSG00000128185	708.4244896	-0.72206996	0.297581582	-2.426460521	0.015246909
ENSG00000077348	600.1414144	-1.536249908	0.633370168	-2.425516681	0.015286613
ENSG00000250959	78.90449101	1.235477242	0.509485538	2.424950563	0.015310471
ENSG00000141401	571.8304473	-1.525515273	0.629109696	-2.424879609	0.015313464
ENSG00000168502	1753.242795	-1.263138489	0.520976564	-2.424559138	0.015326987
ENSG00000103832	8.953429929	-2.938793699	1.212241948	-2.424263327	0.015339478
ENSG00000122877	540.5017001	1.546182476	0.637823756	2.424153165	0.015344132
ENSG00000148341	2084.049905	-0.711650251	0.29356718	-2.424147862	0.015344356
ENSG00000225830	919.2584685	0.825879104	0.340693597	2.424111025	0.015345913
ENSG00000173258	293.1281925	1.013035182	0.418020562	2.423409934	0.015375565
ENSG00000135637	476.0570384	1.203442481	0.496679396	2.422976454	0.015393924
ENSG00000039600	52.69551027	1.832642492	0.75650309	2.422518184	0.015413354
ENSG00000145780	2362.278638	0.764743032	0.315691602	2.422437049	0.015416797
ENSG00000206538	38.51021845	2.039674668	0.842016567	2.422368807	0.015419692
ENSG00000056972	900.6403369	0.723539372	0.298695912	2.422327667	0.015421438
ENSG00000251168	6.43287597	3.619694371	1.494761518	2.42158654	0.015452921
ENSG00000262921	5.638208213	-3.368837785	1.391263846	-2.421422647	0.015459891
ENSG00000161643	18.75556941	1.908691963	0.788309444	2.421247112	0.015467359
ENSG00000206384	7.411395232	2.956910723	1.221320645	2.421076509	0.01547462
ENSG00000166569	9.809795495	3.37978609	1.39644763	2.420274143	0.01550881
ENSG00000159335	7699.52241	-0.995236518	0.411327201	-2.419573797	0.015538707
ENSG00000105185	1612.635349	-0.91810109	0.379593921	-2.418640128	0.015578644
ENSG00000107874	2244.288029	-0.770868441	0.318729563	-2.418565866	0.015581824
ENSG00000109919	3214.63255	-0.693865584	0.286910926	-2.418400697	0.0155889
ENSG00000198060	3404.128843	0.672665879	0.278170124	2.41818161	0.01559829
ENSG00000176095	2215.610151	0.954459317	0.394749232	2.41788771	0.015610893
ENSG00000222493	7.560190168	-2.54234713	1.051546041	-2.417723077	0.015617958
ENSG00000138785	806.629627	0.766072876	0.316890456	2.417469068	0.015628862
ENSG00000163811	4843.373658	-0.864179824	0.357564075	-2.416853048	0.015655336
ENSG00000124209	2374.920332	0.914311828	0.378406535	2.416215749	0.015682766
ENSG00000136932	351.0405572	0.775427928	0.320993371	2.415713215	0.015704425
ENSG00000077942	675.6855187	-1.14266503	0.473110513	-2.415218004	0.015725794
ENSG00000001617	995.2546684	-0.941188334	0.389726276	-2.414998403	0.015735279
ENSG00000119431	951.026113	0.838699844	0.347373413	2.414404248	0.015760965
ENSG00000100003	640.6476658	0.731559027	0.302998898	2.41439501	0.015761365
ENSG00000227199	4.098267038	-4.467274507	1.850316097	-2.414330457	0.015764158
ENSG00000215009	102.1562849	1.027954813	0.425810283	2.41411458	0.015773501
ENSG00000213684	4.075811044	-4.451136918	1.844199975	-2.413586909	0.01579636
ENSG00000240230	997.7088748	1.025444641	0.424866507	2.413569027	0.015797135
ENSG00000254477	6.487730743	3.213642255	1.331724626	2.413143222	0.015815603
ENSG00000105426	4270.853578	-1.039733882	0.431045338	-2.412121857	0.01585998

ENSG00000162572	125.2388451	-1.413843975	0.586348838	-2.411267634	0.015897178
ENSG00000124224	475.8447353	0.975709597	0.404648584	2.41125173	0.015897871
ENSG00000107281	2077.818574	-1.017758471	0.422096367	-2.411199314	0.015900157
ENSG00000196459	877.3271264	0.820834599	0.340466551	2.410911136	0.015912725
ENSG00000110455	204.1620611	-1.162351379	0.482224983	-2.410392287	0.015935376
ENSG00000226067	37.1499991	1.379499184	0.572439859	2.409858716	0.015958699
ENSG00000181163	50280.18947	-0.837795793	0.347664674	-2.409781197	0.01596209
ENSG00000234689	2.762447052	4.938729532	2.049901252	2.40925241	0.015985238
ENSG00000250072	9.833992237	-2.90599791	1.206305814	-2.409005972	0.015996036
ENSG00000110344	4128.685634	0.835014654	0.346627048	2.408971423	0.015997551
ENSG00000244002	3.970551941	5.45960641	2.266421907	2.408910006	0.016000243
ENSG00000196611	24.32367453	1.964402282	0.815517479	2.408780108	0.016005938
ENSG00000141084	2398.789612	1.065633143	0.442397074	2.408770776	0.016006348
ENSG00000251429	45.0009601	1.189015179	0.493699087	2.408380348	0.016023478
ENSG00000133961	3361.43157	0.826750381	0.343359918	2.40782438	0.016047899
ENSG00000236047	6.699562026	4.160663449	1.728007158	2.407781374	0.016049789
ENSG00000112200	2871.485695	0.836001735	0.347219406	2.407704524	0.016053168
ENSG00000169684	259.9083625	-0.832122161	0.345707481	-2.407012306	0.016083628
ENSG00000143224	319.4792785	-1.468235706	0.609997775	-2.406952559	0.016086259
ENSG00000203814	32.70755415	1.717067649	0.713435564	2.406759259	0.016094776
ENSG00000180694	2140.64699	-0.759300698	0.315507365	-2.406602133	0.016101701
ENSG00000223396	133.7476941	0.841483831	0.34982596	2.405435636	0.016153197
ENSG00000142632	1406.092344	-1.029006415	0.427804363	-2.405320055	0.016158307
ENSG00000109743	37.24903594	1.816291327	0.755173218	2.405132072	0.016166622
ENSG00000251180	23.40292376	-1.650488485	0.686327467	-2.404811936	0.01618079
ENSG00000147130	1563.768752	-1.271950944	0.528926793	-2.404776921	0.016182341
ENSG00000257949	10.95692136	2.268065728	0.943193775	2.40466571	0.016187265
ENSG00000162620	36.59991184	-1.335393887	0.555366672	-2.404526511	0.016193432
ENSG00000189007	870.6077814	-1.14097029	0.474530264	-2.404420488	0.01619813
ENSG00000244560	159.6265727	-1.201883607	0.499947631	-2.404019005	0.01621593
ENSG00000117707	3.62837725	5.331519965	2.21816863	2.403568374	0.016235931
ENSG00000137440	116.8259929	-1.452583599	0.604483623	-2.403015637	0.016260492
ENSG00000171314	5210.28113	-0.857875637	0.357015561	-2.402908248	0.016265268
ENSG00000214212	21.98643812	1.884283047	0.784189256	2.402842213	0.016268205
ENSG00000232139	4.001843234	5.470522473	2.277092545	2.402415521	0.016287196
ENSG00000230778	8.390962188	-3.030005698	1.261413705	-2.402071332	0.016302529
ENSG00000115109	1568.929182	0.695147227	0.289408008	2.401962659	0.016307373
ENSG00000161202	4677.710194	0.892574745	0.371625682	2.401811253	0.016314124
ENSG00000103550	503.3477101	-1.422734256	0.592492969	-2.401267747	0.016338377
ENSG00000122257	5126.486954	0.844956559	0.351920926	2.400984131	0.016351046
ENSG00000229180	192.9160353	1.10986193	0.462259025	2.400952432	0.016352462
ENSG00000130638	2675.550309	-0.853408563	0.355514039	-2.400491876	0.016373054
ENSG00000060656	2974.422353	-1.251192115	0.52128463	-2.400209107	0.016385709
ENSG00000087303	58.02917896	1.676442044	0.698577818	2.399792837	0.016404353
ENSG00000165806	2282.861534	0.690664022	0.287894263	2.399019749	0.016439028
ENSG00000258881	121.0394348	1.274768781	0.53155278	2.398197939	0.016475959
ENSG00000114331	4716.697454	0.802988551	0.334851167	2.398046149	0.016482788

ENSG00000100364	3101.050485	-0.870593615	0.363060401	-2.397930515	0.016487993
ENSG00000267010	124.4341471	1.01648191	0.423941079	2.397696192	0.016498543
ENSG00000261559	4.376422102	-4.53586114	1.891836727	-2.397596513	0.016503033
ENSG00000170946	1070.509841	0.733468329	0.305920404	2.397578978	0.016503823
ENSG00000183150	30.25988797	-1.760581218	0.734360505	-2.39743451	0.016510332
ENSG00000228626	14.00295941	2.034716929	0.84872756	2.397373464	0.016513083
ENSG00000059769	663.95802	0.932768033	0.389270666	2.396193994	0.01656632
ENSG00000132906	538.373849	0.702019769	0.292981406	2.396123972	0.016569485
ENSG00000153790	131.6026108	1.088470546	0.454285227	2.396006915	0.016574778
ENSG00000085491	1809.571277	-0.864174472	0.360690611	-2.395888461	0.016580135
ENSG00000172159	151.8143516	1.259454013	0.525798254	2.395317981	0.016605957
ENSG00000215256	318.9183041	-1.100930183	0.459640561	-2.395198068	0.016611389
ENSG00000167536	122.7789984	-1.106504279	0.46199908	-2.395035676	0.016618748
ENSG00000134324	2381.050472	0.879694248	0.367441319	2.394108125	0.016660836
ENSG00000251093	2.685187628	4.907323132	2.049821105	2.394025078	0.016664609
ENSG00000196812	224.5366633	1.05117047	0.439097671	2.393933151	0.016668787
ENSG00000250762	6.586948727	4.128126626	1.724671445	2.393572781	0.016685171
ENSG00000095932	3.95862578	4.442873903	1.856464375	2.393191037	0.016702542
ENSG00000222011	113.5097198	-0.870353941	0.363780219	-2.392526851	0.016732804
ENSG00000264644	5.987133148	3.596983766	1.503457604	2.392474358	0.016735197
ENSG00000065621	3052.366782	-0.770156819	0.32192031	-2.392383443	0.016739344
ENSG00000174374	778.4445409	1.189790728	0.497371017	2.392159349	0.016749569
ENSG00000161847	1323.65482	-0.681518542	0.284905816	-2.39208364	0.016753024
ENSG00000067082	5521.003658	-1.144647954	0.478520023	-2.392058638	0.016754165
ENSG00000140153	1279.945704	0.950987086	0.397636833	2.391597073	0.016775247
ENSG00000270172	507.219054	-1.424784251	0.595750834	-2.391577433	0.016776145
ENSG00000196268	108.7938142	1.391925881	0.582040531	2.391458684	0.016781573
ENSG00000267432	52.02921584	-1.893635694	0.792064758	-2.390758678	0.0168136
ENSG00000235162	1542.546428	-0.740972474	0.309956524	-2.390569054	0.016822285
ENSG00000133069	539.5386761	0.792505869	0.331536011	2.390406599	0.016829729
ENSG00000162645	50.83105278	2.137465184	0.894214677	2.390326662	0.016833393
ENSG00000151657	814.199172	0.800284497	0.334807824	2.39028015	0.016835525
ENSG00000262678	20.29384446	-1.68397223	0.704685057	-2.389680627	0.01686303
ENSG00000149328	1389.814801	-1.410786426	0.590383331	-2.389610873	0.016866232
ENSG00000083807	210.3899381	-0.918729983	0.384491159	-2.389469723	0.016872714
ENSG00000213707	16.92789427	-1.694441209	0.709150071	-2.389397222	0.016876045
ENSG00000124788	2372.147909	0.814165387	0.34078219	2.389107794	0.016889346
ENSG00000168924	3593.495116	-0.738015081	0.30893343	-2.388912982	0.016898304
ENSG00000186566	3810.540917	0.796684721	0.333583461	2.388262055	0.016928266
ENSG00000165494	3176.617407	0.854416048	0.357827279	2.38778902	0.016950069
ENSG00000168476	1613.723581	-0.951791234	0.398700308	-2.38723476	0.016975647
ENSG00000254681	404.5909101	-0.948449663	0.397324421	-2.387091283	0.016982274
ENSG00000200674	2.664326766	4.897333266	2.051648333	2.387023735	0.016985395
ENSG00000197622	3368.978257	0.806278121	0.337813891	2.38675242	0.016997934
ENSG00000271529	681.4892263	0.992605782	0.415901256	2.386638094	0.01700322
ENSG00000204524	350.0101149	0.88469205	0.370697982	2.386557501	0.017006948
ENSG00000163950	3839.201607	-0.770875608	0.323019163	-2.386470207	0.017010986

ENSG00000259366	93.09416332	1.05196904	0.440896792	2.38597572	0.017033876
ENSG00000204070	988.0210955	0.806326742	0.337963096	2.385842571	0.017040044
ENSG00000206172	2.713904956	-4.878807068	2.044950737	-2.3857822	0.017042841
ENSG00000154328	1558.370619	-0.745419851	0.312448813	-2.385734303	0.017045061
ENSG00000008441	1237.002308	-1.077175935	0.451559256	-2.385458654	0.01705784
ENSG00000105784	439.7703501	0.782842129	0.328185195	2.385366985	0.017062091
ENSG00000213152	4.323138169	4.550598113	1.908095779	2.384889775	0.017084239
ENSG00000230424	9.581877453	2.601835068	1.091343686	2.384065718	0.017122544
ENSG00000160325	505.3581868	0.852924268	0.357829928	2.383602379	0.017144115
ENSG00000135740	384.8926284	-0.859991328	0.360798153	-2.383580186	0.017145149
ENSG00000237575	25.02051872	1.874464833	0.786414243	2.383559111	0.017146131
ENSG00000086189	1748.371311	-0.821431516	0.344697173	-2.383052661	0.017169738
ENSG00000043143	942.8527023	-1.231135056	0.516660027	-2.382872668	0.017178135
ENSG00000077721	3986.971626	1.075554604	0.451410655	2.38265223	0.017188424
ENSG00000236305	11.90586582	-2.591720242	1.087748976	-2.382645537	0.017188736
ENSG00000133895	2574.907528	-0.650091001	0.272848524	-2.382607725	0.017190502
ENSG00000252743	14.54992302	-2.680289429	1.125138151	-2.382186959	0.017210157
ENSG00000260252	9.115769914	3.078205928	1.29242822	2.381722931	0.017231857
ENSG00000106686	35.25753509	1.334656401	0.560426248	2.381502305	0.017242183
ENSG00000009780	393.5115813	-1.092236371	0.458636865	-2.38148403	0.017243038
ENSG00000169905	10379.35067	0.863529037	0.362612849	2.381407719	0.017246611
ENSG00000171208	555.3869371	-1.000118824	0.420012856	-2.38116241	0.017258101
ENSG00000178695	253.1921803	-1.46228196	0.614143835	-2.381008939	0.017265293
ENSG00000198205	253.92552	0.855259756	0.359225756	2.38084197	0.01727312
ENSG00000124920	2526.315998	-1.123561252	0.471952718	-2.38066486	0.017281426
ENSG00000260404	710.7526196	1.005628199	0.42244248	2.380509172	0.017288731
ENSG00000101442	916.0288142	-0.969301412	0.407204691	-2.380378799	0.017294849
ENSG00000169302	91.52448917	1.313095029	0.551644759	2.380327206	0.017297271
ENSG00000173039	4659.152896	0.779310123	0.327399939	2.380300143	0.017298542
ENSG00000110090	4763.2494	-0.78196561	0.328566897	-2.379928159	0.017316014
ENSG00000182149	7021.033213	0.720701108	0.302845589	2.379764254	0.017323717
ENSG00000269752	3.098027848	-5.064954968	2.128381963	-2.379720865	0.017325757
ENSG00000224376	63.5286504	1.421284823	0.597261352	2.379669835	0.017328156
ENSG00000213096	259.3843929	0.905842004	0.380715824	2.379312721	0.017344954
ENSG00000205309	160.3611844	-1.560795118	0.656008816	-2.379228878	0.0173489
ENSG00000116668	364.8423096	0.825621734	0.347019579	2.37917911	0.017351243
ENSG00000214796	94.92803377	-1.202484185	0.505465801	-2.378962499	0.017361442
ENSG00000164078	1838.661206	-0.784994455	0.329984939	-2.378879644	0.017365345
ENSG00000234750	20.97062941	2.680866252	1.127076926	2.378600953	0.017378478
ENSG00000147434	7.297108057	3.031514485	1.274627734	2.378352835	0.017390177
ENSG00000198488	4.348815213	4.584878831	1.927968174	2.378088443	0.017402651
ENSG00000145692	5.550270043	3.392010301	1.426445836	2.377945391	0.017409404
ENSG00000229962	3.108837514	5.105882248	2.147240488	2.377880949	0.017412447
ENSG00000198208	289.9358548	-0.998352822	0.419925237	-2.377453733	0.01743263
ENSG00000272911	3.788847103	4.358268658	1.833215626	2.377390087	0.017435638
ENSG00000163347	20.13597592	1.604673183	0.674981816	2.377357648	0.017437172
ENSG00000099810	6426.090111	-1.522001407	0.640249705	-2.377199701	0.017444641

ENSG00000205534	275.5043566	0.726237471	0.305511496	2.377119947	0.017448413
ENSG00000182324	201.2005021	0.933148756	0.392574308	2.376999046	0.017454133
ENSG00000129204	39.8632132	1.68239574	0.707926745	2.376511061	0.017477237
ENSG00000172262	2384.776366	0.645093892	0.271483473	2.376181083	0.017492876
ENSG00000182919	1629.171915	0.672826946	0.283165107	2.376094125	0.017496999
ENSG00000115282	807.722217	-1.010404483	0.425340562	-2.375518754	0.017524302
ENSG00000162999	79.86156079	-1.143542217	0.481431031	-2.375298106	0.017534782
ENSG00000167619	150.5065743	-1.422787702	0.599053148	-2.37506089	0.017546055
ENSG00000226396	42.73648904	1.385685785	0.583605456	2.374353721	0.0175797
ENSG00000154473	7682.024337	-0.870391862	0.366584684	-2.374326859	0.017580979
ENSG00000127884	1471.169253	-0.858625702	0.361630498	-2.374317729	0.017581414
ENSG00000233154	4.906473058	3.981666515	1.67725264	2.373921746	0.01760028
ENSG00000096070	2613.602829	1.058469745	0.445908352	2.373738325	0.017609025
ENSG00000188306	3.918399787	5.441650871	2.292722493	2.373445058	0.017623015
ENSG00000130475	839.8648335	-1.224273118	0.51588463	-2.373152921	0.01763696
ENSG00000267504	3.682404272	4.32486623	1.822534213	2.372995909	0.017644459
ENSG00000152642	1285.433586	-1.352539943	0.569984467	-2.372941758	0.017647046
ENSG00000129911	1586.461471	-0.918516326	0.387093085	-2.372856455	0.017651122
ENSG00000261101	21.88131008	1.731186012	0.729681572	2.372522588	0.017667083
ENSG00000104852	8519.220544	-0.586208936	0.247086241	-2.372487167	0.017668777
ENSG00000108641	306.2962985	-0.887073092	0.373913836	-2.372399754	0.017672958
ENSG00000170315	16617.27014	0.96027242	0.404771993	2.372378612	0.01767397
ENSG00000163935	1637.458986	0.935404625	0.394367405	2.371911604	0.017696324
ENSG00000169967	3381.096072	0.631136307	0.266102918	2.371775219	0.017702857
ENSG00000188175	4.021208366	4.464785315	1.882908914	2.371216835	0.017729626
ENSG00000145088	195.6587989	0.904855655	0.381647552	2.370919581	0.017743891
ENSG00000006837	55.13988569	1.255477803	0.529613305	2.370555631	0.017761371
ENSG00000115944	4178.991935	0.710751448	0.299826998	2.370538517	0.017762193
ENSG00000120690	2145.430706	0.889202316	0.375110317	2.370508822	0.01776362
ENSG00000168143	581.9222046	0.823917763	0.347641362	2.37002225	0.017787015
ENSG00000214733	21.98002241	-2.303647251	0.972008285	-2.369987259	0.017788698
ENSG00000175854	731.1486535	0.856275198	0.361359684	2.369592503	0.0178077
ENSG00000165935	14.40799096	-1.729270083	0.729778058	-2.369583553	0.017808131
ENSG00000012048	1539.354658	-1.13893407	0.480704079	-2.369303944	0.017821601
ENSG00000172014	27.45267361	-1.592859319	0.672329982	-2.369163002	0.017828394
ENSG00000164331	685.1583836	0.657633423	0.277589352	2.36908735	0.017832041
ENSG00000057019	30975.1998	-1.054101359	0.444953248	-2.369015987	0.017835483
ENSG00000104894	350.3891283	-1.364215163	0.575863814	-2.368989212	0.017836774
ENSG00000135999	1182.203036	0.805278117	0.339965829	2.36870311	0.017850576
ENSG00000130803	2803.647278	0.853273951	0.360246851	2.36858129	0.017856456
ENSG00000124575	4.033134527	5.48164979	2.314506246	2.36838842	0.017865768
ENSG00000165810	382.6949665	-1.174161138	0.495901158	-2.367732196	0.017897486
ENSG00000132912	5853.702414	0.836787651	0.353451288	2.367476597	0.017909853
ENSG00000101955	653.5136844	-1.353304302	0.571705567	-2.367135077	0.017926389
ENSG00000263818	9.052406393	-2.767738681	1.169242606	-2.36712096	0.017927073
ENSG00000117601	21.80630382	2.523378089	1.066126802	2.366864883	0.017939481
ENSG00000149476	1623.523587	-1.261436292	0.532982153	-2.366751468	0.017944979

ENSG00000255397	4.221389512	-4.479660944	1.893032515	-2.366394084	0.017962313
ENSG00000063241	967.5183024	-1.182778053	0.499928051	-2.365896556	0.017986469
ENSG00000163817	66.37725423	-1.58938907	0.671821384	-2.365791126	0.017991592
ENSG00000207280	8.942565989	-2.702185858	1.142269726	-2.365628534	0.017999494
ENSG00000129480	471.9845197	-0.854497386	0.361214398	-2.365623825	0.017999723
ENSG00000011332	307.4717107	-0.749987818	0.317064963	-2.365407426	0.018010245
ENSG00000166793	46.4697232	1.239395076	0.52399905	2.365262067	0.018017316
ENSG00000108839	76.14664309	1.417203167	0.599476679	2.364067221	0.018075532
ENSG00000167280	1781.411572	-0.618601021	0.26167153	-2.364036396	0.018077037
ENSG00000099290	430.6620681	0.856525129	0.362344315	2.363843156	0.018086468
ENSG00000173207	2189.364136	-1.088819067	0.460763542	-2.363075565	0.018123974
ENSG00000106853	2219.444019	0.738173305	0.312392905	2.362964373	0.018129412
ENSG00000146826	853.8771726	1.018338112	0.430988496	2.362796507	0.018137626
ENSG00000116127	1232.178321	-0.825864682	0.349537349	-2.362736582	0.018140559
ENSG00000158710	4933.866691	-0.981309564	0.415356395	-2.362572422	0.018148595
ENSG00000117013	298.2899949	-1.281783086	0.542542881	-2.362547056	0.018149837
ENSG00000213588	706.316953	-0.974558062	0.412665083	-2.361619875	0.018195288
ENSG00000155984	183.9366427	0.8050835	0.340945736	2.361324445	0.018209791
ENSG00000196091	11.57911715	3.165006558	1.340627068	2.360840411	0.018233575
ENSG00000251127	25.31749336	1.449365981	0.614011872	2.36048527	0.018251043
ENSG00000136997	8063.594538	-1.446460476	0.612786753	-2.360463031	0.018252137
ENSG00000165632	1071.681511	0.799974926	0.338911506	2.360424214	0.018254048
ENSG00000074054	3201.449636	0.835298131	0.353892372	2.360316856	0.018259332
ENSG00000226598	5.037349729	4.803351732	2.03523443	2.360097521	0.018270131
ENSG00000140199	1133.716866	0.748297292	0.31706224	2.360095897	0.018270211
ENSG00000125966	51.40203162	1.182683453	0.501119914	2.36008073	0.018270958
ENSG00000178187	6.494771681	2.87074921	1.216682215	2.359489747	0.018300087
ENSG00000181555	4509.280813	0.805008787	0.341189292	2.35941985	0.018303534
ENSG00000155008	330.2526119	0.920464507	0.390187624	2.359030503	0.01832275
ENSG00000204922	269.8944986	-0.76106518	0.322651794	-2.358781804	0.018335033
ENSG00000157353	585.0254883	-0.795795193	0.337376707	-2.358773372	0.018335449
ENSG00000102241	3120.752311	-0.748860815	0.317513592	-2.358515776	0.01834818
ENSG00000123473	1999.252945	-1.106392973	0.469108363	-2.358501918	0.018348865
ENSG00000225886	15.65843562	2.95572683	1.253955287	2.357122985	0.018417146
ENSG00000067445	9.324130104	-2.663658156	1.130076516	-2.357060002	0.01842027
ENSG00000264968	7.737347211	2.66839382	1.132251056	2.356715684	0.018437357
ENSG00000126804	1672.82064	0.836878222	0.355184386	2.356179648	0.018463986
ENSG00000213047	629.6855911	0.672679437	0.285500049	2.356144736	0.018465721
ENSG00000174173	1630.525994	-0.606139872	0.257271093	-2.356035668	0.018471144
ENSG00000056736	382.6648275	-1.226712955	0.520691288	-2.355931401	0.018476329
ENSG00000105058	3347.735907	0.834056483	0.354062316	2.355677079	0.018488982
ENSG00000261801	299.3817566	-0.994403124	0.422187929	-2.3553566	0.018504938
ENSG00000253305	2.966948894	5.036274784	2.138321403	2.355246866	0.018510404
ENSG00000169902	1106.21768	0.872802082	0.370593503	2.355146742	0.018515392
ENSG00000067066	2406.556532	0.678445392	0.28809608	2.354927535	0.018526318
ENSG00000198399	2450.644653	0.733344445	0.311508911	2.354168435	0.018564197
ENSG00000267838	10.18111519	3.043061086	1.292783093	2.353883728	0.018578421

ENSG00000180316	16.52239384	2.096853898	0.890831546	2.353816394	0.018581787
ENSG00000175573	1298.717548	-1.190537383	0.505834254	-2.35361163	0.018592025
ENSG00000106404	822.3717061	0.908299683	0.385919265	2.35360026	0.018592593
ENSG00000162512	3259.350572	-1.084313639	0.460717105	-2.353534579	0.018595878
ENSG00000264584	2.977379325	5.04101964	2.141982491	2.353436436	0.018600788
ENSG00000163528	612.3003436	-0.819527943	0.348267788	-2.353154588	0.018614893
ENSG00000106809	3.155343864	-5.096227155	2.165988844	-2.352840907	0.018630603
ENSG00000234741	14799.39915	0.780088247	0.331555835	2.352811091	0.018632097
ENSG00000082641	13911.20782	0.864371684	0.367379831	2.352801137	0.018632596
ENSG00000022556	2400.43374	-0.658074062	0.279749908	-2.352365604	0.01865443
ENSG00000096433	7828.462803	-1.269075112	0.539495938	-2.352334879	0.018655971
ENSG00000271721	72.00623871	1.173927209	0.499051255	2.352317919	0.018656821
ENSG00000158458	34.65402295	-1.869999463	0.794969159	-2.352291838	0.01865813
ENSG00000079459	6173.423496	-0.628637079	0.267256915	-2.35218265	0.018663608
ENSG00000173145	2334.961039	-0.715847486	0.304338332	-2.352143688	0.018665563
ENSG00000207185	2.956518464	5.031584056	2.139172384	2.352117153	0.018666895
ENSG00000112773	2103.974167	0.66626036	0.283321296	2.351607062	0.01869251
ENSG00000161682	765.1826348	-0.898614655	0.382149391	-2.351474783	0.018699157
ENSG00000164466	5089.06738	-0.669687132	0.284796755	-2.351456331	0.018700084
ENSG00000171954	204.4109144	-0.988318271	0.420311767	-2.351393297	0.018703253
ENSG00000196083	428.1163759	0.969801073	0.412455173	2.351288419	0.018708526
ENSG00000165046	527.823007	1.353862293	0.575814713	2.351211702	0.018712384
ENSG00000131779	537.1193473	-0.935966322	0.398182154	-2.35059837	0.018743253
ENSG00000175832	2447.145331	-0.944249406	0.401719044	-2.350521891	0.018747105
ENSG00000205476	1043.199136	-0.827975255	0.352267828	-2.350414055	0.018752538
ENSG00000042088	932.5099475	-0.813435047	0.34612444	-2.350123115	0.018767202
ENSG00000269546	6.643764216	4.337283856	1.846063568	2.349476979	0.018799807
ENSG00000201616	8.600954539	2.449624971	1.042666604	2.349384704	0.018804467
ENSG00000138185	1134.009929	-0.920636869	0.391899674	-2.349164672	0.018815583
ENSG00000273365	21.46811273	2.558076432	1.088941575	2.349140203	0.01881682
ENSG00000013503	659.8340794	-0.684993627	0.291634192	-2.348811102	0.018833459
ENSG00000062725	3920.362484	0.791080094	0.336800759	2.348807337	0.018833649
ENSG00000107185	3117.06673	0.796341498	0.339062902	2.348654169	0.018841397
ENSG00000185220	577.9785979	0.827251239	0.352233481	2.34858775	0.018844758
ENSG00000159459	2795.275747	0.843869372	0.359367342	2.348208291	0.018863969
ENSG00000182963	3262.282523	-0.76236443	0.324679295	-2.348053731	0.018871799
ENSG00000222489	22.31967254	-1.411505615	0.601237398	-2.347667692	0.018891367
ENSG00000143257	31.8639191	1.489555456	0.634493779	2.347628151	0.018893373
ENSG00000260941	39.05148834	2.22422465	0.947549429	2.347344193	0.018907779
ENSG00000105053	2160.582366	1.125736144	0.479615593	2.347163352	0.018916959
ENSG00000259820	342.3551513	0.811204782	0.345657708	2.346844187	0.01893317
ENSG00000261428	35.50829975	-1.55081896	0.660822784	-2.346800076	0.018935412
ENSG00000054118	7625.783477	-0.710485071	0.302787959	-2.346477295	0.018951821
ENSG00000213013	2.987809756	5.045827074	2.150387283	2.34647364	0.018952007
ENSG00000164266	4.043564958	5.485449729	2.337908677	2.346306245	0.018960521
ENSG00000271576	44.92436037	-1.626148833	0.693075104	-2.346280834	0.018961814
ENSG00000168297	853.1952475	0.654911872	0.279131501	2.346248523	0.018963458

ENSG00000185947	1422.140646	1.101194634	0.469369313	2.346115531	0.018970226
ENSG00000177464	7.973599313	2.52650744	1.076908014	2.346075438	0.018972267
ENSG00000087299	718.6222113	-0.843340771	0.359562573	-2.345463163	0.019003456
ENSG00000076321	1000.824403	0.925343804	0.394530367	2.345431132	0.019005089
ENSG00000138772	3698.176889	-1.019467701	0.434718402	-2.345122028	0.019020853
ENSG00000177542	2158.01598	-0.774850078	0.330433435	-2.344950588	0.019029601
ENSG00000133059	1453.521764	0.721765034	0.307834554	2.344652425	0.019044824
ENSG00000132676	7664.982964	0.968021239	0.412931555	2.344265599	0.01906459
ENSG00000180769	159.2451054	0.811341233	0.346121406	2.344094355	0.019073346
ENSG00000134461	184.5247668	-1.170670666	0.499434465	-2.343992551	0.019078552
ENSG00000229298	139.5682428	-1.737868797	0.741431719	-2.343936404	0.019081425
ENSG00000259644	9.989287432	2.993586321	1.277287415	2.343706112	0.019093209
ENSG00000204710	53.28882342	-1.799256021	0.767738341	-2.343579739	0.019099679
ENSG00000174917	679.7452732	-0.869552124	0.371044401	-2.343525793	0.019102441
ENSG00000142227	1394.938298	-0.988450352	0.421924573	-2.342718142	0.019143839
ENSG00000175336	3.785445868	4.375408302	1.867666935	2.342713372	0.019144084
ENSG00000118260	2347.354493	0.597136186	0.254894882	2.342676246	0.019145989
ENSG00000136631	1775.175111	1.097218731	0.468364934	2.342657726	0.019146939
ENSG00000137463	16.43969116	1.806599353	0.771182588	2.342635041	0.019148103
ENSG00000233912	8.888065446	2.387774975	1.019276242	2.342618102	0.019148972
ENSG00000104522	2091.666247	-0.673352626	0.287444985	-2.342544355	0.019152757
ENSG00000117222	3097.061086	0.815102057	0.347960139	2.342515612	0.019154233
ENSG00000075413	4757.316098	0.786010018	0.335583924	2.342215948	0.01916962
ENSG00000225470	656.1817873	0.899155456	0.383910231	2.342098185	0.01917567
ENSG00000065357	1485.498231	-1.230548118	0.525502157	-2.341661404	0.019198123
ENSG00000147614	3.755650306	5.379703435	2.297603498	2.341441176	0.019209453
ENSG00000121753	764.1234325	-1.385669631	0.591816486	-2.341383965	0.019212397
ENSG00000155275	822.45849	0.890835359	0.380479995	2.341346117	0.019214345
ENSG00000140043	320.0072953	-0.965070022	0.412210711	-2.341205591	0.019221579
ENSG00000084070	1709.168518	0.68666249	0.293403462	2.340335334	0.019266432
ENSG00000142327	1338.383706	-1.000123809	0.427398543	-2.340026248	0.019282385
ENSG00000176454	1481.783248	-1.429826677	0.611042826	-2.339977849	0.019284884
ENSG00000155545	1960.863046	0.757746058	0.323874286	2.33963019	0.019302843
ENSG00000105643	1620.559357	0.766743061	0.327752197	2.33939869	0.019314809
ENSG00000236861	24.66206503	1.570310043	0.671319721	2.339138854	0.019328248
ENSG00000256616	16.41518377	-1.909762546	0.816560151	-2.338789793	0.019346315
ENSG00000132383	5410.725583	-0.754144337	0.322462917	-2.338700967	0.019350915
ENSG00000121060	9597.887447	0.767679545	0.328348058	2.338005441	0.019386966
ENSG00000204392	1244.71282	-0.954860315	0.408506219	-2.337443768	0.019416122
ENSG00000140839	52.59132046	1.574123154	0.673483785	2.337284414	0.0194244
ENSG00000024048	2690.410216	0.862701649	0.369107507	2.337263895	0.019425467
ENSG00000267216	39.6228933	1.608254888	0.688161379	2.337031598	0.019437541
ENSG00000260757	56.46395796	1.763999234	0.754850226	2.336886408	0.019445091
ENSG00000079332	6249.806455	0.772367925	0.330597818	2.336276533	0.019476834
ENSG00000130244	731.1904116	-0.889518563	0.380770872	-2.336099285	0.019486068
ENSG00000186591	8621.571118	0.68633759	0.293825586	2.335867341	0.019498157
ENSG00000119599	426.3426532	-1.088215471	0.465884546	-2.335805042	0.019501405

ENSG00000158201	2616.046435	0.901565356	0.386038493	2.33542865	0.019521039
ENSG00000083812	455.4549706	0.921222694	0.394461802	2.335391385	0.019522984
ENSG00000183251	969.920831	0.810886727	0.347230368	2.335298986	0.019527807
ENSG00000173011	1203.427554	0.802327978	0.343674539	2.334557512	0.019566548
ENSG00000138293	9021.235277	0.786001765	0.336715622	2.334319274	0.01957901
ENSG00000107854	3042.510992	0.703496121	0.301414406	2.333983071	0.019596608
ENSG00000230736	14.10647311	2.045974744	0.876651831	2.333850989	0.019603526
ENSG00000197372	317.7942623	1.178074627	0.504784412	2.333817366	0.019605287
ENSG00000269235	5.56222055	3.578697395	1.533411358	2.333814326	0.019605446
ENSG00000264462	265.5088904	1.278127946	0.547702841	2.333615694	0.019615854
ENSG00000237438	457.2650577	1.034615965	0.443409696	2.333318315	0.019631445
ENSG00000269131	155.8347778	-0.882343681	0.378157158	-2.333272457	0.01963385
ENSG00000137513	845.1933102	-1.078588111	0.462284343	-2.333170322	0.019639208
ENSG00000100012	10.31003607	2.680681903	1.149032697	2.332990097	0.019648665
ENSG00000178057	586.9914696	-0.788645414	0.338091537	-2.332638732	0.019667114
ENSG00000113460	2166.629188	-0.802380172	0.343981052	-2.332628987	0.019667626
ENSG00000171425	1049.831246	0.671299091	0.287843637	2.332165818	0.01969197
ENSG00000172478	5.24770919	3.3060891	1.41769804	2.332012182	0.01970005
ENSG00000174574	6124.38147	0.63197303	0.271012849	2.331893241	0.019706308
ENSG00000174206	477.2270322	-1.261110407	0.540880404	-2.331588272	0.019722361
ENSG00000163635	2657.906339	0.727859652	0.312181865	2.331524457	0.019725722
ENSG00000162482	31.80773142	-1.467021034	0.629223374	-2.331478924	0.01972812
ENSG00000164093	499.4491214	0.791357362	0.339437049	2.33138181	0.019733236
ENSG00000213509	4.060116663	4.463154659	1.914396208	2.331364135	0.019734167
ENSG00000254862	3.828663322	5.40641373	2.31903916	2.331316272	0.019736689
ENSG00000253873	28.69013411	2.010875008	0.862669243	2.330991889	0.019753787
ENSG00000165338	411.0910024	0.912685159	0.391559153	2.330899819	0.019758643
ENSG00000111269	1499.490039	0.902423613	0.387158431	2.330889739	0.019759174
ENSG00000039560	2436.992143	-0.758103145	0.325306014	-2.330430768	0.019783395
ENSG00000267632	80.31190696	1.591862436	0.683208918	2.329979008	0.019807261
ENSG00000187730	456.2496744	-1.619677395	0.695179921	-2.329867919	0.019813133
ENSG00000091879	158.4503881	-1.236464212	0.530744079	-2.32968065	0.019823036
ENSG00000016864	1030.264801	-0.820059721	0.352082224	-2.329171043	0.019850006
ENSG00000165985	78.86126948	-1.17735604	0.505495245	-2.329113975	0.019853029
ENSG00000037965	51.60370518	-1.244332762	0.534350197	-2.328684015	0.019875812
ENSG00000172572	43.28908276	1.388028701	0.596126302	2.328413787	0.019890142
ENSG00000196466	201.2698545	1.086960287	0.467095236	2.327063528	0.019961884
ENSG00000138081	2518.251733	0.651062915	0.279850861	2.326463864	0.019993818

padj	Gene symbol
9.29E-27	HMOX1
7.68E-21	DDIT3
1.03E-19	ATF3
2.12E-18	CHAC1
3.23E-15	HERPUD1
8.64E-13	SPX
3.99E-11	DNAJB9
4.51E-11	OSGIN1
9.13E-11	LUCAT1
8.72E-10	TONSL
1.57E-09	CYP4F3
1.77E-09	SDCBP2
2.09E-09	SESN2
3.88E-09	SLC7A11-AS1
5.13E-09	MAP1LC3B
5.13E-09	CBX4
7.11E-09	NMRAL2P
1.39E-08	BEST1
4.55E-08	MCM4
4.64E-08	AC112777.1
5.52E-08	ZFAND2A
5.52E-08	PTPDC1
7.47E-08	FANCA
7.47E-08	TNFRSF10B
8.29E-08	EGR1
9.25E-08	BLM
9.25E-08	E2F2
9.64E-08	HRG
1.32E-07	NCAPH
1.32E-07	MIR616
1.32E-07	KIF18B
1.32E-07	NA
1.32E-07	SRXN1
1.41E-07	CDCA3
1.80E-07	PPP1R15A
1.83E-07	GTSE1
1.92E-07	LY9
1.92E-07	CENPF
1.92E-07	BTG1
1.92E-07	SPC25
1.92E-07	SPC24
1.92E-07	SQSTM1
3.02E-07	MXD1
3.48E-07	MCM2
4.51E-07	IFRD1
4.52E-07	SERPINB8

4.67E-07	SLC7A11
5.20E-07	NPIPB2
6.13E-07	CIT
6.28E-07	MCM6
7.41E-07	DHFR
1.08E-06	CDK1
1.08E-06	MKI67
1.13E-06	NA
1.13E-06	TOP2A
1.13E-06	E2F8
1.15E-06	STMN1
1.23E-06	ZNF367
1.27E-06	FEN1
1.37E-06	MCM7
1.47E-06	GADD45G
1.57E-06	RASSF6
1.57E-06	ESCO2
1.84E-06	LIPH
1.88E-06	TYMS
1.88E-06	ADM2
1.99E-06	ARC
2.14E-06	ISL2
2.15E-06	DSCC1
2.15E-06	HSPA1A
2.17E-06	DLGAP5
2.18E-06	MCM10
2.21E-06	ARHGAP33
2.45E-06	E2F1
2.60E-06	POLE2
2.72E-06	BUB1B
2.72E-06	GADD45B
2.72E-06	MAP1B
2.72E-06	PASK
2.82E-06	TRIB3
2.91E-06	AURKB
3.12E-06	ESPL1
3.40E-06	HSPA1B
3.48E-06	GADD45A
3.56E-06	BIRC5
3.72E-06	PKMYT1
3.83E-06	LOC729654
4.02E-06	SERTAD1
4.14E-06	SKA3
5.26E-06	EID3
5.84E-06	CSRNP1
6.11E-06	PBK
6.20E-06	SCEL

6.22E-06	HBEGF
6.27E-06	RRM2
6.39E-06	STK40
7.28E-06	ERCC6L
7.74E-06	HELLS
8.25E-06	AKR1B10
8.50E-06	KIF22
8.58E-06	CENPI
1.01E-05	RIOK3
1.01E-05	TCF19
1.01E-05	CDCA7
1.04E-05	TMEM97
1.08E-05	LMNB1
1.13E-05	NCAPD3
1.13E-05	GCLM
1.16E-05	TRIM16L
1.17E-05	SLC9A1
1.18E-05	WDHD1
1.18E-05	TRIP13
1.18E-05	DKK1
1.18E-05	ZNF222
1.24E-05	CLSPN
1.26E-05	SAPCD1
1.26E-05	PIMREG
1.26E-05	BARD1
1.38E-05	KCNK3
1.42E-05	JUN
1.42E-05	CD274
1.44E-05	MCM3
1.50E-05	ASPM
1.56E-05	NA
1.65E-05	NA
1.65E-05	SLC6A13
1.71E-05	MAD2L1
1.71E-05	KIF20A
1.81E-05	KIFC1
1.94E-05	CCNF
1.97E-05	ABHD4
1.97E-05	NBR2
2.02E-05	DTYMK
2.10E-05	C21orf58
2.25E-05	NEIL3
2.49E-05	HKDC1
2.61E-05	ARRDC3
2.74E-05	PLK1
2.87E-05	NA
2.87E-05	CMYA5

2.87E-05	CCNA2
2.93E-05	ZNF674
2.97E-05	ADAMTS16
3.07E-05	HJURP
3.30E-05	VRK1
3.30E-05	BUB1
3.35E-05	ADPRM
3.55E-05	KIF23
3.69E-05	ERN1
3.69E-05	ZWINT
3.70E-05	UNG
3.70E-05	MCM5
3.70E-05	DDX11
3.70E-05	UBE2C
3.78E-05	TELO2
3.91E-05	DNMT1
4.20E-05	TK1
4.23E-05	KIF2C
4.40E-05	RND1
4.49E-05	SEC24D
4.92E-05	FTL
5.05E-05	ARHGEF39
5.08E-05	RPSAP44
5.21E-05	TUBB
5.28E-05	FOXM1
5.52E-05	IL1A
5.52E-05	NA
5.54E-05	GRAMD1B
6.00E-05	DYNC2I2
6.46E-05	MIR22HG
6.56E-05	ITPKC
7.33E-05	CHTF18
7.55E-05	POLA1
7.77E-05	HEG1
8.07E-05	RPS2P16
8.13E-05	HTRA3
8.26E-05	RDH12
8.26E-05	DNAJB1
8.26E-05	TTF2
8.26E-05	CENPU
8.26E-05	MAFF
8.31E-05	RECQL4
8.38E-05	POLD1
8.41E-05	PRIM1
8.59E-05	HSPA13
8.59E-05	TTK
8.59E-05	DEPDC1B

8.84E-05	CCNB1
8.99E-05	ZNF547
9.20E-05	HSPA5
9.22E-05	NRCAM
9.44E-05	MMP13
9.65E-05	LIG1
9.65E-05	TMEM187
9.65E-05	PPP1R15B
9.71E-05	KRTAP2-3
9.95E-05	NUF2
9.97E-05	ECT2
0.000104293	DHRS2
0.000109557	TEDC2
0.000110802	ASNS
0.000110802	SYNPO2
0.000112854	DDR2
0.000117667	NDC80
0.000118345	HIRIP3
0.000122164	NA
0.000123873	UBC
0.000128039	EXO1
0.000137385	FTH1
0.000141719	MYB
0.000141719	ZNF697
0.000141719	TACC3
0.000141719	CPEB3
0.000143216	HMGA2-AS1
0.000143216	KIF18A
0.00014417	TIMP1
0.000144862	TUFT1
0.000144862	CDC25C
0.000146428	C2
0.000147122	SLC1A4
0.000148952	NCAPG2
0.000150791	LYSMD3
0.000157138	HSPB8
0.000163046	MYBL2
0.00016949	ANLN
0.000172824	CENPM
0.000177601	VCAN
0.000182087	JUNB
0.000182477	LURAP1L
0.000186606	MAB21L3
0.000194932	TIMELESS
0.000194932	GABARAPL1
0.000195388	CCDC186
0.000201544	YPEL5

0.000206461	NA
0.000210525	KIF15
0.000211663	ITPRIP
0.000212182	GINS2
0.000213065	PIF1
0.000215373	IQGAP3
0.000215521	SLFN5
0.000219355	PCLAF
0.00022186	WDR90
0.000222517	PREPL
0.000232647	FAM72A
0.000234043	SEPHS2
0.000236761	FAM72B
0.000236761	FAM214A
0.000242762	MPZL3
0.000242762	RFC2
0.000242762	CEP55
0.000242762	NA
0.000242762	ANKFN1
0.000242762	CDCA5
0.000243019	AURKA
0.000244697	PTGER3
0.000249024	RNASEH2A
0.000251179	TDO2
0.000252603	MELK
0.000257026	PTHLH
0.000263874	JAM3
0.000266316	CAV1
0.000275077	FANCI
0.000275194	NIBAN1
0.000280613	ZMYM5
0.000287371	PRR11
0.00029179	SKP2
0.000304641	FAM72D
0.000306848	PLEKHM3
0.00030833	TROAP
0.000322262	ZBED2
0.000325143	BEST3
0.000325143	HMGB1
0.000325143	RANBP1
0.000325143	KBTBD8
0.000335253	RFC5
0.000337565	LOC286059
0.000337565	PDE4B
0.000338166	ATP2A1-AS1
0.000343014	RAD51
0.000344769	CCNB2

0.000347143	GMNN
0.000348235	LVRN
0.000354881	H19
0.000354881	NCAPG
0.000357255	SCFD2
0.000368715	CYP4F11
0.000369366	NMU
0.000374562	VEGFD
0.000383246	ATAD3A
0.000384465	CKAP2L
0.00040352	SLC6A12
0.000413552	TBR1
0.000414879	UBE2T
0.000415666	CDKN2B
0.000426691	LINC02273
0.000426691	NA
0.000426691	PTPRH
0.000426691	NA
0.000430592	ADAM17
0.000442938	CREBRF
0.000453209	LRIF1
0.000458649	CTAGE8
0.000459412	CREM
0.000467598	CENPA
0.000480506	NA
0.000491453	RBL1
0.000500579	ZNF654
0.000514886	UBA6-AS1
0.000520559	SCARA3
0.000523253	NA
0.000525588	PRC1
0.000530842	FOS
0.000530842	ZDHHC12
0.000531148	GATD1
0.000533484	NCAPD2
0.000552536	RFC4
0.000552536	C9orf24
0.000560384	PRIMA1
0.000560384	MSH2
0.000571326	NLRP1
0.000574835	LMNB2
0.000574835	LTA
0.000574835	NA
0.000574835	TRBV20OR9-2
0.000576694	ASF1B
0.000580132	PFAS
0.000583007	PARD6B

0.000599156	SPAG5
0.000613788	RCCD1
0.000613788	NA
0.000613788	DUSP10
0.000617682	NEK2
0.00062868	SPP1
0.000654977	NA
0.000654977	RACGAP1
0.000677966	HMMR
0.000679696	TXNL4B
0.000679696	INCENP
0.000681052	PCNA
0.000685907	HMGB3
0.000685907	NA
0.000686119	NA
0.000710972	NA
0.000711758	HMGB2
0.000713292	CLU
0.000775268	GEMIN4
0.00078473	ZNF189
0.00078473	ADGRG1
0.000817929	TMPO
0.000817929	UFM1
0.000831894	NASP
0.000908425	FAM83D
0.000908425	NA
0.000912799	LOC101927354
0.000914729	WNK3
0.000925211	OSER1
0.00092646	PSRC1
0.00092646	NA
0.000935026	GPR87
0.000936392	PRDM1
0.000936392	PGP
0.000943055	APOL6
0.000943055	STAT4
0.000943055	CPEB4
0.000963277	SRPX2
0.000963277	MAPRE3
0.000986015	DNAJC3
0.000996491	GCC1
0.001002619	CHAF1A
0.001022109	YBX2
0.001061637	RAD54L
0.001066182	CDC20
0.001093789	XBP1
0.001103707	BRIP1

0.001103707	SHMT1
0.001142575	ZNF699
0.001198599	NA
0.001198599	CORO1A
0.001203894	SDC1
0.001203894	CHIC2
0.001203894	TP73
0.001203894	PRKDC
0.0012406	RNASEL
0.001262697	KIF11
0.001272043	NA
0.001287204	ARMC6
0.001288371	SEL1L
0.001292618	DMGDH
0.00129729	NSD2
0.001304706	MIDN
0.001304706	FAM111B
0.001311498	LINC02889
0.001313958	CASS4
0.001373519	FAM122B
0.001404432	RPS2P14
0.001404432	ETFDH
0.001409993	CLN6
0.001424825	ADM
0.001439371	FRMD5
0.001444754	NTHL1
0.001462864	DNAJB2
0.001489663	SH2D5
0.001492967	LOC101927040
0.001514264	CDC7
0.001527482	FAM53C
0.001533076	POLR1G
0.001560084	SYNE1
0.001572372	NA
0.001646964	GINS1
0.001646964	CDCA2
0.001660932	SEMA4A
0.001675962	SELENOK
0.00168264	NA
0.0016927	CTSD
0.001700057	HMGB1P5
0.00171452	RAD54B
0.001722703	SAPCD2
0.001738857	RB1CC1
0.001785192	SRM
0.001785192	ARHGAP11A
0.001832076	MIR924HG

0.001832076	C11orf94
0.001832076	TMF1
0.001834633	POLR3K
0.001845493	TF
0.001845493	TERT
0.001845493	NA
0.001857958	TICRR
0.001880171	DHODH
0.001887912	NUSAP1
0.001925346	GOT1
0.00193002	GPAT2
0.001950242	LNCAROD
0.001953845	NA
0.001953845	NA
0.001963625	ZFAND3
0.001971062	KIZ
0.001980702	MFSD13A
0.001980702	E2F7
0.001980702	GBP3
0.002001074	EXOSC2
0.002001074	H2BC15
0.002013335	SGO2
0.002042148	C16orf72
0.002049132	SLC19A1
0.002113376	SQLE
0.002113376	MYOM1
0.002124457	C7orf61
0.002140968	FOXRED2
0.002169812	LINC01504
0.002193632	CHD2
0.002202018	DLGAP2
0.002204466	UPP1
0.00220966	MND1
0.002236533	MBD6
0.002236533	ARL14
0.002268779	NUP93
0.002268779	NRGN
0.002268779	DEPDC1
0.002274336	FAM214B
0.00231825	ZNF235
0.00231825	DENND2C
0.002352496	LOC101928994
0.002354092	LINC00662
0.002354092	GPR158
0.002354092	RFTN2
0.002354092	ARHGAP31
0.002363713	STARD8

0.002374981	GNB3
0.002387366	KRT16
0.002387366	ZNF160
0.002418141	DSN1
0.002418141	LCN2
0.00242639	C9orf131
0.002445007	CDCA8
0.002514939	AZI2
0.002514939	GFPT1
0.002522767	AANAT
0.002537715	RPL3L
0.002552702	F2RL2
0.002574669	TFE3
0.002602309	ATG9B
0.002602309	IFT20
0.002602309	KIAA1217
0.002606876	MTHFD1
0.002615993	LOC102546294
0.002673942	ME1
0.002682776	IL31RA
0.002682776	PNPLA8
0.002688329	DUT
0.002689014	NIT1
0.002711819	USP17L7
0.002737419	CCPG1
0.002737419	DMBX1
0.002762805	CTPS1
0.002762805	GEMIN5
0.002794279	TSC22D3
0.0028072	NHLH1
0.0028072	CEP152
0.00286061	CDKN2AIP
0.002876271	WDR45
0.002876271	CLUH
0.002876271	GIPC2
0.002876271	TNFRSF10D
0.002876271	PTTG1
0.002915464	KNTC1
0.002977352	NA
0.0029945	BAG3
0.0029945	PKD1L1
0.0029945	TBCD
0.002995043	CYB5R1
0.003001493	PEAR1
0.003013674	TRAIP
0.003028106	PIDD1
0.003037313	ZFYVE1

0.003047369	POLD2
0.003047369	EME1
0.003047369	CA13
0.003047369	FANCE
0.003050084	NA
0.003089964	RTN4RL1
0.003131181	CHMP4C
0.003144394	GLI2
0.003149734	SLC22A15
0.003180373	PLEKHJ1
0.003180373	FUT3
0.00320493	NAA80
0.00320493	ANO1
0.00320493	ATRIP
0.003214235	TENT5C
0.003224437	NAT8L
0.003224437	HCLS1
0.00328867	LARGE2
0.003353448	NA
0.003364799	LINC02616
0.003364799	DNAAF5
0.003375307	HEY1
0.003398307	CHPF2
0.003511367	CKAP2
0.003522881	F2
0.003522881	PAICS
0.003534636	DUSP1
0.003534636	CXCL8
0.003534636	FAM219A
0.003552075	CDKN3
0.003554567	RPS7P3
0.0036142	DCTPP1
0.0036142	TFDP1
0.003614481	CBX2
0.003657466	ABCB10
0.003658193	NA
0.00367013	CYLD
0.003687538	CDT1
0.003731731	LGALS8
0.003740828	NA
0.003750782	RPL13P8
0.003761094	KLK1
0.003797958	RUSC1-AS1
0.003807393	SPATA33
0.003807935	WEE1
0.003827364	NRM
0.003836908	MIR31HG

0.003836908	CHD5
0.003839522	ZSWIM6
0.003844088	MAFG
0.003884411	NA
0.00390564	NA
0.003914536	ABCB1
0.003914536	CD320
0.003936402	ZNF468
0.003961899	NA
0.003961899	TCOF1
0.004003762	HIF1A-AS3
0.004007738	NA
0.004007738	POLQ
0.004035213	LAMP3
0.00406888	FAM83G
0.00406888	NA
0.004085747	TPX2
0.004120936	ALOX12P2
0.004120936	SLFNL1
0.004120936	FANCD2
0.004130289	ALPK1
0.004146231	NA
0.004146231	FASN
0.004180375	PRMT7
0.00419188	RLF
0.004197564	TRAPPC6B
0.004200672	ZFP37
0.004206498	CCN2
0.004206498	SLC46A3
0.004215417	KIF4A
0.004224087	RMI2
0.004274703	LOC100288637
0.004292524	RBAK
0.004305708	NCL
0.004315893	REM2
0.004357919	ZNF441
0.004357919	NOC4L
0.004367079	BTN2A1
0.004423527	RPS6KA4
0.004471978	ABCG5
0.004471978	CDO1
0.004514323	PELI1
0.004545988	APOLD1
0.004663846	ACAT2
0.004687686	ANAPC1P4
0.004736612	JAG2
0.004829063	DARS2

0.004829063	FOXRED1
0.004855357	TUBG1
0.004876969	ALS2
0.004901941	ULBP1
0.004901941	MRNIP
0.004905355	LRRC20
0.004929224	GOSR2
0.004929224	EEF2KMT
0.004946948	KIAA0513
0.004967428	HAUS5
0.004973548	RRS1
0.004983242	GABRR2
0.005034763	NFKBIL1
0.0050727	THOP1
0.00511575	TMEM41B
0.005163462	SLC5A11
0.005170029	DAPP1
0.005171542	IRF7
0.005233306	NA
0.005233446	POC1A
0.005265563	GAL
0.005284929	LOC652276
0.005284929	ACLY
0.005284929	NA
0.005284929	ZNF548
0.005284929	NA
0.005342576	ELFN1-AS1
0.005374388	NDC1
0.005376956	CAD
0.005385202	RUVBL1
0.005385202	ZNF44
0.005385202	GCNT3
0.005400465	UAP1L1
0.005424287	BUD31
0.005442531	NA
0.005533687	GINS4
0.005551939	CDC20P1
0.005556318	NA
0.005585071	DEDD2
0.005585071	PRECSIT
0.005632081	ZNF473
0.005691265	ZFP36
0.005741346	KDM6B
0.005742792	MAP1A
0.005748701	KIF20B
0.005786592	MMS22L
0.005786592	ZFAS1

0.005786592	PLEKHM1
0.005786592	CALCOCO2
0.005812354	PIGH
0.00582648	PLK4
0.005859593	GOLGB1
0.005867147	RTKN2
0.005868852	TMEM167B
0.005868852	STC2
0.00588645	ABCB4
0.005920416	KRBOX4
0.005977497	ZNF791
0.006001133	CAB39L
0.006091076	NNAT
0.006108357	GLIS3
0.006184073	NA
0.006281452	TMEM116
0.00633935	CHEK2
0.006373275	ZNF280A
0.006451552	ZNF430
0.006559933	KIF14
0.006559933	NXF1
0.006656762	TAF13
0.006656762	SLC5A6
0.006656762	RFPL3S
0.006698565	ICMT
0.006717155	AADAC
0.006819449	TEX14
0.006844531	TNFSF9
0.006847953	BRF2
0.006862121	FBF1
0.006883097	TEDC1
0.006892443	TSGA10
0.006906386	TMEM40
0.006908907	MAP3K6
0.006967186	NUP35
0.006983478	LURAP1L-AS1
0.007012915	TSPY26P
0.007017236	OCLN
0.007099947	UCK2
0.007126438	NCOA3
0.007173317	SNX29
0.007242414	PPIA
0.0072736	SLC33A1
0.0072736	CAVIN3
0.0072736	NA
0.0072736	FN3KRP
0.007282829	MAGEB3

0.007331318	SLC10A1
0.007343721	SFXN2
0.00735072	ABCA9
0.007495227	TNFRSF13C
0.007495227	SNORD14E
0.007495227	POLE
0.007495227	ZNF597
0.007498	GPAT2P1
0.007575536	TSC22D2
0.007605396	MTARC1
0.007627742	STX5
0.007627742	RIBC2
0.007627742	TLR10
0.007633965	DPYSL5
0.007662075	RN7SL472P
0.007662075	PGF
0.007718482	TUT7
0.007718482	NA
0.007744921	BDH1
0.007744921	CREB3
0.007744921	RFFL
0.007834198	NSMCE4A
0.007853398	DCPS
0.007878299	NA
0.007887524	INSIG1
0.007913727	NA
0.007932106	LOC440700
0.007932106	CREB5
0.007992162	PHTF1
0.00799454	FRS2
0.008111492	TRIML2
0.008120371	USP13
0.008133336	IFRD2
0.008133336	ELL2
0.008133336	UTP20
0.008168509	HRH1
0.008193277	ARMCX3
0.008254257	PARP1
0.008260048	INTS1
0.008260048	GRK7
0.008268658	ARHGEF26-AS1
0.008311281	WNT10B
0.008311281	PRKAB2
0.008320939	UNC13D
0.008320939	PAQR4
0.008360277	MSH4
0.008360277	PDSS1

0.008360277	MEIOSIN
0.008360277	ZSCAN26
0.008409086	ZNF484
0.008417434	PRSS53
0.00841913	MON1B
0.008453011	IL20RB
0.008453011	NA
0.008504703	FBXL19-AS1
0.008541003	BCL10
0.008541003	DPH2
0.008541003	KLHL28
0.008570028	NA
0.008570028	CCDC51
0.008570028	S100P
0.008729164	CTU2
0.008759736	PLCL1
0.008759736	TAGLN3
0.008781552	ZFAND5
0.008785533	DCK
0.008837525	HNRNPD
0.008837525	SUCNR1
0.008868512	NA
0.008868512	TBX3
0.008868512	MPHOSPH9
0.008876648	SLC3A2
0.008887716	MOB3C
0.008893926	FBXL6
0.008893926	ZSCAN12
0.008893926	YIPF5
0.008893926	SLC41A2
0.008941249	LRRC45
0.008972109	GLA
0.0089836	FBXO16
0.009072912	ST8SIA2
0.009112629	CCDC28A
0.009149657	SLC10A7
0.009149657	ERFE
0.009149657	TBRG4
0.009149657	NA
0.009154336	SAC3D1
0.009172296	LARP6
0.009172296	SERINC1
0.009247351	NA
0.009257882	JHY
0.009311396	RNF139-AS1
0.009350433	NA
0.009350433	SMC1A

0.009452123	NA
0.009489016	GAS6
0.009506332	DMRTA2
0.009541154	TMEM177
0.00964417	VEGFA
0.009709787	TAX1BP1
0.009797628	ACAD9
0.009811031	KLF5
0.009811031	TNS4
0.009811031	NA
0.009811031	B4GALT2
0.009811031	ANKLE1
0.009823843	ALDH16A1
0.009848397	ZNF79
0.009853274	GALR2
0.009877105	TLR1
0.009877105	TRAM1
0.009877105	PTPRC
0.009894925	CENPV
0.009914898	LRFN4
0.009932707	BDKRB1
0.009947848	SOS2
0.010030428	USP53
0.010074633	EGFL7
0.010174361	MRTO4
0.010174361	CTDSPL
0.010174361	PARPBP
0.010312034	NUDT1
0.010312034	FABP5
0.010312034	CFAP61
0.010376737	MCF2
0.010432399	DRP2
0.010498449	GARS1
0.010572169	ZFPM2
0.010629669	TLR6
0.010734169	NOL6
0.010823516	RASD1
0.010860394	ZBTB43
0.010996171	P4HA2
0.011055601	NIPAL3
0.011069544	AOX1
0.011121485	PKN3
0.011228167	EFNA2
0.011228167	PLEKHG4
0.011330203	SCYL2
0.011467559	GCSH
0.011489417	BPIFC

0.011489837	PPP2R3B
0.011490092	LSM4
0.011539297	FDPS
0.011668072	MAMDC4
0.011695134	EIF5
0.011762332	TRIM16
0.011906433	NA
0.011978442	NA
0.012003604	NPM3
0.01200385	SIGMAR1
0.012022375	SERP1
0.012039152	METTL1
0.012109851	CSGALNACT2
0.012109851	CCND1
0.012188186	ATG4A
0.012209634	ZNF266
0.012248892	SLC29A1
0.012248892	KNL1
0.012248892	PXDC1
0.012248892	MYLIP
0.012248892	KIAA0319
0.012248892	LINC01647
0.012248892	ZNF701
0.012304716	FAM83C
0.012317848	WDR5
0.012327162	ZEB1
0.012327162	NDUFV1
0.01236143	OGDH
0.012375185	NA
0.012419064	NA
0.012444101	NA
0.0124813	PLEKHO2
0.0124813	ENOSF1
0.012631772	ATAD2
0.012631772	RNF166
0.012631772	TFPI
0.012631772	BCAN
0.012631772	RABL6
0.012631772	FBXO5
0.012633246	IER2
0.012633246	NA
0.012637909	NA
0.012637909	MRPS34
0.012637909	COL12A1
0.012637909	USP38
0.012638177	NUCKS1
0.012663095	FOXA1

0.012663095	CYP2R1
0.012663095	NA
0.012667996	MYEOV
0.01277296	BORA
0.012795785	MIPEP
0.012804816	SNX11
0.012865775	SAMD8
0.012888245	PUSL1
0.012908717	TBRG1
0.012950241	PLAG1
0.013140006	CCDC62
0.013170085	SLC25A25
0.013170085	SHPK
0.013170085	FAM216A
0.013170085	KLHL15
0.013170085	CCR3
0.013251854	PRKG2
0.013262085	GRM8
0.013280229	SPTY2D1
0.013280229	LDHB
0.013395313	GPR75
0.013395313	LOC646665
0.013416796	ASB18
0.01346961	CCDC34
0.01346961	KRT8P12
0.013531517	KLHL17
0.013531517	NA
0.013564497	EFCAB12
0.013577536	RUSC1
0.013656158	UBL3
0.013695635	NA
0.013696152	ABL2
0.013696152	MRPL12
0.013883594	NA
0.013907873	NUP210
0.014004257	TMEM231
0.014144039	KCMF1
0.014208872	SRPK3
0.014256037	SLC17A8
0.014287083	ZNF460
0.014333037	ERO1B
0.014338191	TIPIN
0.014338191	NA
0.014389481	NA
0.014434758	ZNF277
0.014458786	KCNQ1OT1
0.014537239	C6orf62

0.014541762	CHRM5
0.014541762	IFNWP19
0.01458855	ZFYVE21
0.014605953	LINC01534
0.014621042	RAB5A
0.014647941	NA
0.014668259	NA
0.014668259	CIR1
0.014673885	HCG27
0.01467771	ETV5
0.01467771	VASN
0.014916614	HSPA6
0.014953485	PLAT
0.01507392	NA
0.015325614	TYRP1
0.015358881	HOXB4
0.015358881	SCRIB
0.015358881	CDC45
0.015410931	FAM8A1
0.015417922	TBC1D23
0.015417922	EMC3
0.015417922	ZNF394
0.015456936	MCMBP
0.015482973	DDX51
0.015482973	SCART1
0.015495057	RRP1B
0.015495088	TRAPPC2B
0.015649435	TRPV3
0.015649435	VWA5A
0.015649435	NOP56
0.015658408	NA
0.015696473	FAM126B
0.015758154	NA
0.015833235	HADH
0.016051889	MOAP1
0.016147137	FHL2
0.016150241	ATF4
0.016150241	ZNF703
0.016175384	MIR31
0.016175384	DNAJC3-DT
0.016199904	C5orf47
0.016199904	MIR103A2
0.016206732	C1QBP
0.016206732	RAMP1
0.016206732	NA
0.016227596	CIP2A
0.016227596	PRDM2

0.016235443	NA
0.01633627	NAT16
0.01633627	SGTB
0.016346839	TM4SF18
0.016387425	CLCN3
0.016548736	HAPLN3
0.016548736	THUMPD3-AS1
0.016549811	ICOSLG
0.016579219	FBXO30
0.016579219	MGME1
0.016630691	NA
0.016734712	CTSV
0.0168033	PDPK2P
0.016862144	RND3
0.01686559	MTX1P1
0.01686559	NBR1
0.016936331	CIDECP1
0.016971815	LINC01963
0.01700662	SNRPD1
0.01700662	NCKAP5L
0.017085747	QDPR
0.017091722	BLVRB
0.017316975	NA
0.017316975	WDR76
0.017335597	MYBL1
0.017403465	SHCBP1
0.017406829	GEN1
0.017463977	NFE2L2
0.017501866	ZNF383
0.017501866	ZSCAN20
0.017501866	SYNCRIP
0.017513754	CENPJ
0.017538787	NA
0.017585416	PRPS2
0.01767579	ITGA5
0.01769944	ATAD3B
0.017711616	NA
0.017729676	SMAP1
0.017729676	NA
0.017729676	FAM151B
0.017767539	ADAM11
0.01779231	PCYOX1L
0.01779231	TCEANC
0.017850454	SLC6A9
0.017850454	LTN1
0.017863817	LOC101927533
0.017863817	TNFSF13B

0.017903609	SMG1P7
0.017903609	SUV39H1
0.017903609	PA2G4
0.017903609	RRP1
0.017983526	SLC25A30-AS1
0.01800312	ANP32A
0.01802036	ZNF192P1
0.01803552	C21orf91
0.01805406	HNRNPR
0.01805406	PHF19
0.01805406	SYNJ1
0.01805406	IPO9
0.018076062	PLD6
0.018191879	NA
0.018191879	FRK
0.018191879	SLC25A39
0.018227394	DYNC1I1
0.018301964	URB2
0.018386788	CNTRL
0.018393361	GAMT
0.018422609	TRIM47
0.018422609	POLD3
0.018422609	DIAPH3
0.018511894	JAM2
0.018511894	DNAH12
0.018511894	NA
0.018511894	SPDYA
0.018511894	IRS2
0.018511894	NA
0.018511894	ZNF808
0.018548334	HBP1
0.018736569	RGMB-AS1
0.018827788	NA
0.018951769	COQ2
0.019081944	STYK1
0.019092607	NA
0.019111892	PWP2
0.019139808	PRODH
0.019139808	CENPX
0.019171385	CENPE
0.019297713	YDJC
0.019302233	NA
0.019304246	BSN
0.019304246	PTGES2
0.019466737	TMEM160
0.019630116	ALDH1B1
0.019640129	AFF4

0.01964299	CLK1
0.01964299	RNF11
0.01964299	NME1
0.01964299	ZNF615
0.019658525	PRTG
0.019658525	TOX4
0.019691438	BSDC1
0.019761819	CCDC32
0.019832667	RELB
0.019832667	NOP14
0.019832667	MAP7D2
0.019848065	HTR6
0.019848065	PSMD10P2
0.019860195	TMEM39A
0.019979408	TMEM87A
0.020030392	ARL5B
0.020030392	ADCY10
0.020086225	WDR92
0.020129979	NA
0.020144586	RAD51AP1
0.020187808	NQO2
0.020187808	SLU7
0.020187808	MORC3
0.020190997	TREML3P
0.020190997	LRP3
0.020212469	NA
0.02021357	MTBP
0.020333288	CAV2
0.020613603	PYCR3
0.020651138	NA
0.020667574	WDR18
0.020715694	RAB30
0.020833933	NA
0.020869506	BANP
0.02095094	MTERF3
0.021048924	BRI3BP
0.021048924	RHBDD3
0.021048924	CKB
0.021076613	NUDT15
0.021094151	LOC284930
0.021106998	CCDC126
0.021106998	LOC101929709
0.021425313	NA
0.021494214	KCNE4
0.021530031	SGO1
0.021558218	IFT122
0.021615694	GOLPH3L

0.021615694	WARS1
0.021615694	MAST4
0.021637622	TMEM237
0.021637622	TUBA1B
0.0216479	POPDC3
0.021659118	ERVMER34-1
0.021691495	PPIH
0.021738303	PSAP
0.021751136	LSMEM1
0.021851816	LOC100130714
0.021864809	MSH5
0.021869472	C1QL1
0.022102992	ZBTB21
0.02211809	PPP5C
0.0222851	AAK1
0.022373798	NA
0.022373798	NA
0.022412945	EIF1B
0.02242579	TTBK2
0.02242579	CSE1L
0.022458059	MXD3
0.022489471	MRPS26
0.022493365	GNGT1
0.022592077	ZNF408
0.022787182	BBS12
0.022787182	SMC2
0.022795795	TMPO-AS1
0.022821587	RFC3
0.022826997	CEBPB
0.022828654	NA
0.022857516	SLC25A10
0.022857516	RAN
0.022867921	LOC102724064
0.022867921	TIMM8A
0.022891703	OIP5
0.022963361	BLZF1
0.023101003	TRIM46
0.023104939	ZNF761
0.023104939	DDX54
0.023109936	KYAT1
0.023142865	DIAPH2-AS1
0.023180351	LINC00324
0.023342857	NA
0.023371769	CHEK1
0.023465988	NA
0.023569339	TSPAN8
0.023569339	TTN

0.023569339	SLC43A3
0.023569339	RNF6
0.023569339	CBR3
0.023569339	RPL7AP16
0.023572226	LOC100128007
0.023572226	PRNP
0.023649138	DIS3L2
0.023664767	TESK1
0.0236683	SMARCB1
0.0236683	RRP9
0.023710016	DUSP8
0.023729669	LOC100133091
0.023729669	OXCT1
0.023769525	SLC25A11
0.023781879	LOC400627
0.023903505	CKMT1A
0.024038764	ANGPT4
0.024044562	GDF9
0.024044562	NDUFB10
0.024115576	LRRC66
0.024115576	RNU1-1
0.024115576	GTPBP2
0.024157297	ZNF227
0.024157297	NOCT
0.024420285	SNRNP25
0.024420285	NOP16
0.024420626	NLE1
0.024511843	CENPK
0.024550455	MID2
0.024550455	SIPA1L2
0.024550455	FRRS1
0.024550455	ABCB6
0.024550455	INAFM2
0.024550455	LHX2
0.024570017	NA
0.024674623	ARF4
0.024694527	PTMAP5
0.024698179	WHAMM
0.024698179	NUBP2
0.024838119	CYC1
0.02489405	FAM135A
0.02489405	NA
0.02489405	QTRT1
0.024966955	ZNF613
0.024966955	PARP14
0.024966955	HES6
0.024966955	CCDC170

0.025036842	NA
0.025055259	KISS1R
0.025055259	PTP4A3
0.025055259	SLC39A10
0.025055259	MGAM
0.025055259	DKC1
0.025055259	ZSWIM3
0.025060157	HSD17B8
0.025186621	CEP131
0.025215597	MIS18A
0.025292532	ATP6V0E2
0.025316205	CKAP5
0.025316205	C4orf46
0.02537717	CCDC137
0.025410915	DCAF4L1
0.02548843	AMDHD2
0.025501642	KCTD5
0.025538283	AAAS
0.025603762	CNOT4
0.02560694	TNNC1
0.025643537	PRR22
0.02575032	STX2
0.025819176	KLHL23
0.025832646	PP2D1
0.02595302	MACO1
0.026014126	SETX
0.026035035	NRARP
0.026359445	NEK10
0.026381115	NA
0.026381115	NCOA7
0.026381115	COA7
0.026381115	PAXX
0.026381115	ODF2
0.026381115	GPRASP1
0.026644247	MIR3682
0.026699923	CHP1P2
0.026749761	CCDC86
0.026752869	ZNF778
0.02687114	SMARCA4
0.02702544	CNTLN
0.027099352	ZNF329
0.027100173	CASP2
0.027105619	NA
0.027143409	LINC00907
0.027171792	ZSCAN31
0.02717747	MTFR2
0.027255678	NRBF2

0.027271533	ABCD1
0.027271533	TAF7
0.027393196	NA
0.027393196	SLC2A4RG
0.02744016	INTS6
0.027464829	OLAH
0.027464829	ERP44
0.027549502	CTDSP1
0.027551643	NEU1
0.027563752	NCS1
0.027580816	H1-6
0.027580816	THSD1
0.027580816	TMEM19
0.027603921	ETV3
0.027603921	NME4
0.027613838	KDM2A
0.027661167	CLDN6
0.027773167	CBX5
0.027816793	RNF148
0.027901694	SLC25A35
0.027936628	MED24
0.028050604	CD55
0.028106368	FAM160B1
0.028206182	KLHL21
0.028211454	SPSB1
0.028276028	TFR2
0.028315085	LINC02367
0.028403027	MRPL24
0.028475534	TXNL1
0.02851461	FBXL20
0.02851461	SLC1A5
0.028590224	SFXN5
0.0286525	CD163
0.028822045	ATG14
0.028829371	DUSP26
0.028906262	FAM189B
0.028906262	TALDO1
0.028961524	LAMTOR3
0.02910636	CTXN1
0.02910636	MTMR3
0.02910636	VEZT
0.02910636	AADACP1
0.029115029	ANKAR
0.029139844	PLPP5
0.029178678	DNPH1
0.02923332	JMJD8
0.029466932	ZNF28

0.02955685	CALM3
0.02955685	FH
0.029600063	NA
0.029600069	CA3
0.029600069	CHAF1B
0.029600069	GGCT
0.029600069	CERS1
0.029615503	ARL2
0.029626134	ZNF485
0.029626134	LRRC8A
0.029626134	ZADH2
0.029661154	PSEN1
0.029661154	EIF1
0.029683711	HARBI1
0.029709372	B4GALNT1
0.029737685	OTUD1
0.029744894	SLC25A15
0.029746564	TMEM59
0.029776761	NA
0.029905127	ZNF800
0.029905127	SVIL-AS1
0.029917229	KCNN1
0.029933802	TMEM200B
0.029990469	KCNQ2
0.030155334	RPIA
0.030191272	C2orf78
0.030199373	FAIM
0.030206026	WNT3A
0.030206026	RPARP-AS1
0.030206026	NA
0.030238519	CD22
0.030397523	CRIPT
0.030445804	TIPARP
0.030513592	NA
0.030513592	PITHD1
0.030513592	IRAK2
0.030513592	HAVCR2
0.030513592	ESRP2
0.030625267	PUM3
0.030633926	RHOT2
0.030745472	LSS
0.030752437	WDR26
0.030777147	LOC150051
0.030885786	DDN
0.030885786	RPS6KC1
0.030885786	NA
0.030891761	ATG101

0.030891761	TMEM117
0.030982361	PEX12
0.031067199	ZNF271P
0.031067199	HDHD5
0.031159754	ACBD7
0.031231105	SPACA6
0.031243661	NA
0.031243661	MOB2
0.031253636	NA
0.031276258	NA
0.031332141	SLC30A7
0.031332141	BEND3
0.031393378	WNK2
0.031399653	TSPYL2
0.031430202	HGH1
0.031430202	CD4
0.031450607	COLGALT1
0.031478602	HMGA2
0.031609353	PELO
0.031668324	SUPT6H
0.031668324	NA
0.031668324	NA
0.031698757	ALPL
0.031698757	FAM161B
0.031978356	ZNF823
0.032034209	TFAP4
0.032039443	NA
0.032076834	MAPK12
0.032112795	FBXO42
0.032200785	ZACN
0.032226264	SUPT7L
0.032268864	PLTP
0.032268864	NFKBIZ
0.032604374	SIVA1
0.032895126	MSH6
0.032905669	GRAMD2A
0.03291019	ZNF490
0.032931716	MBD5
0.032931716	TEAD4
0.032931716	SEC24A
0.032931716	XRCC1
0.032931716	LMF2
0.032955046	AXL
0.033051264	ATP10D
0.033051264	NA
0.033051264	LINC02405
0.033114012	PYGM

0.033258125	TBC1D15
0.033261712	NA
0.033379185	FSCN3
0.033514449	AFF3
0.033514449	FAM241B
0.033514449	TSEN54
0.03351992	SNX5
0.033564008	SHROOM1
0.033565259	CCDC160
0.033639202	NFIA
0.033741727	R3HCC1L
0.033755821	GUSBP1
0.033755821	ZNF37A
0.033773173	BMPR1B
0.033815218	SSRP1
0.033981444	TBPL1
0.034071613	LRRC26
0.034134467	ALKBH6
0.034247226	RNF24
0.034377622	ZNF622
0.034395586	SFI1
0.034395586	GPSM2
0.034395586	ZNF274
0.034408336	CC2D2A
0.034462778	SLC51A
0.034462778	FMO5
0.034546053	CYB5RL
0.034706774	DHRS4
0.034724814	EXOC8
0.034816565	NA
0.035055404	NA
0.035055404	STAC3
0.035182179	NPR3
0.035596355	SCLY
0.035596355	MSRB1
0.035824232	CDKN1A
0.035876323	ZNF175
0.03600483	STAT6
0.036057892	HNRNPF
0.036057892	STK25
0.036061131	CSPG4
0.036061131	ZNF721
0.036292935	FLOT2
0.036332421	NA
0.036332421	ITGB3BP
0.036332421	POGLUT3
0.036459343	RUSC2

0.03653427	MED8
0.036559742	TMEM121
0.036610441	GLOD4
0.036610441	IER5
0.036813411	EGF
0.036851751	PP7080
0.037063475	COG3
0.037205067	ARID3B
0.037205067	FSCN1
0.037205067	CREB3L3
0.037205067	ZNF197
0.037205067	RYBP
0.037205067	RPS4XP17
0.037400295	HES4
0.037537172	D2HGDH
0.037572649	UBE2B
0.037613555	SEC14L1P1
0.037640097	TSPAN31
0.037685884	VARS1
0.037704702	MID1
0.037704702	NA
0.037711877	TUFM
0.037728367	TG
0.037728367	FBXO8
0.037858541	TEAD2
0.037885642	NA
0.03799805	ABCA10
0.038076718	TRIM38
0.038076718	WDR4
0.038076718	SUMO3
0.038094835	ULK3
0.038329239	NCOR1
0.038329239	HSCB
0.038329239	OR6E1P
0.038442127	JPH1
0.038442127	CNTROB
0.038442127	HSPH1
0.038442127	SLC35G2
0.03847416	POLR3G
0.038526371	SLC10A5
0.038797832	SNRPA
0.038797832	GGT1
0.038797832	SLC37A4
0.038903581	PIGQ
0.038944955	CASP10
0.038990481	WDR12
0.038990481	DUSP13

0.039102452	TRIM5
0.039120389	NAALAD2
0.039127336	NR2C2AP
0.03920092	CENPW
0.039292043	NOS3
0.039498934	GDAP2
0.039515798	PMS2CL
0.039553157	PRKRIP1
0.039674417	NA
0.039726943	SDF2
0.039960647	SNIP1
0.039993534	UTP4
0.039993534	NA
0.040005543	STX3
0.040074556	ZNF14
0.040214313	MIS18BP1
0.040482068	POU2F2
0.040482068	SLC3A1
0.040482812	FARSB
0.040482812	BACH2
0.04049049	CPLX1
0.04049049	ARHGEF5
0.04049049	TNC
0.040613719	NA
0.040725333	SHOC1
0.040768504	NA
0.040792778	CEP78
0.040792778	FLNC
0.040852769	MARCHF1
0.040914596	MPP4
0.040919549	IMP3
0.041061987	MFSD11
0.041092702	EHMT2
0.041093131	RNF19A
0.041150084	NAGLU
0.041215554	ENHO
0.041215554	ZNF641
0.041215554	ZNF512B
0.041215554	CCSER2
0.041360193	JMJD6
0.041360193	RBM48
0.04137589	DHX37
0.04137589	ATP5MC1
0.04137589	MIRLET7F1
0.04137589	SUSD2
0.04137589	TNKS
0.04137589	FAM209A

0.041520811	NA
0.041539728	NA
0.041673825	ZNF557
0.041743714	BMT2
0.041743714	SLC66A1
0.041743714	CLHC1
0.041786041	PHB
0.041786041	CEMIP2
0.041934182	NA
0.042013899	ZNF404
0.042057329	TET2
0.042261256	NUP37
0.042318824	HOXA1
0.042435925	TLCD1
0.04254798	CARMIL2
0.042569385	ZNF621
0.042668241	TXNRD1
0.042668241	NA
0.042701293	NA
0.042926934	NA
0.042926934	CCNE2
0.042926934	PM20D2
0.042926934	ABCA5
0.042926934	OR2A20P
0.042926934	SDR42E1
0.042926934	P3H3
0.042926934	PODNL1
0.043029634	CSTA
0.043029634	FIBP
0.043029634	ZKSCAN8
0.043119215	SERINC3
0.043144274	AGRN
0.043175949	BLOC1S2
0.043353284	DNAJA4
0.043561755	ABHD12B
0.043585393	NR2F2
0.043585393	NA
0.043701972	KDM4A
0.043878636	PUS3
0.044205912	VPS37B
0.044266751	ZNF585B
0.04427362	RPS17P3
0.044337645	PPAT
0.044411965	PACSIN3
0.044423443	MARVELD1
0.044423443	CDK2
0.044423443	CFAP77

0.044423443	MTR
0.044460492	HLA-E
0.044460492	IMP4
0.0444754	ZBED3-AS1
0.044639436	E2F6
0.044704975	RNASEH2B
0.044734607	NF1
0.044751729	TCAM1P
0.044780975	PHF1
0.044780975	PRMT6
0.044780975	MUC1
0.044780975	ARHGAP4
0.044780975	TDH
0.044849558	IPO5
0.044945648	ZNF546
0.044945648	OPLAH
0.044964754	LINC-PINT
0.044964754	TFG
0.044975134	C20orf27
0.045021012	ZBTB49
0.045021012	MRGPRF
0.045021012	CAMKK2
0.045183673	REL
0.045213753	EMP2
0.045213753	UBN2
0.045213753	NA
0.045213753	LOC100422627
0.045213753	RNF10
0.045231418	NA
0.045231418	NUDT8
0.045420381	LATS2
0.045423394	SH3TC1
0.045423394	PDHA1
0.045436914	ENTPD7
0.045641291	MECR
0.045641291	ADSL
0.045723146	RNASE1
0.045723146	RPL22L1
0.045816667	CEBPG
0.045816667	DUS1L
0.045824814	PTMA
0.045846248	LAMA3
0.045846248	GPATCH3
0.045846248	ATP6V1B1-AS1
0.045846248	INO80D
0.045846248	LINC02608
0.045846248	ZNF567

0.046057791	NA
0.046070504	TCAP
0.046186518	CPNE7
0.046243474	UGDH
0.046252824	LAT2
0.046252824	KCNS1
0.046252824	NA
0.046381697	PI4K2B
0.046423236	UTP15
0.046430092	RTN4R
0.046573462	NECAB2
0.046573462	ZSCAN5A
0.046573462	HELB
0.046573462	NXPE3
0.046574657	H2BC12
0.046591393	AKT1
0.046656111	ZNF286B
0.046669889	CFAP70
0.04678514	THBS1
0.04679667	PKMP3
0.04679667	FAM71F2
0.046884261	MAPK15
0.046910942	PTH1R
0.047003174	RNF103
0.047078616	MILR1
0.047078616	GNB4
0.047083988	INO80B
0.047083988	TRAP1
0.047124576	BEND7
0.047131017	NA
0.047159234	RNF185
0.047196151	TBC1D13
0.047227069	CYP19A1
0.047258306	LOC107986114
0.047258306	NA
0.047258306	NA
0.047333057	NA
0.047338709	ANKRD13B
0.047413257	DLAT
0.047413257	DSG4
0.047413257	MDN1
0.047413257	AP1G1
0.047413257	OFD1
0.047433007	UBAC1
0.047505748	ARL9
0.047505748	PNPLA3
0.047515226	GAS2L1

0.047536545	NA
0.047624335	GCSIR
0.047731885	GPATCH2L
0.047774607	PALB2
0.047841732	ATIC
0.047991744	GPAT3
0.048057024	KDM1A
0.048100132	APEH
0.048100132	NDRG1
0.048168884	NA
0.048168884	NA
0.048239747	RIPOR1
0.048255246	EML3
0.048453882	SETMAR
0.048490134	GPAA1
0.048594634	EPC1
0.048594634	SLC29A2
0.048594634	BRICD5
0.048594634	GSDMD
0.048594634	HMBS
0.048628208	MACROH2A2
0.048670766	NA
0.048717156	NA
0.048746941	POLA2
0.048779204	FER1L6
0.04877993	GCNT4
0.048831594	NA
0.049124178	HSPA8
0.049160394	BOD1L1
0.049167403	MYBBP1A
0.049167403	NA
0.049241527	SHOC2
0.04934947	NUP188
0.04934947	CEP192
0.049357986	DCUN1D3
0.049357986	AFTPH
0.049391529	ZSCAN25
0.049391529	TTLL12
0.049427893	PROZ
0.049427893	FAM183BP
0.049442687	HECW1
0.049538621	NA
0.049538621	EAF1
0.049576594	ZWILCH
0.049669759	AIMP2
0.049837203	NA
0.049837203	TMEM147

0.049837203	YIPF1
0.04985343	EGR3
0.049891553	TUBB8P2
0.049891553	SPATA2
0.049891553	DCLRE1B
0.049891553	INSC
0.049891553	DIRAS3
0.049905455	HUS1B
0.049979672	DENND4A
0.050041012	KCTD20
0.050041012	C2orf42
0.050041012	NA
0.050099941	NA
0.050120255	ARHGEF2
0.050124207	SDHA
0.050332353	ELAPOR1
0.050368312	NA
0.050368312	FICD
0.050368312	NA
0.050474289	MRPL23
0.050635191	NA
0.050635191	PPM1G
0.050662183	RAD51C
0.05080537	NA
0.050850703	ZNF234
0.050936003	KCNH3
0.051034514	FKBP5
0.051080038	CFD
0.051386856	RPL12P24
0.051479387	MAP3K14
0.051479387	ACTN4
0.051484587	KDM5B
0.051484587	ADSS2
0.051484587	POLR1B
0.05165484	CHMP5
0.051740392	TRAPPC2L
0.051886401	NA
0.051886401	KANK2
0.051895641	PRXL2B
0.051905078	LGR5
0.051905078	ST7
0.052044517	N4BP2L1
0.052063174	TSPAN19
0.05208205	NA
0.052115315	ZC3H12A
0.052115315	BRD2
0.052148631	TSLP

0.052185041	FBRSL1
0.052207184	QPCTL
0.052388693	CSNK1G1
0.052455259	STK32C
0.052478203	NA
0.052619525	HECA
0.05261999	LRP10
0.052674661	CTC1
0.052694411	NA
0.052958616	SLC25A1
0.053093192	ADCK5
0.053109917	LMO4
0.053118759	PGAP3
0.053180142	ADCY3
0.053298781	POLRMT
0.053301248	CAPN10-DT
0.053325721	NA
0.053337579	KLHDC7B
0.053584282	RGS4
0.053634547	ING3
0.053634547	ZIC2
0.053649343	DCXR
0.05365313	IL6R
0.053662581	KDM6A
0.053662581	TAB1
0.053753816	B3GALT5
0.053753816	ENC1
0.053888195	IGSF1
0.053888195	EPG5
0.053888195	ENG
0.053954949	CARS1
0.053997289	NA
0.054032134	HIPK3
0.05414869	CAPN7
0.054184086	KATNB1
0.054220963	CCDC174
0.05437997	WDR47
0.054472082	PRMT1
0.054538412	TSPAN4
0.054775762	HM13
0.0548144	SHLD3
0.054817315	NA
0.054833431	NA
0.055004973	NA
0.055004973	SEPTIN8
0.055004973	PARVB
0.055154817	MRPS12

0.0552164	ROCK1P1
0.0552164	MED26
0.055267567	LINC02280
0.055267567	POU4F3
0.055267567	NA
0.05546615	BZW2
0.055522613	SPDL1
0.055522613	FBXL16
0.05557373	PIWIL2
0.05557373	ABI3
0.055605684	PCYT2
0.055642337	SRSF7
0.055665135	SH3BP5L
0.055800955	FJX1
0.055880108	THCAT158
0.055880108	NA
0.056389127	SPPL2B
0.056389127	PSME4
0.056389127	BROX
0.05643165	SDCBP2-AS1
0.056446858	TMEM217
0.056503561	TPMT
0.056589711	ZSCAN30
0.056589711	ABHD16B
0.056628284	PRKG2-AS1
0.056628284	EEF2K
0.056663352	TPT1
0.056676423	MED31
0.056676423	NA
0.056707741	ZNF669
0.056707741	SLC25A40
0.056850198	MCL1
0.056850198	NA
0.057111283	NA
0.057111283	NLN
0.057111283	URB1
0.057129029	CABP7
0.057191817	ZNF226
0.057191817	DISP1
0.057191817	ZNF143
0.057193295	METTL8
0.057374643	ZNF436
0.057523416	SLC30A3
0.057540615	BRCA2
0.057650067	FAM120AOS
0.057685146	NA
0.057685146	CACNA1F

0.057685146	BCAS2
0.057771188	NA
0.057803853	NDEL1
0.057803853	FGD3
0.058068413	ARVCF
0.058068413	UBQLNL
0.058077372	PPM1B
0.058145574	GZF1
0.058163078	SERPINH1
0.058292491	NA
0.058336159	NA
0.058336159	BCAT2
0.058336159	ENTR1
0.058460048	KMT2C
0.058636409	TMEM203
0.058636409	ITGAD
0.058810311	TRARG1
0.059058451	NUP85
0.059058451	EAPP
0.059102989	H3C13
0.059187825	PPRC1
0.059333971	LATS1
0.059333971	FLYWCH1
0.059333971	ATP6V1D
0.059418625	OTUD5
0.059418625	SSH2
0.059488923	ANKDD1B
0.059509185	H4C8
0.059510454	KLHDC10
0.059536939	GDF15
0.059584438	RGS3
0.059729339	ACAP3
0.059779346	MFAP3
0.059791515	CENPQ
0.059791515	YPEL2
0.059796111	DIS3L
0.059796111	RAPGEF2
0.059841129	NA
0.059881446	ARHGEF33
0.060179323	ZNF586
0.060260916	NA
0.060315785	QSOX2
0.060352265	H2AZ2
0.060352265	TMIE
0.060363761	NA
0.060363761	DCTN5
0.060380392	NA

0.060479296	UQCC2
0.060572033	RNF144B
0.060691853	ASS1P12
0.060691853	YY1AP1
0.060767939	BCL2L1
0.060767939	KIZ-AS1
0.060832515	NA
0.060832515	APLN
0.060977181	CTH
0.061004191	NKIRAS2
0.061004191	IL10RA
0.061049106	RRAD
0.061267756	SAMD1
0.061288776	SKIL
0.061288776	FARSA
0.061288776	PI4KB
0.061310743	NOM1
0.061585108	NA
0.061585108	KCNE5
0.061843049	NA
0.061843049	PEBP1
0.061945255	UTS2B
0.062365649	ATG16L2
0.062365649	NOC2L
0.062628024	TSPAN9
0.062628024	GASK1B
0.062628024	NA
0.062714819	ZNF350
0.062714819	SLC4A8
0.062714819	GARRE1
0.062714819	IKZF5
0.062757678	ZDHHC8
0.062772091	HMBOX1
0.062801017	UBAP1
0.062992346	WDR3
0.063033274	SLC39A4
0.063033274	DEK
0.063033274	OSM
0.063121975	DAG1
0.063132315	FGFR3
0.063183214	SMG1P3
0.063183214	KLHL25
0.063183214	PUS1
0.063359751	PKP4
0.063375402	PPARGC1B
0.063446169	PCSK9
0.063446169	KIF24

0.063446169	IQUB
0.063456909	SMARCD2
0.06346533	NA
0.063588197	ZNF765
0.063588197	LLGL1
0.063588197	NA
0.063658904	BACH1
0.063733141	NA
0.063888066	ATXN1L
0.063888066	HGD
0.063888066	GBA
0.063888066	LTV1
0.063904279	ANTKMT
0.063944559	GCNA
0.06400638	LINC01910
0.06400638	CLEC11A
0.06400638	NA
0.064346103	AUNIP
0.064383915	HSD17B3
0.064485252	ZNF275
0.064520212	LY6E
0.064676758	IMPDH1
0.064736231	DDX46
0.064835866	SECISBP2L
0.06492814	NA
0.064948484	RNF25
0.065064933	KCNN4
0.065112437	NAA30
0.065112437	ZRSR2
0.065112437	DCHS1
0.065141801	THADA
0.065141801	CARS2
0.065141801	FTX
0.065151764	HSD3B7
0.065151764	DPP7
0.065161427	ACAT1
0.065190011	SRRM4
0.065219	NA
0.065219	KDM5A
0.065229442	NBEAL2
0.065229442	NHP2
0.065229442	ITGAX
0.065229442	TNFAIP8L1
0.065229442	TRAF6
0.065261786	GOLGA2P7
0.065288141	GUCY1A2
0.065320095	C19orf48

0.065361137	BICD1
0.065391832	NA
0.065391832	NME2
0.065463667	DNAH11
0.065463667	NA
0.065463667	CHST14
0.065463667	SLC27A3
0.065506098	NA
0.065506098	C18orf25
0.065699191	ZSCAN10
0.065707868	FBN3
0.065726	NEDD4L
0.065726	CLN8
0.065726	PRKAB1
0.065809536	XPOT
0.065875337	RNF114
0.065875337	ZBTB34
0.065875337	SLC4A11
0.065875337	HTR1B
0.065974625	KCTD6
0.066219258	ELFN1
0.066221752	HCG25
0.066221752	NA
0.066326255	NA
0.066594287	CGRRF1
0.066855077	IFNA1
0.066876782	NA
0.066931154	SFXN4
0.067242863	COPRS
0.067242863	PRRT2
0.067242863	HCN3
0.067327486	RBBP8
0.067327486	UFD1
0.067327486	MYOM2
0.067327486	ULK1
0.067357696	SNORD63B
0.067395351	FAM20C
0.067395351	COX6B2
0.067572096	ADGRF3
0.067572096	ZNF416
0.06760761	MAP3K21
0.06760761	SNHG7
0.067695931	TES
0.067759977	UBA7
0.067759977	VIM
0.06802276	TRIM39
0.068182878	NA

0.068203046	TOMM5
0.068215812	JMY
0.068240918	LGMN
0.068331199	HMGN3
0.068331199	NA
0.068331199	ZSCAN9
0.068331199	PEA15
0.068463507	HYAL2
0.068567682	ZNF596
0.068633625	COQ10B
0.068633625	XRCC2
0.06863524	HAGHL
0.06863524	PLA2G4C
0.068837962	ACTL6A
0.068922644	ABHD14B
0.068922644	NA
0.069082569	TNPO1
0.069094946	TUBGCP3
0.069177462	NA
0.06923201	ADM5
0.06923201	MDM4
0.06923201	TMEM79
0.06923201	ZBTB10
0.06923201	NA
0.069278294	NUDCD1
0.069300653	XYLB
0.069303373	MIA2
0.069374408	SCG5
0.069374408	VPS54
0.069523818	GOLGA3
0.069834513	ARNTL2
0.069834513	TMEM81
0.069893953	USP16
0.069930368	HPS6
0.069930368	TIRAP
0.069930368	NA
0.069930368	NA
0.069930368	EFCAB5
0.069930368	MINCR
0.069976343	ALG2
0.069976343	NA
0.070174344	NUDT3
0.070335281	RTCA
0.070353848	PCYT1A
0.070472489	YWHAH
0.070472489	NA
0.070503648	MROH6

0.070503648	TNK1
0.070503648	MIR27A
0.070655534	TNFRSF8
0.070655534	NA
0.070736663	PNLDC1
0.070736663	FOXO3B
0.070780862	C1orf52
0.070834748	SERTAD3
0.070834748	UCP2
0.070834748	CALCB
0.070834748	NA
0.070845632	NA
0.071027961	ZNF440
0.071071299	ARFGEF1
0.071077719	GTPBP6
0.071077719	NA
0.071077719	TYW5
0.071107726	FAM157A
0.071107726	TMEM263
0.071135208	C9orf40
0.071135208	MBNL2
0.071135208	MAP3K13
0.071195752	GSTM3P1
0.071195752	P3H4
0.071311637	ZSCAN29
0.071395996	MIR425
0.071546504	HAUS1
0.071546504	NA
0.071546504	EIF2B3
0.071817411	OXLD1
0.07209449	NSL1
0.072126384	PLEK
0.072242459	ENAM
0.07228333	DNAJC9-AS1
0.072352957	APEX1
0.072352957	NA
0.072352957	FOXA3
0.072352957	SGCG
0.072413079	GPS1
0.072581755	HOXB8
0.072916016	TMSB15A
0.072916016	SMC6
0.072933264	TMEM201
0.07301563	SLC7A7
0.073088755	PLCB3
0.073088755	LOC285638
0.073088755	TMCO1

0.073088755	IDH2
0.073088755	NA
0.073188201	DBN1
0.073207076	ATP6V0A1
0.073207076	ZNF221
0.073240524	LOC101926913
0.073240524	NA
0.07326907	NXPH3
0.073364008	TMED9
0.073385359	RGMA
0.073385359	GEMIN6
0.073385359	THOC3
0.073418756	PAPLN
0.073504252	ARAP2
0.073534412	PNPO
0.073606808	MELTF-AS1
0.073606808	ZKSCAN5
0.073606808	BRAP
0.073895508	STEAP1
0.073895508	NA
0.073900179	VMP1
0.073900179	AADAT
0.074022704	RNFT1
0.074039467	RBFA
0.074083948	ATP6V0A4
0.074257612	DUSP9
0.07437596	HS6ST2
0.074384974	NA
0.074384974	RN7SL75P
0.074397394	PPP1R14B
0.074397394	GSTCD
0.074397394	NA
0.074441137	TSSK6
0.07447029	SLC26A6
0.07447029	SMS
0.07447029	ZGRF1
0.07447029	MED10
0.07452478	HELZ
0.07452478	SNAI1
0.074556448	H2BC20P
0.074865296	ALG6
0.074877362	NA
0.074909271	HSPB6
0.074917247	SAMD4B
0.074917247	SEC61B
0.074917247	TESMIN
0.074917247	ARL6IP6

0.074917247	TOM1
0.075128704	LOC105369980
0.075128704	MIR7-3HG
0.075128704	DVL2
0.075325211	CBX3
0.075445782	ZNF598
0.075445782	COL6A3
0.075489511	LINC00862
0.075489511	SLC35E4
0.075489511	TRIM28
0.075489511	H2BC5
0.075686709	NA
0.075690276	ASB13
0.075747269	HMCN2
0.075748676	SELPLG
0.075748676	SDCBP
0.075835681	ANKRD42
0.075835681	ULK4
0.075835681	LRFN1
0.075840376	MAP4K2
0.075919458	NA
0.075931094	NT5C
0.075932122	H2BC11
0.075932122	MALL
0.075998961	MFSD9
0.075998961	ALG11
0.075998961	FHOD1
0.076078151	JOSD1
0.076078151	NA
0.076078151	NA
0.076353251	HIC2
0.076353251	SIGLEC10
0.076353251	S100A3
0.076607274	GSR
0.07667638	SRD5A3
0.076705536	PDRG1
0.076708578	VPS9D1-AS1
0.076708578	GPATCH2
0.076709456	HDGF
0.076709456	HASPIN
0.076762836	NA
0.076762836	ANKRD11
0.076799658	ENDOG
0.076913292	TJAP1
0.077066545	PSMG1
0.07713303	ASB8
0.077233179	POFUT2

0.077274207	NA
0.077274207	MFSD4A
0.077289072	MOK
0.077289072	NA
0.077289072	APOBEC3C
0.077466162	SMPD1
0.077643972	CDC25B
0.077643972	UCP3
0.077701658	NA
0.077785475	RPUSD3
0.077785475	MMS19
0.077951955	B4GAT1
0.077951955	NAT14
0.078017503	RAD54L2
0.078131291	DYRK1B
0.078474996	SLC45A2
0.078634801	SCUBE2
0.078634801	TMEM127
0.078888544	ELOVL3
0.078925911	NA
0.078966222	PAFAH1B3
0.078978719	PMS1
0.078991025	THAP1
0.079087754	MANEAL
0.079153769	ZNF35
0.079406928	SLC35D2
0.079430149	TMEM115
0.079430149	RAD51D
0.079436438	C16orf74
0.079550592	NA
0.079604316	PCNT
0.079666189	LAS1L
0.07973659	HYAL1
0.079817112	ATP5F1B
0.079849202	TDRD5
0.079897928	POP5
0.079897928	TVP23B
0.079897928	NA
0.079911956	NA
0.079919389	SMOX
0.0801538	ZNF268
0.080351503	TARS2
0.080425398	VIM-AS1
0.080425398	NA
0.080498457	ACTL10
0.08054191	C1orf116
0.08054191	ATP13A2

0.080879697	FUT1
0.080879697	UQCRC1
0.081033791	MIDEAS
0.081033791	VWA1
0.081055467	ZNF461
0.081055467	DPYSL3
0.08125796	FAM171A1
0.081266176	FBXO48
0.081266176	FKBP15
0.081315927	FAM104A
0.081315927	TMTC4
0.081315927	PLGRKT
0.081367937	ARHGAP29
0.08150367	PRDX3
0.08150367	LINC00654
0.081557991	NA
0.081692028	PI4K2A
0.08184255	IL22RA1
0.08184255	LANCL1
0.08184255	NA
0.08184255	NA
0.081848247	DPH3
0.081848247	SEPHS1
0.081896725	AIFM2
0.081896725	RPUSD2
0.082112308	NA
0.082220364	LINC01134
0.082289544	GPBAR1
0.082289544	CCDC9
0.082349389	SFR1
0.082350866	UMPS
0.082352372	ASL
0.082352372	RBBP7
0.082367367	LIG4
0.082369474	POLR3H
0.082518904	LHFPL3-AS2
0.082578275	CKMT1B
0.0826058	WDR7
0.082851095	PRRT4
0.083102779	RPP40
0.083102779	TMEM107
0.083102779	ISG20
0.083159997	NA
0.083214026	LCA5
0.083280189	ERLIN1
0.083285941	CDCA4
0.083285941	NRIP1

0.083285941	AIFM1
0.083285941	RRAGC
0.083285941	RAB3GAP1
0.083285941	MTF1
0.083662795	AMBRA1
0.083662795	MRPS18B
0.083662795	ANKRD36
0.083662795	FKBP14
0.083842817	PELP1
0.083842817	CNTNAP1
0.083922421	H2AC6
0.083922421	ZNF775
0.083922421	UNC5A
0.083934103	ACTL8
0.084097329	PTCD3
0.084165242	PES1
0.084182182	CLCF1
0.084282643	CHCHD10
0.084282643	SELENOS
0.08438083	SUGP2
0.08438083	PKP3
0.08438083	KIAA0895
0.084430018	CEP41
0.084430018	SCN11A
0.084498261	LYPD6
0.084498261	ZNF264
0.084498261	P4HB
0.084517736	MZT1
0.084517736	NA
0.084528158	NA
0.084528158	NA
0.084616869	DERL2
0.084720068	CPSF1
0.084760445	MIR324
0.084788859	CDK13
0.084826344	NIPBL
0.084897006	PMP22
0.084898634	DTL
0.084898634	SEMA6B
0.084978422	NA
0.085171001	SLC35F5
0.085259798	XYLT2
0.085512584	FAM13B
0.085603743	TMBIM1
0.085631178	NIPAL2
0.085650855	DFFB
0.085690568	METTL27

0.085690568	PIGX
0.085782003	NA
0.085782003	TCFL5
0.085923073	TRMT61A
0.085923073	THEM6
0.086004985	CDC14A
0.086251773	FLVCR1
0.086261778	SLC45A4
0.086261778	SETD1B
0.086261778	APEX2
0.086261778	GCC2
0.086261778	PLAC8L1
0.086450432	PRPF18
0.086696616	PLA2G15
0.086847313	NUDT14
0.086847313	PLEKHA6
0.086847313	NA
0.086851707	ZNF618
0.086974346	RAB11FIP5
0.087092334	NA
0.087138914	SCMH1
0.087267376	NA
0.087297967	CFAP53
0.087347146	KLHL13
0.087451041	ANKRD49
0.087538035	SCPEP1
0.0875497	SENP5
0.0875497	NUP107
0.0875497	NFIL3
0.0875497	TBC1D30
0.087571542	KPNA4
0.087571542	FBL
0.087571542	EPDR1
0.087571542	NT5DC2
0.087603464	NA
0.087603464	BTAF1
0.087675841	BEX3
0.087881607	TLK2
0.088018234	ATG7
0.088018234	SLC44A2
0.088093077	LYRM1
0.088312008	DEDD
0.088339025	OSBPL6
0.088339025	FANCG
0.088339025	YIPF4
0.088344178	TENT5B
0.088344178	ARHGEF35

0.088497766	SLCO2B1
0.088513468	FBXO4
0.088543593	SEPTIN11
0.088543593	MRPS9-AS2
0.088543593	PNKD
0.088543593	ZNF211
0.088618363	CASC8
0.088640214	MARCO
0.088655097	PHLDB2
0.088729928	TMEM223
0.088756265	KIAA0232
0.088801451	LFNG
0.088888181	NA
0.08916331	C1orf35
0.089322178	IARS2
0.089322178	NA
0.089322178	DIO1
0.089322178	ACADS
0.089525764	HAUS6
0.089624191	NMT2
0.089701114	DPM3
0.089846872	RNF26
0.089873186	GMPS
0.089881575	PTPN18
0.08997341	SRBD1
0.090043549	ZNF655
0.090104544	PTGFRN
0.090113925	CBX1
0.090113925	NUTF2
0.090305464	GTF2IRD2
0.090310065	NA
0.090310065	SYTL3
0.090461118	TBC1D22B
0.090461118	TBC1D12
0.090481923	FAM222A-AS1
0.090698595	PRSS8
0.090725988	EVI5
0.090725988	ZNF304
0.090725988	IFITM10
0.091404072	ZNF181
0.091591429	FBRS
0.091591429	NPC1
0.091656297	PLCE1
0.091656297	FDXACB1
0.091781347	PLEKHA3
0.091822543	BCL2
0.091959302	PLCG1

0.092087111	PANK4
0.092087111	MTO1
0.092087111	UBTD1
0.092087111	AFG3L2
0.092199445	DOK7
0.09231731	ATP6V0E1
0.092424764	ZSWIM4
0.092424764	NA
0.09252641	VAV2
0.09252641	TVP23C
0.092607747	SMYD5
0.092607747	VDAC1
0.092607747	CCDC13
0.092761202	SNORD63
0.092761202	NA
0.092761202	ATP5F1D
0.092761202	NA
0.092761202	MKRN1
0.092853914	NA
0.092853914	DNAH5
0.092898399	NA
0.092917588	CRB3
0.093012249	LOC107984450
0.093071979	MKNK2
0.093083926	NT5C2
0.093083926	DMXL1
0.093254909	LZTS1
0.093258566	TMCO6
0.093258566	ASPSCR1
0.093334152	JMJD1C
0.093419268	PGM3
0.093460832	NA
0.093460832	ATP6V0D1
0.093460832	MIS12
0.093476058	RETREG3
0.093476058	TOB2
0.093476058	NA
0.093519128	EOLA1
0.093820422	GRIN1
0.093928966	ADORA1
0.094010895	ATR
0.094114008	RNF181
0.094243792	TEX19
0.094310154	DNASE1
0.094310154	LINC00943
0.094598705	NA
0.094614023	SH3BP5-AS1

0.094753817	PSMG4
0.094806145	NA
0.095068757	OR51B6
0.095276558	NA
0.095276558	TSC22D4
0.095597404	EBNA1BP2
0.095678984	IL10RB
0.095892187	KIF26A
0.095911235	TRPM4
0.095911235	DGKG
0.095911235	BOLA3
0.095978823	CBL
0.09598141	ILVBL
0.09598141	ZNF417
0.096157558	LRRK1
0.09622278	LYPD5
0.096398254	CCDC61
0.096398254	GPBP1
0.096398254	RIPK4
0.096437048	CARD8
0.096437048	SYNGAP1
0.096546703	CSNK1G3
0.096546703	HOTAIRM1
0.096572198	NA
0.096626246	NA
0.096626246	FAAH2
0.096663687	CCDC181
0.09672417	WDR49
0.09672417	TMEM240
0.096991538	H3C4
0.097041414	ELL
0.097041414	LOC102723566
0.097041414	LINC01115
0.097041414	LCMT1-AS2
0.097212033	GALNT18
0.097226041	AOC4P
0.097226041	NA
0.097226041	LOC100133315
0.097226041	PIN1
0.097407151	ARHGDIA
0.097407151	BAIAP2
0.097677981	KHNYN
0.097678839	ANP32B
0.097798974	HNRNPU
0.097798974	TBL3
0.097848844	LRP5
0.097848844	NA

0.097924948	GRK6
0.097924948	SACS
0.097987538	TAOK3
0.098032352	NRG1
0.098032352	TLCD3B
0.098044086	SMARCC1
0.098274983	ZNF814
0.098347477	NA
0.098347477	USP47
0.098459772	RNU6-216P
0.098459772	CTSL
0.098492062	FST
0.098550692	NA
0.098550692	CUTA
0.098550692	RTKN
0.098550692	CETN3
0.098559323	ELFN2
0.098559323	NAT10
0.098562783	NA
0.098694701	SLC35A2
0.098723412	ARFGEF3
0.09873911	DCAF5
0.099018878	MAP3K7CL
0.099170608	NA
0.099220967	MKRN2
0.099250703	LOC100379224
0.099358661	COL9A2
0.099415664	DHRS4L2
0.099415664	SH2D2A
0.099483399	SLC16A9
0.099606386	AFF1
0.099627802	USP31
0.099778691	RPL34-DT
0.099778691	LINC01589
0.099926679	DPH6
0.099926679	PIGZ
0.099926679	NA
0.099990232	NA
0.099990232	TMED5
0.099990232	ALKBH3
0.099990232	SUN3
0.099990232	KCTD10
0.099990232	NA
0.099990232	PSAT1
0.099990232	DIP2C
0.099990232	GGH
0.099990232	NA

0.099990232	FAM86EP
0.099990232	ZNF77
0.099990232	CDNF
0.100013631	ODAPH
0.100013631	SLC25A19
0.100013631	GPR107
0.100013631	PDE4DIPP6
0.100013631	NA
0.100257822	ZNF559
0.100363401	RUBCN
0.100412104	FPGS
0.10044542	NUTM1
0.100451	WIPF2
0.100451	FDXR
0.100470018	C3orf80
0.100787192	LSM6
0.100787192	ANKRD9
0.100920185	PGAM5
0.100920185	NA
0.101084806	ASH2LP3
0.101118121	TMEM109
0.101224838	NCKIPSD
0.10136784	MTMR9
0.101483343	PLEKHF2
0.101488479	MAFB
0.101488479	ZNF527
0.101575471	TMEM234
0.101595385	TPM2
0.101745381	EEF1AKMT1
0.1018365	ZNF782
0.101984454	UBQLN4
0.102049121	TAMM41
0.102111466	FAM102A
0.102111466	FAM217B
0.102249548	PPIL1
0.102249548	RO60
0.102249548	MSH3
0.102249548	XRRA1
0.102450394	TMA7
0.102450394	SPAG1
0.102642867	NA
0.102642867	NA
0.102646185	NA
0.10264686	HEATR1
0.10264686	C12orf50
0.102700927	DENND4B
0.102701385	NA

0.102704139	LOC648987
0.102704139	RAB2B
0.102898543	RPA3
0.102898543	MIR621
0.102949605	RC3H1
0.102949605	ZNF136
0.103174145	NKX3-1
0.103331844	ABCG4
0.103421236	NA
0.103439508	NA
0.103439508	NA
0.10344459	NIPSNAP2
0.10344459	NKAPL
0.103459421	ZMYND19
0.103459421	NA
0.103459421	TRPV1
0.103459421	LINC01278
0.103544529	WRAP73
0.10373098	OSBPL2
0.10373098	NA
0.10373098	GPD2
0.10373098	ARMC4
0.10373098	AQP8
0.103771058	BIRC2
0.103781263	CLEC4O
0.103781263	NELFB
0.103931165	GPR25
0.103931165	NXT1
0.103982773	ATG13
0.103982773	NPRL3
0.103982773	EXOSC7
0.104178247	NA
0.104291792	NKD2
0.104291792	CFL1
0.104327415	ZSCAN22
0.104709619	TRIM23
0.104709619	LINC02323
0.104771567	KRT18P31
0.104967994	BEX2
0.104967994	ALDH3B1
0.105053489	PYROXD1
0.105053489	ZPLD1
0.105053489	FASTKD5
0.105053489	AGPAT5
0.105090587	PRKACB
0.105213756	SRSF12
0.10523047	NOTCH4

0.105318699	CLCN2
0.105318699	GNMT
0.105318699	NA
0.105318699	NA
0.105411386	PATZ1
0.105802466	ALAS1
0.105827495	TCHP
0.105932602	LINC01267
0.106015061	MNX1-AS1
0.106215955	SLC38A5
0.106378259	NA
0.106379347	STAM2
0.106379347	SYF2
0.106379347	TMEM14A
0.106379347	TRIM52
0.106379347	RHPN1
0.10661719	KIF1B
0.10705913	RAP1GAP2
0.10713231	NA
0.10722977	ASB7
0.107258448	TMEM129
0.107265458	COA4
0.107265458	ZC3H12C
0.107361183	NSG2
0.107449501	NA
0.107449501	WDR77
0.107546544	AP4M1
0.107596012	GLDC
0.107634603	EFCAB13
0.107634603	COPB2
0.107772465	NA
0.107978024	TTYH1
0.108017047	ZMAT3
0.108208984	DYRK1A
0.108245941	AHCY
0.108545168	TMCC1-AS1
0.108545168	AKR7A2
0.10858314	SNORD60
0.10858314	NA
0.108650774	TARS1
0.108650774	APOL2
0.108650774	S1PR5
0.108650774	FUOM
0.108671714	NAXE
0.108704365	TERF2IP
0.108795832	DNAJC11
0.108795832	ABCA7

0.108921072	ZNF91
0.108921072	NA
0.108936997	CDKN2AIPNL
0.109441746	CTPS2
0.10953182	LOC257396
0.109552934	LSM14B
0.109881773	TNFRSF18
0.11013837	TANGO2
0.110177171	THAP5
0.110247177	TRIM68
0.110247177	GCAT
0.110247177	SBDSP1
0.11058453	TBC1D10A
0.110633556	FAM107B
0.110903749	MOV10
0.110969649	HSPA1L
0.110969649	BBIP1
0.110969649	TESK2
0.111042686	TCF3
0.111130283	MUTYH
0.111152442	PUDP
0.111152442	GPR61
0.111159921	EP300
0.111288474	CLTB
0.111365802	CSRNP2
0.111400649	CFDP1
0.111558525	ENOPH1
0.111558525	C9orf116
0.11157403	LRRC41
0.11157403	ZMYND15
0.111650307	TMEM225B
0.111650307	SRRM5
0.111661007	SLC6A17
0.111787426	TUBA4B
0.111787426	AFAP1L1
0.111814854	PHLDA3
0.111814854	NAXD
0.111862626	MGAT5B
0.111862626	HAUS7
0.111862626	LAD1
0.112155144	SHKBP1
0.112155144	EXOSC9
0.112155144	SNX31
0.112155144	NA
0.112155144	FEM1B
0.112155144	SLC6A8
0.112225667	NA

0.112341755	NA
0.112430262	NA
0.112824982	SIKE1
0.112948762	GTPBP3
0.113068414	SAR1B
0.113113177	NA
0.113113177	LOC100129931
0.113250658	ZCCHC8
0.113349202	CSNK2A2
0.113389906	LDLRAD3
0.113389906	HIPK1
0.113486143	ACHE
0.113525583	LUARIS
0.11390403	FMO1
0.113937659	GALNT11
0.113937659	GHITM
0.114186212	TRHDE
0.114186212	LOC100506302
0.114341966	NA
0.114356682	CAPS2
0.114356682	ZNF16
0.114388565	NA
0.114426115	CAPN15
0.114580653	PEX10
0.114580653	GOLGA2
0.114604247	PTPRVP
0.114604247	LINC01238
0.114604247	ENTPD5
0.114604247	TMEM156
0.114604247	IPPK
0.114604247	ZNF134
0.114737945	UBE2V1P5
0.114737945	INPP5K
0.114737945	LYG1
0.114737945	TRGV4
0.115148004	ABI1
0.115148004	NA
0.115287298	NA
0.115805241	SETD6
0.115805241	ZNF603P
0.11591001	NA
0.115922155	NA
0.115922155	PRDM6
0.115922155	RSL24D1
0.116035037	COMTD1
0.116104085	MCM8
0.116224656	TAS2R20

0.116224656	GTF2B
0.116224656	PHLDB1
0.116224656	FRZB
0.116224656	ADAMTS1
0.116321808	UBE2G2
0.116323361	RTEL1
0.116323361	FANCB
0.116367503	CNNM1
0.116475617	NA
0.116489	RNF111
0.116702214	CIRBP
0.116702214	NA
0.116702214	MEAK7
0.116702214	KIF1C
0.116702214	APTR
0.116702214	PCIF1
0.116702214	NA
0.116729316	NA
0.11678058	NA
0.116934048	TTLL7
0.116990544	TXNL4A
0.117023309	RFPL4A
0.117140721	MFF-DT
0.117140721	SMCR8
0.117164748	NA
0.117164748	RGPD8
0.117173357	DENND2B
0.117173357	NA
0.117173357	MPHOSPH6
0.117173357	DNAJA1
0.117173357	HNRNPAB
0.117173357	RTN4IP1
0.117237106	ACOXL
0.117237106	CCDC138
0.117237106	RPS10P11
0.117237106	GIGYF1
0.117303492	CD14
0.117406841	NA
0.117558091	ISOC1
0.117558091	GJB4
0.117808696	CDK5RAP3
0.117898359	IQCC
0.117960442	NA
0.118089045	DHCR24
0.118089045	HOXA13
0.118141751	NA
0.118156671	NA

0.118241919	FERMT1
0.118328072	MGAM2
0.118430064	SPOUT1
0.118452785	SLC25A38
0.118541968	SERPINI1
0.119114876	PPP1R26-AS1
0.119114876	RPL32P3
0.119114876	MMAB
0.119114876	UTP18
0.119171541	DOCK8
0.119178539	LINC00173
0.119178539	PSMC4
0.119178539	TFB2M
0.119382666	NA
0.119632093	HYLS1
0.119632093	SAT2
0.119632093	PSIP1
0.119638393	METTL4
0.119661932	NA
0.119840062	MLST8
0.11984506	ACVR2A
0.11984506	NA
0.119948628	SNRPE
0.120016401	DNAJB11
0.120049303	TRMT10A
0.120049303	NA
0.120077214	PYGL
0.120110158	C8orf88
0.120684332	REST
0.120688133	ZMAT5
0.120688133	NA
0.120784279	LNPK
0.120943222	RASL12
0.120943222	ATF6
0.120996053	NA
0.121028906	SKA2
0.121053944	FGFR4
0.121053944	CCM2
0.121065139	ATP5MF-PTCD1
0.121136586	ARG1
0.121136586	CCDC77
0.121175629	C15orf65
0.121203397	PLRG1
0.121355888	MRPL16
0.121448784	NA
0.121452335	ADAM32
0.121452335	LRP8

0.121665197	TTC17
0.121751587	TOMM40
0.121774823	NA
0.121774846	RAPH1
0.121801795	UNKL
0.121853292	DIXDC1
0.12187026	PRMT3
0.121987128	MPP2
0.121990546	GDE1
0.121990546	IL15
0.121990546	SDC4
0.122005811	LRRC8E
0.122005811	MBD1
0.122095568	PNRC1
0.122142824	BTN2A2
0.122236135	PAPPA2
0.122247142	PXMP4
0.122247142	TRIP4
0.122247142	ATP5F1A
0.122247142	NA
0.122247142	ACOT7
0.122303794	SELENOM
0.122303794	MCCC2
0.12231429	CREBBP
0.12231429	C1orf159
0.122391106	NA
0.122405164	POU6F1
0.122405164	NRG4
0.122490311	NA
0.122490888	PRSS23
0.122559774	SLC5A5
0.122584716	ANKRD27
0.122596183	DMKN
0.122650196	SERPINB7
0.122650196	RELT
0.122684334	NA
0.122755173	NA
0.122755173	ZC3H12D
0.122931723	NA
0.122931723	NA
0.122962169	VDAC3
0.122965978	PJA1
0.123059466	ACTG1P14
0.123176806	CXCL10
0.123176806	DNAJB4
0.12337378	TBC1D20
0.12337378	KIAA2012

0.12337378	NPW
0.12337378	COL5A2
0.123494053	RBBP4
0.123494624	N4BP2L2
0.123494624	LAGE3
0.123558842	GPRIN1
0.123774705	GPAM
0.123787268	CEACAM19
0.123948858	MIR4435-2HG
0.123948858	AGO3
0.12396449	NA
0.124038316	ATF7IP2
0.124194496	POLR1E
0.124327628	YEATS4
0.124327628	CDK7
0.12445266	CCDC106
0.124647196	ABHD14A
0.124647196	TTC6
0.124647196	NA
0.124843938	PSMA6
0.125162637	TRIP11
0.125180983	TBC1D16
0.125180983	ASB16
0.125204131	NUP133
0.125204131	NA
0.125204131	TRPM3
0.125204131	MRPL23-AS1
0.125204131	ZNF845
0.125204131	HEATR3
0.125246733	MAPKAPK3
0.125246733	PPP6R2
0.125250516	NA
0.125250516	OPTN
0.125250516	ARID4B
0.125250516	MED19
0.125250516	LTBP4
0.125250516	RBCK1
0.125250516	MVK
0.125565096	FAAP100
0.12562369	SAMD10
0.125697408	NA
0.125792199	UCHL3
0.125801196	CHM
0.125854311	GLI4
0.125854311	ZNF707
0.125888071	CCNG2P1
0.125893548	MESP1

0.125946614	RFPL2
0.125946614	TOLLIP
0.125946614	VN1R108P
0.125946614	C3orf38
0.125946614	AKT1S1
0.125946614	MAPRE3-AS1
0.126085515	ZNF112
0.126085515	TMEM88
0.126127325	WDR45B
0.126136931	PRKG1
0.126252386	ZNF692
0.126540468	CCNYL6
0.126577327	SEC61A1
0.126577327	LMTK2
0.126577327	DDX55
0.126577327	FAM111A
0.126678073	GOLGA7
0.126678073	NA
0.126678073	AHCYP2
0.126678073	NA
0.126678073	IDH1
0.126844719	NA
0.126844719	TSR3
0.126844719	NA
0.126844719	ZNF543
0.126988848	DDIT4
0.127179556	MRPL15
0.127355209	C6orf52
0.127404449	TIMM21
0.128177901	DMAC1
0.128177901	NA
0.128177901	CWC22
0.128177901	USP49
0.128252805	GLB1L
0.128533562	WDR6
0.128533562	NOLC1
0.12858008	MRI1
0.128597546	ANGPTL8
0.128693772	COQ9
0.128693772	ZNF875
0.128722361	OSGEPL1
0.128790065	ZNF225
0.128790684	SEPTIN5
0.128790684	OSTM1
0.128790684	FCGRT
0.128790684	SNORD104
0.128790684	NA

0.128849344	RNF19B
0.128858186	TTC41P
0.129018161	GLRX2
0.129145467	NA
0.129145467	ZNF230
0.129145467	NA
0.129153741	YPEL1
0.129355479	LOC392196
0.129482685	CRYBG3
0.129510523	NA
0.129585854	NSMCE3
0.129627191	EOLA2
0.129673423	NT5E
0.12984014	NPHP4
0.129882445	NA
0.129936864	TTC1
0.129951794	LINC01265
0.129951794	NA
0.130441324	NA
0.130547146	NKAPD1
0.130547146	MED13
0.130547146	FAM86C1P
0.130804054	LINC01123
0.130925733	STAM
0.131036496	CHP1
0.131161153	ZFX
0.13123593	NA
0.13123593	MMADHC
0.131277824	PLEKHG2
0.131486478	PSENEN
0.131748315	CANX
0.131806525	ZNF594
0.131924111	ALDH7A1
0.131924111	SERBP1
0.131940569	SPTY2D1OS
0.131968266	MYO16
0.13238571	VN1R110P
0.132440698	CDK2AP1
0.132529179	LINC02481
0.132739078	LINC01405
0.132742266	ZNF45
0.133061395	NA
0.133186633	TMEM106C
0.133191812	PAK1IP1
0.133191812	NA
0.133272092	MMP20
0.133272092	TRIM66

0.133394849	BTN2A3P
0.133509625	CLK2
0.133545225	ACBD3
0.133812597	ZNF431
0.133970123	NBPF1
0.133970123	ZNF92
0.133970123	NA
0.134052216	WAC
0.134132616	NA
0.134132616	WDFY3
0.134166952	TSEN2
0.134166952	MTHFD2L
0.134186555	AKAP17A
0.134186555	CGAS
0.134186555	ITPRID2
0.134186555	RHNO1
0.134209613	BDKRB2
0.134326123	NA
0.134359832	TACC2
0.13439979	CD200
0.134400522	BCL2L2
0.134509784	NA
0.134604134	UBXN1
0.134618906	CBLL1
0.135043316	TNFSF15
0.135246144	NA
0.135302666	NA
0.135555348	NA
0.135817825	BAAT
0.136095527	ECHDC3
0.136095527	NA
0.136147014	PLEKHS1
0.136249951	ANKRD53
0.136290967	POLR1C
0.136476987	HSD11B2
0.136476987	ECI2-DT
0.136494479	HBE1
0.136498965	BAK1
0.136498965	NA
0.136585999	PAXIP1
0.136597527	LOC100506282
0.136629393	LYRM7
0.136629393	GNAI1
0.136629393	FAM136A
0.136801261	ZMYM2
0.136814625	SLC7A5
0.136851106	RPGRIP1L

0.136885046	FBXO46
0.137297067	RBM7
0.137303659	RTF2
0.137303659	CDC6
0.137406985	ZNF81
0.137406985	PCBP4
0.137406985	EHMT1
0.137569654	LOC171391
0.137569654	METTL25
0.137573309	GALK1
0.137573309	NA
0.137612365	HSPA5P1
0.137612365	JMJD7-PLA2G4B
0.137704297	GGCX
0.137704297	FGF7
0.137704297	NUP58
0.13778191	ACY1
0.137845347	ZNF513
0.137963412	SUV39H2
0.137963412	CSNK1A1
0.137995827	LRRC4
0.138153053	LINC01484
0.138154237	USP30
0.138282876	MAMLD1
0.138282876	THAP2
0.138310003	ARRDC1
0.138310003	NA
0.13847497	BNIPL
0.138494101	PPP1R35
0.13858693	AAR2
0.13864329	CEP250
0.13864329	NA
0.13864329	RASGEF1C
0.13864329	ZNF821
0.13864329	SLC7A2
0.13864329	TTC27
0.13864329	CATSPERD
0.138690577	SUSD6
0.138834126	SORD
0.138834126	ERCC2
0.138902588	NA
0.138951526	TYRO3
0.138964676	OTULINL
0.139392319	ABHD17B
0.139683047	NOXRED1
0.139722762	TCTE1
0.139722762	AP4B1

0.139848763	SCN1B
0.139925933	ORC5
0.139925933	NA
0.139925933	SLC35B2
0.140020278	PARP11
0.140077253	ANGEL1
0.14016983	UBE2D3-AS1
0.140196032	CCDC78
0.140382419	MSANTD3
0.140382419	CASP16P
0.140499773	NIPA2
0.140731443	CALML4
0.141119268	GSKIP
0.141119268	SLC12A9
0.141208927	PHPT1
0.141356201	VRTN
0.141356201	MARCHF4
0.141356201	MOSPD3
0.141358049	CDHR3
0.141358049	TIMM13
0.141400152	NA
0.141400152	PGPEP1
0.141556199	HNRNPDL
0.141566335	MDH2
0.141566335	ZNF702P
0.141615891	NA
0.141695782	GAS2L3
0.141695782	BYSL
0.141695782	LMBRD2
0.141695782	PIP4K2A
0.141695782	RIPPLY1
0.141695782	QTRT2
0.141695782	CEP68
0.141695782	PLCB2
0.141695782	NA
0.141939442	MRPL57
0.141939442	EXOC6B
0.141939442	SLC9A3R2
0.141939442	DNAJC2
0.141939442	DNAJC27
0.142062265	PLAU
0.14219719	ASXL3
0.142230039	TRIP12
0.142738983	NAT1
0.142922744	NUDCD3
0.142974919	NA
0.142974919	PPP3CB-AS1

0.142974919	TTC12
0.143064444	FAM161A
0.143172166	ANAPC4
0.143254645	NA
0.143254645	SUCO
0.143254645	DCLK1
0.143334353	GPR83
0.143388161	NA
0.143415892	NA
0.14342029	CCDC130
0.143424984	SLC35F6
0.143469522	ATF5
0.143469522	LINC02365
0.143469522	MAN1B1
0.143469522	ARHGAP22
0.143469522	NA
0.143509194	NA
0.143579856	ZNF233
0.143579856	RLIM
0.143579856	HAGH
0.143625509	AACSP1
0.14362803	NPLOC4
0.143662754	DIABLO
0.143665152	NA
0.143740587	ZNF611
0.143831745	ZNF646
0.143862725	POLR2J4
0.143862725	FHL3
0.143862725	OSBP
0.143862725	NA
0.143862725	LINC00888
0.143862725	FYN
0.143862725	H2AZ1
0.143862725	RIOX2
0.143944346	NA
0.143944346	COMMD4
0.143944346	C12orf4
0.144094841	HES2
0.144147982	SERTAD4
0.144175329	KDM5C
0.144175329	RHBDL1
0.144175329	SLC4A7
0.144175329	HLF
0.144175329	STRIP2
0.144368029	EBP
0.144368029	DAZAP1
0.144520076	FBXW9

0.144652814	RBSN
0.144665389	NA
0.144671352	PDHB
0.14484596	NA
0.144913326	SCLT1
0.145017842	RPL12P37
0.145017842	ZC3H7B
0.145070035	NUDT16
0.145070035	HSD17B7
0.14518788	YTHDF3
0.14518788	SNORA11F
0.145303973	RN7SL145P
0.145589522	DIRAS1
0.145756882	SLC15A4
0.145763177	NA
0.146277335	SIAH2
0.146369389	NA
0.146401276	GBF1
0.146536617	NA
0.146536617	NLRP4
0.146691288	CAMLG
0.146706405	PEG10
0.146912916	NA
0.147030688	METTL26
0.147057678	TNFRSF12A
0.147057678	SPIN4
0.147057678	NA
0.147057678	POMT2
0.147057678	CNDP2
0.147057678	MYT1
0.147057678	PARP8
0.147057678	ASH2L
0.147057678	RAC3
0.147213739	ILRUN
0.147213739	SLC25A5
0.147213739	NA
0.147213739	TCF7L2
0.14735721	NA
0.147409132	TCIRG1
0.147792057	NA
0.148154638	LOC101927164
0.148154638	DIPK1B
0.148156954	SGMS1
0.148281893	ZDHHC9
0.148281893	KAT14
0.148561041	RBKS
0.148808879	ZNF776

0.14883449	C3orf62
0.148889442	NA
0.148889442	PINX1
0.148929784	NA
0.149170469	FUT5
0.149344887	PCNX2
0.149344887	NA
0.149344887	ST6GAL1
0.149344887	NAA60
0.149344887	ATP5ME
0.149344887	BTLA
0.149344887	JADE1
0.149344887	GZMM
0.149344887	NA
0.149344887	NA
0.149344887	PSPN
0.149496784	HIVEP2
0.149738418	GAB2
0.149738418	MRPL28
0.149738418	PGAP2
0.149774458	MRPL11
0.149800816	DDRGK1
0.149804584	SREBF2
0.149908007	MCAM
0.14993735	GCNT7
0.150095754	ARL8A
0.150095754	IVNS1ABP
0.150264209	ZNF263
0.150636612	GGT5
0.151263567	NA
0.151534406	PHF24
0.151534406	RABGEF1
0.151534406	NA
0.151767335	USPL1
0.151767335	METRN
0.151767335	LYAR
0.151897934	NWD1
0.151930417	RPE65
0.152242269	KIAA1191
0.152374805	C18orf21
0.152488879	RN7SL737P
0.152488879	LOC100422317
0.152488879	SPPL2A
0.152488879	MTFMT
0.152616941	STOX2
0.152616941	CLCN7
0.152616941	CENPH

0.152652548	RICTOR
0.152652548	LINC01119
0.152652548	RPS11P5
0.152652548	RTEL1-TNFRSF6B
0.152676064	MTA1
0.15307073	NUP155
0.153248201	NA
0.153297172	KRTAP4-7
0.153435121	AGAP3
0.153435121	SRP19
0.153435121	UTP14C
0.153435121	IPO11
0.153435121	FUT6
0.153482885	NA
0.153570665	AP5Z1
0.153570665	HNRNPA2B1
0.153570665	AP3S2
0.153595531	AMZ2P2
0.153595531	RADIL
0.153708599	ARHGDIB
0.153770772	ESD
0.153785997	FBXL15
0.153785997	NA
0.15396619	MYO5C
0.15396619	GPATCH1
0.15396619	TLR9
0.15396619	ABCA2
0.15396619	PITX1
0.154081947	ECT2L
0.154081947	NFKBIE
0.154081947	NA
0.154185066	MUC3A
0.154233219	NA
0.154359274	NA
0.154404656	MACROD1
0.154453912	NA
0.154537553	TUBBP1
0.154674073	GUSB
0.154674073	DOLPP1
0.154851048	LOC101928069
0.154853743	NA
0.155025947	RALGAPA1
0.155096179	KIFAP3
0.155340291	NXN
0.155492419	YAF2
0.155659599	RPSAP52
0.155659599	SIN3A

0.155755948	TMEM62
0.155893547	RSAD1
0.155935028	VEGFB
0.155935028	ZNF446
0.156049934	CCT2
0.156049934	KRI1
0.156053252	DGCR6L
0.156414732	EXOSC5
0.156599603	GLUD1P3
0.156599603	IMPA2
0.156692953	MTCL1
0.156706734	NA
0.156706734	EGR2
0.156706734	SH3GLB2
0.156706734	ERCC6
0.156964582	ZNF483
0.157107026	CCDC142
0.157207853	SOX30
0.157207853	FEM1C
0.157207853	VGLL3
0.157207853	TRAF3IP2
0.157483771	NA
0.157509786	NA
0.15754086	SIGLEC16
0.15756981	COL6A6
0.15787287	CPLX4
0.158132069	PTMS
0.158480397	PDCD5
0.158480397	CUEDC2
0.158507152	MTCH2
0.158557414	MARCHF5
0.15864031	IP6K1
0.15866688	NA
0.158732441	INTS12
0.158956044	WDR43
0.159189225	RAB22A
0.159363714	TRMO
0.159535163	FBLN1
0.159585979	SEMA3F
0.159742568	HDHD3
0.159742568	SEC14L2
0.159742568	ST7-AS1
0.15979184	ACSM4
0.159940388	NA
0.159940388	COX19
0.16008192	NA
0.160485533	PTPRS

0.160755226	SCNN1D
0.160755226	NA
0.160755226	NPDC1
0.160836696	TRAPPC2
0.161019998	ACCS
0.161198576	NA
0.161198576	NPM1
0.161371318	NA
0.161371318	SH3TC2-DT
0.161371318	UBE4A
0.161371318	RPSAP39
0.161371318	MMP1
0.161371318	RANBP10
0.161498358	AIDAP2
0.161660521	NUMB
0.161660521	RPL13AP12
0.161660521	ZNF451
0.161902321	CHRNA5
0.161902321	PPOX
0.161942326	H2BC18
0.161966307	TMEM64
0.162438482	NA
0.162444061	ARHGEF19
0.162481841	BST1
0.162548193	NA
0.162548193	ZMYM3
0.162551873	TEN1
0.162568012	LRRIQ3
0.162569406	ADAT2
0.162702267	LOC155060
0.162857116	PROX1
0.163043263	FGFBP1
0.163043263	PGAM1
0.163043263	C19orf38
0.163187728	LINC00867
0.163295473	ANKRD63
0.163298122	EPB41L5
0.163319858	DVL3
0.163516751	KNOP1
0.163565903	RBBP6
0.163565903	GS1-124K5.11
0.16372595	ATXN10
0.163806554	PTPRU
0.163946978	NID2
0.164247493	CASP7
0.164570372	NA
0.164592482	ACAP2

0.164598356	KIAA0930
0.16461813	NA
0.16461813	NA
0.16461813	DNAJC24
0.164618406	GPR19
0.164618406	NA
0.165088367	DNAJC25
0.165088367	CASP9
0.165094971	C7orf31
0.165102214	SLC25A24
0.165313182	FRMD3
0.165321107	DHRS4-AS1
0.165348198	DHRS13
0.165707348	LPIN1
0.165707348	NA
0.165707348	ZSCAN16
0.165823997	NA
0.165950388	SMIM24
0.166177135	FAM185A
0.166177135	NA
0.166177135	GSTO2
0.166185475	NA
0.166185475	RAVER1
0.166185475	KLF6
0.166310967	WDR20
0.166310967	NA
0.166318538	ZNF493
0.166589656	NA
0.166621703	C12orf75
0.166621703	TMCC2
0.166621703	GBP2
0.166621703	KIN
0.166833002	NA
0.166833002	GLB1L2
0.166837507	SLC27A5
0.166837507	NA
0.166922726	ATXN1
0.166964985	LETM1
0.167214695	GPATCH8
0.167383694	PCF11
0.167589869	REEP4
0.167593301	PKD1P5
0.167593301	RN7SKP160
0.167660237	CDC42SE1
0.167660237	NA
0.167660237	ZNF805
0.167660237	SLBP

0.167810552	NA
0.167810552	SYS1
0.167810552	HBA1
0.167810552	NEIL2
0.167885517	NFIX
0.167885517	RUNDC3B
0.168057073	RPL7AP60
0.168387426	EMC1-AS1
0.168479986	CACFD1
0.168479986	SLC9A5
0.168479986	PYY2
0.168665478	DIMT1
0.16868357	JADE2
0.16868357	UBE2A
0.16868357	SLC12A9-AS1
0.16868357	MEN1
0.168829987	RNU6-850P
0.168996368	NA
0.169001625	SPATA6L
0.169001625	FAM76A
0.169001625	TOR1AIP2
0.169067756	NETO2
0.169091756	KCTD12
0.169121965	ZXDA
0.169138662	MYRF
0.169138662	NA
0.169138662	ACTR5
0.169138662	STK32A
0.169138662	RELA
0.169242595	CPT1A
0.169242595	IST1
0.169242595	NA
0.169242595	NA
0.169328945	ZNF254
0.169328945	NT5M
0.169328945	SWT1
0.169373859	NA
0.169373859	MST1R
0.169455601	RPS2P53
0.169523324	CHRNA6
0.169598563	B3GNT6
0.169601321	BHMT
0.169601321	NA
0.169703088	RPS6KL1
0.169703088	NA
0.169703088	CLDN1
0.169719849	MTAP

0.169719849	NA
0.16972919	KCNJ14
0.169907527	USP6
0.170006943	ZNF131
0.170006943	C11orf54
0.170225846	TTC31
0.170281262	DUSP19
0.170344348	TMEM145
0.170548327	NA
0.170548327	BUB3
0.170548327	ECHS1
0.170684904	LINC01762
0.170723279	BRPF3
0.170812471	LRRIQ4
0.170899095	FCHO1
0.170899095	NA
0.170899095	GPD1L
0.170899095	KLF16
0.170934657	NA
0.170934657	SNRNP70
0.170934657	B9D1
0.170934657	UBB
0.171104451	SFMBT1
0.171121218	MAP3K2
0.171333535	HEPACAM2
0.171424931	EAF2
0.171476157	CDKL3
0.171476157	COX7A2L
0.171476157	ELF1
0.171625322	FAM83B
0.171625322	NA
0.171719886	SWI5
0.171719886	SMCO2
0.171763844	BRCA1
0.171763844	NA
0.171763844	ANKRA2
0.171763844	DCBLD2
0.171763844	CD37
0.171850349	EPC2
0.171860556	ZNF317
0.171903788	H1-3
0.172162514	BTNL9
0.172235015	DCTN4
0.172307677	SRPX
0.172307677	RDM1P5
0.172380476	SERPINC1
0.172386853	TKFC

0.172506901	NA
0.172680151	ISOC2
0.172680151	SLC6A20
0.172680151	SNORD20
0.172680151	DTD2
0.172734626	DPF1
0.172755979	YPEL4
0.173235433	ALOX12
0.173235433	ENGASE
0.173279247	WASHC2A
0.173591936	CKS1B
0.173597399	PTGR1
0.173606503	MAP11
0.173606503	ALMS1
0.173606503	TAGLN2
0.173606503	KCNQ4
0.173994579	ZBTB9
0.174086582	NA
0.174267237	MYBPC1
0.174297514	NA
0.174297514	MYC
0.174297514	TAF3
0.174297514	CLASP1
0.174297514	NA
0.174297514	SLC12A6
0.174297514	MMP24
0.174514901	ZNF454
0.174514901	SETD2
0.174651411	APOOL
0.174679078	UQCC3
0.174679078	FCSK
0.174713532	HTATSF1
0.174713532	STIL
0.175299817	NA
0.175299817	TRO
0.175415614	NA
0.175591775	ZBTB1
0.175591775	DENND1B
0.175596514	TRMT10C
0.175598995	IL17RB
0.175672429	FAM32A
0.175777192	LOXL1-AS1
0.175782287	PCDHGB6
0.175782848	TPST1
0.17583976	SP100
0.176152396	ITSN2
0.17621825	NA

0.17621825	PNPLA1
0.17621825	C11orf68
0.17621825	CLDN15
0.17621825	SDC3
0.17621825	NA
0.176305042	CHCHD4
0.176329218	OGN
0.176329218	GAS5
0.176329218	NFE2L1
0.176329218	NLRP2
0.176329218	ITPR3
0.176329218	NA
0.176329218	NRG2
0.176329218	FDFT1
0.176329218	NOC3L
0.176329218	RNU6-1157P
0.176478419	TENT5A
0.176478419	FAM171A2
0.176478419	SFXN1
0.176478419	CYP4F22
0.176478419	IL1RAP
0.176478419	LETM2
0.176712401	PEX11B
0.176712401	ETV4
0.176716899	CCDC85C
0.176808368	TDP1
0.177065879	NA
0.177065879	RNU1-91P
0.177088674	ENTPD1
0.177088674	NA
0.177153546	POLR3B
0.177153546	APPBP2
0.177164575	RGP1
0.177164575	PGBD2
0.177298437	UBR1
0.177325288	GJC1
0.177434494	SNORA79B
0.177434494	NR1I3
0.177523039	LINC00622
0.177562477	VRK3
0.177642161	NA
0.177642161	NA
0.177660733	THRAP3
0.177660733	RPS15AP36
0.177660733	SPINK1
0.177660733	NA
0.177660733	PXK

0.177660733	ZNF267
0.177660733	GPR4
0.177874666	L2HGDH
0.177874666	KLHL20
0.177975493	ANXA3
0.178010639	SLC25A22
0.178106317	DSTYK
0.178244417	DAP3
0.178261599	WDFY3-AS2
0.178261599	ANKRD16
0.178261599	TUBB8P1
0.17831779	NA
0.17831779	SPDYC
0.17831779	MICOS13
0.178427581	EMP3
0.178427581	APOF
0.178427581	CREB1
0.178427581	VPS45
0.178427581	MGARP
0.178427581	NA
0.178427581	GFUS
0.178427581	RBBP5
0.17852428	MARK3
0.178533995	JPX
0.178696389	DGKA
0.178707441	ATP6V0D2
0.178707441	ADGRB2
0.178707441	TRMT44
0.178728107	PTGR2
0.179098465	SMAP2
0.179176567	RNPEPL1
0.179176567	LPCAT4
0.179296709	MIER3
0.179361142	ARRDC2
0.179439211	NA
0.179556149	LOC100996333
0.179556149	RPA1
0.179843865	TRIM25
0.180060493	LSM2
0.180060493	CLEC18B
0.180060493	UBR2
0.180125592	ZNF8-ERVK3-1
0.180148743	NA
0.180395952	SAR1A
0.18043461	FAM98C
0.180482894	UBE2H
0.180482894	DCAF4

0.180586637	ABHD3
0.180586637	ZNF324
0.180586637	OR51B4
0.18089799	TADA2B
0.180966285	NA
0.181022944	TNKS2
0.181022944	LOC339666
0.181022944	ZNF675
0.181022944	ZNF350-AS1
0.181072157	MIR3648-2
0.181144491	NA
0.181144491	NA
0.181147054	NARS2
0.181187419	SEC14L3
0.181268515	NDUFAF3
0.181268515	BRIX1
0.181445981	ZNF581
0.181473545	MAB21L4
0.181482874	AKIRIN1
0.181482874	C12orf66
0.181482874	ATXN7
0.181482874	AKR7A3
0.181482874	PITX2
0.181482874	NA
0.181482874	LGR4-AS1
0.181549189	PCDHGA11
0.181549189	HECTD2
0.181549189	CREBL2
0.181724907	RAI14
0.181897272	NA
0.181904354	GABRD
0.181948427	ANGPT2
0.182129958	GLT8D1
0.182129958	C1QL3
0.182292069	HOXC8
0.182376596	PDE3A
0.182987361	ZNF799
0.183232989	FBXO11
